# Mid-Cenozoic climate change, extinction, and faunal turnover in Madagascar, and their bearing on the evolution of lemurs

**DOI:** 10.1186/s12862-020-01628-1

**Published:** 2020-08-08

**Authors:** Laurie R. Godfrey, Karen E. Samonds, Justin W. Baldwin, Michael R. Sutherland, Jason M. Kamilar, Kristen L. Allfisher

**Affiliations:** 1 Department of Anthropology, University of Massachusetts, 217 Machmer Hall, 240 Hicks Way, Amherst, MA 01003 USA; 2 Department of Biological Sciences, Northern Illinois University, DeKalb, IL 60115 USA; 3 Department of Public Health, School of Biostatistics and Epidemiology, University of Massachusetts, Amherst, MA 01003 USA; 4 Present Address: Department of Biology, Washington University, St. Louis, MO 63130 USA; 5 Department of Mathematics and Statistics, University of Massachusetts, Amherst, MA 01003 USA; 6 Present Address: USDA, APHIS, Riverdale, MD 20737 USA

**Keywords:** Primate, Diversification, Eocene-Oligocene transition, Mid-Cenozoic extinction, Colonization, Fossils, Evolutionary modeling, transoceanic dispersal

## Abstract

**Background:**

Was there a mid-Cenozoic vertebrate extinction and recovery event in Madagascar and, if so, what are its implications for the evolution of lemurs? The near lack of an early and mid-Cenozoic fossil record on Madagascar has inhibited direct testing of any such hypotheses. We compare the terrestrial vertebrate fauna of Madagascar in the Holocene to that of early Cenozoic continental Africa to shed light on the probability of a major mid-Cenozoic lemur extinction event, followed by an “adaptive radiation” or recovery. We also use multiple analytic approaches to test competing models of lemur diversification and the null hypothesis that no unusual mid-Cenozoic extinction of lemurs occurred.

**Results:**

Comparisons of the terrestrial vertebrate faunas of the early Cenozoic on continental Africa and Holocene on Madagascar support the inference that Madagascar suffered a major mid-Cenozoic extinction event. Evolutionary modeling offers some corroboration, although the level of support varies by phylogeny and model used. Using the lemur phylogeny and divergence dates generated by Kistler and colleagues, RPANDA and TESS offer moderate support for the occurrence of unusual extinction at or near the Eocene-Oligocene (E-O) boundary (34 Ma). TreePar, operating under the condition of obligate mass extinction, found peak diversification at 31 Ma, and low probability of survival of prior lineages. Extinction at the E-O boundary received greater support than other candidate extinctions or the null hypothesis of no major extinction. Using the lemur phylogeny and divergence dates generated by Herrera & Dàvalos, evidence for large-scale extinction diminishes and its most likely timing shifts to before 40 Ma, which fails to conform to global expectations.

**Conclusions:**

While support for large-scale mid-Cenozoic lemur extinction on Madagascar based on phylogenetic modeling is inconclusive, the African fossil record does provide indirect support. Furthermore, a major extinction and recovery of lemuriforms during the Eocene-Oligocene transition (EOT) would coincide with other major vertebrate extinctions in North America, Europe, and Africa. It would suggest that Madagascar’s lemurs were impacted by the climate shift from “greenhouse” to “ice-house” conditions that occurred at that time. This could, in turn, help to explain some of the peculiar characteristics of the lemuriform clade.

## Background

Several competing interpretations of lemur diversification [1–3] have strikingly different implications for the selective context of lemur evolutionary history. The first [1] is predicated on the assumption that lemurs were present on Madagascar ~ 50 million years ago when Chiromyiformes (Daubentoniidae) and Lemuriformes diverged [4]. It maintains that there was a major extinction followed by an explosive radiation of lemurs in the middle of the Cenozoic. This would have been triggered by the Eocene-Oligocene transition (EOT), long after the arrival of the common ancestor of all lemurs on Madagascar (see also [5, 6]).

The second [2] maintains that lemurs experienced positive diversification rates throughout their history; there was no explosive adaptive radiation, either following initial colonization of Madagascar or in the middle of the Cenozoic. Instead, diversification rates increased gradually through time likely due to increasing resource competition, ecological specialization, geographic isolation and/or habitat fragmentation. This second hypothesis implies that lemurs escaped the large-scale faunal extinctions that occurred globally during the mid-Cenozoic, or that such an extinction event did not occur on Madagascar.

Debate continues as to the likelihood of transoceanic dispersal by terrestrial (“dispersal disadvantaged”) vertebrates [7–10]. The third hypothesis [3] holds that Malagasy lemurs descend from two colonizations, each arriving after the initial divergence of Chiromyiformes and Lemuriformes on continental Africa. According to this hypothesis, the Daubentoniidae is sister to the extinct African Plesiopithecidae, which, like the Daubentoniidae, would therefore belong to the Chiromyiformes [3, 11, 12]. This hypothesis is plausible and intriguing, it but leaves open the question of arrival timing for ancestral Lemuriformes and Daubentoniidae, and of which lemurs if any were present on Madagascar during the EOT.

For our purposes, we will assume that the common ancestor of the Daubentoniidae and all non-daubentoniid lemurs was present on Madagascar 50 million years ago (following [1, 4, 7]), and we will return to the third hypothesis afterwards. Large-scale extinction is widely recognized as having occurred globally as the Eocene transitioned into the Oligocene. While minor in comparison to the five recognized “mass” extinctions, this event is accepted as the most significant trigger of faunal and floral turnover since the mass extinction at the Cretaceous-Paleogene (K-Pg) boundary and prior to the Pleistocene [13–21]. It affected marine and terrestrial animals, including primates, across multiple continents. A precipitous climate shift from “greenhouse” to “icehouse” conditions marked the Eocene-Oligocene (E-O) boundary at 34 Ma [22, 23]. There was a prolonged period of shallower temperature decline and aridification beginning ~ 45 Ma in the mid-Eocene [24, 25]. In North Africa and Asia, the EOT is believed to have played a pivotal role in the extinction of some primate clades [26–33].

While, due to Madagascar’s poor mid-Cenozoic fossil record, there is no direct evidence of the effect of the EOT on terrestrial vertebrates on Madagascar, it is reasonable to expect that it may have impacted this island’s fauna as well. Masters et al. [34] hypothesized that global cooling and drying may have contributed to the “apparently contemporaneous” divergence of both African and Malagasy non-daubentoniid strepsirrhines during the late Eocene and early Oligocene. However, Marivaux et al. [35, 36] questioned the significance of EOT impacts in the tropics; they showed that anomaluroid and hystricognathous rodents in North Africa maintained high diversity throughout this time. During the early Cenozoic and into the EOT when the Antarctic Ice Cap began building, Madagascar was more than 780 km south of its current location and thus less tropical than it is today; the northern tip of Madagascar at the time of the E-O boundary was ~19^o^S [37]. The question of the severity of EOT impacts on Madagascar remains open.

If the EOT affected lemurs on Madagascar, we would expect there to have been a sharp reduction in the number of lemur species at around the time of the E-O boundary followed by a recovery over the subsequent several million years. If there were minimal or no effects of the EOT on lemur diversification dynamics, then neither extinction nor speciation rates would have been perturbed, and we would expect no extinction-followed-by-recovery pulse. Other patterns, such as gradually-increasing lemur diversification over time, may have occurred.

These hypotheses regarding lemur diversification are very different. The near lack of an early and mid-Cenozoic fossil record on Madagascar has precluded their direct testing. It is impossible to compare terrestrial vertebrates before and after the EOT because of an approximately 65 million-year gap in the island’s terrestrial fossil record. Without such evidence, how can we shed further light on this problem?

One approach might be to examine the broader paleontological context for transoceanic dispersal during the early Cenozoic. Despite the lack of terrestrial vertebrate fossils from the early Cenozoic of Madagascar itself, there is a rich fossil record from the early Cenozoic of continental Africa. Molecular data support the inference that the source of Madagascar’s terrestrial vertebrates is largely Africa [38]. Transoceanic dispersal of dispersal-disadvantaged species may have been easier during the Paleocene and Eocene than later, as there were likely favorable currents from Africa to Madagascar at that time [8, 39]. Therefore, if the character of the modern and recent terrestrial vertebrate fauna of Madagascar bears little resemblance to the vertebrate fossils from the Paleocene and Eocene deposits of Africa, and if arrival dates are largely post-Eocene, this may be because a major extinction occurred during the EOT.

Using fossil data from vertebrate clades of the Paleocene and Eocene of continental Africa, we can address a number of simple but informative questions. Some of these clades are still extant while others are extinct. We can ask whether extinct and extant clades differ significantly in the extent to which they were geographically widespread in the Paleocene or Eocene. We can also ask whether they differ significantly in the degree to which they were dispersal-advantaged. If the answer to both questions is “no,” then we might expect extinct and extant clades to have dispersed at similar rates from Africa to Madagascar during the early Cenozoic. However, because Madagascar has virtually no early and mid-Cenozoic fossil record, the dispersal status of clades that are not represented in either the Late Pleistocene or modern faunas of Madagascar is unknown. Furthermore, if there are no significant differences in dispersal advantage or in geographic range of early Cenozoic African taxa that are today extinct or extant, then we can use the extant group to estimate the percentage of extinct clades that likely successfully dispersed to Madagascar during the early Cenozoic. This in turn would allow us to estimate how much of the dispersal record from Africa to Madagascar might be missing, possibly due to mid-Cenozoic extinction on the island of Madagascar.

Using fossil data, we can also examine the extent to which Madagascar’s Holocene fauna can be described as post-Eocene in character. A similar approach has been taken with regard to the K-Pg extinction on Madagascar; the fact that so few of the Late Cretaceous vertebrate clades also belong to Madagascar’s modern fauna has been taken as evidence of mass extinction in Madagascar at the K-Pg boundary [40]. Only one of 75 Holocene clades is present in Madagascar’s Mesozoic fossil fauna, and of the 19 known terrestrial vertebrate clades of the Late Cretaceous in Madagascar, only one survives. How does this compare to the survival of clades that likely arrived on Madagascar during the early Cenozoic?

A complementary approach would be to draw inferences on diversification and extinction of lemurs from computer-intensive modeling. Computer-intensive phylogenetic modeling has become a core component of research in macroevolutionary biology; indeed, this is what Herrera [2] used to defend his hypothesis of gradually increasing diversification rates in lemur evolution. However, this method has several weaknesses. First, computer-intensive modeling may be hampered by incomplete phylogenies. Lemurs lack a pre-Pleistocene fossil record in Madagascar, and the total number of extant lemur species is also under debate [41]. When phylogenies include a small number of species and particularly when they are incomplete, modeling inferences from different programs are more likely to be contradictory [42, 43]. A bigger problem, however, may be uncertainty in the estimated divergence dates and topology of known species. It is critical that the extinct lemurs be included as some extinct lemurs may be implicated in early splits [4, 12, 44]. Only a few phylogenetic analyses of lemurs to date have incorporated all three families of extinct subfossil lemurs (the Megaladapidae, Archaeolemuridae, and Palaeopropithecidae), and only two ([1, 12]) have produced divergence times for all three subfossil families, in addition to extant ones. However, there are fundamental differences in both divergence dates and topology between the two. Whereas both agree that the Daubentoniidae (Infraorder Chiromyiformes) and the ancestor of all non-daubentoniid lemurs (Infraorder Lemuriformes) diverged around 50 million years ago, the two trees differ in the amount of time that has elapsed between the tree root (divergence of Lemuriformes and Chiromyiformes) and the initial divergence of Lemuriformes (without *Daubentonia*). Thus, according to Kistler et al. [1], the Lemuriformes share a last common ancestor around 31 million years ago (95% CI 27–35 Ma), then radiated in fairly rapid succession. In contrast, Herrera & Dávalos [12] and Herrera [2] place the initial diversification of Lemuriformes earlier in the Cenozoic (at ~ 42 Ma) and much closer in time (9.448 Ma in File S1 of ref. [2]) to the initial split between stem Chiromyiformes and stem Lemuriformes.

The most important topological difference is in the placement of the Megaladapidae. According to Herrera & Dàvalos [12] and Herrera [2], *Megaladapis* is the sister to all other Lemuriformes and the Lemuridae is the sister to the Indriidae and their extinct relatives (the Palaeopropithecidae and Archaeolemuridae). Kistler et al. [1] support a sister-taxon relationship of *Megaladapis* and the Lemuridae and a sister taxon relationship for the megaladapid-lemurid and the indriid-archaeolemurid-palaeopropithecid-cheirogaleid-lepilemurid clades.

The goal of this paper is to evaluate the potential existence and impact on lemurs of a large-scale mid-Cenozoic extinction and diversification event on Madagascar, using multiple approaches, including comparative analysis of fossil and extant vertebrate databases and evolutionary modeling. First, we compared extinct and extant terrestrial vertebrate clades of Africa in the Paleocene and Eocene to determine how much of the dispersal record from Africa to Madagascar is likely to be missing. We then examined the resemblance of modern or subfossil terrestrial vertebrates from Madagascar to early Cenozoic terrestrial vertebrates from continental Africa and the degree to which the Malagasy fauna is post-Eocene in character. Next, we reexamined models of lemur diversification and extinction dynamics to determine whether modern phylogenetic modeling methods preclude a major lemur extinction event during the EOT, and whether they support instead a gradual increase in lemur diversification through time. We also asked to what extent our conclusion depends on the selected phylogeny or on the selected models: would we draw different inferences if we were to base our analysis on Kistler et al.’s [1] as opposed to Herrera’s [2] phylogeny? To address these questions, we tested our lemur diversification and extinction models on phylogenies generated by both.

**Fig. 1:**
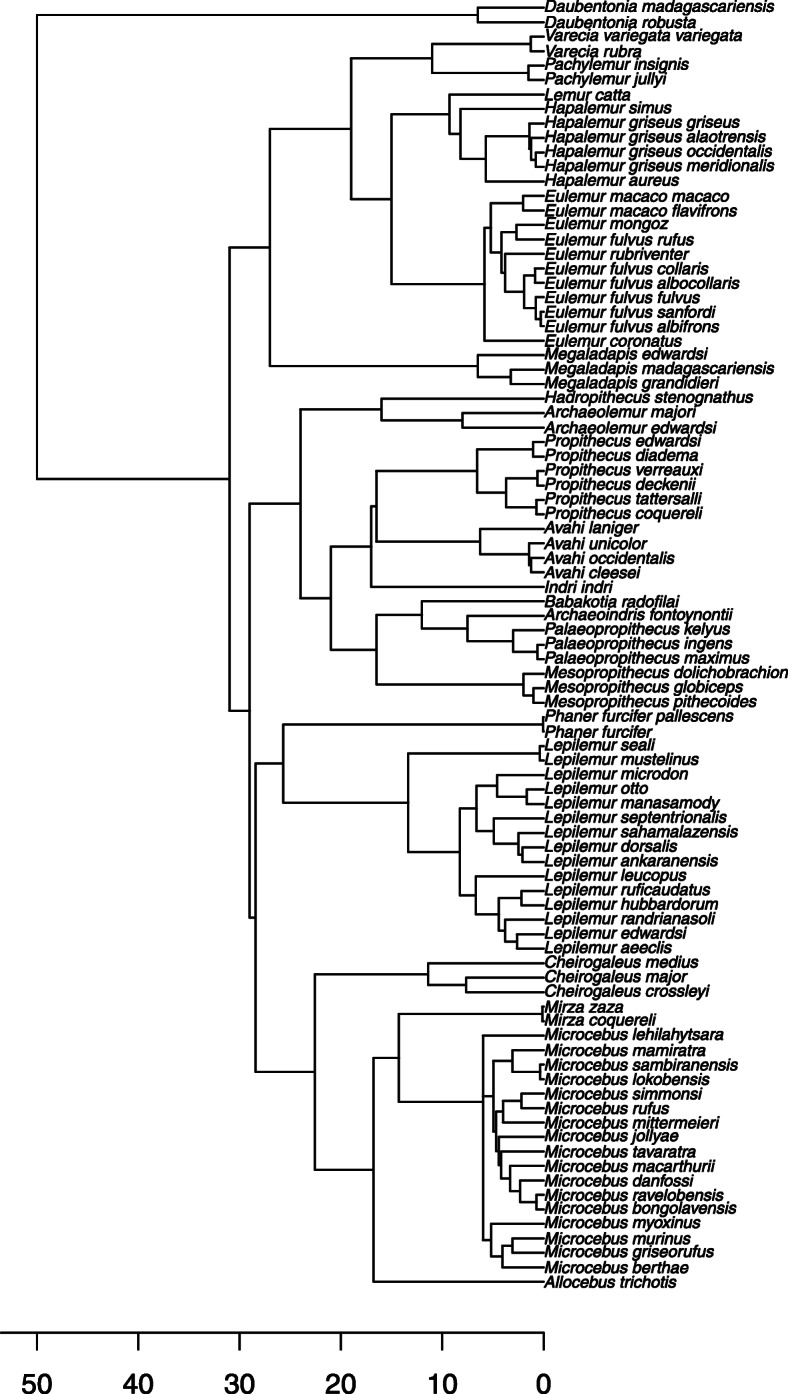
Phylogeny of lemurs used in this analysis, modified from [1]

**Fig. 2:**
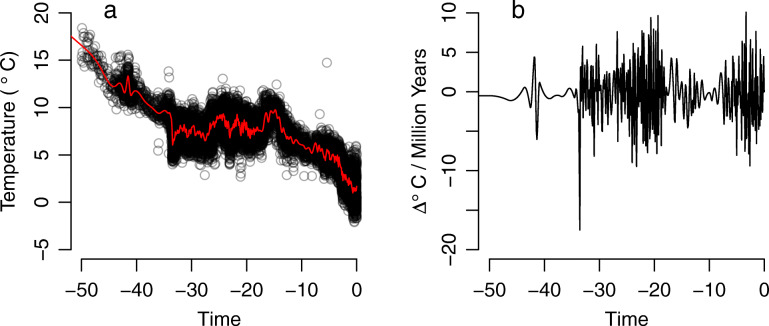
Changes in global temperatures during the Cenozoic. **a** Global temperature from 50 Ma to today. Smoothed values (red line) are generated by a 300-knot generalized additive model (GAM) with cubic splines of temperature over time. **b** Rate of change of global temperature from 50 Ma to today

There are, potentially, three reasons to favor the phylogeny of Kistler et al. [1] over that of Herrera [2]. First, preliminary research on the nuclear genome of extant and extinct lemurs [45, 46] supports inferences drawn by Kistler et al. [1] and not Herrera [2]. Secondly, the phylogeny published by Kistler et al. [1] has higher clade support for the node representing the last common ancestor of Lemuriformes than does that of Herrera [2] (= 0.77). Finally, a consensus phylogeny for extant lemurs generated by 10kTrees (downloaded October 29, 2018) is 100% consistent with familial relationships recovered by Kistler et al. [1] and not by Herrera [2]. This consistency meant we could easily expand the number of species included in our Kistler et al. [1] phylogeny without altering familial nodes or familial divergence dates (see Methods) (Fig. 1). To compare modeling results based on the two phylogenies, we limited our phylogeny based on ref. [2] to the same taxa included in our expanded Kistler et al. [1] phylogeny. Using both phylogenies, we tested a variety of hypotheses, including two that are quite specific: i.e., that lemur extinction rates were exponentially influenced by global temperature (which dropped precipitously at 34 Ma) or that extinction rates were exponentially influenced by the rate of change of global temperature (Fig. 2).

## Results

### Evidence from comparative analysis of vertebrate fossils

Seventy-five terrestrial vertebrate family (or superfamily) clades are known from the Paleocene or Eocene of continental Africa (Additional file 1: Table S1). Of those, 11 (14.7%) are believed to have likely colonized Madagascar prior to the E-O boundary (Table 1). This excludes taxa that could have arrived in the early Cenozoic, but genetic evidence affirms a later colonization. The best candidates for early arrival in Madagascar include reptiles (Boidae, Gekkonidae, and Scincomorpha) in addition to the Podocnemididae which were in Madagascar during the Cretaceous and were geographically widespread in the early Cenozoic (Paleocene of North and South America, Eocene of Europe) and the ancestor of the Lamprophiidae (which was likely an African colubrid). Mammals that likely arrived early include some bats (Myzopodidae, Nycteridae), the ancestor of the lemurs, and the ancestor of the now-extinct Plesiorycteropodidae (which may have been an afrosoricid). The Myzopodidae and Nycteridae have molecular divergence times within the Paleogene [47]; other bats known to have existed in Africa prior to the Oligocene may have arrived on Madagascar earlier, but none of the living members of these families appears to derive from colonizations during the Paleocene or Eocene. Amphibian fossils within the superfamily Ranoidea were recently found in Africa in the Eocene of Namibia. Finally, the Phaethontidae (tropicbirds) are known from the Eocene of Morocco. For some of the above groups we have supporting molecular evidence of early arrival.

**Table 1:** Holocene terrestrial vertebrate taxa of Madagascar that were present on continental Africa in the Paleocene or Eocene and that likely colonized (or whose close relatives may have colonized) Madagascar prior to the Oligocene, arriving either via vicariance or via transoceanic dispersal from continental Africa

African Taxon (Paleocene or Eocene)	Malagasy Taxon (Holocene)	Class, Order	Disp. adv.?	Fossils found where?
Podocnemididae	Podocnemididae	Sauropsida, Testudines	yes	Cretaceous of Madagascar, Eocene of Algeria and Egypt
Boidae	Boidae	Sauropsida, Squamata	no	Eocene of Egypt and Paleocene of Morocco
Colubridae	Lamprophiidae	Sauropsida, Squamata	no	Eocene of Egypt
Gekkonidae	Gekkonidae	Sauropsida, Squamata	no	Paleocene of Morocco
Scincomorpha	Scincomorpha	Sauropsida, Squamata	no	Paleocene of Morocco
Ranoidea^a^	Ranidae	Amphibia, Anura	no	Eocene of Namibia
Phaethontidae	Phaethontidae	Aves, Phaethontiformes	yes	Eocene of Morocco
Lorisoidea	Lemuroidea	Mammalia, Primates	no	Eocene of Egypt and Tunisia
Afrosoricida	Plesiorycteropodidae	Mammalia, Afrosoricida	no	Eocene of Egypt
Myzopodidae	Myzopodidae	Mammalia, Chiroptera	yes	Eocene of Egypt
Nycteridae	Nycteridae	Mammalia, Chiroptera	yes	Eocene of Tunisia

^a^Ranoidea, previously a superfamily, is now considered a major clade of Neobatrachian amphibians

All 11 early arrivals belong to families (or superfamilies) that are still extant (or were so in the Holocene, as evidenced by their occurrence in Madagascar’s subfossil deposits). There are 14 additional extant terrestrial vertebrate families (or superfamilies) from the early Cenozoic fossil record of continental Africa that are believed to have failed to colonize Madagascar in the early Cenozoic. Fifty early Cenozoic fossil terrestrial vertebrate families (or superfamilies) from continental Africa are extinct; whether they colonized Madagascar is entirely unknown. Table 2 compares frequencies of clades belonging to each of these categories. Of the extant (or Holocene) clades, almost half (44%) are believed to have colonized Madagascar during the early Cenozoic. Of the 50 extinct clades, none is known to have done so.

**Table 2:** Known pattern of early Cenozoic dispersal from continental Africa to Madagascar of extinct and extant terrestrial vertebrate families

Colonization history	Family extinct	Family extant	TOTAL
Believed to have colonized Madagascar during the early Cenozoic	0	11	11
Not believed to have colonized Madagascar during the early Cenozoic	50	14	64
TOTAL	50	25	75

We found that extinct and extant African Paleocene and Eocene clades *do not differ at all* in whether they were geographically widespread at that time (Fisher’s Exact Test *p* = 1.000, 2-sided), so there is no reason to assume that the extinct families would have been more restricted in distribution during the Paleocene and Eocene than the extant families. The difference in the degree to which extinct and extant clades were dispersal advantaged is greater, but also not statistically significant (Fisher’s Exact Test *p* = 0.111, 2-sided). Thus, there is no reason to expect differences in dispersal patterns for extinct and extant clades.

Therefore, if we expect that the clades that went extinct prior to the Holocene colonized Madagascar in proportions resembling those exhibited by clades surviving into the Holocene, then we should see frequencies like those in Table 3. The estimated number of clades colonizing Madagascar from Africa during the early Cenozoic is 33, of which we have lost evidence of 22 – i.e., two-thirds of the hypothesized early colonizing clades.

**Table 3:** Inferred pattern of early Cenozoic dispersal from continental Africa to Madagascar assuming similar probabilities of dispersal for extinct and extant terrestrial vertebrate families

Colonization history	Family extinct	Family extant	TOTAL
Colonized Madagascar during the early Cenozoic	22	11	33
Did not colonize Madagascar during the early Cenozoic	28	14	42
TOTAL	50	25	75

We are not arguing that all 22 “missing” clades would have gone extinct at the E-O boundary. On continental Africa, 11 of the 50 extinct families are known to have crossed the E-O boundary as they are present in deposits younger than the earliest Oligocene (see Additional file 1: Table S1). Thus, we can infer that up to (but likely much fewer than) 52% [(50–11)/75] of the African Paleocene or Eocene families (or superfamilies) may have disappeared at or around the end of the Eocene (i.e., they are extinct and there is no fossil evidence that they or a close relative survived the EOT). It is not unreasonable to assume a similar scenario for Madagascar. Of course, disappearance of a certain percentage of families or taxa of higher rank usually implies a much higher rate of extinction of individual species. For example, Raup & Sepkoski estimated that, at the K-Pg mass extinction, the loss of fewer than 20% of marine families translated into a loss of 75% of marine species [48]. Ward estimated that over 70% of terrestrial species disappeared during the largest mass extinction (at the end of the Permian) when the Earth lost over 90% of its marine species [49]. The percentage of families lost, however, was fewer than 60%.

Another approach is to examine the character of the Holocene clades on Madagascar (Additional file 2 Table S2). Of the 71 Holocene clades that we examined, 23 are believed to have colonized the island before the EOT; this is a third of Madagascar’s Holocene terrestrial vertebrate fauna. Only half of these are also known from the continental African fossil deposits of the Paleocene or Eocene; 48 appear to have arrived after the EOT, making the Holocene fauna predominantly post-Eocene in character (Table 4). Taken alone, this does not necessarily mean that there was a major extinction and turnover event during the mid-Cenozoic. Even if the probability of clade extinction per million years were constant throughout the Cenozoic, one would expect to see considerably greater extinction of early colonizers than late colonizers due to the fact that the early colonizers are “at risk” of extinction over longer periods of time.

**Table 4:** Known past and present terrestrial vertebrate clades on Madagascar, with time of arrival

Cretaceous clades	Holocene clades	
Ceratophryinae Bothremydidae Podocnemidae^b^ Mahajangasuchidae Notosuchia (twice) Trematochampsidae ?Cordylidae Dromaeosauridae Madtsoiidae (twice) Nigerophiidae Abelisauridae Noasauridae Nemegtosauridae Ornithurae Marsupialia Multituberculata Sudamericidae *N* = 19	Arrived pre E-O boundary	Arrived post E-O boundary
	Microhylidae (twice) Mantellidae Ranoidea^a^ Podocnemidae^b^ Chamaeleonidae Gerrhosauridae Opluridae Gekkonidae^a^ Scincidae^a^ Boidae^a^ Lamprophiidae^a^ Typhlopidae Xenotyphlopidae Aepyornithidae Mesitornithidae Phaethonidae^a^ Psittacidae (twice) Lemuroidea^a^ Myzopodidae^a^ Nycteridae^a^ Plesiorycteropodidae^a^ Arrived during the Eocene or earlier: *N* = 23	Hyperoliidae Ptychadenidae Testudinae Crocodylidae (twice) Gekkonidae (twice) Scincidae (twice) Lamprophiidae (twice) Acrocephalidae Apodidae Bernieridae Campephagidae Dicruridae Motacillidae Nectarinidae (twice) Pycnonotidae Strigidae Sturnidae Vangidae Zosteropidae (twice) Emballonuridae^a^ (3 times) Eupleridae Hippopotamidae (twice) Hipposideridae^a^ (3 times) Molossidae (> 6 times) Nesomyinae Pteropodidae (twice) Vespertillionidae^a^ (4 times) Tenrecidae Arrived during the Oligocene or later: *N* = 48

^a^Known (or close relative known) from Paleocene or Eocene deposits in Africa (see qualifier regarding Ranoidea, Table 1)

^b^Cretaceous and Holocene

One can model this, for example, if one assumes that both the probabilities of clade arrival and clade extinction per million years are constant throughout the Cenozoic. Such a simple model implies that only 10 to 20% of all clades arriving before the E-O boundary would be extant today. However, these assumptions may be violated if: (1) the probability of successful colonization were greater in the early Cenozoic due to currents being more conducive to transoceanic dispersal at that time (as appears to be the case for Madagascar [8, 38]); and/or (2) the probability of clade extinction decreases with clade size, which increases with clade age. Under these circumstances, one might expect the post-Eocene character of the modern fauna to be muted, unless clade extinction rates were much higher in the early Cenozoic, or unless there was a mid-Cenozoic extinction pulse. Long-term constant extinction is a powerful force but not a replacement for stochastic pulses of extinction.

### Evidence from phylogenetic models of lemur diversification

Using Kistler et al.’s phylogeny [1], BAMM generated a gradual increase in lemur diversification rates through time, from 0.017 at 50 Ma to 0.092 today. However, the estimated diversification rate carried uncertainty sufficiently large to encompass a time-invariant diversification estimate.

Using RPANDA, we fit four models of diversification that each allowed speciation and extinction rates to be either constant or time-varying. The models were indistinguishable from one another (maximum ΔAICc = 1.35) (Table 5).

**Table 5:** Four models in RPANDA, postulating constant or varying speciation and extinction rates

Speciation Rate λ	Extinction Rate μ	AICc	λ_1_	λ_2_	μ_1_	μ_2_
Constant	Constant	539.30	0.25		0.19	
Time-varying	Constant	540.07	0.23	−0.02	0.09	
Constant	Time-varying	539.67	0.22		0.11	0.02
Time-varying	Time-varying	540.65	0.23	0.08	0.22	0.08

**Fig. 3:**
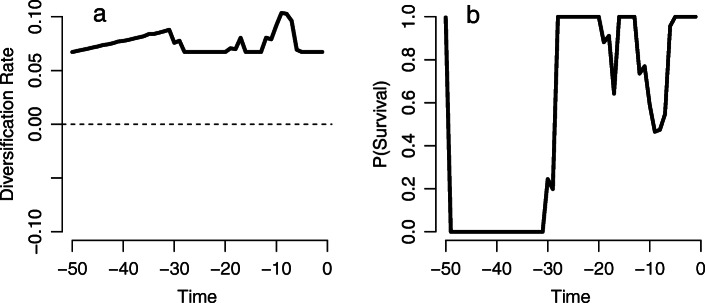
Modeling lemur diversification and probability of survival using TreePar. **a** Changes in diversification over time, when the model is forced to include mass extinction. **b** Probability of survival over time, when the model is forced to include mass extinction

Assuming that one “mass” extinction had occurred, we used TreePar to estimate changes in diversification rate and the probability of lineage survival, and thus as an indicator of the likely timing of significant turnover events (i.e., dips in the probability of lineage survival followed by peaks in recovery diversification rates). The diversification rate was estimated to be ~ 0.067 at the beginning and end of the series, with higher peaks at 31 Ma, 17 Ma, and 9 Ma (Fig. 3a), associated with the declines in estimated lineage survival. The probability of lineage survival was estimated to be near zero prior to 33 million years ago, after which it climbed rapidly to near one at 28 million years ago (Fig. 3b). Two subsequent dips in lineage survival were observed at 17 and 9 Ma, during which lineage survival declined to ~ 0.642 and ~ 0.465, respectively.

The estimated diversification rate from TESS showed a punctuated increase in diversification rates through time, from − 0.011 at 50 Ma to + 0.121 today. However, the uncertainty around the mean was sufficiently large to accommodate a time-invariant estimate of diversification that begins and ends at contemporary levels.

**Fig. 4:**
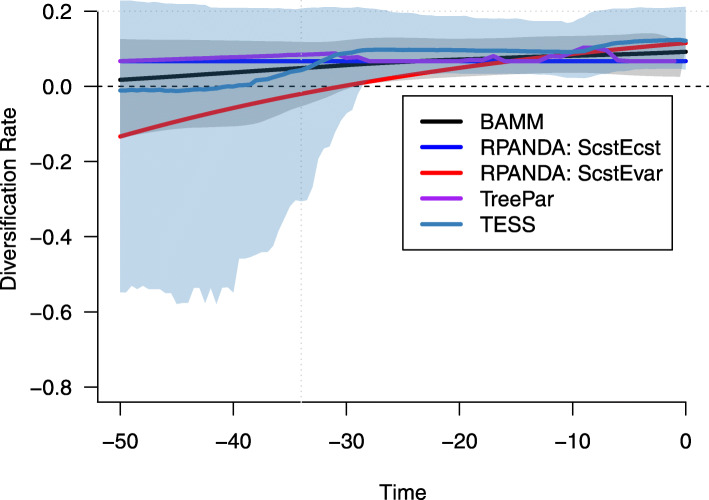
Composite modeling results (BAMM, RPANDA, Tree Par, and TESS) with credible intervals from BAMM (grey) and TESS (light blue). Scst = speciation rate constant (for the model); Ecst = extinction rate constant; Evar = Extinction rate variable

All of the modeling methods that we employed revealed considerable uncertainty among estimated diversification rates over time (Fig. 4). The credible intervals estimated by the Bayesian methods BAMM and TESS were very wide and encompassed all the mean diversification rate estimates from the maximum likelihood methods of TreePar and RPANDA. Moreover, the credible intervals were wide enough across the whole domain of time to fully encompass a straight line, thus rendering the estimated mean diversification rates statistically indistinguishable from a time-invariant (flat) diversification rate estimate. While the mean estimates of all methods suggest a minor increase in diversification rate through time, the rates have too much uncertainty to reject the null hypothesis of no change in diversification rate (a flat line). Indeed, a large number of diversification models, including those suggesting dramatic extinction followed by a recovery with a diversification peak, fit within the credible intervals produced by BAMM and TESS.

### Evidence from phylogenetic models of large-scale lemur extinction

**Fig. 5:**
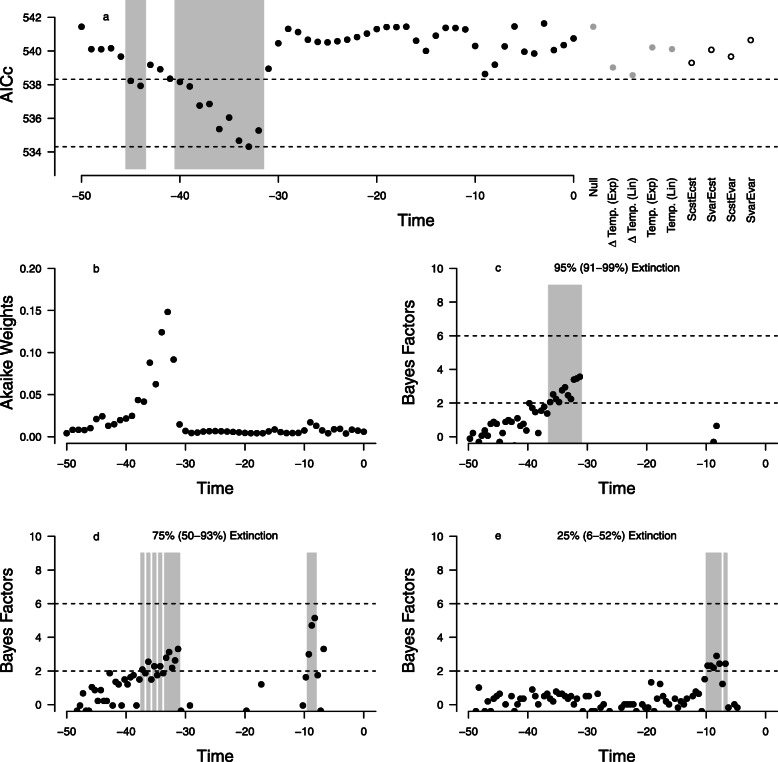
Comparison of profiles. **a** AICc profile of tests for large-scale extinction in RPANDA. AICc scores from models that feature constant speciation rates and allow extinction rate to vary as a function of an environmental variable. The model that had a sharp increase in extinction rate at 34 Ma was best but was statistically indistinguishable from other models with extinctions at similar times (black points on grey shading) and moderately better supported than all other candidate models (dashed lines indicate ΔAIC of 4). Grey points show AICc of models with temperature and its rates of change influencing extinction rate and null model; white circles show AICc of models with time-varying speciation and extinction rates. **b** AIC weight profile for models with extinctions compared to null model. **c** Bayes Factor profile for timing of extinctions with 95% extinction (95% CI: 91–99%). **d** Bayes Factor profile for timing of extinctions with75% extinction (95% CI: 50–93%). **e** 2 Bayes Factor profile for timing of extinctions with 5% extinction (95% CI: 6–52%). Grey shaded areas indicate times at which moderate amount of support for extinction is found

Using our preferred lemur phylogeny (based on divergence dates produced by Kistler et al., 2015), we employed RPANDA and TESS to test for large-scale extinction events. In RPANDA, the models that had raw temperature (Fig. 2a) (ΔAICc = 1.228 to null model) and the rate of change of temperature (Fig. 2b) (ΔAICc = 2.423 to null model) as predictors of extinction rate were both indistinguishable from the null model. Neither temperature nor its rate of change explained variation in extinction rate (Fig. 5a, grey circles).

In contrast, when we modeled unique time-varying punctuated (or “pulsed”) environmental perturbations, RPANDA revealed large differences. Our best-fit model (with the lowest raw AICc score) posited a diversification rate of 0.085 throughout, except during a several million-year period around 33 Ma where it dropped precipitously and then recovered. Models with simulated peaks predicting extinction at 45, 44 and 40–32 Ma were supported as well as the best model (ΔAICc to best model < 4, solid black circles between dashed lines in Fig. 5a). However, the best model was moderately better supported than all other models positing extinctions at other points in time (ΔAICc > 4, all circles above highest dashed line in Fig. 5a). The best model with the extinction peak at 33 Ma received moderately better support than the null model or than models that posited a correlation between extinction rates and either variation in temperature itself or its rate of change (ΔAICc > 4, grey circles above highest dashed line in Fig. 5a). It also received moderately better support than time-variant speciation and extinction rate models (ΔAICc > 4, white circles above highest dashed line in Fig. 5a). Our AIC weights show that the best model (extinction at 33 Ma) was 35.3 times more likely than the null model; the second best model (extinction at 34 Ma) was 29.6 times more likely. Looking across the Cenozoic, pulse model weights are low until ~ 45 Ma, when they begin a slow and then more rapid climb, peaking at 33 Ma before falling dramatically to values virtually indistinguishable from that of the null model (Fig. 5b). The probability that extinction at 33 Ma is preferred over the null model is 97%, and the probability that models positing extinction between 36 and 32 Ma are preferred over the null model is 99% (Additional file 3). This is true despite the fact that no extinction pulse can be said to be “strongly” supported. The only other models with moderate support were those positing an extinction pulse at 9 Ma or 8 Ma (when the model weights show these pulses to be 4.1 and 3.1 times more likely than no pulse).

We also repeated the analyses summarized in Fig. 5a, but now we treated environmental variables (absolute temperature and rate of change in temperature) as predictors of speciation rates. Model uncertainty was much higher and there was much weaker evidence favoring any single model over any other (Additional file 3).

The test of large-scale extinction in TESS showed a result qualitatively similar to RPANDA. There was moderate support for an extinction at 31.25 Ma (Bayes Factor = 3.56; 5% survival, 95% CI 0.01–0.10) (Fig. 5c). Of all potential extinction events, an event at 31.25 Ma was best supported. However, these results were sensitive to our choice of prior. When we decreased the magnitude of extinction and increased uncertainty about extinction magnitude (Figs. 5d and e), we found less support for an extinction event near the E-O boundary. Allowing a less dramatic extinction (25% survival, 95% CI 0.07–0.50) to occur, we found moderate support for its placement at either the E-O boundary or near 9 Ma (Fig. 5d). Allowing an even less dramatic extinction (75% survival, 95% CI 0.48—0.94) to occur, we found moderate support for its placement near 9 Ma (Fig. 5e).

The phylogeny generated by Herrera [2] yields results that are qualitatively similar to those obtained from Kistler et al. [1]. Both suggest a moderate amount of evidence for a large-scale extinction, but only when the extinction is constrained to be massive and with low uncertainty (Additional file 4). Moreover, both analyses place the most likely time for major extinction event as immediately prior to the diversification of the Lemuriformes (excluding *Daubentonia*) (42 Ma for [2], 33 Ma for [1]). The main difference is that there is greater model uncertainty associated with the placement of the extinction at 42 Ma. The weaker support for a large-scale extinction event generated when using Herrera’s phylogeny [2] is likely related to the shorter time between the initial divergence of Chiromyiformes and Lemuriformes and the last common ancestor of the Lemuriformes.

## Discussion

We draw two primary inferences from our results: first, the African fossil record does provide support for the inference that Madagascar suffered a major mid-Cenozoic extinction event that affected its terrestrial vertebrate fauna; and second, the modeling evidence supporting “gradually increasing diversification of lemurs” and “no unusual extinction event in the mid-Cenozoic” is inconclusive. All “best” reconstructions of changes in lemur diversification rate over time fit within the BAMM and TESS credible intervals, but they draw very different conclusions regarding the temporal pattern of change. We interpret this to suggest that modeling alone does not provide a single robust solution, i.e., that modeling results obtained from time-calibrated phylogenies do not conclusively resolve diversification dynamics, in line with other recent results [50].

Like Herrera [2], we found that the mean estimates of all methods suggest an increase in lemur diversification rate through time. This overall trend can be explained, as Herrera [2] suggests, by increasing resource competition, ecological specialization, geographic isolation and/or habitat fragmentation. Such a trend does not preclude extinction/recovery pulses, however. TreePar, operating under the condition of mass extinction, found a spike in diversification at 31 Ma with low probability of survival for lemurs on the island prior to this spike. However, TreePar has built-in assumptions that make it less compelling as a modeling tool than is RPANDA or TESS.

Model comparisons using AICc scores (RPANDA) found likely large-scale extinction between 40 Ma and 32 Ma; this covers the period from the mid-Eocene to the end of the EOT. AIC weights (RPANDA) identified the period between 36 and 32 Ma as showing the greatest probability of extinction. Support for a large-scale extinction between 31.25 and 36.25 Ma using Bayes factors (TESS) was moderate.

If we model extinction pulses as small dips in lineage survival, then we find evidence for such perturbations at ~ 17 Ma (TreePar) and between 9 and 8 Ma (TESS, TreePar) – i.e., during the early and late Miocene respectively. Like Herrera [2], we could not reject the null hypothesis of “no mass extinction.” However, we found that the null hypothesis provides a poor description of the observed data and that some candidate pulse models, particularly those positing extinction during the EOT, fared moderately better.

There may have been a threshold temperature drop or pace of drop that had to be exceeded before large-scale extinction would occur. It is suggestive that our two best supported extinction models posit high extinction during or just after the most rapid temperature drop during the entire Cenozoic (Fig. 2b, temperature drop of 17 °C per million years). Certainly, however, there is no simple correlation between the pace of temperature decline and extinction rate.

Finally, we found weaker support for a large-scale mid-Cenozoic extinction when we employed the phylogeny used by Herrera [2] rather than by Kistler et al. [1]. These phylogenies differ mainly in their assessment of the timing of divergence of *Megaladapis*. Resolving the divergence date for the Megaladapidae may be key to understanding the timing of the origin of some shared derived traits of the Lemuriformes. If Herrera [2] is correct that the Megaladapidae diverged from other lemuriforms 42 Ma (so the LCA of the Lemuriformes dates to the mid-Eocene), the environmental context for the origin of lemuriform synapomorphies would be very different from the alternative scenario, where the LCA of the Lemuriformes dates to around the time of the EOT.

The most compelling argument for large-scale mid-Cenozoic extinction of terrestrial vertebrates on Madagascar comes from the fossil record. This argument references differences between extinct and extant vertebrate families (or superfamilies) from the Paleocene and Eocene of continental Africa and how they relate to Holocene vertebrate families on Madagascar. The extinct group has twice as many families (or superfamilies) as the extant group. While nearly half of the extant families are believed to have colonized Madagascar in the early Cenozoic, the lack of early Cenozoic fossils in Madagascar precludes any knowledge of pre-Oligocene arrival of extinct taxa. If the now-extinct clades crossed the Mozambique Channel during the Paleocene or Eocene with the same relative frequency as the extant clades, the implication is clear: we have lost evidence of two-thirds of the terrestrial vertebrate clades colonizing Madagascar from Africa at that time. Because the extinct and extant families do not differ statistically in the degree to which they were geographically widespread or dispersal-advantaged, we cannot attribute the observed difference to variation in geographic spread or transoceanic dispersal ability of these groups.

This suggests that the percentage of terrestrial vertebrate families or superfamilies from Africa believed to have colonized Madagascar during the early Cenozoic (i.e., 14.7%) may be grossly underestimated. The actual percentage may be much higher, but, as on the continent, the EOT may have precipitated high faunal turnover on Madagascar. This may explain why the Holocene terrestrial vertebrate fauna of Madagascar is largely post-Eocene in character. Presumably in Madagascar as well as on the continent of Africa [51], increasingly arid ecosystems replaced warm, lush forests after the Eocene. Buerki et al. [52] argue on the basis of genetic evidence that the plant communities of Madagascar changed significantly at that time, and many of the taxa adapted to Madagascar’s most harsh arid habitats likely arrived or radiated after the end of the Eocene.

We suggest that at 31 Ma, the ancestral lemuriform had evolved a number of traits that can be understood as adaptations to the global cooling, forest reduction, and forest fragmentation that occurred during the EOT. These include adaptations to minimize energy expenditure, maximize energy intake from foods of varying quality, and survive episodes of food scarcity whether seasonal or due to environmental resource unpredictability. Some are preserved in the morphology or chemistry of bones of extinct animals – for example, in strontium isotopes which suggest that the extinct lemurs had small home ranges [53], in skeletal adaptations for slow locomotion [54–56], or in the relative size of the semicircular canals which offer independent confirmation of reduced activity levels [57, 58]. The ancestral lemuriform exhibited high folivory [59], “lemur syndrome” adaptations such as reduced sexual dimorphism and dominance of adult females over males [60], and depressed Retzius periodicity [5] (the latter perhaps associated with risk-averse life histories). Folivores are able to survive on less desirable, low “quality” foods, when necessary.

## Conclusions

In conclusion, direct comparisons of the terrestrial vertebrate faunas of the early Cenozoic on continental Africa and the Pleistocene and Holocene fauna of Madagascar support the inference that a major terrestrial vertebrate extinction/recovery event may have occurred on Madagascar during the EOT. Of the terrestrial vertebrate clades that arrived during the Paleocene or Eocene from continental Africa, it appears that we have lost evidence of two-thirds of them. Not all of these would have become extinct during the EOT. However, if the EOT on Madagascar resembled that on continental Africa, then up to ~ 50% of the terrestrial vertebrate families (or superfamilies) that were present on Madagascar in the early Cenozoic may have disappeared at or around the end of the Eocene. Those families that did not disappear may have nevertheless suffered dramatic species loss. The fossil evidence is compelling and it suggests that there may have been very few lemur species on Madagascar near the end of the Eocene (prior to the initial radiation of Lemuriformes). This is certainly consistent with results that can be derived from modeling lemur phylogeny. It suggests that the global climate shift from “greenhouse” to “ice-house” conditions that occurred during the EOT may help to explain shared derived characteristics of the lemuriform clade and subsequent specializations of particular families.

Our research demonstrates that modeling speciation and extinction based on lemur phylogeny alone does not exclude the possibility of a major mid-Cenozoic extinction and recovery event involving these animals, and may indeed support it. However, the strength of support via modeling for a mid-Cenozoic extinction depends on the phylogeny selected. If the phylogeny preferred by Herrera [2] is used, there is no support for a major extinction event during the EOT. There is, instead, weak support for a major extinction at around 42 Ma. This also implies that the peculiar shared derived traits of the Lemuriformes (including the Megaladapidae) would have evolved before the climate shift associated with the EOT.

Even when Kistler et al.’s [1] phylogeny is used, evolutionary modeling produces different predictions depending on the specific model assumptions applied. We found the greatest support for a major extinction (and subsequent recovery) event at 33 Ma, but we also found some support for a major extinction (and subsequent recovery) event at 17 Ma and again at between 8 and 9 Ma. Extinction during the late Miocene (at 8–9 Ma) is interesting because environmental stressors in Africa at that time resembled those of the late Eocene. On continental Africa, global cooling during the middle Miocene as well as marked temperature decline and aridification during the late Miocene resulted in the spread of open, grassy biomes and high terrestrial vertebrate turnover [51]. In contrast, the early Miocene was warm and wet; extinction at 17 Ma is unexpected on climatic grounds. However, it may have resulted from the slightly earlier arrival and initial diversification of euplerids (carnivorans that became skilled lemur predators [61]).

A final caveat is in order. There is a possible alternative explanation for lemur bottlenecks at 33 Ma and again during the Miocene. As stated in our introduction, whereas a single colonization of Madagascar by the ancestor of all lemurs is widely accepted, recent research has defended the possibility of separate colonizations of Madagascar by the ancestors of the Daubentoniidae and all other lemurs [3, 11, 12]. If the divergence of Chiromyiformes and Lemuriformes occurred on continental Africa, then these two clades would have diversified independently on Madagascar, after their separate arrivals. Those colonizations could have occurred long after the initial divergence on continental Africa of Chiromyiformes and Lemuriformes; indeed, Gunnell et al. [3] argue forcefully for a Miocene arrival of the ancestor of the Chiromyiformes. The ancestor of the Lemuriformes would have arrived earlier, as molecular evidence strongly supports diversification of Lemuriformes during the EOT. However, separate colonizations means that there could have been a very limited number of lemur species present on Madagascar at 31 Ma (not long after initial arrival), and those species would have had to adapt to the environmental conditions that existed at that time if they were to survive. Ironically, the result for models of lemur diversification forced to begin 50 million years ago would be evidence of bottlenecks in both main lineages, associated with their respective colonization events. Colonizations would resemble mass extinction/recovery events, even if no mass extinction had occurred. Thus, independent colonizations of Lemuriformes and Chiromyiformes respectively could explain our observed modeling support for “extinction” (or bottlenecks) in our lemur phylogeny around 33 million years ago (EOT) and during the Miocene.

## Methods

### Fossil evidence of a large-scale extinction and turnover event

To examine fossil evidence of mid-Cenozoic terrestrial vertebrate extinction in Madagascar, we created two databases (Additional file 1 Table S1 and Additional file 2 Table S2). The first reflects our current understanding of the biogeographic history of African Paleocene and Eocene vertebrate faunas; this addresses potential source clades present during the Paleogene for dispersal events from Africa to Madagascar. For each family or superfamily we recorded overwater dispersal ability, whether it was geographically “widespread,” persisted after the Eocene, and colonized Madagascar. Information was compiled from the literature as well as multiple online sources (http://fossilworks.org; https://paleobiodb.org).

Our second database (Additional file 2) comprises Malagasy terrestrial vertebrate clades (groups of Malagasy species descended from a single ancestral species that arrived on Madagascar either via transoceanic dispersal or vicariance). Each clade was coded for class, time of arrival (as inferred in the literature), and overwater dispersal ability. This database includes clades known from the Late Cretaceous and Holocene, as well as modern vertebrates with no fossil record.

### Generating our phylogeny

To expand the phylogeny obtained from Kistler et al. [1], we used divergence dates for extant lemurs derived from the 10kTrees website [62]. Our approach was modeled after others who have used a broad phylogeny as the backbone for their research and then added species based on additional information [63–67]. Upham et al. [68] provide a detailed comparison of “backbone and patch” vs. “supertree” approaches to phylogeny construction, and show that the two approaches yield broadly concordant node ages.

We downloaded 200 phylogenetic trees from the 10kTrees site to derive a single consensus phylogeny for the extant lemurs included on the website. The 10kTrees online resource allows users to produce up to 10,000 different phylogenetic trees, each with slightly different branch lengths that are determined using the Bayesian phylogenetic analysis method of Metropolis-coupled Markov chain Monte Carlo (MC^3^) algorithms. This method compensates for parameter uncertainty by sampling most often from parameter space with the highest posterior probability, while also accounting for the imperfection of parsimonious assumptions in biology by including some samples with lower posterior probabilities.

We used the divergence dates reported by [1] for all clades comprising at least some extinct taxa, when known. For clades containing only extant taxa (which were invariably far better represented in 10kTrees than in [1]), we used the 10kTrees divergence dates (Table 6). This was easy to do because their topologies were 100% congruent for the species they shared in common. Furthermore, all divergence dates in our 10kTrees consensus phylogeny for nodes that also appeared in Kistler et al. [1] fell squarely within the confidence limits of the corresponding dates in [1]. With the exception of the deep divergence of *Megaladapis* from all other Lemuriformes in the Eocene, most node ages for our phylogeny were also broadly concordant with those derived by [12]. We also included additional extinct lemurs, using previously published topologies for the Megaladapidae [69] and the Palaeopropithecidae [70, 71]. We conservatively estimated divergence dates not included in the Kistler database [1] by dividing known branch lengths into equal segments given by the number of branching events required.

**Table 6:** Comparison of node ages generated by Kistler et al. [1] and 10kTrees, showing node ages used in our expanded phylogeny

Node (Last common ancestor of …)	Kistler et al. [1]	10kTrees	Used
Chiromyiformes and Lemuriformes (including extinct taxa)	50 Ma (42–57)	51.1	50
Lemuriformes (including extinct taxa)	31 Ma (27–35)	33.3	31
^a^Archaeolemurid-indriid-^a^palaeopropithecid-lepilemurid-cheirogaleid clade	29 Ma (25–33)	–	29
Lepilemurid-cheirogaleid clade (including *Phaner*)^b^	25 Ma (21–30)	28.5	28.5
^a^Megaladapid-lemurid clade	27 Ma (23–32)	–	27
*Phaner*-lepilemurid clade	–	25.7	25.7
^a^Archaeolemurid-indriid-^a^palaeopropithecid clade	24 Ma (20–28)	–	24
Cheirogaleids (except *Phaner*)	–	22.6	22.6
Indriid-^a^palaeopropithecid clade	21 Ma (17–24)	–	21
Lemuridae (including extinct taxa)	19 Ma (16–22)	20.6	19
Indriidae	17 Ma (14–20)	17.0	17
*Lemur*-*Hapalemur*-*Eulemur* clade^b^	14 Ma (11–17)	15.0	15.0
*Lepilemur*	12 Ma (9–15)	13.3	13.3
^a^*Pachylemur*-*Varecia* clade	11 Ma (8–14)	–	11
^a^*Palaeopropithecus ingens*-^a^*P. maximus* clade	0.6 Ma (< 1 k)	–	0.6

^a^Extinct taxon

^b^Kistler et al. [1] do not include *Phaner* or *Hapalemur* in their analysis

Next, we ran the full suite of modeling experiments on the phylogeny used by Herrera (Supplementary Material pages 62–63, File S1 in ref. [2]) to determine the degree to which the phylogeny affects modeling results. To do so without biasing the results due to different numbers of taxa, we pruned the Herrera tree to include only the taxa we had represented in the expanded Kistler et al. tree. The resulting analysis is presented in Additional file 4.

### Assessing changes in diversification rates over time

We used four software packages to model changes in lemur diversification rates (speciation - extinction) over time: BAMM v. 2.5 [72], and R Core Team [73] packages RPANDA [74], TreePar [75], and TESS [76]. BAMM and TESS employ reversible jump Markov Chain Monte Car*l*o estimation procedures that explore multiple model spaces at once, while RPANDA and TreePar employ maximum likelihood procedures and consider one model at a time. The code we used for all modeling experiments, with rationale, is described in Additional file 3.

We first applied TreePar [75]. This program uses a likelihood function called the birth-death-shift process to estimate all rate parameters throughout a phylogeny, and returns diversification rates and the probability of survival at all points in time across the tree. It allows investigators to force the model to include a major extinction event without specifying where it might fall. However, this analysis assumes that before and after the extinction event, speciation and extinction rates remain identical and constant. If at some point in the phylogeny the speciation rate temporarily shifted downward, TreePar would model a decrease in lineage survival rather than change in speciation rate, because it does not allow for changes in speciation rate. The program thus forces any paucity of species to result from decreases in lineage survival. TreePar does not allow practitioners to decompose estimated diversification rates into their constituent extinction and speciation rates.

In estimating diversification rate, RPANDA allows speciation and extinction rates to vary over time or to be constant, in any combination [74]. Using RPANDA, we fit four diversification models (constant speciation-constant extinction, constant speciation-time varying extinction, time varying speciation-constant extinction, time varying speciation- time varying extinction). For each run, we estimated diversification rates over time, and compared size-corrected Akaike information criterion (AICc) values to determine the best-fitting model. A model’s AICc score reflects how well it reflects the observed data; the lower the AICc, the closer the fit [77]. To account for incomplete sampling of known tips, we estimated the fraction of known species in the clade that are included in the phylogeny. Our phylogeny is based on 89 species, including 17 Holocene subfossil and 62 extant species of lemurs. The total lemur species count in the Holocene has been estimated at ~ 120. We thus provided the fraction 89/120, or 0.74, to account for incomplete taxon sampling, and we used the same fraction in other modeling experiments, when needed (Additional file 3).

BAMM is even more flexible [72]; it employs reversible jump Markov Chain Monte Carlo (rjMCMC) estimation procedures that explore multiple model subspaces at once. This allows users to consider multiple fundamentally different models simultaneously that may include rate shifts and extinctions as they estimate diversification dynamics across a phylogeny. Each distinct diversification model corresponds to a model subspace and the estimation procedure selects the most likely diversification model by beginning with a single diversification rate regime for the whole tree and subsequently adding regime shifts and comparing model fits. Using BAMM, we implemented a speciation-extinction model with random incomplete taxon sampling and one extinction.

We used the CoMET model in the TESS package [76] to estimate diversification rates. The CoMET model is similar BAMM as it uses rjMCMC estimation, but it allows for inclusion of Bayesian priors on the magnitude of rate-shift events such as extinctions. We set priors to define a single mass extinction event during which 5% species survival (95% CI: 0.01–0.10) was expected, and we specified a single rate change. We also set Normal priors on the speciation and extinction rates (λ and μ, respectively) that were informed by the results of the BAMM analysis (Additional file 3). Last, we reran the CoMET model with weaker magnitudes of extinction, once with mean 25% survival (95% CI: 0.07–0.50) and once with mean 75% survival (95% CI: 0.48–0.94). Diversification rate estimates remained qualitatively unchanged by choice of prior for extinction magnitude.

### Testing for large-scale extinction events

We first tested for large-scale extinctions using RPANDA, positing environmental predictors of extinction, and comparing candidate models to a null model that posits no extinction pulse. Because global temperature changed drastically during the Cenozoic (Fig. 2a), dropping precipitously at 34 Ma (Fig. 2b), we fitted two more models: first, that global temperature exponentially influenced extinction rate [24, 74], and next, that the rate of change of global temperature exponentially influenced extinction rate.

To test if an environmental perturbation triggered a major extinction at the E-O boundary (34 Ma), we created a model that simulated a disturbance occurring only then. We used the probability distribution function of a Normal distribution with a mean equal to 34 and variance equal to 0.25 over the domain 50 to 0 Ma. This variable is an artificially simulated peak and not a ‘traditional’ environmental variable. We simulated 49 more additional environmental pulse variables to determine whether a pulse at the E-O boundary explains lemur extinction better than a pulse at any other million-year interval. Each was generated as described above, but with its own unique peak disturbance time. We then fitted a sequence of models each with a single pulse exponentially influencing extinction rate. Last we fitted a null model with a flat line exponentially influencing extinction rate. We obtained AICc scores and ΔAICc values (AICc – min AICc). Elaborating from Bolker [78], differences in ΔAICc < 4 were taken to mean that the models being compared are indistinguishable, 4 < ΔAICc < 10 were taken to mean that the model with the lower raw score is moderately better than the other, and 10 < ΔAICc were taken to suggest strong support for the model with the lower AICc score [75]. We obtained an Akaike weight profile for models positing major extinction events from 50 to 0 Ma. Akaike weights are conditional probabilities that can be used to assess the relative strengths of competing models in accounting for the raw data [77, 79].

We also considered the environmental pulse variables as exponential predictor variables of speciation rate in order to see if a punctuated reduction in diversification rate may be due to a drop in speciation rate as opposed to a spike in extinction rate. To do so, we repeated the process above and considered all simulated environmental pulse variables, as well as the environmental temperature and its rate of change, as exponential predictors of the speciation rate. Last, we ensured that all models in RPANDA with environmental variables (i.e., absolute temperature and rate of change in temperature) as exponential predictors of extinction rates were qualitatively similar when considered as linear predictors of extinction rates (Additional file 3).

We next employed the CoMET model in the TESS package to test for major extinctions. CoMET measures evidence of a major extinction in Bayes Factors, which compare the ratios of marginal likelihoods models with a single major extinction event happening at a given time to the marginal likelihood of a model with no rise in extinction rates happening at that time. The output (a Bayes Factor support profile for single large-scale extinctions at given times) can be interpreted like the Akaike weight profile output from RPANDA. Following Kass and Raftery [80], we interpreted Bayes Factors above 2 as “moderate” support for a major extinction occurring at a given point in time, and above 6 as “strong” support. We checked to see if evidence for a major extinction at any time was sensitive to our choice of prior on extinction magnitude. All Bayesian models in TESS and BAMM were run until satisfactory convergence with details in Additional file 3, and we interpreted parameters as converged using Gelman-Rubin statistics and a cut-off value of 1.1 [81]. All modeling was performed with R version 3.4.0, except for the TESS analysis which was performed in R version 3.3.1 on the Massachusetts Green High Performance Computing Cluster. T-tests of differences between extinct and extant clades were performed using SPSS Version 26.

## Supplementary information

**Additional file 1:** Supplementary Data (Paleocene and Eocene). **Table S1.** Terrestrial vertebrate families present on continental Africa during the Paleocene, Eocene, or both.

**Additional file 2:** Supplementary Data (Holocene and Cretaceous). **Table S2.** Known Madagascar terrestrial vertebrate clades and when they arrived.

**Additional file 3.** Supplementary R Code and Output (Based on Kistler et al., 2015). R Code for modeling lemur diversification using expanded phylogeny from Kistler and colleagues.

**Additional file 4. **Supplementary R Code and Output (Based on Herrera, 2017). R Code for modeling lemur diversification using phylogeny from Herrera.

## Data Availability

All data analyzed during this study are included in this published article and its supplementary information files (Additional file 1 Table S1 and Additional file 2 Table S2).

## Abbreviations

E-O: Eocene-Oligocene
EOT: Eocene-Oligocene Transition
K-Pg: Cretaceous-Paleogene
Ma: Million years ago
