# Supplementary material for: Mid-Cenozoic climate change, extinction, and faunal turnover in Madagascar, and their bearing on the evolution of lemurs
Source: BMC Evol Biol. 2020 Aug 8;20:97. doi: 10.1186/s12862-020-01628-1 (PMC7414565; doi:10.1186/s12862-020-01628-1)
Supplement: Supplementary file 1 — Additional file 1: Supplementary Data (Paleocene and Eocene). Table S1. Terrestrial vertebrate families present on continental Africa during the Paleocene, Eocene, or both. [file 12862_2020_1628_MOESM1_ESM.docx]

*BMC Evolutionary Biology*

**Mid-Tertiary climate change, extinction, and faunal turnover in Madagascar, and their bearing on the evolution of lemurs**

Laurie R. Godfrey, Karen E. Samonds, Justin W. Baldwin, Michael R. Sutherland, Jason M. Kamilar, Kristen L. Allfisher

**Additional file 1: Supplementary Data (Paleocene and Eocene)**

*Continental Africa’s Paleocene and Eocene terrestrial vertebrate faunas*

The African Paleogene fossil record has improved greatly during the last few years with recent discoveries of Lissamphibians (Gardner & Rage, 2016), snakes (McCartney & Seiffert, 2016), mammals (Werdelin & Sanders, 2010; Gunnell *et al.*, 2014; Pickford, 2015c, b, a; Borths *et al.*, 2016; Ravel *et al.*, 2016; Simmons *et al.*, 2016), and birds (Mourer-Chauviré *et al.*, 2015). With the exception of the snake *Nigerophis* known from Niger (Rage, 1975) all Paleocene African fossils are known from Morocco (e.g., Hua & Jouve, 2004; Augé & Rage, 2006; Gaffney *et al.*, 2006; Gheerbrant *et al.*, 2014). The Paleocene record is dominated by reptiles, including geckos, snakes, turtles, skinks, and Amphisbaeniformes (Augé & Rage, 2006; Gaffney *et al.*, 2006). Four mammalian genera (hyaenodonts and condylarths) are also represented in the Paleocene (Gheerbrant *et al.*, 2001; Solé *et al.*, 2009; Lewis & Morlo, 2010). Two afrosoricids and one odontopterygiform bird are also described from Morocco/Algeria during both the Paleocene and Eocene (Gheerbrant, 1995; Bourdon *et al.*, 2010; De Bast *et al.*, 2012).

Many more taxa are reported from the Eocene, with the richest fossil record from Egypt (e.g., Seiffert & Simons, 2000; Seiffert *et al.*, 2005; Sallam *et al.*, 2010; Simmons *et al.*, 2016). Other localities include Tunisia, Algeria, Namibia, Morocco, and a few sites in Tanzania, Nigeria, Libya, Oman, and Togo (e.g., Delmer *et al.*, 2006; Rage *et al.*, 2013; Ravel *et al.*, 2016).

**Table S1. Terrestrial vertebrate families present on continental Africa during the Paleocene, Eocene, or both**

| **Class** | **Order** | **Family** | **Dispersal Advant.?** | **Family or close clade known to have colonized Madagascar?** | **Still extant (family-level taxon lives today, independent of its range)** | **Source** |
| --- | --- | --- | --- | --- | --- | --- |
| Amphibia | Anura | Pipidae | no | no | yes | Báez & Harrison, 2005 |
| Amphibia | Anura | “Ranoidea”^[[1]](#footnote-1)^ | no | yes | yes | Rage *et al.*, 2013; Gardner & Rage, 2016 |
| Aves | Cariamiformes | Phorusrhacidae? | no | no | no | Mourer-Chauviré *et al.*, 2011 |
| Aves | Galliformes | Paraortygidae | yes | no | no | Mourer-Chauviré *et al.*, 2015 |
| Aves | Odontopterygiformes | Pelagornithidae | yes | no | no | Bourdon *et al.*, 2010 |
| Aves | Phaethontiformes | Phaethontidae | yes | yes | yes | Bourdon *et al.*, 2008 |
| Aves | Phaethontiformes | Prophaethontidae | yes | no | no | Bourdon *et al.*, 2005 |
| Mammalia | Afrosoricida | Adapisoriculidae | no | no | no | De Bast *et al.*, 2012 |
| Mammalia | Afrosoricida | Chambilestidae | no | no | no | Gheerbrant & Hartenberger, 1999 |
| Mammalia | Afrosoricida | Chrysochloridae | no | no | yes | Pickford, 2015 |
| Mammalia | Afrosoricida | incertae sedis 1 (=*Dilambdogale*) | no | yes? | no | Seiffert, 2010 |
| Mammalia | Afrosoricida | incertae sedis 2 (=*Widanelfarasia*) | no | yes? | no | Seiffert & Simons, 2000 |
| Mammalia | Artiodactyla | Anthracotheriidae | no | yes? | no | Holroyd *et al.*, 1996 |
| Mammalia | Chiroptera | Aegyptonycteridae | yes | no | no | Simmons *et al.*, 2016 |
| Mammalia | Chiroptera | Emballonuridae | yes | yes | yes | Gunnell, 2010 |
| Mamamlia | Chiroptera | Hipposideridae | yes | yes | yes | Ravel *et al.*, 2016 |
| Mammalia | Chiroptera | Megadermatidae | yes | no | yes | Gunnell, 2010 |
| Mammalia | Chiroptera | Myzopodidae | yes | yes | yes | Gunnell *et al.*, 2014 |
| Mammalia | Chiroptera | Necromantidae | yes | no | no | Ravel *et al.*, 2016 |
| Mammalia | Chiroptera | Nycteridae | yes | yes | yes | Ravel *et al.*, 2016 |
| Mammalia | Chiroptera | Philisidae | yes | no | no | Gunnell *et al.*, 2008; Ravel *et al.*, 2012 |
| Mammalia | Chiroptera | Rhinopomatidae | yes | no | yes | Gunnell *et al.*, 2008 |
| Mammalia | Chiroptera | Tanzanycterididae | yes | no | no | Gunnell *et al.*, 2003 |
| Mammalia | Chiroptera | Vespertilionidae | yes | yes | yes | Gunnell, 2010 |
| Mammalia | Cimolesta | Todralestidae | no | no | no | Gheerbrant, 1991 |
| Mammalia | Condylarthra | Hyopsodontidae | no | no | no | Tabuce *et al.*, 2005 |
| Mammalia | Condylarthra | Mioclaenidae | no | no | no | Gheerbrant *et al.*, 2001 |
| Mammalia | Didelphimorphia | Peradectidae | no | no | no | Hartenberger *et al.*, 2001 |
| Mammalia | Hyaenodonta | Apternodontinae | no | no | no | Lewis & Morlo, 2010 |
| Mammalia | Hyaenodonta | Hyainailourinae | no | no | no | Borths *et al.*, 2016 |
| Mammalia | Hyaenodonta | Teratodontidae | no | no | no | Borths *et al.*, 2016; Borths *et al.*, 2017 |
| Mammalia | Hyracoidea | Geniohyidae | no | no | no | Court & Mahboubi, 1993 |
| Mammalia | Hyracoidea | Pliohyracidae | no | no | no | Barrow *et al.*, 2010 |
| Mammalia | Hyracoidea | Saghatheriidae | no | no | no | Rasmussen & Gutiérrez, 2010 |
| Mammalia | Hyracoidea | Titanohyracidae | no | no | no | Rasmussen & Gutiérrez, 2010 |
| Mammalia | Macroscelidea | Apheliscidae | no | no | no | Hooker & Russell, 2012 |
| Mammalia | Macroscelidea | Macroscelididae | no | no | yes | Simons *et al.*, 1991 |
| Mammalia | ?Paenungulata | Ocepeiidae | no | no | no | Gheerbrant *et al.*, 2014 |
| Mammalia | Primates | Adapidae | no | no | no | Seiffert *et al.*, 2009 |
| Mammalia | Primates | Afrotarsiidae | no | no | no | Jaeger *et al.*, 2010 |
| Mammalia | Primates | Azibiidae | no | no | no | Tabuce *et al.*, 2004 |
| Mammalia | Primates | Djebelemuridae | no | no | no | Simons, 1997 |
| Mammalia | Primates | Lorisoidea | no | yes | yes | Seiffert *et al.*, 2003; Pickford, 2015a |
| Mammalia | Primates | Oligopithecidae | no | no | no | Simons, 1989 |
| Mammalia | Primates | Parapithecidae | no | no | no | Seiffert *et al.*, 2005 |
| Mammalia | Primates | Plesiopithecidae | no | yes? | no | Godinot, 2010 |
| Mammalia | Primates | Proteopithecidae | no | no | no | Simons, 1989 |
| Mammalia | Proboscidea | Moeritheriidae | no | no | no | Delmer *et al.*, 2006 |
| Mammalia | Proboscidea | Numidotheriidae | no | no | no | Court, 1995 |
| Mammalia | Proboscidea | Phosphatheriidae | no | no | no | Gheerbrant *et al.*, 1996 |
| Mammalia | Ptolemaiida | Ptolemaiidae | no | no | no | Simons & Bown, 1995 |
| Mammalia | Rodentia | Anomaluridae | no | no | yes | Sallam *et al.*, 2010; Heritage *et al.*, 2016 |
| Mammalia | Rodentia | Gaudeamuridae | no | no | yes | Sallam *et al*., 2011 |
| Mammalia | Rodentia | Nementchamyidae | no | no | no | Jaeger *et al.*, 1985 |
| Mammalia | Rodentia | Phiomyidae | no | no | no | Marivaux *et al.*, 2014 |
| Mammalia | Rodentia | Zegdoumyidae | no | no | no | Adaci et al., 2007 |
| Sauropsida | Crocodylia | Dyrosauridae | yes | no | no | Jouve *et al.*, 2008 |
| Sauropsida | Crocodylia | Gavialidae | yes | no | yes | Jonet & Wouters, 1977 |
| Sauropsida | Crocodylia | Rhabdosauridae | yes | no | no | Bergounioux, 1955 |
| Sauropsida | Crocodylia | Sebecosuchia  (=*Eremosuchus*) | yes | no | no | Buffetaut, 1989 |
| Sauropsida | Crocodylia | Tomistomidae | yes | no | no | Brochu & Gingerich, 2000; Jouve *et al.*, 2015 |
| Sauropsida | Squamata | Amphisbaeniformes | no | no | yes | Augé & Rage, 2006 |
| Sauropsida | Squamata | Boidae | no | yes | yes | McCartney & Seiffert, 2016 |
| Sauropsida | Squamata | Colubridae | no | yes | yes | Rage *et al.*, 2013; McCartney & Seiffert, 2016 |
| Sauropsida | Squamata | Coniophidae | no | no | no | Augé & Rage, 2006 |
| Sauropsida | Squamata | Gekkonidae | yes | yes | yes | Augé & Rage, 2006 |
| Sauropsida | Squamata | Madtsoiidae | no | no | no | Rage, 1987; McCartney & Seiffert, 2016 |
| Sauropsida | Squamata | Nigerophiidae | no | no | no | Rage, 1975 |
| Sauropsida | Squamata | Palaeophiidae | no | no | no | Hoffstetter, 1961; McCartney & Seiffert, 2016 |
| Sauropsida | Squamata | Russellophiidae | no | no | no | McCartney & Seiffert, 2016 |
| Sauropsida | Squamata | Scincomorpha | no | yes | yes | Augé & Rage, 2006 |
| Sauropsida | Squamata | Tropidophiidae | no | no | yes | Augé & Rage, 2006; McCartney & Seiffert, 2016 |
| Sauropsida | Squamata | Varanidae | no? | no | yes | Holmes *et al.*, 2010 |
| Sauropsida | Testudines | Bothremydidae | yes | no | no | Gaffney *et al.*, 2006 |
| Sauropsida | Testudines | Podocnemididae | yes | yes | yes | Adaci *et al.*, 2007 |

**References**

Adaci, M., Tabuce, R., Mebrouk, F., Bensalah, M., Fabre, P.-H., Hautier, L., Jaeger, J.-J., Lazzari, V., Mahboubi, M.h., Marivaux, L., Otero, O., Peigné, S. & Tong, H. (2007) Nouveaux sites à vertébrés paléogènes dans la région des Gour Lazib (Sahara nord-occidental, Algérie). *Comptes Rendus Palevol*, **6**, 535-544.

Augé, M. & Rage, J.-C. (2006) Herpétofaunes du Paléocène supérieur et de l'Eocène inférieur du Maroc. *Annales de paleontologie*, **92**, 235-253.

Báez , A.M. & Harrison, T. (2005) A new Pipine frog from an Eocene crater lake in north-central Tanzania. *Palaeontology*, **48**, 723-737.

Barrow, E., Seiffert, E.R. & Simons, E.L. (2010) A primitive hyracoid (Mammalia, Paenungulata) from the early Priabonian (Late Eocene) of Egypt. *Journal of Systematic Palaeontology*, **8**, 213-244.

Bergounioux, F.-M. (1955) Les crocodiliens fossiles des depots phosphates du Sud-Tunisien. *Comptes rendus hebdomadaires de seances de l'Academie des sciences* **240**, 1917-1918.

Borths, M.R. & Seiffert, E.R. (2017) Craniodental and humeral morphology of a new species of *Masrasector* (Teratodontinae, Hyaenodonta, Placentalia) from the late Eocene of Egypt and locomotor diversity in hyaenodonts. PLoS ONE, 12(4): e0173527. https://doi.org/10.1371/journal.pone.0173527.

Borths, M.R., Holroyd, P.A. & Seiffert, E.R. (2016) Hyainailourine and teratodontine cranial material from the late Eocene of Egypt and the application of parsimony and Bayesian methods to the phylogeny and biogeography of Hyaenodonta (Placentalia, Mammalia). *PeerJ*, **4**, e2639.

Bourdon, E., Bouya, B. & Iarochene, M. (2005) Earliest African neornithine bird: A new species of prophaethontidae (Aves) from the Paleocene of Morocco. *Journal of Vertebrate Paleontology*, **25**, 157-170.

Bourdon, E., Amaghzaz, M. & Bouya, B. (2008) A new seabird (Aves, cf. Phaethontidae) from the Lower Eocene phosphates of Morocco. *Geobios*, **41**, 455-459.

Bourdon, E., Amaghzaz, M. & Bouya, B. (2010) Pseudotoothed birds (Aves, Odontopterygiformes) from the early Tertiary of Morocco. *American Museum Novitates*, **3704**, 1-71.

Brochu, C.A. & Gingerich, P.D. (2000) New tomistomine crocodylian from the middle Eocene (Bartonian) of Wadi Hitan, Fayum Province, Egypt. *Contributions from the Museum of Paleontology, The University of Michigan*, **30**, 251-268.

Buffetaut, E. (1989) A new ziphodont mesosuchian crocodile from the Eocene of Algeria. *Palaeontographica Abteilung A*, **208**, 1-10.

Court, N. (1995) A New Species of Numidotherium (Mammalia: Proboscidea) from the Eocene of Libya and the Early Phylogeny of the Proboscidea. *Journal of Vertebrate Paleontology*, **15**, 650-671.

Court, N. & Mahboubi, M. (1993) Reassessment of Lower Eocene *Seggeurius amourensis*: Aspects of primitive dental morphology in the mammalian Order Hyracoidea. *Journal of Paleontology*, **67**, 889-893.

De Bast, E., Sigé, B. & Thierry Smith, T. (2012) Diversity of the adapisoriculid mammals from the early Palaeocene of Hainin, Belgium. *Acta Palaeontologica Polonica*, **57**, 35-52.

Delmer, C., Mahboubi, M., Tabuce, R. & Tassy, P. (2006) A new species of *Moeritherium* (Proboscidea, Mammalia) from the Eocene of Algeria: New perspectives on the ancestral morphotypes of the genus. *Palaeontology*, **49**, 421-434.

Gaffney, E.S., Tong, H. & Meylan, P.A. (2006) Evolution of the side-necked turtles: The families Bothremydidae, Euraxemydidae, and Araripemydidae. *Bulletin of the American Museum of Natural History*, **300**, 1-318.

Gardner, J.D. & Rage, J.-C. (2016) The fossil record of lissamphibians from Africa, Madagascar, and the Arabian Plate. *Palaeobiodiversity and Palaeoenvironments*, **96**, 169-220.

Gheerbrant, E. (1991) *Todralestes variablis* n. g., n. sp., nouveau Proteuthérien (Eutheria, Todralestidae fam. nov.) du Paléocène du Maroc. *Comptes Rendus de l'Académie des Sciences Paris, Série II* **312**:1249-1255.

Gheerbrant, E. (1995) Les mammifères Paléocènes du Bassin d'Quarzazate (Maroc). III. Adapisoriculidae et autres mammifères (Carnivora, ? Creodonta, Condylarthra, ? Ungulata et incertae sedis). *Palaeontographica Abteilung A*, **237**, 39-132.

Gheerbrant, E. & Hartenberger, J.-L. (1999) Nouveau mammifère insectivore (?Lipotyphla, ?Erinaceomorpha) de l’Eocène inférieur de Chambi (Tunisie). *Paläontologische Zeitschrift*, **73**, 143.

Gheerbrant, E., Sudre, J. & Cappetta, H. (1996) A Palaeocene proboscidean from Morocco. *Nature*, **383**, 68.

Gheerbrant, E., Sudre, J., Iarochene, M. & Moumni, A. (2001) First ascertained African “Condylarth” mammals (primitive ungulates: cf. Bulbulodentata and cf. Phenacodonta) from the earliest Ypresian of the Ouled Abdoun Basin, Morocco. *Journal of Vertebrate Paleontology*, **21**, 107-118.

Gheerbrant, E., Amaghzaz, M., Bouya, B., Goussard, F. & Letenneur, C. (2014) *Ocepeia* (Middle Paleocene of Morocco): The Oldest Skull of an Afrotherian Mammal. *PLOS ONE*, **9**, e89739.

Godinot, M. (2010) Paleogene Prosimians. *Cenozoic Mammals of Africa* (ed. by L. Werdelin and W.J. Sanders), pp. 319-331. University of California Press, Berkeley, California.

Gunnell, G.F. (2010) Chiroptera. *Cenozoic Mamamls of Africa* (ed. by L. Werdelin and W.J. Sanders), pp. 581-597. University of California Press, Berkeley, California.

Gunnell, G.F., Simons, E.L. & Seiffert, E.R. (2008) New bats (Mammalia: Chiroptera) from the Late Eocene and Early Oligocene, Fayum Depression, Egypt. *Journal of Vertebrate Paleontology*, **28**, 1-11.

Gunnell, G.F., Simmons, N.B. & Seiffert, E.R. (2014) New Myzopodidae (Chiroptera) from the Late Paleogene of Egypt: Emended family diagnosis and biogeographic origins of Noctilionoidea. *PLOS ONE*, **9**, e86712.

Gunnell, G.F., Jacobs, B.F., Herendeen, P.S., Head, J.J., Kowalski, E., Msuya, C.P., Mizambwa, F.A., Harrison, T., Habersetzer, J. & Storch, G. (2003) Oldest placental mammal from sub-Saharan Africa: Eocene microbat from Tanzania - evidence for early evolution of sophisticated echolocation. *Palaeontologia Electronica*, **5**, 1-10.

Hartenberger, J.-L., Crochet, J.-Y., Martinez, C., Marandat, B. & Sigé, B. (2001) The Eocene mammalian fauna of Chambi (Tunisia) in its geological context. *Eocene Biodiversity: Unusual Occurrences and Rarely Sampled Habitats* (ed. by G.F. Gunnell), pp. 237-250. Springer US, Boston, MA.

Heritage, S., Fernández, D., Sallam, H.M., Cronin, D.T., Esara Echube, J.M. & Seiffert, E.R. (2016) Ancient phylogenetic divergence of the enigmatic African rodent *Zenkerella* and the origin of anomalurid gliding. *PeerJ*, **4**, e2320.

Hoffstetter, R. (1961) Nouveaux restes d'un serpent boïdé (*Madtsoia madagascariensis* nov. sp.) dans le Crétacé supérieur de Madagascar. *Bulletin du Muséum national d'Histoire naturelle, Paris*, **33**, 152-160.

Holmes, R.B., Murray, A.M., Attia, Y.S., Simons, E.L. & Chatrath, P. (2010) Oldest known *Varanus* (Squamata: Varanidae) from the Upper Eocene and Lower Oligocene of Egypt: support for an African origin of the genus. *Palaeontology*, **53**, 1099-1110.

Holroyd, P.A., Simons, E.L., Bown, T.M., Polly, P.D. & Kraus, M.J. (1996) New records of terrestrial mammals from the upper Eocene Qaser el Sagha Formation, Fayum Depression, Egypt. *Paleovertebrata*, **23**, 175-192.

Hooker, J.J. & Russell, D.E. (2012) Early Palaeogene Louisinidae (Macroscelidea, Mammalia), their relationships and north European diversity. *Zoological Journal of the Linnean Society*, **164**, 856-936.

Hua, S. & Jouve, S. (2004) A primitive marine gavialoid from the Paleocene of Morocco. *Journal of Vertebrate Paleontology*, **24**, 341-350.

Jaeger, J.-J., Denys, C. & Coiffait, B. (1985) New Phiomorpha and Anomaluridae from the Late Eocene of North-West Africa: Phylogenetic Implications. *Evolutionary Relationships among Rodents: A Multidisciplinary Analysis* (ed. by W.P. Luckett and J.-L. Hartenberger), pp. 567-588. Springer US, Boston, MA.

Jaeger, J.-J., Beard, K.C., Chaimanee, Y., Salem, M., Benammi, M., Hlal, O., Coster, P., Bilal, A.A., Duringer, P., Schuster, M., Valentin, X., Marandat, B., Marivaux, L., Métais, E., Hammuda, O. & Brunet, M. (2010) Late middle Eocene epoch of Libya yields earliest known radiation of African anthropoids. *Nature*, **467**, 1095.

Jonet, S. & Wouters, G. (1977) *Maroccosuchus zennaroi*, crocodilien eusuchien nouveau des phosphates du Maroc. *Notes de la Service Geologique du Maroc*, **38**, 177-202.

Jouve, S., Bouya, B. & Amaghzaz, M. (2008) A long-snouted dyrosaurid (Crocodyliformes, Mesoeucrocodylia) from the Paleocene of Morocco: Phylogenetic and paleobiological implications. *Palaeontology*, **51**, 281-294.

Jouve, S., Bouya, B., Amaghzaz, M. & Meslouh, S. (2015) *Maroccosuchus zennaroi* (Crocodylia: Tomistominae) from the Eocene of Morocco: phylogenetic and palaeobiogeographical implications of the basalmost tomistomine. *Journal of Systematic Palaeontology*, **13**, 421-445.

Lewis, M.E. & Morlo, M. (2010) Creodonta. *Cenozoic Mammals of Africa* (ed. by L. Werdelin and W.J. Sanders), pp. 543-560. University of California Press, Berkeley, California.

Marivaux, L., Essid, E.M., Marzougui, W., Khayati Ammar, H., Adnet, S., Marandat, B., Merzeraud, G., Ramdarshan, A., Tabuce, R., Vianey-Liaud, M. & Yans, J. (2014) A morphological intermediate between eosimiiform and simiiform primates from the late middle Eocene of Tunisia: Macroevolutionary and paleobiogeographic implications of early anthropoids. *American Journal of Physical Anthropology*, **154**, 387-401.

McCartney, J.A. & Seiffert, E.R. (2016) A Late Eocene snake fauna from the Fayum Depression, Egypt. *Journal of Vertebrate Paleontology*, **36**, e1029580.

Mourer-Chauviré, C., Pickford, M. & Senut, B. (2015) Stem group galliform and stem group psittaciform birds (Aves, Galliformes, Paraortygidae, and Psittaciformes, family incertae sedis) from the Middle Eocene of Namibia. *Journal of Ornithology*, **156**, 275-286.

Mourer-Chauviré, C., Tabuce, R., Mahboubi, M.h., Adaci, M. & Bensalah, M. (2011) A Phororhacoid bird from the Eocene of Africa. *Naturwissenschaften*, **98**, 815.

Pickford, M. (2015) Chrysochloridae (Mammalia) from the Lutetian (Middle Eocene) of Black Crow, Namibia. *Communications of the Geological Survey of Namibia*, **16**, 105-113.

Rage, J.-C. (1975) Un serpent du Paléocène du Niger. Etude préliminaire sur l’origine des Caenophidians (Sauropsida, Serpentes). *Comptes Rendus à l’ Académie des Sciences, Paris, Sciences de la Terre et des Planètes*, **281**, 515-518.

Rage, J.-C., Pickford, M. & Senut, B. (2013) Amphibians and squamates from the middle Eocene of Namibia, with comments on pre-Miocene anurans from Africa. *Annales de Paléontologie*, **99**, 217-242.

Rage, J.C. (1987) Fossil history. *Snakes: Ecology and Evolutionary Biology* (ed. by R.A. Seigel, J.T.C. Collins and S.S. Novak), pp. 57-76. MacMillan, New York.

Rasmussen, D.T. & Gutiérrez, M. (2010) Hyracoidea. *Cenozoic Mammals of Africa* (ed. by L. Werdelin and W.J. Sanders). University of California Press, Berkeley, California.

Ravel, A., Marivaux, L., Tabuce, R., Ben Haj Ali, M., Essid, E.M. & Vianey-Liaud, M. (2012) A new large philisid (Mammalia, Chiroptera, Vespertilionoidea) from the late Early Eocene of Chambi, Tunisia. *Palaeontology*, **55**, 1035-1041.

Ravel, A., Adaci, M., Bensalah, M., Charruault, A.-L., Essid, E.M., Ammar, H.K., Marzougui, W., Mahboubi, M., Mebrouk, F., Merzeraud, G., Vianey-Liaud, M., Tabuce, R. & Marivaux, L. (2016) Origine et radiation initiale des chauves-souris modernes : nouvelles découvertes dans l'Éocène d'Afrique du Nord. *Geodiversitas*, **38**, 355-434.

Sallam, H.M., Seiffert, E.R., Simons, E.L. & Brindley, C. (2010) A large-bodied anomaluroid rodent from the earliest late Eocene of Egypt: phylogenetic and biogeographic implications. *Journal of Vertebrate Paleontology*, **30**, 1579-1593.

Sallam HM, Seiffert ER, Simons EL (2011) Craniodental morphology and systematics of a new family of Hystricognathous rodents (Gaudeamuridae) from the Late Eocene and Early Oligocene of Egypt. PLOS ONE 6(2): e16525. <https://doi.org/10.1371/journal.pone.0016525>.

Seiffert, E.R. (2010) The oldest and youngest records of Afrosoricida (Placentalia, Afrotheria) from the Fayum Depression of northern Egypt. *Acta Palaeontologica Polonica*, **55**, 599-616.

Seiffert, E.R. & Simons, E.L. (2000) *Widanelfarasia*, a Diminutive Placental from the Late Eocene of Egypt. *Proceedings of the National Academy of Sciences of the United States of America*, **97**, 2646-2651.

Seiffert, E.R., Simons, E.L. & Attia, Y. (2003) Fossil evidence for an ancient divergence of lorises and galagos. *Nature*, **422**, 421-424.

Seiffert, E.R., Perry, J.M.G., Simons, E.L. & Boyer, D.M. (2009) Convergent evolution of anthropoid-like adaptations in Eocene adapiform primates. *Nature*, **461**, 1118.

Seiffert, E.R., Simons, E.L., Clyde, W.C., Rossie, J.B., Attia, Y., Bown, T.M., Chatrath, P. & Mathison, M.E. (2005) Basal anthropoids from Egypt and the antiquity of Africa's higher primate radiation. *Science*, **310**, 300-304.

Simmons, N.B., Seiffert, E.R. & Gunnell, G.F. (2016) A new family of large omnivorous bats (Mammalia, Chiroptera) from the Late Eocene of the Fayum Depression, Egypt, with comments on use of the name “Eochiroptera”. *American Museum Novitates*, **3857**, 1-43.

Simons, E.L. (1989) Description of two genera and species of late Eocene Anthropoidea from Egypt. *Proceedings of the National Academy of Sciences*, **86**, 9956-9960.

Simons, E.L. (1997) Discovery of the smallest Fayum Egyptian primates (Anchomomyini, Adapidae). *Proceedings of the National Academy of Sciences*, **94**, 180-184.

Simons, E.L. & Bown, T.M. (1995) Ptolemaiida, a new order of Mammalia--with description of the cranium of *Ptolemaia grangeri*. *Proceedings of the National Academy of Sciences*, **92**, 3269-3273.

Simons, E.L., Holroyd, P.A. & Bown, T.M. (1991) Early tertiary elephant-shrews from Egypt and the origin of the Macroscelidea. *Proceedings of the National Academy of Sciences*, **88**, 9734-9737.

Solé, F., Gheerbrant, E., Amaghzaz, M. & Bouya, B. (2009) Further evidence of the African antiquity of hyaenodontid (‘Creodonta’, Mammalia) evolution. *Zoological Journal of the Linnean Society*, **156**, 827-846.

Tabuce, R., Mahboubi, M., Tafforeau, P. & Sudre, J. (2004) Discovery of a highly-specialized plesiadapiform primate in the early-middle Eocene of northwestern Africa. *Journal of Human Evolution*, **47**, 305-321.

Tabuce, R., Adnet, S., Cappetta, H., Noubhani, A. & Quillevéré, F. (2005) Aznag (bassin d'Ouarzazate, Maroc), nouvelle localité à sélaciens et mammifères de l'Eocène moyen (Lutétien) d'Afrique. *Bulletin de la Société Géologique de France*, **176**, 381-400.

Werdelin, L. & Sanders, W.J. (eds.) (2010) *Cenozoic Mammals of Africa*. University of California Press, Berkeley, California.

1. Ranoidea was previously classified as a superfamily and is now a major clade of Neobatrachians [↑](#footnote-ref-1)
