# Supplementary material for: Mid-Cenozoic climate change, extinction, and faunal turnover in Madagascar, and their bearing on the evolution of lemurs
Source: BMC Evol Biol. 2020 Aug 8;20:97. doi: 10.1186/s12862-020-01628-1 (PMC7414565; doi:10.1186/s12862-020-01628-1)
Supplement: Supplementary file 2 — Additional file 2: Supplementary Data (Holocene and Cretaceous). Table S2. Known Madagascar terrestrial vertebrate clades and when they arrived. [file 12862_2020_1628_MOESM2_ESM.docx]

*BMC Evolutionary Biology*

**Mid-Cenozoic climate change, extinction, and faunal turnover in Madagascar, and their bearing on the evolution of lemurs**

Laurie R. Godfrey, Karen E. Samonds, Justin W. Baldwin, Michael R. Sutherland, Jason M. Kamilar, Kristen L. Allfisher

**Additional file 2: Supplementary Data (Holocene and Cretaceous)**

*Madagascar’s Cretaceous terrestrial vertebrate fauna*

The phylogenetic relationships of most of Madagascar’s Late Cretaceous fossil groups do not indicate that they are sister groups of the modern and/or Holocene fauna, and therefore are hypothesized to have gone extinct during the Cenozoic (e.g., a Late Cretaceous giant ceratophryine frog with South American affinities; Evans *et al.*, 2008) the bothremydid turtle *Kinkonychelys* (Gaffney *et al.*, 2009) and *Sokatra*, a more basal pelomedusoid (Gaffney & Krause, 2011). However, *Eremnochelys* from the Late Cretaceous of Madagascar (Gaffney & Forster, 2003) represents the only Cretaceous occurrence of a genus currently represented in the extant Malagasy vertebrate fauna (Krause, 2003). Other Late Cretaceous reptiles thought to have arrived before the end of the Eocene include cordylids lizard (Krause, 2003), which today are restricted to sub-Saharan Africa, at least four species of notosuchian and two species of neosuchian crocodyliforms (Buckley *et al.*, 2000; Rasmusson Simons & Buckley, 2009; Krause & Kley, 2010), three species of basal snake (LaDuke *et al.*, 2010), two species of sauropod dinosaurs (Rogers & Forster, 2001; Rogers & Wilson, 2014), and three species of non-avian theropod dinosaurs (Forster *et al.*, 1998; Sampson *et al.*, 2001; Sampson & Krause, 2007). The Mesozoic record of mammals also displays this same pattern; a Jurassic australosphenidan (Flynn *et al.*, 1999) and several Late Cretaceous non-placental taxa, are both distantly related to the extant clades of placental mammals living on Madagascar today, and not hypothesized to be their ancestors (Krause *et al.*, 2006; Krause *et al.*, 2014). Based on molecular evidence, extant reptiles thought to have arrived before the K-T boundary with living representatives today include oplurid lizards and boid snakes (Noonan & Chippendale, 2006), and the endemic family of Malagasy blind snakes Xenotyphlopidae (Vidal *et al.*, 2010).

*Madagascar’s Holocene terrestrial vertebrate fauna*

The Madagascan terrestrial vertebrate fauna of the Holocene is very different from that of the Paleocene and Eocene of Africa. For groups that have well-understood biogeographic relationships, the fauna is dominated by roughly equal numbers of reptiles and mammals, with birds less well-represented, and amphibians comparatively poorly understood (see Samonds *et al.*, 2013). Most Malagasy amphibians and reptiles are hypothesized to have arrived before the end of the Eocene, while birds and mammals are largely thought to have arrived after the Eocene. Molecular work provides strong evidence that Madagascar’s carnivorans and nesomyine rodents arrived after the Eocene, probably all from Africa (Poux *et al.*, 2005). Many birds and bats also arrived after the end of the Eocene; these events are often reconstructed as dispersals from multiple geographic areas and, in some cases, multiple dispersals within families (Warren *et al.*, 2005; Trujillo *et al.*, 2009; Goodman, 2011; Reddy *et al.*, 2012).

**Table S2. Known Madagascar terrestrial vertebrate clades and when they arrived**

| **Class** | **Clade / Taxon**  **(Ancestor of)** | **Malagasy Genera** | **Period** | **Arrival Time** | **Dispersal Ability** | **References** |
| --- | --- | --- | --- | --- | --- | --- |
| Amphibia | Ceratophryinae | *Beelzebufo* | Cret. | Pre E/O | Disadvantaged | Evans *et al.*, 2008 |
| Amphibia | Hyperoliidae | *Heterixalus* | Holocene | Post E/O | Disadvantaged | Vences *et al.*, 2003a |
| Amphibia | Microhylidae | Cophylinae + Scaphiophryninae | Holocene | Pre E/O | Disadvantaged | van der Meijden *et al.*, 2007 |
| Amphibia | Microhylidae | Dyscophinae | Holocene | Pre E/O | Disadvantaged | van der Meijden *et al.*, 2007 |
| Amphibia | Mantellidae | *Aglyptodactylus*, *Blommersia*, *Boehmantis*, *Boophis*, *Gephyromantis*, *Guibemantis*, *Laliostoma*, *Mantella*, *Mantidactylus*, *Spinomantis*, *Tsingymantis*, *Wakea* | Holocene | Pre E/O | Disadvantaged | Bossuyt & Milinkovitch, 2001; Vences & Glaw, 2001; Vences *et al.*, 2003b) (Bossuyt *et al.*, 2006; Van Bocxlaer *et al.*, 2006; van der Meijden *et al.*, 2007; Kurabayashi *et al.*, 2008 |
| Amphibia | Ptychadenidae | *Ptychadena* | Holocene | Post E/O | Advantaged | Vences *et al.*, 2004b; Measey *et al.*, 2007; Crottini *et al*., 2012; Zimkus *et al.*, 2017 |
| Sauropsida | Bothremydidae | Kinkonychelys | Cret. | Pre E/O | Advantaged | Gaffney *et al.*, 2009 |
| Sauropsida | Podocnemidae | Erymnochelys | Holocene & Cret. | Pre E/O | Advantaged | Gaffney & Forster, 2003; Noonan & Chippendale, 2006 |
| Sauropsida | Testudinae | *Astrochelys, Aldabrachelys* | Holocene | Post E/O | Advantaged | Palkovacs *et al.*, 2002 |
| Sauropsida | Crocodylidae | *Crocodylus* | Holocene | Post E/O | Advantaged | Brochu, 2007; Hekkala *et al.*, 2011; Crottini *et al*., 2012 |
| Sauropsida | Crocodylidae | *Voay* | Holocene | Post E/O | Advantaged | Brochu, 2007; Bickelmann & Klein, 2009 |
| Sauropsida | Mahajangasuchidae | *Mahajangasuchus* | Cret. | Pre E/O | Advantaged | Buckley & Brochu, 1999; Turner & Buckley, 2008 |
| Sauropsida | Notosuchia | *Araripesuchus* | Cret. | Pre E/O | Advantaged | Turner, 2006 |
| Sauropsida | Notosuchia | *Simosuchus* | Cret. | Pre E/O | Advantaged | Buckley *et al.*, 2000; Turner & Sertich, 2010 |
| Sauropsida | Trematochampsidae | *Miadanasuchus* | Cret. | Pre E/O | Advantaged | Rasmusson Simons & Buckley, 2009 |
| Sauropsida | Chamaeleonidae | *Brookesia*, *Calumma*, *Furcifer* | Holocene | Pre E/O | Disadvantaged | Raxworthy *et al.*, 2002 |
| Sauropsida | ?Cordylidae | *Konkasaurus* | Cret. | Pre E/O | Disadvantaged | Krause *et al.*, 2003 |
| Sauropsida | Dromaeosauridae | *Rahonavis* | Cret. | Pre E/O | Advantaged | Forster *et al.*, 1998; Makovicky *et al.*, 2005 |
| Sauropsida | Gerrhosauridae | *Zonosaurus, Tracheloptychus* | Holocene | Pre E/O | Disadvantaged | Raselimanana *et al.*, 2009 |
| Sauropsida | Opluridae | *Chalarodon, Oplurus* | Holocene | Pre E/O | Disadvantaged | Noonan & Chippendale, 2006 |
| Sauropsida | Gekkonidae^^[[1]](#footnote-1)^^ | *Blaesodactylus* | Holocene | Pre E/O | Advantaged | Vences *et al.*, 2004a |
| Sauropsida | Gekkonidae^1^ | *Hemidactylus* mercatorius | Holocene | Post E/O | Advantaged | Vences *et al.*, 2004a; Crottini *et al*., 2012 |
| Sauropsida | Gekkonidae^1^ | *Hemidactylus* playcephalus | Holocene | Post E/O | Advantaged | Vences *et al.*, 2004a; Crottini *et al*., 2012 |
| Sauropsida | Scincidae | *Trachylepis* | Holocene | Post E/O | Disadvantaged | Mausfeld *et al.*, 2000 |
| Sauropsida | Scincidae^^[[2]](#footnote-2)^^ | Cryptoblepharus | Holocene | Post E/O | Advantaged | Rocha *et al.*, 2006 |
| Sauropsida | Scincidae | Amphiglossus, Androngo, Madascincus, Paracontias, Pseudoacontias, Pygomeles, Sirenoscincus, Voeltzkowia | Holocene | Pre E/O | Disadvantaged | Brandley *et al.*, 2008; Crottini *et al.*, 2009 |
| Sauropsida | Boidae | *Acrantophis*, *Sanzinia*, *Calabaria* | Holocene | Pre E/O | Disadvantaged | Noonan & Chippendale, 2006 |
| Sauropsida | Lamprophiidae | *Alluaudina*,  *Compsophis*,  *Dromicodryas*,  *Geodipsas*,  *Heteroliodon*,  *Ithycyphus*, *Langaha*,  *Leioheterodon*,  *Liophidium*,  *Liopholidophis*,  *Madagascarophis*,  *Micropisthodon*,  *Pseudoxyrhopus*,  *Stenophis* | Holocene | Post E/O | Disadvantaged | Nagy *et al.*, 2003; Rage *et al.*, 2013 |
| Sauropsida | Lamprophiidae | *Mimophis* | Holocene | Post E/O | Disadvantaged | Nagy *et al.*, 2003 |
| Sauropsida | Madtsoiidae | *Madtsoia* | Cret. | Pre E/O | Disadvantaged | Hoffstetter, 1961 |
| Sauropsida | Madtsoiidae | *Menarana* | Cret. | Pre E/O | Disadvantaged | LaDuke *et al.*, 2010 |
| Sauropsida | Nigerophiidae | *Kelyophis* | Cret. | Pre E/O | Disadvantaged | LaDuke *et al.*, 2010 |
| Sauropsida | Typhlopidae | *Typhlops* | Holocene | Pre E/O | Disadvantaged | Vidal *et al.*, 2010 |
| Sauropsida | Xenotyphlopidae | *Xenotyphlops* | Holocene | Pre E/O | Disadvantaged | Vidal *et al.*, 2010 |
| Sauropsida | Abelisauridae | *Majungasaurus* | Cret. | Pre E/O | Disadvantaged | Krause *et al.*, 2007 |
| Sauropsida | Noasauridae | *Masiakasaurus* | Cret. | Pre E/O | Disadvantaged | Sampson *et al.*, 2001 |
| Sauropsida | Nemegtosauridae | *Rapetosaurus* | Cret. | Pre E/O | Disadvantaged | Rogers & Forster, 2001 |
| Aves | Aepyornithidae | *Aepyornis, Mullerornis* | Holocene | Pre E/O | Disadvantaged | Burney *et al.*, 1997; Cooper *et al.*, 2001; Bourdon *et al.*, 2009 |
| Aves | Acrocephalidae | *Nesillas* | Holocene | Post E/O | Advantaged | Fuchs *et al.*, 2016 |
| Aves | Apodidae | *Aerodramus* | Holocene | Post E/O | Advantaged | Johnson & Clayton, 1999 |
| Aves | Bernieridae | *Thamnornis, Xanthomixis* | Holocene | Post E/O | Advantaged | Beresford *et al.*, 2005 |
| Aves | Campephagidae | *Coracina* | Holocene | Post E/O | Advantaged | Fuchs *et al.*, 2007; Fuchs *et al.*, 2008 |
| Aves | Dicruridae | *Dicrurus* | Holocene | Post E/O | Advantaged | Pasquet *et al.*, 2007 |
| Aves | Eurylaimidae | *Neodrepanis, Philepitta* | Holocene | Pre E/O? | Advantaged | Beresford *et al.*, 2005 |
| Aves | Mesitornithidae | *Mesitornis, Monias* | Holocene | Pre E/O | Disadvantaged | Burney *et al.*, 1997; Fain & Houde, 2004 |
| Aves | Motacillidae | *Motacilla* | Holocene | Post E/O | Advantaged | Voelker, 2002 |
| Aves | Nectarinidae | *Nectarinia* *souimanga* clade | Holocene | Post E/O | Advantaged | Warren *et al.*, 2003 |
| Aves | Nectarinidae | *Nectarinia* *notata* clade | Holocene | Post E/O | Advantaged | Warren *et al.*, 2003 |
| Aves 1 | Ornithurae | *Vorona* | Cret. | Pre E/O | Advantaged | Forster *et al.*, 1996 |
| Aves | Phaethontidae | *Phaethon* | Holocene | Pre E/O | Advantaged | Kennedy & Spencer, 2004; Safford & Hawkins, 2013 |
| Aves | Psittacidae | *Agapornis* | Holocene | Pre E/O | Advantaged | Burney *et al.*, 1997; Schweizer *et al.*, 2010 |
| Aves | Psittacidae | *Coracopsis* | Holocene | Pre E/O | Advantaged | Burney *et al.*, 1997; Wright *et al.*, 2008; Schweizer *et al.*, 2010 |
| Aves | Pycnonotidae | *Hypsipetes* | Holocene | Post E/O | Advantaged | Burney *et al.*, 1997; Warren *et al.*, 2005 |
| Aves | Strigidae | *Otus* | Holocene | Post E/O | Advantaged | Burney *et al.*, 1997; Fuchs *et al.*, 2008 |
| Aves | Sturnidae | *Hartlaubius* | Holocene | Post E/O | Advantaged | Zuccon *et al.*, 2006 |
| Aves | Vangidae | *Artamella*, *Calicalicus*, *Cyanolanius*, *Euryceros*, *Falculea*, *Hypositta*, *Leptopterus*, *Mystacornis*, *Newtonia*, *Oriolia*, *Pseudobias*, *Schetba*, *Tylas*, *Xenopirostris*, *Vanga* | Holocene | Post E/O | Advantaged | Reddy *et al.*, 2012 |
| Aves | Zosteropidae | *Zosterops* *borbonicus* lineage | Holocene | Post E/O | Advantaged | Warren *et al.*, 2006 |
| Aves | Zosteropidae | *Zosterops* *maderaspatanus* lineage | Holocene | Post E/O | Advantaged | Warren *et al.*, 2006 |
| Mammalia | Emballonuridae | *Paremballonura atrata, P. tiavato* | Holocene | Post E/O | Advantaged | Teeling *et al.*, 2005; Goodman *et al.*, 2012 |
| Mammalia | Emballonuridae | *Coleura kibomalandy* | Holocene | Post E/O | Advantaged | Teeling *et al.*, 2005; Goodman, 2011; Goodman *et al.*, 2012 |
| Mammalia | Emballonuridae | *Taphozous mauritianus* | Holocene | Post E/O | Advantaged | Teeling *et al.*, 2005; Goodman *et al.*, 2012 |
| Mammalia | Eupleridae | *Cryptoprocta, Eupleres, Fossa, Galidia, Galidictis* | Holocene | Post E/O | Disadvantaged | Poux *et al.*, 2005; Muldoon *et al.*, 2009 |
| Mammalia | Hippopotamidae | *Hippopotamus lemerlei, H. laloumena* | Holocene | Post E/O | Advantaged | Stuenes, 1989; Fovet *et al.*, 2011 |
| Mammalia | Hippopotamidae | *Hexaprotodon guldbergi* | Holocene | Post E/O | Advantaged | Stuenes, 1989; Boisserie, 2005; Fovet *et al.*, 2011 |
| Mammalia | Hipposideridae | *Hipposideros* | Holocene | Post E/O | Advantaged | Teeling *et al.*, 2005; Samonds, 2007 |
| Mammalia | Hipposideridae | *Triaenops* (*T. auritus* + *T. furculus + T. goodmani*) | Holocene | Post E/O | Advantaged | Samonds, 2007; Russell *et al.*, 2008 |
| Mammalia | Hipposideridae | *Triaenops* (*T. menamena)* | Holocene | Post E/O | Advantaged | Russell *et al.*, 2008 |
| Mammalia | Lemuroidea | *Cheirogaleus, Daubentonia, Eulemur, Hapalemur, Lemur, Lepilemur, Microcebus, Mirza, Propithecus, Varecia* | Holocene | Pre E/O | Disadvantaged | Horvath *et al.*, 2008; Springer *et al.*, 2012 |
| Mammalia | Marsupialia | Genus indet. | Cret. | Pre E/O | Disadvantaged | Krause, 2001 |
| Mammalia | Molossidae | *Chaerephon leucogaster* | Holocene | Post E/O | Advantaged | Jones *et al.*, 2005; Goodman, 2011; Lamb *et al.*, 2011 |
| Mammalia | Molossidae | *Chaerephon atsinanana* | Holocene | Post E/O | Advantaged | Goodman, 2011; Lamb *et al.*, 2011 |
| Mammalia | Molossidae | *Chaerephon jobimena* | Holocene | Post E/O | Advantaged | Goodman, 2011; Lamb *et al.*, 2011 |
| Mammalia | Molossidae | *Mops leucostigma* | Holocene | Post E/O | Advantaged | Goodman, 2011; Lamb *et al.*, 2011 |
| Mammalia | Molossidae | *Mops midas* | Holocene | Post E/O | Advantaged | Goodman, 2011; Lamb *et al.*, 2011 |
| Mammalia | Molossidae | *Mormopterus jugularis* | Holocene | - | Advantaged | Muldoon *et al.*, 2009; Goodman, 2011; Lamb *et al.*, 2011 |
| Mammalia | Molossidae | *Otomops madagascariensis* | Holocene | - | Advantaged | Muldoon *et al.*, 2009; Goodman, 2011; Lamb *et al.*, 2011 |
| Mammalia | Molossidae | *Tadarida fulminans* | Holocene | Post E/O | Advantaged | Goodman, 2011 |
| Mammalia | Multituberculata | Genus indet. | Cret. | Pre E/O | Disadvantaged | Krause *et al.*, 2006 |
| Mammalia | Myzopodidae | *Myzopoda* | Holocene | Pre E/O | Advantaged | Teeling *et al.*, 2005 |
| Mammalia | Nesomyinae | *Brachytarsomys, Eliurus, Hypogeomys, Macrotarsomys* | Holocene | Post E/O | Disadvantaged | Poux *et al.*, 2005; Samonds *et al.*, 2010 |
| Mammalia | Nycteridae | *Nycteris* | Holocene | Pre E/O? | Advantaged | Teeling *et al.*, 2005 |
| Mammalia | Plesiorycteropodidae | *Plesiorycteropus* | Holocene | Pre E/O | Disadvantaged | MacPhee, 1994; Asher *et al.*, 2003; Horovitz, 2004 |
| Mammalia | Pteropodidae | *Pteropus rufus* | Holocene | Post E/O | Advantaged | Samonds, 2007; O'Brien *et al.*, 2009 |
| Mammalia | Pteropodidae | *Eidolon dupreanum* | Holocene | - | Advantaged | Teeling *et al.*, 2005; Samonds, 2007 |
| Mammalia | Pteropodidae | *Rousettus madagascariensis* | Holocene | Post E/O | Advantaged | Samonds, 2007; Goodman *et al.*, 2010 |
| Mammalia | Sudamericidae | *Lavanify, Vintana* | Cret. | Pre E/O | Disadvantaged | Krause *et al.*, 1997; Krause *et al.*, 2014 |
| Mammalia | Tenrecidae | *Limnogale, Microgale, Oryzorictes, Echinops, Setifer, Hemicentetes, Tenrec* | Holocene | Pre E/O? | Disadvantaged | Poux *et al.*, 2008; Muldoon *et al.*, 2009; Everson *et al.*, 2016 |
| Mammalia | Vespertilionidae | *Myotis* | Holocene | Post E/O | Advantaged | Stadelmann *et al.*, 2004; Teeling *et al.*, 2005; Samonds, 2007 |
| Mammalia | Vespertilionidae | *Scotophilus marovaza* | Holocene | Post E/O | Advantaged | Trujillo *et al.*, 2009 |
| Mammalia | Vespertilionidae | *Scotophilus robustus* | Holocene | Post E/O | Advantaged | Trujillo *et al.*, 2009 |
| Mammalia | Vespertilionidae | *Neoromica matroka* | Holocene | Post E/O | Advantaged | Goodman, 2011 |

Dispersal-disadvantaged (terrestrial), dispersal-advantaged (facultative swimmer or volant).

**References**

Asher, R., Novacek, M. & Geisler, J. (2003) Relationships of endemic African mammals and their fossil relatives based on morphological and molecular evidence. *Journal of Mammalian Evolution*, **10**, 131-194.

Beresford, P., Barker, F.K., Ryan, P.G. & Crowe, T.M. (2005) African endemics span the tree of songbirds (Passeri): molecular systematics of several evolutionary 'enigmas'. *Proceedings of the Royal Society B-Biological Sciences*, **272**, 849-858.

Bickelmann, C. & Klein, N. (2009) The late Pleistocene horned crocodile *Voay robustus* (Grandidier & Vaillant, 1872) from Madagascar in the Museum fur Naturkunde Berlin. *Fossil Record*, **12**, 13-21.

Boisserie, J.-R. (2005) The phylogeny and taxonomy of Hippopotamidae (Mammalia: Artiodactyla): a review based on morphology and cladistic analysis. *Zoological Journal of the Linnean Society*, **143**, 1-26.

Bossuyt, F. & Milinkovitch, M.C. (2001) Amphibians as indicators of early tertiary "out-of-India" dispersal of vertebrates. *Science*, **292**, 93-95.

Bossuyt, F., Brown, R.M., Hillis, D.M., Cannatella, D.C. & Milinkovitch, M.C. (2006) Phylogeny and biogeography of a cosmopolitan frog radiation: Late Cretaceous diversification resulted in continent-scale endemism in the family ranidae. *Systematic Biology*, **55**, 579-594.

Bourdon, E., de Ricqles, A. & Cubo, J. (2009) A new Transantarctic relationship: morphological evidence for a Rheidae–Dromaiidae–Casuariidae clade (Aves, Palaeognathae, Ratitae). *Zoological Journal of the Linnean Society*, **156**, 641-663.

Brandley, M., Huelsenbeck, J. & Wiens, J. (2008) Rates and patterns in the evolution of snake-like body form in squamate reptiles: Evidence for repeated re-evolution of lost digits and long-term persistence of intermediate body forms. *Evolution* **62**, 2042–2064.

Brochu, C.A. (2007) Morphology, relationships, and biogeographical significance of an extinct horned crocodile (Crocodylia, Crocodylidae) from the Quaternary of Madagascar. *Zoological Journal of the Linnean Society*, **150**, 835-863.

Buckley, G. & Brochu, C. (1999) An enigmatic new crocodile from the Upper Cretaceous of Madagascar. *Special Papers in Paleontology*, **60**, 149-175.

Buckley, G., Brochu, C., Krause, D. & Pol, D. (2000) A pug-nosed crocodyliform from the Late Cretaceous of Madagascar. *Nature*, **405**, 941-944.

Burney, D.A., James, H.F., Grady, F.V., Jean-Gervais Rafamantanantsoa, R., Wright, H.T. & Cowart, J.B. (1997) Environmental change, extinction and human activity: evidence from caves in NW Madagascar. *Journal of Biogeography*, **24**, 755-767.

Cooper, A., Lalueza-Fox, C., Anderson, S., Rambaut, A., Austin, J. & Ward, R. (2001) Complete mitochondrial genome sequences of two extinct moas clarify ratite evolution. *Nature*, **409**, 704-707.

Crottini, A., Dordel, J., Köhler, J., Glaw, F., Schmitz, A. & Vences, M. (2009) A multilocus phylogeny of Malagasy scincid lizards elucidates the relationships of the fossorial genera *Androngo* and *Cryptoscincus*. *Molecular Phylogenetics and Evolution*, **53**, 345–350.

Crottini, A., Madsen, O., Poux, C., Strauss, A., Vieites, D.R. & Vences, M. (2012) A vertebrate timetree elucidates the biogeographic pattern of a major biotic change around the K-T boundary in Madagascar. *Proceedings of the National Academy of Sciences, U.S.A.*, **109**, 5358–5363.

Evans, S.E., Jones, M.E.H. & Krause, D.W. (2008) A giant frog with South American affinities from the Late Cretaceous of Madagascar. *Proceedings of the National Academy of Sciences, U.S.A.*, **105**, 2951-2956.

Everson, K.M., Soarimalala, V., Goodman, S.M. & Olson, L.E. (2016) Multiple loci and complete taxonomic sampling resolve the phylogeny and biogeographic history of tenrecs (Mammalia: Tenrecidae) and reveal higher speciation rates in Madagascar's humid forests. *Systematic Biology*, **65**, 890-909.

Fain, M.G. & Houde, P. (2004) Parallel radiations in the primary clades of birds. *Evolution*, **58**, 2558-2573.

Flynn, J.J., Parrish, J.M., Rakotosamimanana, B., Simpson, W.F. & Wyss, A.R. (1999) A Middle Jurassic mammal from Madagascar. *Nature*, **401**, 57-60.

Forster, C.A., Chiappe, L.M., Krause, D.W. & Sampson, S.D. (1996) The first Cretaceous bird from Madagascar. *Nature*, **382**, 532-534.

Forster, C.A., Sampson, S.D., Chiappe, L.M. & Krause, D.W. (1998) The theropod ancestry of birds: new evidence from the Late Cretaceous of Madagascar. *Science*, **279**, 1915-1919.

Fovet, W., Faure, M. & Guérin, C. (2011) *Hippopotamus guldbergi* n. sp.: Révision du statut d’*Hippopotamus madagascariensis* Guldberg, 1883, après plus d’un siècle de malentendus et de confusions taxonomiques. *Zoosystema*, **33**, 61-82.

Fuchs, J., Cruaud, C., Couloux, A. & Pasquet, E. (2007) Complex biogeographic history of the cuckoo-shrikes and allies (Passeriformes: Campephagidae) revealed by mitochondrial and nuclear sequence data. *Molecular Phylogenetics and Evolution*, **44**, 138-153.

Fuchs, J., Lemoine, D., Parra, J.L., Pons, J.-M., Raherilalao, M.J., Prys-Jones, R., Thebaud, C., Warren, B.H. & Goodman, S.M. (2016) Long-distance dispersal and inter-island colonization across the western Malagasy Region explain diversification in brush-warblers (Passeriformes: *Nesillas*). *Biological Journal of the Linnean Society*, **119**, 873-889.

Fuchs, J., Pons, J.M., Goodman, S.M., Bretagnolle, V., Melo, M., Bowie, R.C.K., Currie, D., Safford, R., Virani, M.Z., Thomsett, S., Hija, A., Cruaud, C. & Pasquet, E. (2008) Tracing the colonization history of the Indian Ocean scops-owls (Strigiformes: *Otus*) with further insight into the spatio-temporal origin of the Malagasy avifauna. *Bmc Evolutionary Biology*, **8**, 197.

Gaffney, E. & Forster, C. (2003) Side-necked turtle lower jaws (Podocnemididae, Bothremydidae) from the Late Cretaceous Maevarano Formation of Madagascar. *American Museum Novitates*, 1-13.

Gaffney, E.S. & Krause, D.W. (2011) *Sokatra*, a new side-necked turtle (Late Cretaceous, Madagascar) and the diversification of the main groups of Pelomedusoides. *American Museum Novitates*, **3728**, 1-28.

Gaffney, E.S., Krause, D.W. & Zalmout, I.S. (2009) *Kinkonychelys*, a new side-necked turtle (Pelomedusoides: Bothremydidae) from the Late Cretaceous of Madagascar. *American Museum Novitates*, 1-25.

Goodman, S.M. (2011) *Les chauves-souris de Madagascar*. Association Vahatra, Antananarivo.

Goodman, S.M., Chan, L., Nowak, M. & Yoder, A. (2010) Phylogeny and biogeography of western Indian Ocean *Rousettus* (Chiroptera: Pteropodidae). *Journal of Mammalogy*, **91**, 593-606.

Goodman, S.M., Puechmaille, S.J., Friedli-Weyeneth, N., Gerlach, J., Ruedi, M., Schoeman, M.C., Stanley, W.T. & Teeling, E.C. (2012) Phylogeny of the Emballonurini (Emballonuridae) with descriptions of a new genus and species from Madagascar. *Journal of Mammalogy*, **93**, 1440-1455.

Hekkala, E., Shirley, M.H., Amato, G., Austin, J.D., Charter, S., Thorbjarnarson, J., Vliet, K.A., Houck, M.L., Desalle, R.O.B. & Blum, M.J. (2011) An ancient icon reveals new mysteries: mummy DNA resurrects a cryptic species within the Nile crocodile. *Molecular Ecology*, **20**, 4199-4215.

Hoffstetter, R. (1961) Nouveaux restes d'un serpent boïdé (*Madtsoia madagascariensis* nov. sp.) dans le Crétacé supérieur de Madagascar. *Bulletin du Muséum national d'Histoire naturelle, Paris*, **33**, 152-160.

Horovitz, I. (2004) Eutherian mammal systematics and the origins of South American ungulates as based on postcranial osteology. *Fanfare for an Uncommon Paleontologist: Papers in Honor of Malcolm C. McKenna* (ed. by M.R. Dawson and J.A. Lillegraven), pp. 63-79. Bulletin of the Carnegie Museum of Natural History.

Horvath, J.E., Weisrock, D.W., Embry, S.L., Fiorentino, I., Balhoff, J.P., Kappeler, P., Wray, G.A., Willard, H.F. & Yoder, A.D. (2008) Development and application of a phylogenomic toolkit: resolving the evolutionary history of Madagascar's lemurs. *Genome Research*, **18**, 489-499.

Johnson, K. & Clayton, D. (1999) Swiftlets on islands: genetics and phylogeny of the Seychelles and Mascarene swiftlets. *Phelsuma*, **7**, 9-13.

Jones, K.E., Bininda-Emonds, O.R.P. & Gittleman, J.L. (2005) Bats, clocks, and rocks: Diversification patterns in Chiroptera. *Evolution*, **59**, 2243-2255.

Kennedy, M. & Spencer, H.G. (2004) Phylogenies of the Frigatebirds (Fregatidae) and Tropicbirds (Phaethonidae), two divergent groups of the traditional order Pelecaniformes, inferred from mitochondrial DNA sequences. *Molecular Phylogenetics and Evolution*, **31**, 31-38.

Krause, D.W. (2001) Fossil molar from a Madagascan marsupial. *Nature*, **412**, 497-498.

Krause, D.W. (2003) Late Cretaceous vertebrates of Madagascar: a window into Gondwanan biogeography at the end of the age of the dinosaurs. *The Natural History of Madagascar* (ed. by S.M. Goodman and J.P. Benstead), pp. 40-47. The University of Chicago Press, Chicago.

Krause, D.W. & Kley, N.J. (2010) *Simosuchus clarki* (Crocodyliformes: Notosuchia) from the Late Cretaceous of Madagascar. *Journal of Vertebrate Paleontology*, **30**, 236 pp.

Krause, D.W., Hartman, J.H. & Wells, N.A. (1997) Late Cretaceous vertebrates from Madagascar: implications for biotic change in deep time. *Natural Change and Human Impact in Madagascar* (ed. by S.M. Goodman and B.D. Patterson), pp. 3-43. Smithsonian Institution Press, Washington D. C.

Krause, D.W., Evans, S.E. & Gao, K.-Q. (2003) First definitive record of Mesozoic lizards from Madagascar. *Journal of Vertebrate Paleontology*, **23**, 842-856.

Krause, D.W., Sampson, S.D., Carrano, M.T. & O'Connor, P.M. (2007) Overview of the history of discovery, taxonomy, phylogeny, and biogeography of *Majungasaurus crenatissimus* (Theropoda: Abelisauridae) from the Late Cretaceous of Madagascar. *Majungasaurus crenatissimus (Theropoda: Abelisauridae) from the Late Cretaceous of Madagascar* (ed. by S.D. Sampson and D.W. Krause), pp. 1-20. Society of Vertebrate Paleontology Memoir.

Krause, D.W., O'Connor, P.M., Rogers, K.C., Sampson, S.D., Buckley, G.A. & Rogers, R.R. (2006) Late Cretaceous terrestrial vertebrates from Madagascar: implications for Latin American biogeography. **93**, 178-208.

Krause, D.W., Hoffmann, S., Wible, J.R., Kirk, E.C., Schultz, J.A., von Koenigswald, W., Groenke, J.R., Rossie, J.B., O/'Connor, P.M., Seiffert, E.R., Dumont, E.R., Holloway, W.L., Rogers, R.R., Rahantarisoa, L.J., Kemp, A.D. & Andriamialison, H. (2014) First cranial remains of a gondwanatherian mammal reveal remarkable mosaicism. *Nature*, **515**, 512-517.

Kurabayashi, A., Sumida, M., Yonekawa, H., Glaw, F., Vences, M. & Hasegawa, M. (2008) Phylogeny, recombination, and mechanisms of stepwise mitochondrial genome reorganization in mantellid frogs from Madagascar. *Molecular Biology and Evolution*, **25**, 874–891.

LaDuke, T.C., Krause, D.W., Scanlon, J.D. & Kley, N.J. (2010) A Late Cretaceous (Maastrichtian) snake assemblage from the Maevarano Formation, Mahajanga Basin, Madagascar. *Journal of Vertebrate Paleontology*, **30**, 109–138.

Lamb, J.M., Ralph, T.M.C., Naidoo, T., Taylor, P.J., Ratrimomanarivo, F., Stanley, W.T. & Goodman, S.M. (2011) Toward a molecular phylogeny for the Molossidae (Chiroptera) of the Afro-Malagasy region. *Acta Chiropterologica*, **13**, 1-16.

MacPhee, R.D.E. (1994) Morphology, adaptations, and relationships of *Plesiorycteropus*, and a diagnosis of a new order of eutherian mammals. *Bulletin of the American Museum of Natural History*, **220**, 1-214.

Makovicky, P.J., Apesteguía, S. & Agnolín, F.L. (2005) The earliest dromaeosaurid theropod from South America. *Nature*, **437**, 1007.

Mausfeld, P., Vences, M., Schmitz, A. & Veith, M. (2000) First data on the molecular phylogeography of scincid lizards of the genus *Mabuya*. *Molecular Phylogenetics and Evolution*, **17**, 11-14.

Measey, G.J., Vences, M., Drewes, R.C., Chiari, Y., Melo, M. & Bourles, B. (2007) Freshwater paths across the ocean: molecular phylogeny of the frog *Ptychadena newtoni* gives insights into amphibian colonization of oceanic islands. *Journal of Biogeography*, **34**, 7-20.

Muldoon, K.M., DeBlieux, D.D., Simons, E.L. & Chatrath, P.S. (2009) The subfossil occurrence and paleoecological significance of small mammals at Ankilitelo Cave, southwestern Madagascar. *Journal of Mammalogy*, **90**, 26-55.

Nagy, Z., Joger, U., Wink, M., Glaw, F. & Vences, M. (2003) Multiple colonization of Madagascar and Socotra by colubrid snakes: evidence from nuclear and mitochondrial gene phylogenies. *Proceedings of the Royal Society of London*, **270**, 2613-2621.

Noonan, B.P. & Chippendale, P.T. (2006) Vicariant origin of Malagasy reptiles supports Late Cretaceous Antarctic landbridge. *American Naturalist*, **168**, 730-741.

O'Brien, J., Mariani, C., Olson, L., Russell, A.L., Say, L., Yoder, A.D. & Hayden, T.J. (2009) Multiple colonisations of the western Indian Ocean by *Pteropus* fruit bats (Megachiroptera: Pteropodidae): the furthest islands were colonised first. *Molecular Phylogenetics and Evolution*, **51**, 294-303.

Palkovacs, E.P., Gerlach, J. & Caccone, A. (2002) The evolutionary origin of Indian Ocean tortoises (*Dipsochelys*). *Molecular Phylogenetics and Evolution*, **24**, 216-227.

Pasquet, E., Pons, J.M., Fuchs, J., Cruaud, C. & Bretagnolle, V. (2007) Evolutionary history and biogeography of the drongos (Dicruridae), a tropical Old World clade of corvoid passerines. *Molecular Phylogenetics and Evolution*, **45**, 158-167.

Poux, C., Madsen, O., Glos, J., de Jong, W.W. & Vences, M. (2008) Molecular phylogeny and divergence times of Malagasy tenrecs: influence of data partitioning and taxon sampling on dating analyses. *BMC Evolutionary Biology*, **8**

Poux, C., Madsen, O., Marquard, E., Vieites, D.R., de Jong, W.W. & Vences, M. (2005) Asynchronous colonization of Madagascar by the four endemic clades of primates, tenrecs, carnivores, and rodents as inferred from nuclear genes. *Systematic Biology*, **54**, 719-730.

Rage, J.-C., Pickford, M. & Senut, B. (2013) Amphibians and squamates from the middle Eocene of Namibia, with comments on pre-Miocene anurans from Africa. *Annales de Paléontologie*, **99**, 217-242.

Raselimanana, A.P., Noonan, B., Karanth, K.P., Gauthier, J. & Yoder, A.D. (2009) Phylogeny and evolution of Malagasy plated lizards. *Molecular Phylogenetics and Evolution*, **50**, 336-344.

Rasmusson Simons, E.L. & Buckley, G.A. (2009) New material of "*Trematochampsa*" *oblita* (Crocodyliformes,Trematochampsidae) from the Late Cretaceous of Madagascar. *Journal of Vertebrate Paleontology*, **29**, 599-604.

Raxworthy, C., Forstner, M. & Nussbaum, R. (2002) Chameleon radiation by oceanic dispersal. *Nature*, **415**, 784-787.

Reddy, S., Driskell, A., Rabosky, D.L., Hackett, S.J. & Schulenberg, T.S. (2012) Diversification and the adaptive radiation of the vangas of Madagascar. *Proceedings of the Royal Society B*,

Rocha, S., Carretero, M.A., Vences, M., Glaw, F. & Harris, D.J. (2006) Deciphering patterns of transoceanic dispersal: the evolutionary origin and biogeography of coastal lizards (*Cryptoblepharus*) in the Western Indian Ocean region. *Journal of Biogeography*, **33**, 13-22.

Rogers, K.C. & Forster, C.A. (2001) The last of the dinosaur titans: a new sauropod from Madagascar. *Nature*, **412**, 530-534.

Rogers, K.C. & Wilson, J.A. (2014) *Vahiny depereti*, gen. et sp. nov., a new titanosaur (Dinosauria, Sauropoda) from the Upper Cretaceous Maevarano Formation, Madagascar. *Journal of Vertebrate Paleontology*, **34**, 606-617.

Russell, A.L., Goodman, S.M. & Cox, M.P. (2008) Coalescent analyses support multiple mainland-to-island dispersals in the evolution of Malagasy *Triaenops* bats (Chiroptera: Hipposideridae). *Journal of Biogeography*, **35**, 995-1003.

Safford, R. & Hawkins, F. (2013) *The Birds of Africa: The Malagasy Region*. Bloomsbury Publishing, New York.

Samonds, K., Parent, S., Muldoon, K., Crowley, B. & Godfrey, L. (2010) Rock matrix surrounding subfossil lemur skull yields diverse collection of mammalian subfossils: implications for reconstructing Madagascar’s paleoenvironments. *Malagasy Nature*, **4**, 1-16.

Samonds, K.E. (2007) Late Pleistocene bat fossils from Anjohibe Cave, northwestern Madagascar. *Acta Chiropterologica*, **9**, 39-65.

Samonds, K.E., Godfrey, L.R., Ali, J.R., Goodman, S.M., Vences, M., Sutherland, M.R., Irwin, M.T. & Krause, D.W. (2013) Imperfect isolation: Factors and filters shaping Madagascar’s extant vertebrate fauna. *PLOS ONE*, **8**, e62086.

Sampson, S., Carrano, M. & Forster, C. (2001) A bizarre predatory dinosaur from the Late Cretaceous of Madagascar. *Nature*, **409**, 504-506.

Sampson, S.D. & Krause, D.W. (2007) *Majungasaurus crenatissimus* (Theropoda: Abelisauridae) from the Late Cretaceous of Madagascar. *Journal of Vertebrate Paleontology, Memoir 8*, 184 pp.

Schweizer, M., Seehausen, O., Guntert, M. & Hertwig, S.T. (2010) The evolutionary diversification of parrots supports a taxon pulse model with multiple trans-oceanic dispersal events and local radiations. *Molecular Phylogenetics and Evolution*, **54**, 984-994.

Springer, M.S., Meredith, R.W., Gatesy, J., Emerling, C.A., Park, J., Rabosky, D.L., Stadler, T., Steiner, C., Ryder, O.A., Janečka, J.E., Fisher, C.A. & Murphy, W.J. (2012) Macroevolutionary dynamics and historical biogeography of primate diversification inferred from a species supermatrix. *PLOS ONE*, **7**, e49521.

Stadelmann, B., Jacobs, D., Schoeman, C. & Ruedi, M. (2004) Phylogeny of African *Myotis* bats (Chiroptera, Vespertilionidae) inferred from cytochrome b sequences. *Acta Chiropterologica*, **6**, 177–192.

Stuenes, S. (1989) Taxonomy, habits and relationships of the sub-fossil Madagascan hippopotamuses, *Hippopotamus lemerlei* and *H. madagascariensis*. *Journal of Vertebrate Paleontology*, **9**, 241-268.

Teeling, E.C., Springer, M.S., Madsen, O., Bates, P., O'Brien, S.J. & Murphy, W.J. (2005) A molecular phylogeny for bats illuminates biogeography and the fossil record. *Science*, **307**, 580-584.

Trujillo, R.G., Patton, J.C., Schlitter, D.A. & Bickham, J.W. (2009) Molecular phylogenetics of the bat genus *Scotophilus* (Chiroptera: Vespertilionidae): Perspectives from paternally and maternally inherited genomes. *Journal of Mammalogy*, **90**, 548-560.

Turner, A.H. (2006) Osteology and phylogeny of a new species of *Araripesuchus* (Crocodyliformes: Mesoeucrocodylia) from the Late Cretaceous of Madagascar. *Historical Biology*, **18**, 255-369.

Turner, A.H. & Buckley, G.A. (2008) *Mahajangasuchus insignis* (Crocodyliformes: Mesoeucrocodylia) cranial anatomy and new data on the origin of the eusuchian-style palate. *Journal of Vertebrate Paleontology*, **28**, 382-408.

Turner, A.H. & Sertich, J.J.W. (2010) Phylogenetic history of *Simosuchus clarki* (Crocodyliformes: Notosuchia) from the Late Cretaceous of Madagascar. *Journal of Vertebrate Paleontology*, **30**, 177-236.

Van Bocxlaer, I., Roelants, K., Biju, S.D., Nagaraju, J. & Bossuyt, F. (2006) Late Cretaceous vicariance in Gondwanan amphibians. *PLoS ONE*, **1**, 6.

van der Meijden, A., Vences, M., Hoegg, S., Boistel, R., Channing, A. & Meyer, A. (2007) Nuclear gene phylogeny of narrow-mouthed toads (Family: Microhylidae) and a discussion of competing hypotheses concerning their biogeographical origins. *Molecular Phylogenetics and Evolution*, **44**, 1017-1030.

Vences, M., Kosuch, J., Glaw, F., Bohme, W. & Veith, M. (2003a) Molecular phylogeny of hyperoliid treefrogs: biogeographic origin of Malagasy and Seychellean taxa and re-analysis of familial paraphyly. *Journal of Zoological Systematics and Evolutionary Research*, **41**, 205-215.

Vences, M., Wanke, S., Vieites, D.R., Branch, W.R., Glaw, F. & Meyer, A. (2004a) Natural colonization or introduction? Phylogeographical relationships and morphological differentiation of house geckos (*Hemidactylus*) from Madagascar. *Biological Journal of the Linnean Society*, **83**, 115-130.

Vences, M., Vieites, D.R., Glaw, F., Brinkmann, H., Kosuch, J., Veith, M. & Meyer, A. (2003b) Multiple overseas dispersal in amphibians. *Proceedings of the Royal Society of London Series B-Biological Sciences*, **270**, 2435-2442.

Vences, M., Kosuch, J., Rodel, M.O., Lotters, S., Channing, A., Glaw, F. & Bohme, W. (2004b) Phylogeography of *Ptychadena mascareniensis* suggests transoceanic dispersal in a widespread African-Malagasy frog lineage. *Journal of Biogeography*, **31**, 593-601.

Vences, M.J. & Glaw, F. (2001) When molecules claim for taxonomic changes: new proposals on the classification of Old World treefrogs. *Spixiana*, **24**, 85-92.

Vidal, N., Marin, J., Morini, M., Donnellan, S., Branch, W.R., Thomas, R., Vences, M., Wynn, A., Cruaud, C. & Hedges, S.B. (2010) Blindsnake evolutionary tree reveals long history on Gondwana. *Biology Letters*, **6**, 558-561.

Voelker, G. (2002) Systematics and historical biogeography of wagtails: dispersal versus vicariance revisited. *Condor*, **104**, 725-739.

Warren, B.H., Bermingham, E., Prys-Jones, R.P. & Thebaud, C. (2005) Tracking island colonization history and phenotypic shifts in Indian Ocean bulbuls (*Hypsipetes*: Pycnonotidae). *Biological Journal of the Linnean Society*, **85**, 271-287.

Warren, B.H., Bermingham, E., Prys-Jones, R.P. & Thebaud, C. (2006) Immigration, species radiation and extinction in a highly diverse songbird lineage: white-eyes on Indian Ocean islands. *Molecular Ecology*, **15**, 3769-3786.

Warren, B.H., Bermingham, E., Bowie, R.C.K., Prys-Jones, R.P. & Thebaud, C. (2003) Molecular phylogeography reveals island colonization history and diversification of western Indian Ocean sunbirds (*Nectarinia*: Nectariniidae). *Molecular Phylogenetics and Evolution*, **29**, 67-85.

Wright, T.F., Schirtzinger, E.E., Matsumoto, T., Eberhard, J.R., Graves, G.R., Sanchez, J.J., Capelli, S., Mueller, H., Scharpegge, J., Chambers, G.K. & Fleischer, R.C. (2008) A multilocus molecular phylogeny of the parrots (Psittaciformes): support for a Gondwanan origin during the Cretaceous. *Molecular Biology and Evolution*, **25**, 2141-2156.

Zimkus, B.M., Lawson, L.P., Barej, M.F., Barratt, C.D., Channing, A., Dash, K.M., Dehling, J.M., Du Preez, L., Gehring, P.-S., Greenbaum, E., Gvoždík, V., Harvey, J., Kielgast, J., Kusamba, C., Nagy, Z.T., Pabijan, M., Penner, J., Rödel, M.-O., Vences, M. & Lötters, S. (2017) Leapfrogging into new territory: How Mascarene ridged frogs diversified across Africa and Madagascar to maintain their ecological niche. *Molecular Phylogenetics and Evolution*, **106**, 254-269.

Zuccon, D., Cibois, A., Pasquet, E. & Ericson, P.G.P. (2006) Nuclear and mitochondrial sequence data reveal the major lineages of starlings, mynas and related taxa. *Molecular Phylogenetics and Evolution*, **41**, 333-344.

1. Scored as dispersal-advantaged due to saltwater-tolerant hard-shelled eggs, and demonstrated dispersal ability. [↑](#footnote-ref-1)
2. Scored as dispersal-advantaged as they are “semi-marine” and have demonstrated dispersal ability. [↑](#footnote-ref-2)
