# Supplementary material for: Mid-Cenozoic climate change, extinction, and faunal turnover in Madagascar, and their bearing on the evolution of lemurs
Source: BMC Evol Biol. 2020 Aug 8;20:97. doi: 10.1186/s12862-020-01628-1 (PMC7414565; doi:10.1186/s12862-020-01628-1)
Supplement: Supplementary file 3 — Additional file 3. Supplementary R Code and Output (Based on Kistler et al., 2015). R Code for modeling lemur diversification using expanded phylogeny from Kistler and colleagues. [file 12862_2020_1628_MOESM3_ESM.pdf]

BMC Evolutionary Biology

Mid-Cenozoic climate change, extinction, and faunal turnover in Madagascar, and their bearing on the evolution of lemurs

Laurie R. Godfrey, Karen E. Samonds, Justin W. Baldwin,
Michael R. Sutherland, Jason M. Kamilar, Kristen L. Allfisher

Additional file 3: Supplementary R Code and Output (Based on Kistler et al., 2015)

R Code for modeling lemur diversification using expanded phylogeny from Kistler and colleagues

This document shows all necessary code to replicate our findings.

```
library(BAMMtools)
library(coda)
library(mgcv)
library(phangorn)
library(phytools)
library(RPANDA)
library(scales)
library(TESS)
library(TreePar)
library(xtable)
```

1. Generate Phylogeny and Retrieve Temperature Data

Methods for phylogeny creation are explained in text.

```
lemurs <- read.tree("godfrey_lemur_phylog2.tre")
plot.phylo(lemurs,cex = 0.6)
axisPhylo()
```

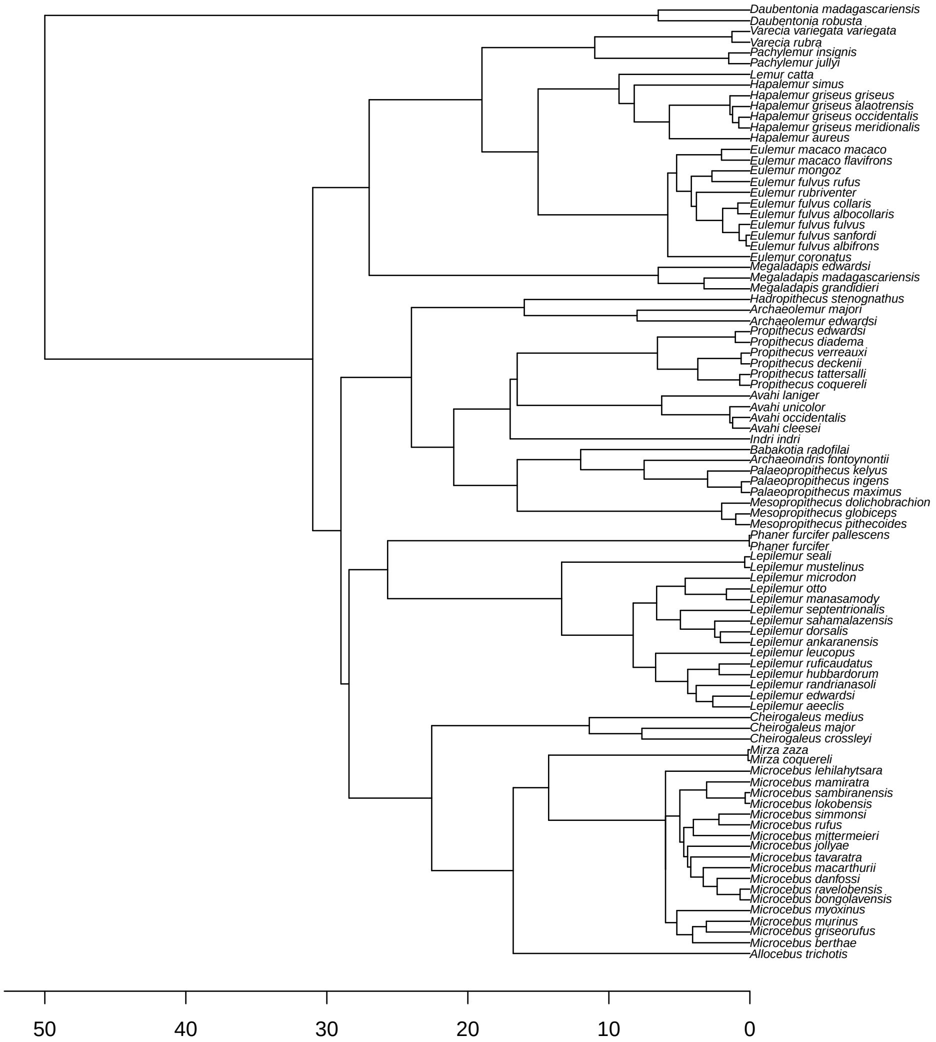

Now we will use the global temperature database from RPANDA (Zachos et al. 2008; Condamine et al. 2013).

```
data(InfTemp)
plot(InfTemp$Temperature~InfTemp$Age)
```

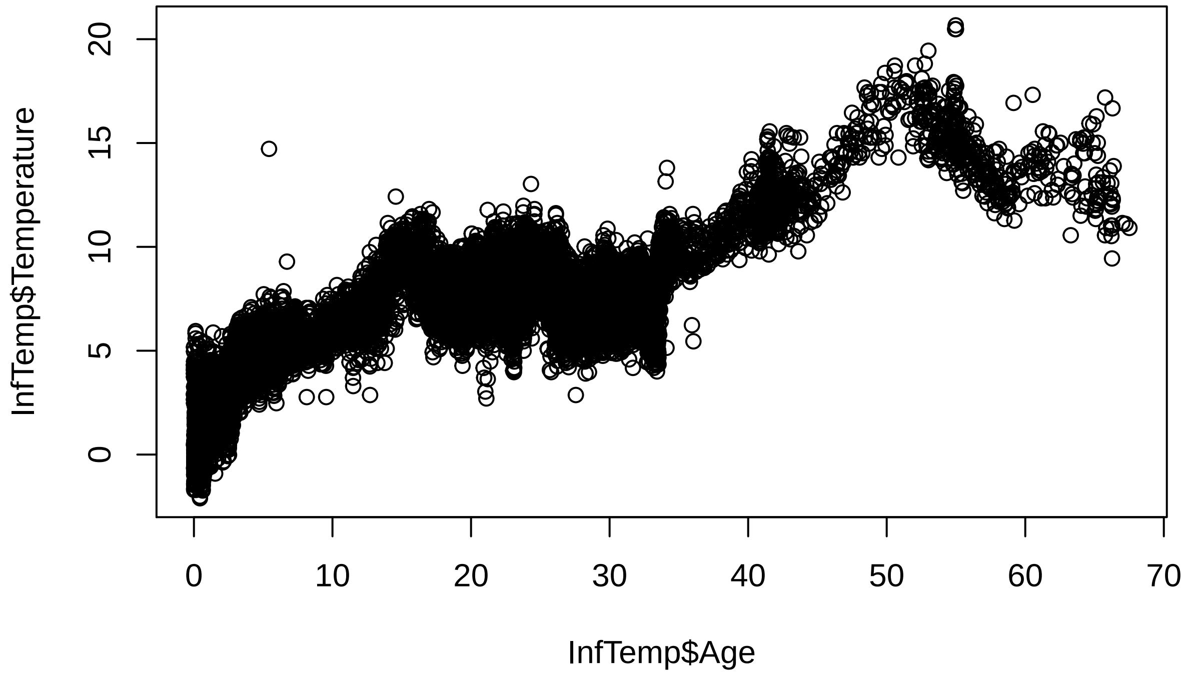

We use a generalized additive model (GAM) with 300 knots to predict the average temperature between 50 and 0 Ma. Black are the raw temperatures, red is the predicted temperature from the GAM.

```
InfTemp2=subset(InfTemp, InfTemp$Age<50)
set.seed(1)
G1 <- gam(Temperature ~ s(Age, k = 300, bs = "cs"), data = InfTemp2)
newdata=data.frame("Age"=seq(0, 70 ,length.out = 700))
newdata$predY <- predict(G1, type = "response", newdata = newdata)
InfTemp2$Nage=-InfTemp2$Age
newdata$Nage=-newdata$Age
plot(InfTemp2$Temperature~InfTemp2$Nage, xlab="Time",
     ylab=expression("Temperature ("~ degree~"C)"),
     axes=F, col=alpha("black", 0.35),ylim=c(-5,20))
axis(side=1)
axis(side=2)
lines(newdata$Nage,newdata$predY,col="red")
```

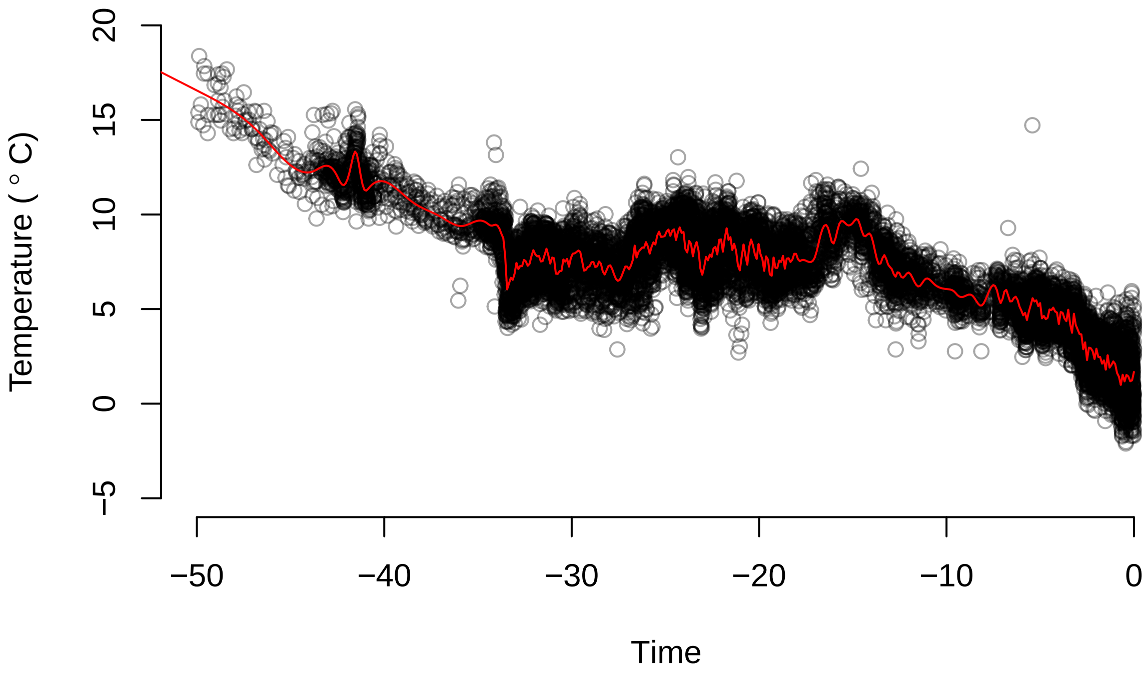

We also consider the rate of change of predicted temperature.

```
newdata$predY_diff <- c(0,diff(newdata$predY)/diff(newdata$Nage))
plot(predY_diff~Nage, data=newdata,type="l",xlim=c(-50,0), axes=F,ylim=c(-20,10),
     ylab = expression(Delta*degree ~ C ~ "/ Million Years"),
     xlab = "Time")
axis(side=1)
axis(side=2)
```

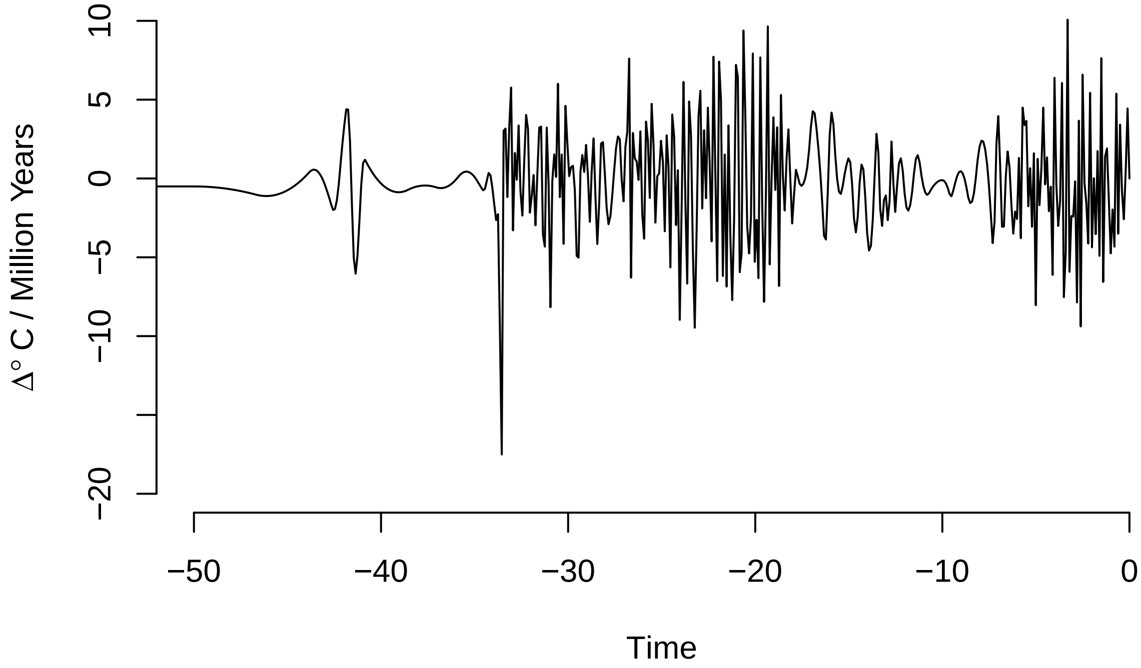

2. Estimate Diversification Rates

2.1. TreePar

We first used TreePar to estimate diversification rates over time.

```
res.shifts <- bd.shifts.optim(x,sampling=c(1,1),ME=TRUE, grid=1, start=0,end = 50)
```

```
x<-sort(getx(lemurs),decreasing=TRUE)
treepar.df <- data.frame()
for(i in 1:50){treepar.df <- rbind(treepar.df,
  res.shifts[[1]][[2]][[i]][[1]]$par
)}
names(treepar.df) <- c("turnover", "diversification", "probsurvive")
treepar.df$time_mya <- -1:-50
plot(diversification~time_mya, data=treepar.df, type="l", main="",
     xlab="Time", ylim=c(-0.1,0.1),
     ylab="Diversification Rate",axes=F,lwd=3)
axis(side=1)
axis(side=2)
abline(h=0, col="black", lty=2)
```

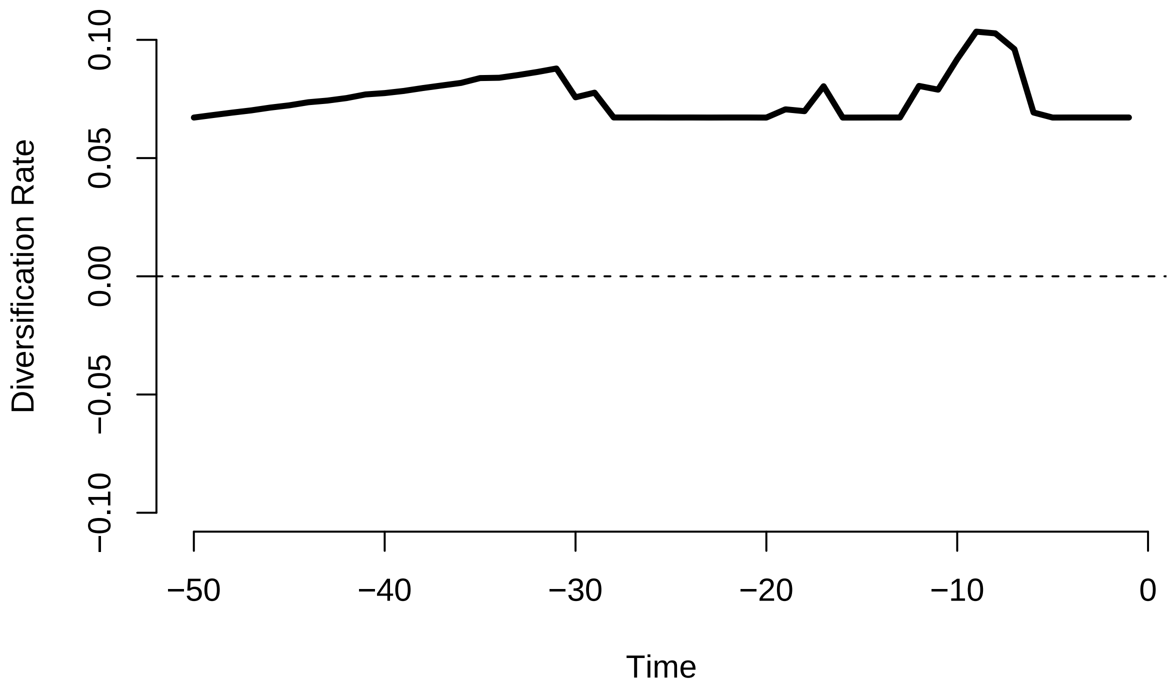

Below we report diversification rates at the beginning and end of the series.

```
fintab=data.frame(rbind(cbind(head(-treepar.df$time_mya,1),
                              head(treepar.df$diversification,1)),
             cbind(tail(-treepar.df$time_mya,1),
                   tail(treepar.df$diversification,1))))
names(fintab)=c("time", "div.rate")
fintab
```

```
##   time   div.rate
## 1    1 0.06716204
## 2   50 0.06714099
```

We also estimate survival rates over time.

```
plot(probsurvive~time_mya, data=treepar.df, type="l", main="",
     xlab="Time", ylim=c(0,1),
     ylab="P(Survival)",axes=F, lwd=3)
axis(side=1)
axis(side=2)
```

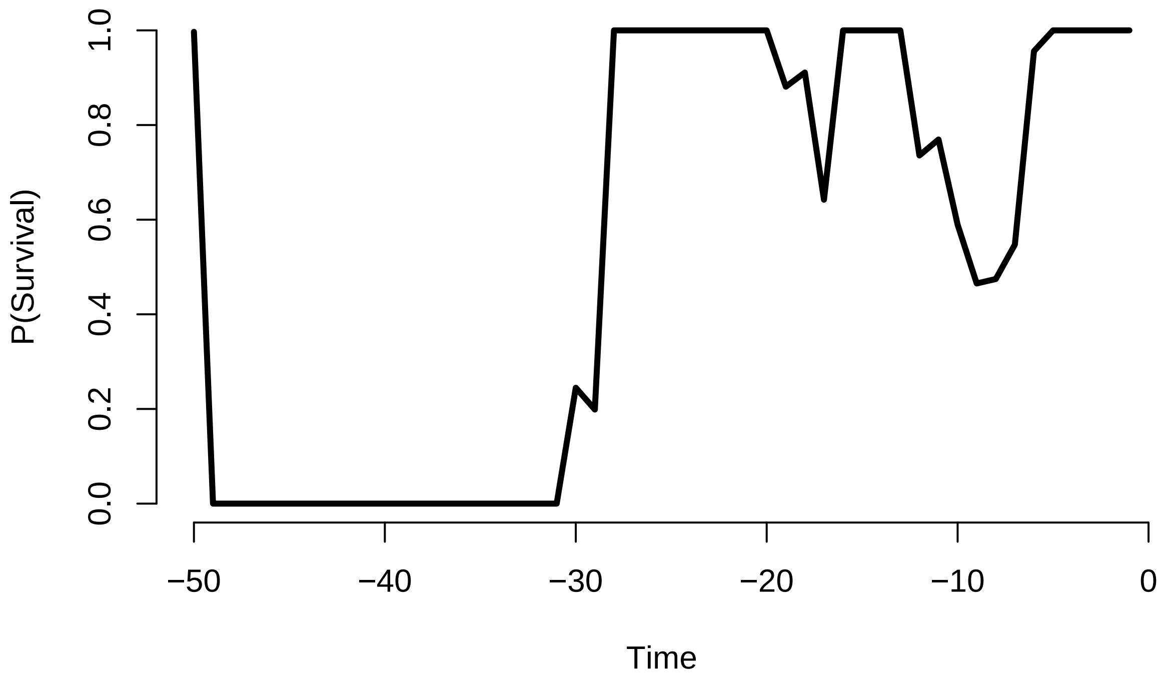

2.2. RPANDA

Morlon et al.'s package fits time-dependent diversification models that ignore enviromental variables. There are 4 choices: time-invariant speciation and extinction rates, time-invariance of either speciation or extinction rates and variable speciation and extinction rates. In all cases, we must provide the fraction of species of the clade that are included in the phylogeny to account for incomplete taxon sampling. The fraction we use is `0.7417`, stemming from the assumption that there likely 120 species of lemurs in the Quaternary and our phylogeny accounts for 89 species.

```
f=0.7417
lambda.cst <- function(x,y){y}
lambda.var <- function(x,y){y[1]*exp(y[2]*x)}
mu.cst <- function(x,y){y}
mu.var <- function(x,y){y[1]*exp(y[2]*x)}
fit.multi.rpanda <- function(tree,par)
    {
        bcstdcst <- fit_bd(tree, max(branching.times(tree)), f.lamb=lambda.cst, f.mu=mu.cst,
                           lamb_par=par[[1]][1],mu_par=par[[1]][2],
                           cst.lamb=TRUE,cst.mu=TRUE,cond="crown",f=f,dt=1e-3)
        bvardcst <- fit_bd(tree, max(branching.times(tree)), f.lamb=lambda.var, f.mu=mu.cst,
                           lamb_par=par[[2]][c(1,2)],mu_par=par[[2]][3],expo.lamb=TRUE,
                           cst.mu=TRUE,cond="crown",f=f,dt=1e-3)
        bcstdvar <- fit_bd(tree, max(branching.times(tree)), f.lamb=lambda.cst, f.mu=mu.var,
                           lamb_par=par[[3]][1],mu_par=par[[3]][c(2,3)],
                           cst.lamb=TRUE,expo.mu=TRUE,cond="crown",f=f,dt=1e-3)
```

```r
        bvardvar <- fit_bd(tree, max(branching.times(tree)), f.lamb=lambda.var, f.mu=mu.var,
                           lamb_par=par[[4]][c(1,2)],mu_par=par[[4]][c(3,4)],expo.lamb=TRUE,
                           expo.mu=TRUE,cond="crown",f=f,dt=1e-3)
        return(list("bcstdcst"=bcstdcst,"bvardcst"=bvardcst,
                    "bcstdvar"=bcstdvar,"bvardvar"=bvardvar))
}
lemurs.par <- list(c(0.4,0),c(0.4,-0.05,0),c(0.4,0.1,0.05),c(0.4,-0.05,0.1,0.05))
results <- list("lemurs.res"=fit.multi.rpanda(lemurs,lemurs.par))
aicc.table <- matrix(nrow=4,ncol=1,NA)
for(i in 1:1)
    {
        for(j in 1:4)
            {
                aicc.table[j,i] <- results[[i]][[j]]$aicc
            }
}
aicc.table <- data.frame("AICc"=c(aicc.table))
aicc.table$'Speciation Rate' <- c("Constant","Time-varying","Constant","Time-varying")
aicc.table$'Extinction Rate' <- c("Constant","Constant","Time-varying","Time-varying")

par.table <- data.frame(
  "ConstantSpeciation_ConstantExtinction"=c(results[[1]]$bcstdcst$lamb_par[1:2],
                                              results[[1]]$bcstdcst$mu_par[1:2]),
  "VaryingSpeciation_ConstantExtinction"=c(results[[1]]$bvardcst$lamb_par[1:2],
                                             results[[1]]$bvardcst$mu_par[1:2]),
  "ConstantSpeciation_VaryingExtinction"=c(results[[1]]$bcstdvar$lamb_par[1:2],
                                             results[[1]]$bcstdvar$mu_par[1:2]),
  "VaryingSpeciation_VaryingExtinction"=c(results[[1]]$bvardvar$lamb_par[1:2],
                                            results[[1]]$bvardvar$mu_par[1:2]))
row.names(par.table) <- c("$\\lambda_1$","$\\lambda_2$","$\\mu_1$","$\\mu_2$")
aicc.table <- cbind(aicc.table, t(par.table))
aicc.table <- aicc.table[,c(2,3,1,4:7)]
```

Speciation Rate	Extinction Rate	AICc	λ_1	λ_2	μ_1	μ_2
Constant	Constant	539.30	0.25		0.19	
Time-varying	Constant	540.07	0.23	-0.02	0.09	
Constant	Time-varying	539.67	0.22		0.11	0.02
Time-varying	Time-varying	540.65	0.23	0.08	0.22	0.08

The models are characterized by the following functions. $\mu(.)$ describes the extinction rate function and $\lambda(.)$ describes the speciation rate function. Depending on the choice of model, the functions are parameterized by either one or two parameters. The table below shows the estimated parameter values for each model and its Akaike Information Criterion with finite size correction (AICc).

$$\lambda_{constant}(\lambda_1) = \lambda_1$$

$$\lambda_{variable}(x, \lambda_1, \lambda_2) = \lambda_1 * e^{x\lambda_2}$$

$$\mu_{constant}(\mu_1) = \mu_1$$

$$\mu_{variable}(x, \mu_1, \mu_2) = \mu_1 * e^{x\mu_2}$$

The four model predictions are shown below. Allowing extinction rate to vary over time allows for high extinction rates before 30 Ma (red and green lines). The strongest increase in diversification rate is observed

for the model in which both rates are allowed to vary over time (green) with a rapid change in slope near 30 Ma. Allowing only the extinction rate to vary over time (red) shows a weaker increase in diversification rate. It is important to note that these models are all supported equally well/poorly by the data, according to AICc scores (above: they are all within 2 scores of each other).

```
div.time <- c(max(branching.times(lemurs)))
tot_time <- max(branching.times(lemurs))
t <- seq(tot_time-div.time, tot_time, 0.01)
r <- function(fit.bd, t) {fit.bd$f.lamb(t) - fit.bd$f.mu(t)}

plot(-t, r(results$lemurs.res$bcstdcst, t), type = "l", xlab = "Time",
     ylab = "Diversification Rate", ylim=c(-1, 0.2),
             main = "", col="blue",axes=F,lwd=3)
axis(side=1)
axis(side=2)
lines(-t, r(results$lemurs.res$bvardcst, t), col="black",lwd=3)
lines(-t, r(results$lemurs.res$bcstdvar, t),  col="red",lwd=3)
lines(-t, r(results$lemurs.res$bvardvar, t),  col="green",lwd=3)
abline(h=0, col="black", lty=2)
legend(-25,-0.2, c("ScstEcst","SvarEcst","ScstEvar","SvarEvar"),
       lty=c(1,1,1,1),lwd=c(3,3,3,3),col=c("blue","black","red","green"))
```

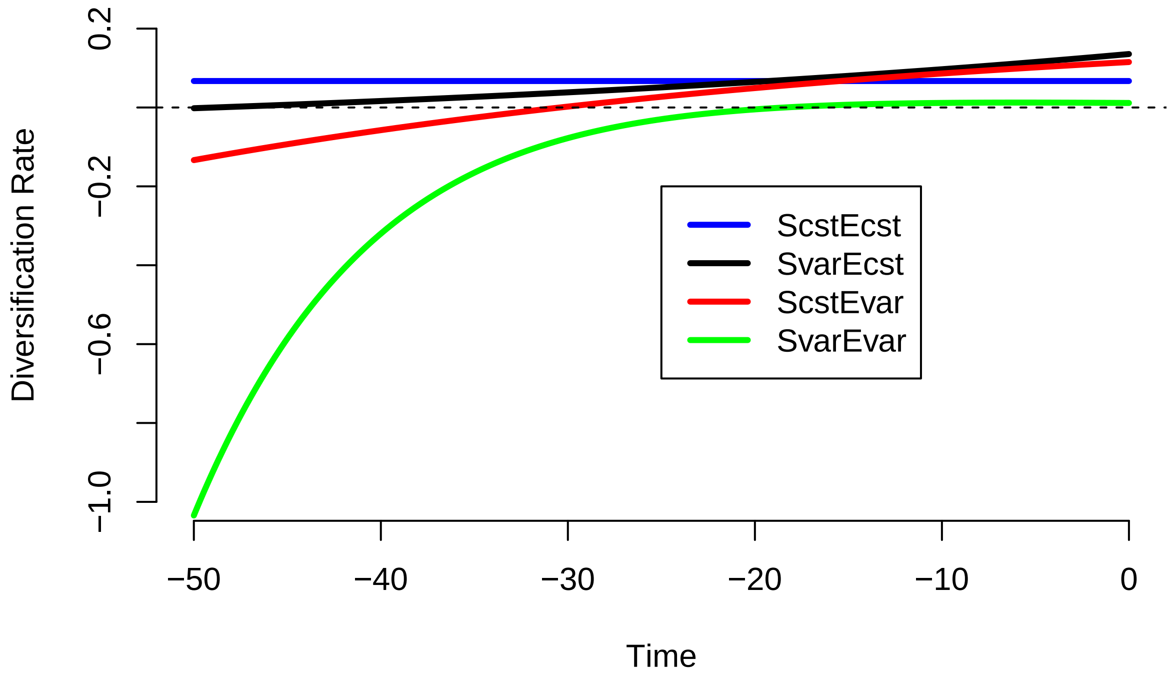

Again we report diversification rates at the beginning and end of the series.

```
fintab=data.frame(rbind(
                  cbind(rbind(cbind(head(-t,1),head(r(results$lemurs.res$bcstdcst, t),1)),
                  cbind(tail(-t,1),tail(r(results$lemurs.res$bcstdcst, t),1))),
```

```r
                    rep("black",2)),
                    cbind(rbind(cbind(head(-t,1),head(r(results$lemurs.res$bvardcst, t),1)),
                    cbind(tail(-t,1),tail(r(results$lemurs.res$bvardcst, t),1))),
                    rep("blue",2)),
                    cbind(rbind(cbind(head(-t,1),head(r(results$lemurs.res$bcstdvar, t),1)),
                    cbind(tail(-t,1),tail(r(results$lemurs.res$bcstdvar, t),1))),
                    rep("red",2)),
                    cbind(rbind(cbind(head(-t,1),head(r(results$lemurs.res$bvardvar, t),1)),
                    cbind(tail(-t,1),tail(r(results$lemurs.res$bvardvar, t),1))),
                    rep("green",2))
                    )
)
names(fintab)=c("time", "div.rate", "color")
fintab
```

```
##   time             div.rate color
## 1    0   0.0671400042693129 black
## 2  -50   0.0671400042693129 black
## 3    0    0.135531819576557  blue
## 4  -50 -0.00184224619562687  blue
## 5    0    0.115231918776262   red
## 6  -50   -0.133433094827325   red
## 7    0   0.0114612966009389 green
## 8  -50    -1.03450254635703 green
```

2.3. BAMM

This is the text of the BAMM control file we used. We use the sampling fraction of 89/120 as above (details in text).

```
# BAMM configuration file for speciation/extinction analysis
# ==========================================================
#
# Format
# ------
#
#     - Each option is specified as: option_name = option_value
#     - Comments start with # and go to the end of the line
#     - True is specified with "1" and False with "0"


################################################################################
# GENERAL SETUP AND DATA INPUT
################################################################################

modeltype = speciationextinction
# Specify "speciationextinction" or "trait" analysis

treefile = godfrey_lemur_phylog2.tre
# File name of the phylogenetic tree to be analyzed

runInfoFilename = run_info.txt
# File name to output general information about this run
```

```
sampleFromPriorOnly = 0
# Whether to perform analysis sampling from prior only (no likelihoods computed)

runMCMC = 1
# Whether to perform the MCMC simulation. If runMCMC = 0, the program will only
# check whether the data file can be read and the initial likelihood computed

simulatePriorShifts = 1
# Whether to simulate the prior distribution of the number of shift events,
# given the hyperprior on the Poisson rate parameter. This is necessary to
# compute Bayes factors

loadEventData = 0
# Whether to load a previous event data file

eventDataInfile = event_data_in.txt
# File name of the event data file to load, used only if loadEventData = 1

initializeModel = 1
# Whether to initialize (but not run) the MCMC. If initializeModel = 0, the
# program will only ensure that the data files (e.g., treefile) can be read

useGlobalSamplingProbability = 1
# Whether to use a "global" sampling probability. If False (0), expects a file
# name for species-specific sampling probabilities (see sampleProbsFilename)

globalSamplingFraction = 0.7416667
# The sampling probability. If useGlobalSamplingFraction = 0, this is ignored
# and BAMM looks for a file name with species-specific sampling fractions

sampleProbsFilename = sample_probs.txt
# File name containing species-specific sampling fractions

# seed = -1
# Seed for the random number generator.
# If not specified (or is -1), a seed is obtained from the system clock

overwrite = 1
# If True (1), the program will overwrite any output files in the current
# directory (if present)


################################################################################
# PRIORS
################################################################################

expectedNumberOfShifts = 1.0
# prior on the number of shifts in diversification
# Suggested values:
#     expectedNumberOfShifts = 1.0 for small trees (< 500 tips)
#     expectedNumberOfShifts = 10 or even 50 for large trees (> 5000 tips)

# Prior block chosen by BAMMtools::setBAMMpriors
```

```
lambdaInitPrior = 2.63470649014838
# Prior (rate parameter of exponential) on the initial lambda value for rate
# regimes

lambdaShiftPrior = 0.0230258509299405
# Prior (std dev of normal) on lambda shift parameter for rate regimes
# You cannot adjust the mean of this distribution (fixed at zero, which is
# equal to a constant rate diversification process)

muInitPrior = 2.63470649014838
# Prior (rate parameter of exponential) on extinction rates

lambdaIsTimeVariablePrior = 1
# Prior (probability) of the time mode being time-variable (vs. time-constant)


################################################################################
# MCMC SIMULATION SETTINGS & OUTPUT OPTIONS
################################################################################

numberOfGenerations = 10000000
# Number of generations to perform MCMC simulation

mcmcOutfile = mcmc_out.txt
# File name for the MCMC output, which only includes summary information about
# MCMC simulation (e.g., log-likelihoods, log-prior, number of processes)

mcmcWriteFreq = 10000
# Frequency in which to write the MCMC output to a file

eventDataOutfile = event_data.txt
# The raw event data (these are the main results). ALL of the results are
# contained in this file, and all branch-specific speciation rates, shift
# positions, marginal distributions etc can be reconstructed from this output.
# See R package BAMMtools for working with this output

eventDataWriteFreq = 10000
# Frequency in which to write the event data to a file

printFreq = 10000
# Frequency in which to print MCMC status to the screen

acceptanceResetFreq = 10000
# Frequency in which to reset the acceptance rate calculation
# The acceptance rate is output to both the MCMC data file and the screen

# outName = BAMM
# Optional name that will be prefixed on all output files (separated with "_")
# If commented out, no prefix will be used


################################################################################
# OPERATORS: MCMC SCALING OPERATORS
```

```
################################################################################

updateLambdaInitScale = 2.0
# Scale parameter for updating the initial speciation rate for each process

updateLambdaShiftScale = 0.1
# Scale parameter for the exponential change parameter for speciation

updateMuInitScale = 2.0
# Scale parameter for updating initial extinction rate for each process

updateEventLocationScale = 0.05
# Scale parameter for updating LOCAL moves of events on the tree
# This defines the width of the sliding window proposal

updateEventRateScale = 4.0
# Scale parameter (proportional shrinking/expanding) for updating
# the rate parameter of the Poisson process


################################################################################
# OPERATORS: MCMC MOVE FREQUENCIES
################################################################################

updateRateEventNumber = 0.1
# Relative frequency of MCMC moves that change the number of events

updateRateEventPosition = 1
# Relative frequency of MCMC moves that change the location of an event on the
# tree

updateRateEventRate = 1
# Relative frequency of MCMC moves that change the rate at which events occur

updateRateLambda0 = 1
# Relative frequency of MCMC moves that change the initial speciation rate
# associated with an event

updateRateLambdaShift = 1
# Relative frequency of MCMC moves that change the exponential shift parameter
# of the speciation rate associated with an event

updateRateMu0 = 1
# Relative frequency of MCMC moves that change the extinction rate for a given
# event

updateRateLambdaTimeMode = 0
# Relative frequency of MCMC moves that flip the time mode
# (time-constant <=> time-variable)

localGlobalMoveRatio = 10.0
# Ratio of local to global moves of events

```

```
################################################################################
# INITIAL PARAMETER VALUES
################################################################################

lambdaInit0 = 0.032
# Initial speciation rate (at the root of the tree)

lambdaShift0 = 0
# Initial shift parameter for the root process

muInit0 = 0.005
# Initial value of extinction (at the root)

initialNumberEvents = 0
# Initial number of non-root processes


################################################################################
# METROPOLIS COUPLED MCMC
################################################################################

numberOfChains = 4
# Number of Markov chains to run

deltaT = 0.01
# Temperature increment parameter. This value should be > 0
# The temperature for the i-th chain is computed as 1 / [1 + deltaT * (i - 1)]

swapPeriod = 1000
# Number of generations in which to propose a chain swap

chainSwapFileName = chain_swap.txt
# File name in which to output data about each chain swap proposal.
# The format of each line is [generation],[rank_1],[rank_2],[swap_accepted]
# where [generation] is the generation in which the swap proposal was made,
# [rank_1] and [rank_2] are the chains that were chosen, and [swap_accepted] is
# whether the swap was made. The cold chain has a rank of 1.


################################################################################
# NUMERICAL AND OTHER PARAMETERS
################################################################################

minCladeSizeForShift = 1
# Allows you to constrain location of possible rate-change events to occur
# only on branches with at least this many descendant tips. A value of 1
# allows shifts to occur on all branches.

segLength = 0.02
# Controls the "grain" of the likelihood calculations. Approximates the
# continuous-time change in diversification rates by breaking each branch into
# a constant-rate diversification segments, with each segment given a length
# determined by segLength. segLength is in units of the root-to-tip distance of
```

```
# the tree. So, if the segLength parameter is 0.01, and the crown age of your
# tree is 50, the "step size" of the constant rate approximation will be 0.5.
# If the value is greater than the branch length (e.g., you have a branch of
# length < 0.5 in the preceding example) BAMM will not break the branch into
# segments but use the mean rate across the entire branch.
```

We then inspect the chain for adequate mixing.

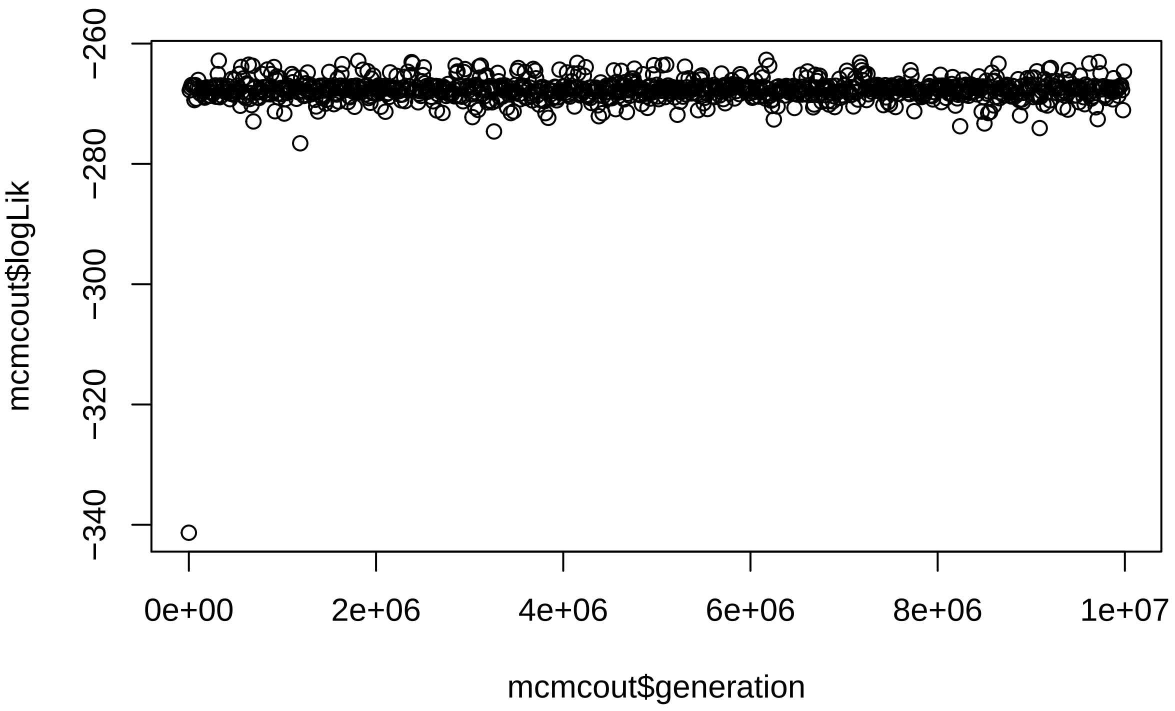

This looks like adequate mixing, so we conclude we have run it for a sufficient number of generations. We then consider the effective sample size.

```
burnstart <- floor(0.1 * nrow(mcmcout))
postburn <- mcmcout[burnstart:nrow(mcmcout), ]
effectiveSize(postburn$N_shifts)
```

```
##     var1
## 682.8889
```

```
effectiveSize(postburn$logLik)
```

```
##     var1
## 788.0687
```

The effective sample size is well over the recommended cutoff of 200. We conclude that convergence has been achieved. Code below is modified lightly from BAMMtools package functions.

```
events.lemurs <- read.csv("BAMM runs\\5_15_2017_Lemurs\\event_data.txt")
ed <- getEventData(lemurs, events.lemurs, burnin = 0.1)
```

```
## Processing event data from data.frame
##
## Discarded as burnin: GENERATIONS <  990000
## Analyzing  901  samples from posterior
##
## Setting recursive sequence on tree...
##
## Done with recursive sequence
```

```r
rmat <- getRateThroughTimeMatrix(ed, nslices = 100,
            nodetype = "include")
        rate <- rmat$lambda - rmat$mu
        ratelabel <- "Diversification Rate"
    maxTime <- max(rmat$times)
    nanCol <- apply(rate, 2, function(x) any(is.nan(x)))
    rate <- rate[, which(nanCol == FALSE)]
    rmat$times <- rmat$times[which(nanCol == FALSE)]
    rmat$times <- max(rmat$times) - rmat$times

    #intervals is CI
intervals=c(0.025, 0.975)
    if (!is.null(intervals)) {
        mm <- apply(rate, MARGIN = 2, quantile, intervals)
        poly <- list()
        q1 <- 1
        q2 <- nrow(mm)
        repeat {
            if (q1 >= q2) {
                break
            }
            a <- as.data.frame(cbind(rmat$times, mm[q1, ]))
            b <- as.data.frame(cbind(rmat$times, mm[q2, ]))
            b <- b[rev(rownames(b)), ]
            colnames(a) <- colnames(b) <- c("x", "y")
            poly[[q1]] <- rbind(a, b)
            q1 <- q1 + 1
            q2 <- q2 - 1
        }
    }
    useMedian=TRUE
    avg <- unlist(apply(rate, 2, median))
    smooth=TRUE
    smoothParam = 0.2
    if (smooth) {
        for (i in 1:length(poly)) {
            p <- poly[[i]]
            rows <- nrow(p)
            p[1:rows/2, 2] <- loess(p[1:rows/2, 2] ~ p[1:rows/2,
                1], span = smoothParam)$fitted
            p[(rows/2):rows, 2] <- loess(p[(rows/2):rows, 2] ~
                p[(rows/2):rows, 1], span = smoothParam)$fitted
            poly[[i]] <- p
        }
        avg <- loess(avg ~ rmat$time, span = smoothParam)$fitted
```

```
  }
ylim=xlim="auto"
      ylim=c(-1,0.2)
plot.new()
xMin <- maxTime
              xMax <- 0
              yMin <- ylim[1]
              yMax <- ylim[2]
plot.window(xlim = c(xMin, xMax), ylim = c(yMin,
              yMax))
          xticks=yticks=NULL
              axis(at = c(round(1.2 * xMin), axTicks(1)), cex.axis = 1,
                side = 1)
              axis(at = round(c(-1, axTicks(2)),2), cex.axis = 1,
                las = 1, side = 2)
              axis.labels=TRUE
              xline = 3
              yline = 3
          if (axis.labels) {
              mtext(side = 1, text = "Time",
                line = xline)
              mtext(side = 2, text = "Diversification Rate", line = yline)
          }
               intervalCol='gray80'
              opacity=1
      if (!is.null(intervals)) {
          for (i in 1:length(poly)) {
              polygon(x = poly[[i]][, 1], y = poly[[i]][, 2],
                col = transparentColor(intervalCol, opacity),
                border = NA)
          }
      }
      lines(x = rmat$time, y = avg, lwd = 3, col = "black")
      abline(h=avg[length(avg)],col="blue",lwd=3)
      legend(25,-0.2, c("Constant Rate","BAMM"),lty=c(1,1),lwd=c(3,3),
             col=c("blue","black"))
```

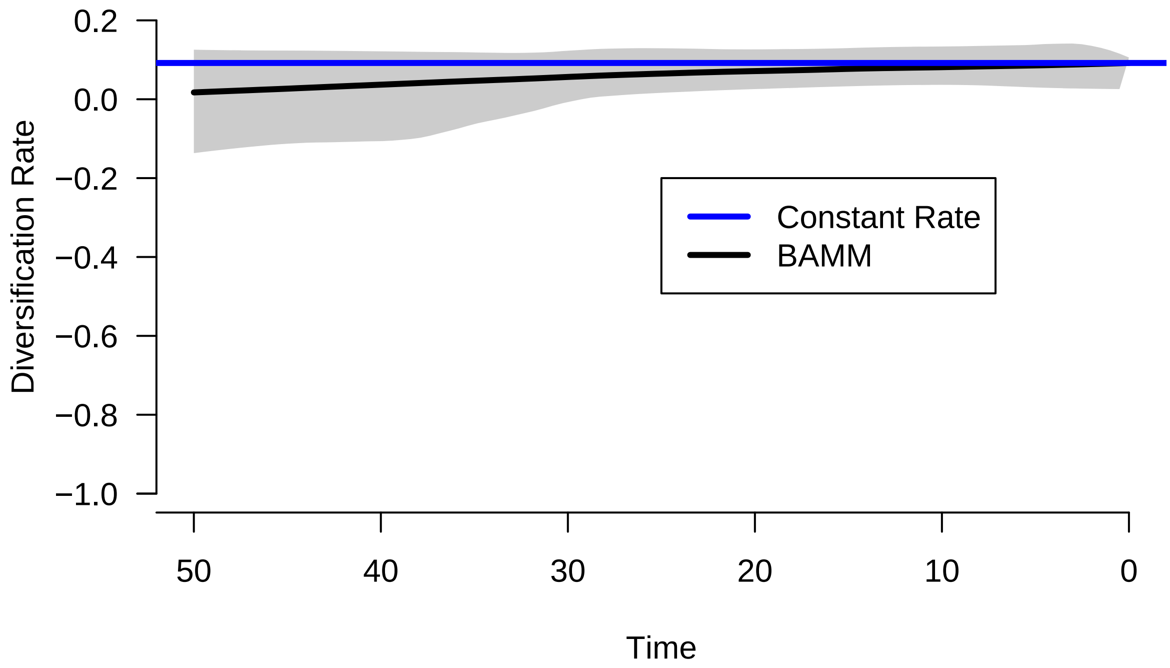

Now we report the diversification rates at the beginning and end of the series.

```
fintab=data.frame(rbind(cbind(head(-rmat$times,1),head(avg,1)),
             cbind(tail(-rmat$times,1),tail(avg,1))))
names(fintab)=c("time", "div.rate")
fintab
```

```
##        time   div.rate
## 1 -50.00001 0.01733618
## 2   0.00000 0.09197529
```

2.4. TESS

The model we run has a strong prior on the magnitude of the single mass extinction. We hypothesize that the mean survival was 5% (95% CI: 0.01-0.10).

```
# Specify the mean and standard deviation of the lognormal
# prior on the speciation rate in real space
speciationPriorMu <- 0.2
#speciationPriorSigma <- 10
speciationPriorSigma <- 0.5
# Specify the mean and standard deviation of the lognormal
# prior on the extinction rate in real space

extinctionPriorMu <- 0.15
extinctionPriorSigma <- 0.5
#We then transform the hyperparameters to reflect the mean and standard of the
```

```r
#log-transformed speciation and extinction rates. These are the mu and sigma parameters
#of the lognormal priors on speciation and extinction rates.
# Transform the priors on the speciation rate into log space.
speciationRatePriorMean <- log((speciationPriorMu^2)/sqrt(speciationPriorSigma^2+
                                                            speciationPriorMu^2))
speciationRatePriorStDev <- sqrt( log(1+speciationPriorSigma^2/(speciationPriorMu^2)))
# Transform the priors on the extinction rate into log space.
extinctionRatePriorMean <- log((extinctionPriorMu^2)/sqrt(extinctionPriorSigma^2+
                                                            extinctionPriorMu^2))
extinctionRatePriorStDev <- sqrt( log(1+extinctionPriorSigma^2/(extinctionPriorMu^2)))

# Finally, we need to specify the prior density on the survival probability of a massextinction
# event. This value reflects the probability that a lineage survives a
# particular mass-extinction event, and therefore must be between 0 (each lineage
# will always go extinct) and 1 (each lineage will always survive). A convenient prior
# density for this parameter is the beta distribution, which has two shape parameters,
# alpha and beta. We use the definition of what constitutes to a mass extinction to inform
# our prior density on this parameter.
# We begin parameterizing this distribution by specifying the expected survival
# probability.
expectedSurvivalProbability <- 0.05

# Using the expected survival probability, we compute the alpha and beta parameters of
# the beta distribution. We set the value of beta to be large, which focuses the prior
# density more tightly around the expected survival probability. Then, we compute
# alpha based on the expected survival probability and the specified beta value.
pMassExtinctionPriorShape2 <- 100
pMassExtinctionPriorShape1 <- - pMassExtinctionPriorShape2 *expectedSurvivalProbability /
  (expectedSurvivalProbability - 1)
# We can inspect this beta distribution to confirm that it accurately reflects our
# prior belief regarding the survival probability
# Plot the density function of our beta distribution.
curve(dbeta(x,shape1=pMassExtinctionPriorShape1,shape2=pMassExtinctionPriorShape2),
      n=1001, xlab='survival probability',ylab='density',las=1)
# Plot the 95% prior interval on the survival probability.
abline(v = qbeta(c(0.025,0.975),shape1=pMassExtinctionPriorShape1,
                 shape2=pMassExtinctionPriorShape2),lty=2)
```

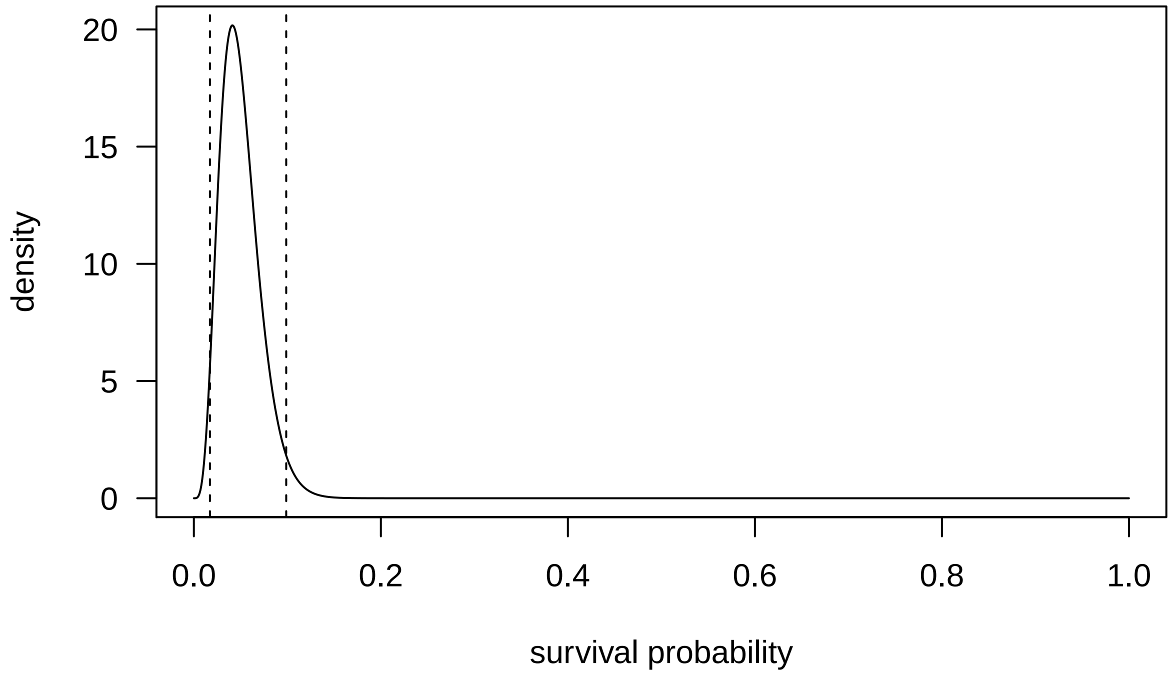


```r
# This beta distribution seems to reflect our prior belief that the survival probability
# is expected to be ca. 5%, but can range from about 1% to about 10%.
# Having specified prior distributions for all of the parameters of the CoMET model,
# we can now perform an analysis.

samplingFraction=0.74
numExpectedMassExtinctions=1
numExpectedRateChanges=1
set.seed(12345)

#make ultrametric
nnls<-nnls.tree(cophenetic(lemurs),lemurs,rooted=TRUE)
```

```
## [1] "RSS: 6.76176366918855e-08"
```

```r
tess.analysis(nnls,
empiricalHyperPriors = FALSE,
initialSpeciationRate = speciationPriorMu,
speciationRatePriorMean = speciationRatePriorMean,
speciationRatePriorStDev = speciationRatePriorStDev,
initialExtinctionRate = extinctionPriorMu,
extinctionRatePriorMean = extinctionRatePriorMean,
extinctionRatePriorStDev = extinctionRatePriorStDev,
samplingProbability = samplingFraction,
numExpectedRateChanges = numExpectedRateChanges,
numExpectedMassExtinctions = numExpectedMassExtinctions,
pMassExtinctionPriorShape1 = pMassExtinctionPriorShape1,
```

```
pMassExtinctionPriorShape2 = pMassExtinctionPriorShape2,
MAX_ITERATIONS = 10e+06, BURNIN = 1e+05,
dir = "tess_analysis_thinpriors_mu_lambda")
```

```
tess.analysis(nnls,
empiricalHyperPriors = FALSE,
initialSpeciationRate = speciationPriorMu,
speciationRatePriorMean = speciationRatePriorMean,
speciationRatePriorStDev = speciationRatePriorStDev,
initialExtinctionRate = extinctionPriorMu,
extinctionRatePriorMean = extinctionRatePriorMean,
extinctionRatePriorStDev = extinctionRatePriorStDev,
samplingProbability = samplingFraction,
numExpectedRateChanges = numExpectedRateChanges,
numExpectedMassExtinctions = numExpectedMassExtinctions,
pMassExtinctionPriorShape1 = pMassExtinctionPriorShape1,
pMassExtinctionPriorShape2 = pMassExtinctionPriorShape2,
MAX_ITERATIONS = 10e+06, BURNIN = 1e+05,
dir = "tess_analysis_thinpriors_mu_lambda_run2")
```

```
tess.analysis(nnls,
empiricalHyperPriors = FALSE,
initialSpeciationRate = speciationPriorMu,
speciationRatePriorMean = speciationRatePriorMean,
speciationRatePriorStDev = speciationRatePriorStDev,
initialExtinctionRate = extinctionPriorMu,
extinctionRatePriorMean = extinctionRatePriorMean,
extinctionRatePriorStDev = extinctionRatePriorStDev,
samplingProbability = samplingFraction,
numExpectedRateChanges = numExpectedRateChanges,
numExpectedMassExtinctions = numExpectedMassExtinctions,
pMassExtinctionPriorShape1 = pMassExtinctionPriorShape1,
pMassExtinctionPriorShape2 = pMassExtinctionPriorShape2,
MAX_ITERATIONS = 10e+06, BURNIN = 1e+05,
dir = "tess_analysis_thinpriors_mu_lambda_run3")
```

```
tess.analysis(nnls,
empiricalHyperPriors = FALSE,
initialSpeciationRate = speciationPriorMu,
speciationRatePriorMean = speciationRatePriorMean,
speciationRatePriorStDev = speciationRatePriorStDev,
initialExtinctionRate = extinctionPriorMu,
extinctionRatePriorMean = extinctionRatePriorMean,
extinctionRatePriorStDev = extinctionRatePriorStDev,
samplingProbability = samplingFraction,
numExpectedRateChanges = numExpectedRateChanges,
numExpectedMassExtinctions = numExpectedMassExtinctions,
pMassExtinctionPriorShape1 = pMassExtinctionPriorShape1,
pMassExtinctionPriorShape2 = pMassExtinctionPriorShape2,
MAX_ITERATIONS = 10e+06, BURNIN = 1e+05,
dir = "tess_analysis_thinpriors_mu_lambda_run4")
```

First we diagnose the convergence.

```
numExpectedRateChanges=1
numExpectedMassExtinctions=1
output <- tess.process.output("tess_analysis_thinpriors_mu_lambda",
                              numExpectedRateChanges = numExpectedRateChanges,
                              numExpectedMassExtinctions = numExpectedMassExtinctions,
                              criticalBayesFactors = c(2,6,10))

output2 <- tess.process.output("tess_analysis_thinpriors_mu_lambda_run2",
                              numExpectedRateChanges = numExpectedRateChanges,
                              numExpectedMassExtinctions = numExpectedMassExtinctions,
                              criticalBayesFactors = c(2,6,10))

output3 <- tess.process.output("tess_analysis_thinpriors_mu_lambda_run3",
                              numExpectedRateChanges = numExpectedRateChanges,
                              numExpectedMassExtinctions = numExpectedMassExtinctions,
                              criticalBayesFactors = c(2,6,10))

output4 <- tess.process.output("tess_analysis_thinpriors_mu_lambda_run4",
                              numExpectedRateChanges = numExpectedRateChanges,
                              numExpectedMassExtinctions = numExpectedMassExtinctions,
                              criticalBayesFactors = c(2,6,10))

l=list(output, output2, output3, output4)
tess.plot.multichain.diagnostics(l,parameters="net-diversification rates")
```

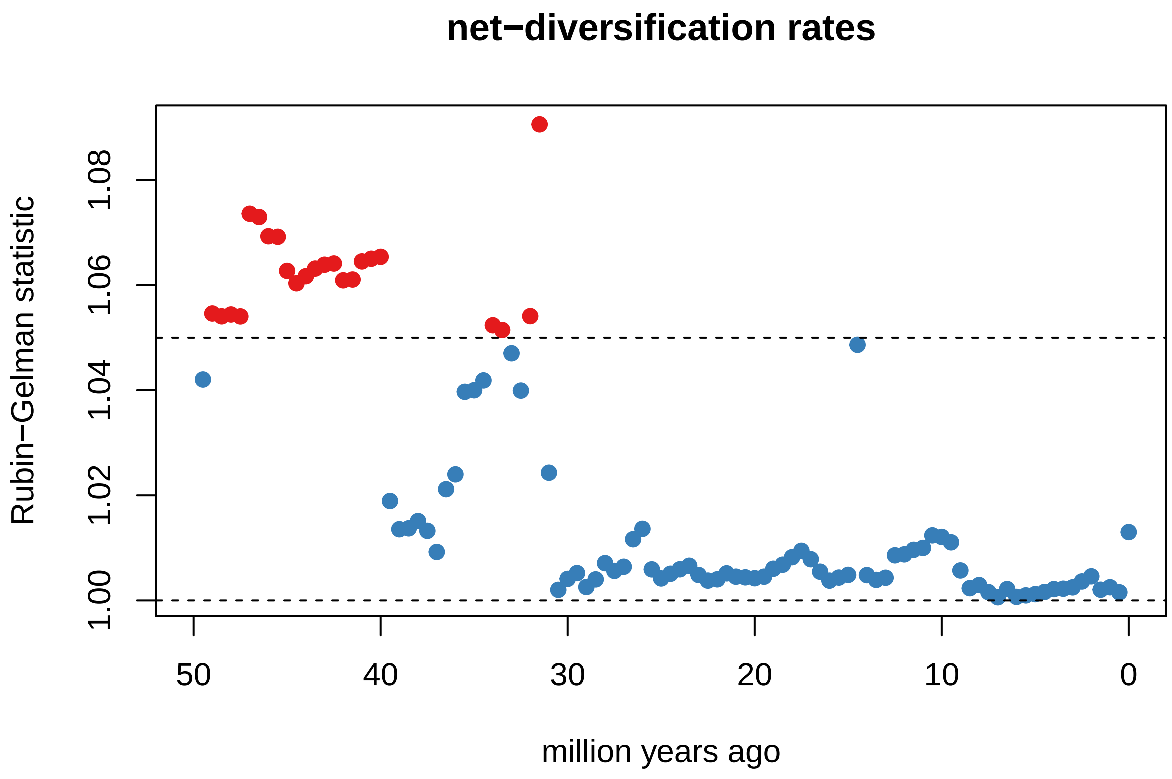

Gelman-Rubin statistics are a measure of within-chain variation to between-chain variation. Borderline

unsatisfactory convergence of Rhat values near 1.05 for pre 40 Ma diversification rates would likely become satisfactory with a few more chains added. This makes sense too, the part of the tree before 40 Ma is poorly parameterized so that is likely where convergence errors would show up. In any case, we decide to use the more lenient threshold of Rhat values of 1.1 to identify converged parameters. Thus, we can look at this plot and say that there is no drastically problematic convergence.

We must select one chain to continute with to use the `tess.plot.output` functions and the next convergence diagnostics. We now look at each chain individually.

```
tess.plot.singlechain.diagnostics(output, parameters = "net-diversification rates")
```

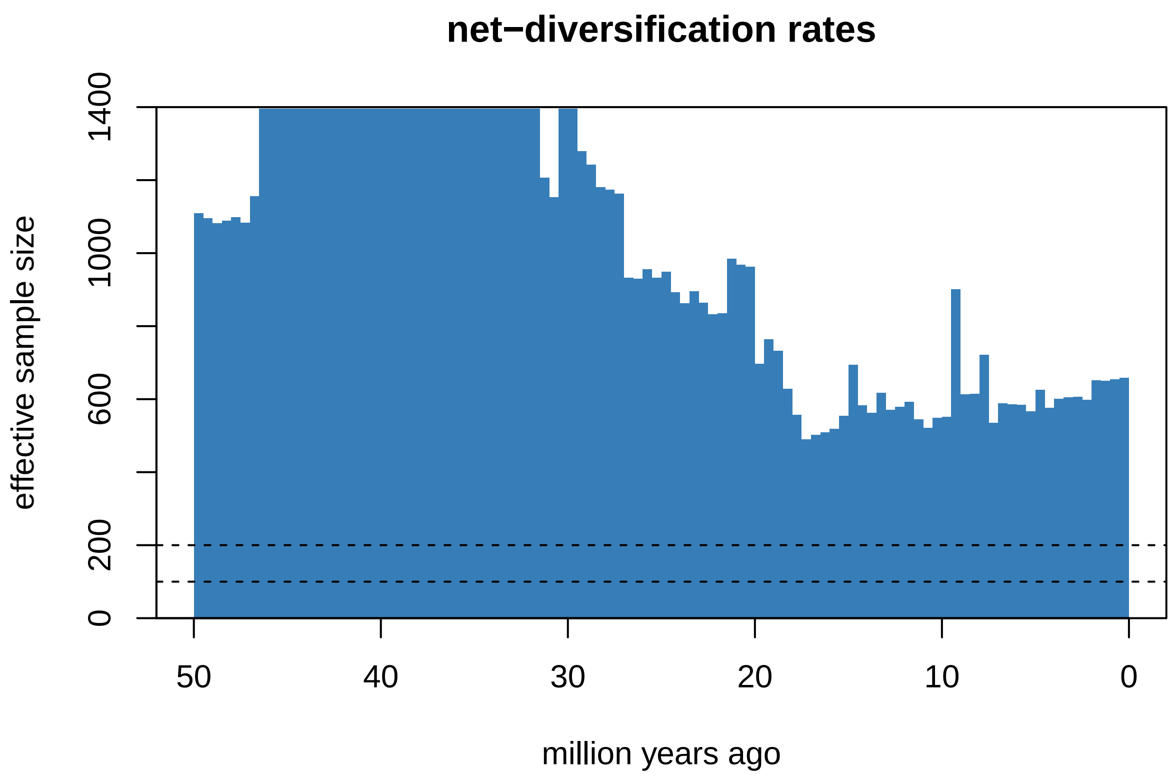

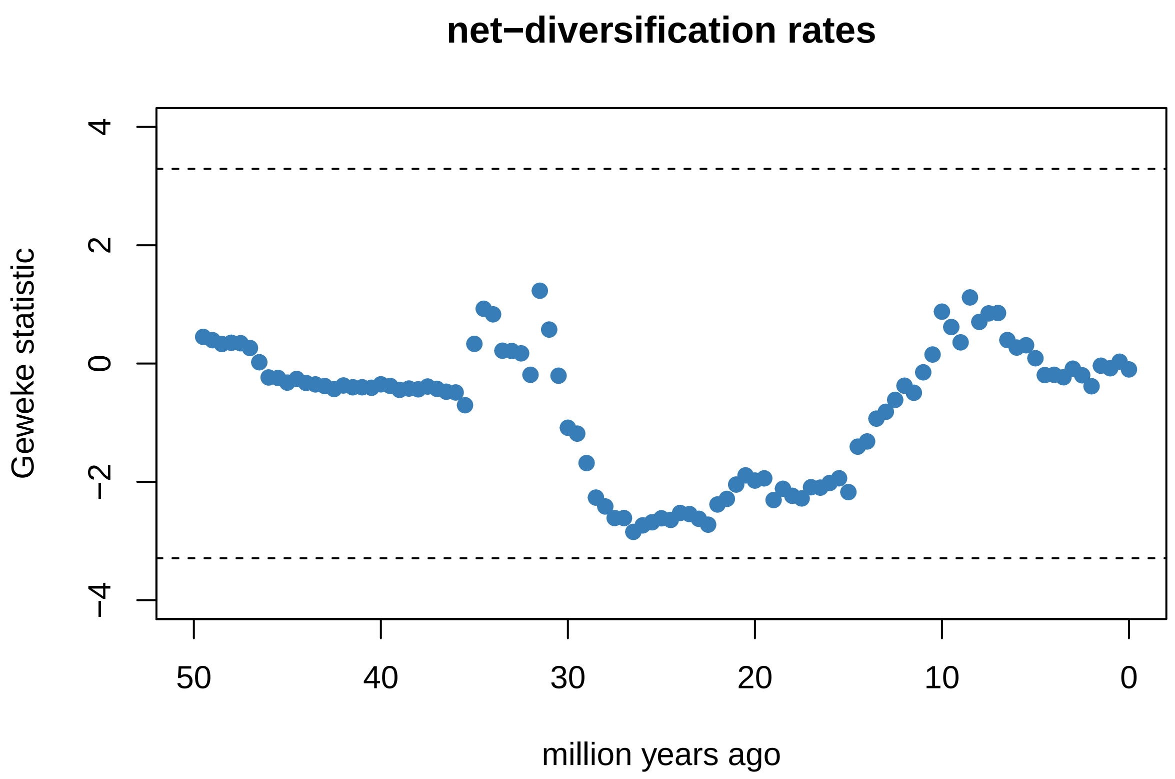


```r
tess.plot.singlechain.diagnostics(output2, parameters = "net-diversification rates")
```

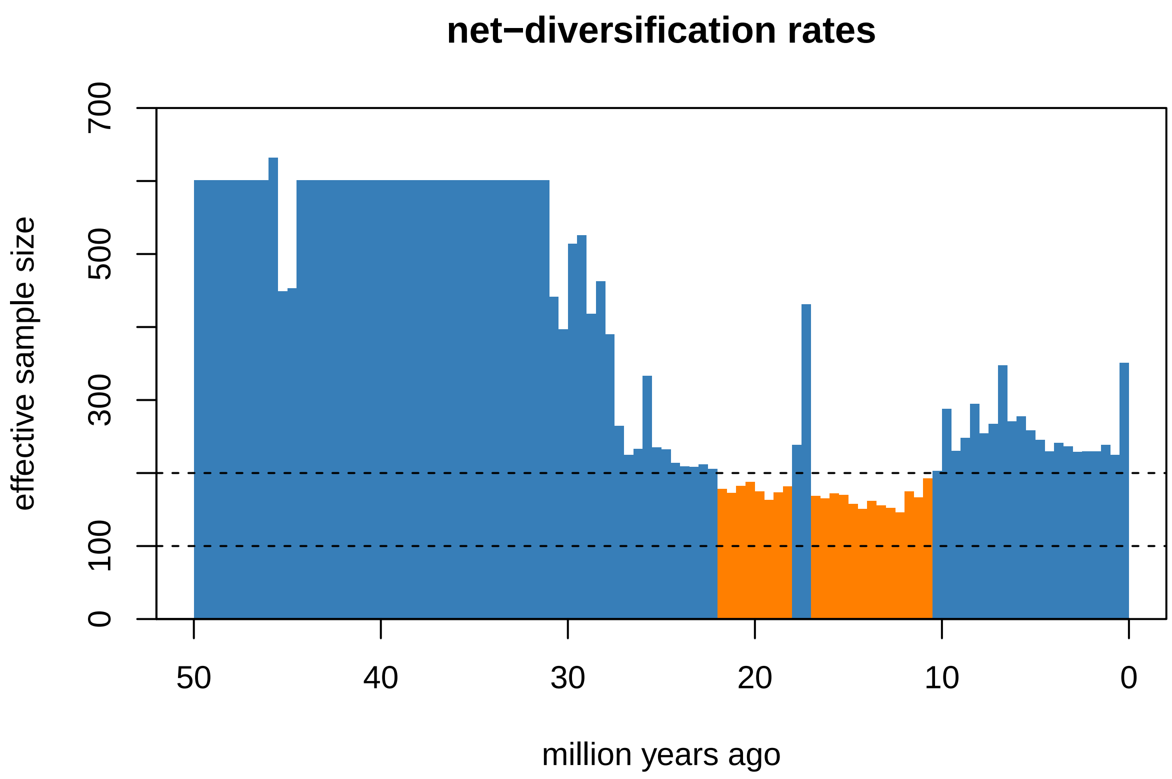
net−diversification rates
effective sample size
0
100
300
500
700
50
40
30
20
10
0
million years ago

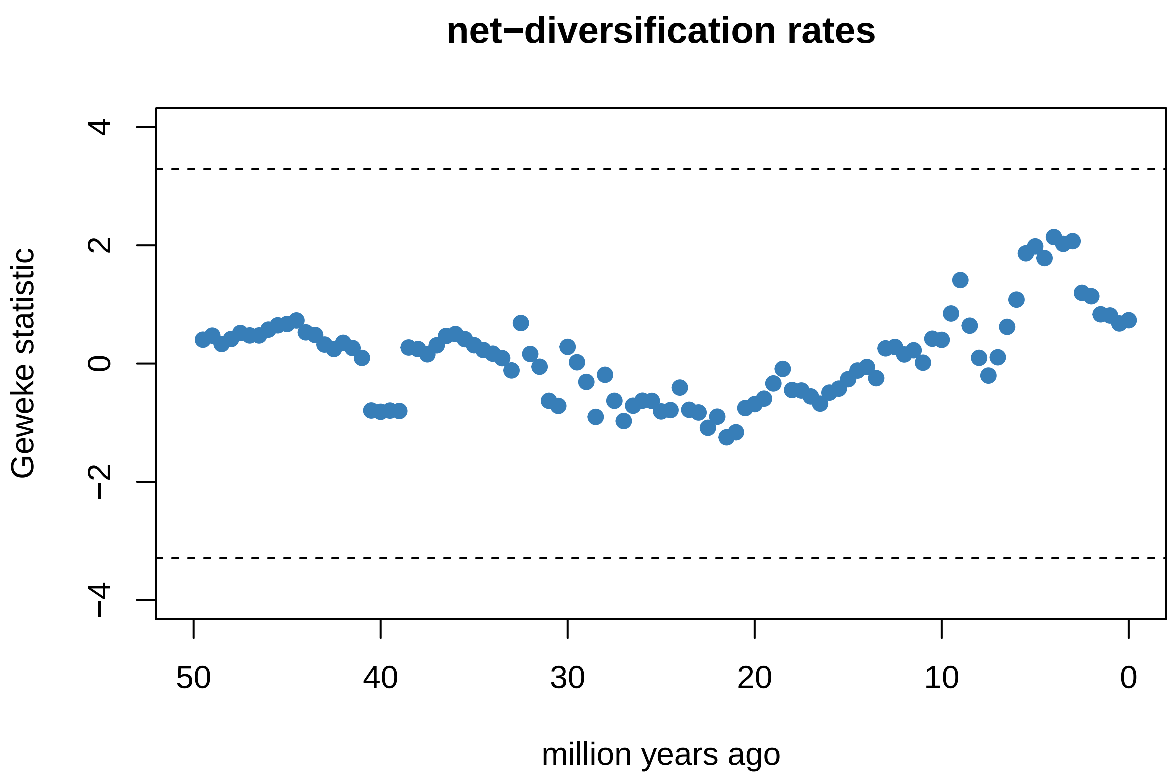


```
tess.plot.singlechain.diagnostics(output3, parameters = "net-diversification rates")
```

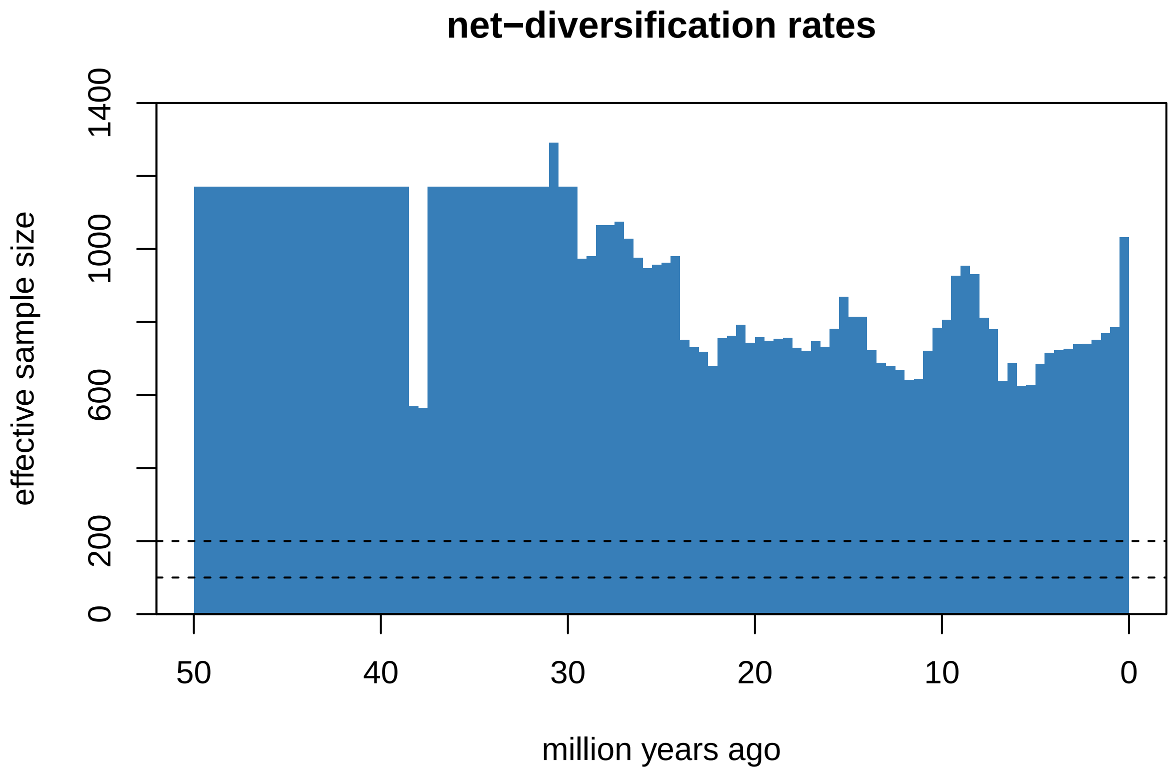

net−diversification rates
effective sample size
0
200
600
1000
1400
50
40
30
20
10
0
million years ago

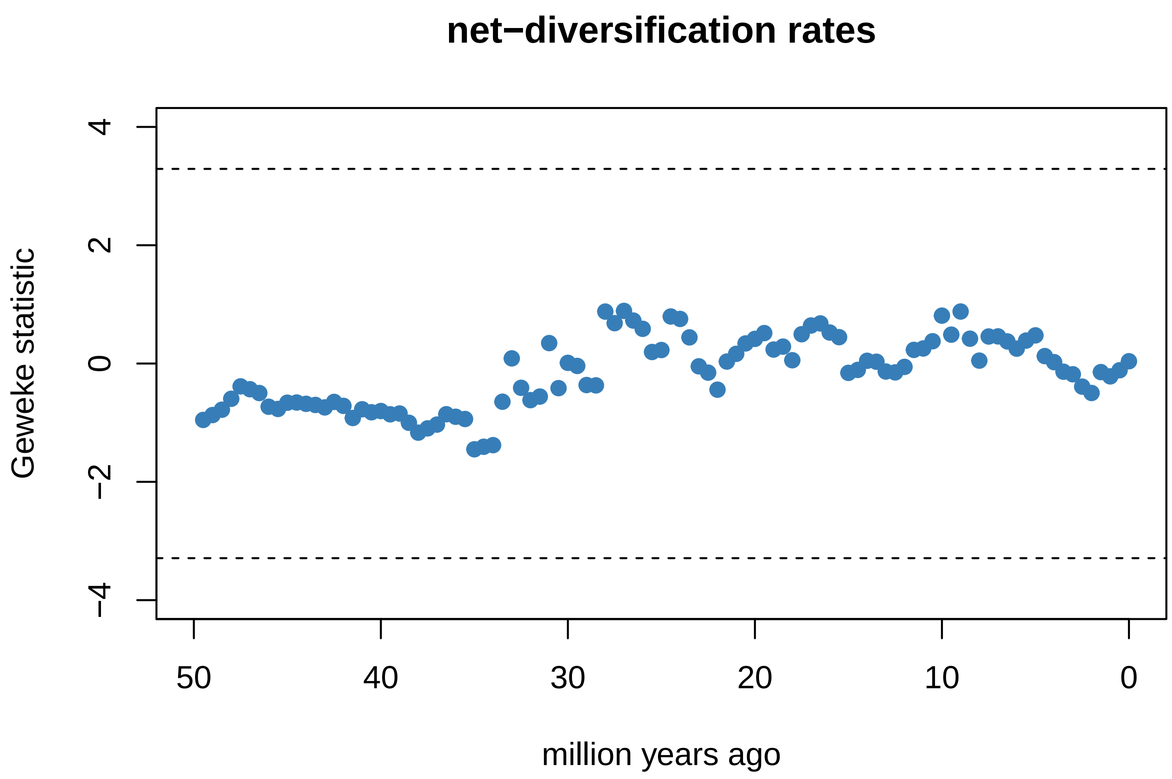


```
tess.plot.singlechain.diagnostics(output4, parameters = "net-diversification rates")
```

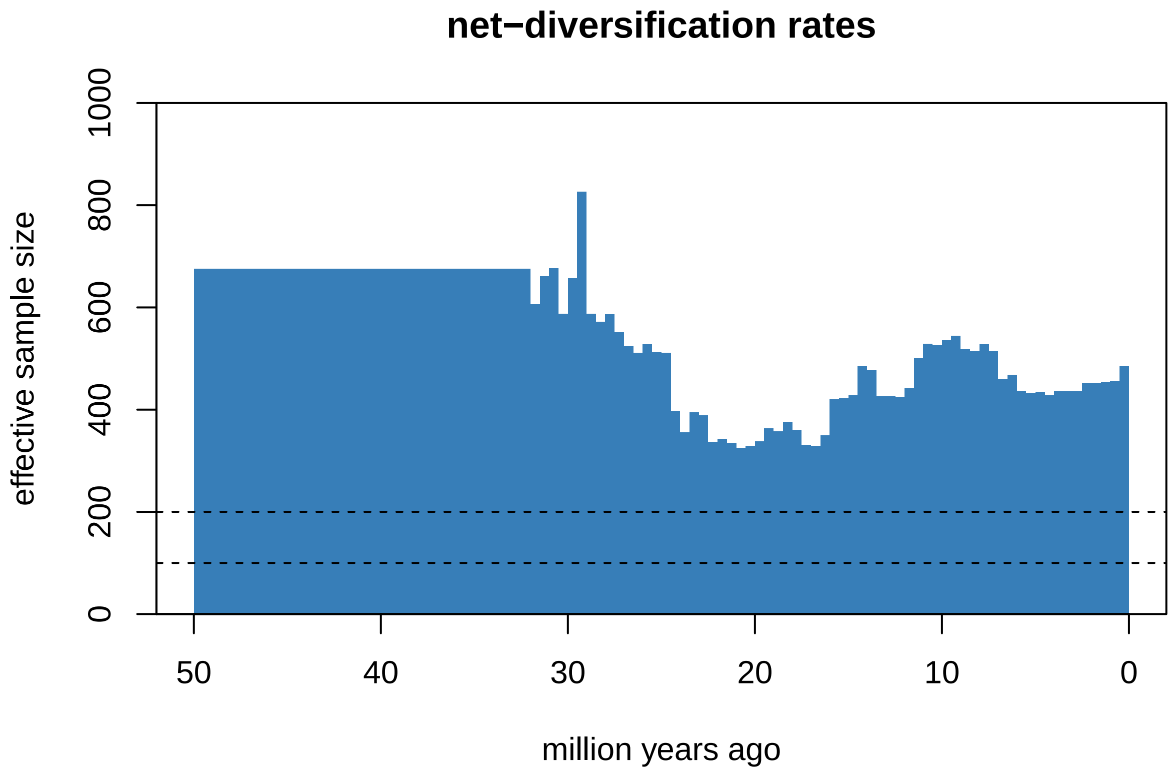
net−diversification rates
effective sample size
0
200
400
600
800
1000
50
40
30
20
10
0
million years ago

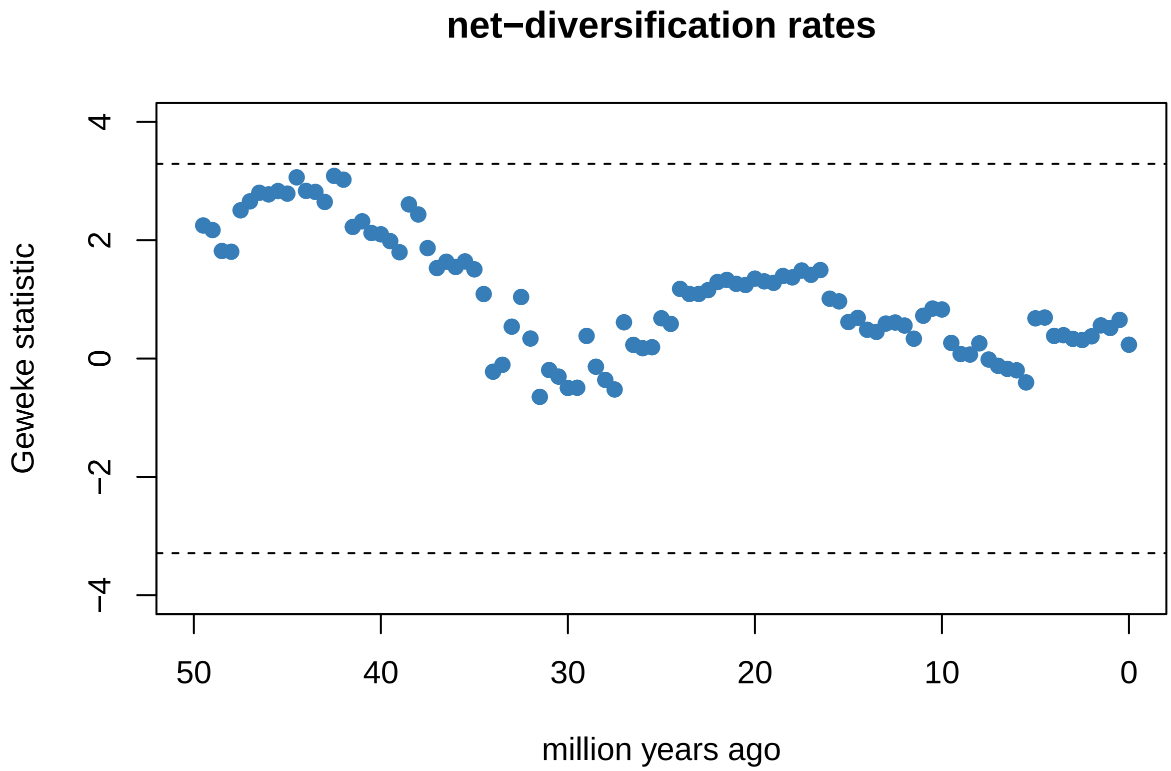

Geweke statistics compare pre burn-in parameter estimates to post-burnin samples with a 2 sample mean test. We thus infer that the burn-in has gone on sufficiently long enough for chains 3 and 4, but not 1 and 2. The effective sample size is sufficiently high for chains 3 and 4 but not 1 and 2.

We now can look if the output from the single chains are different.

```
tess.plot.output(output, "net-diversification rates")
```

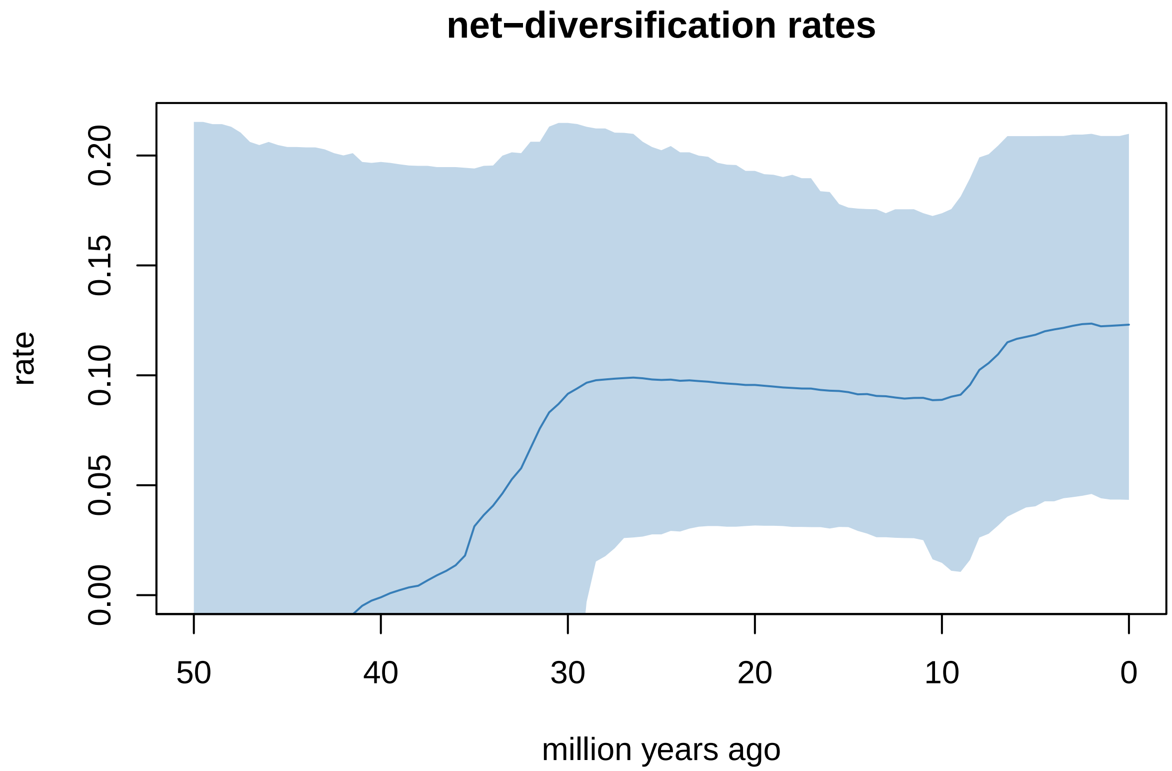


```r
tess.plot.output(output2, "net-diversification rates")
```

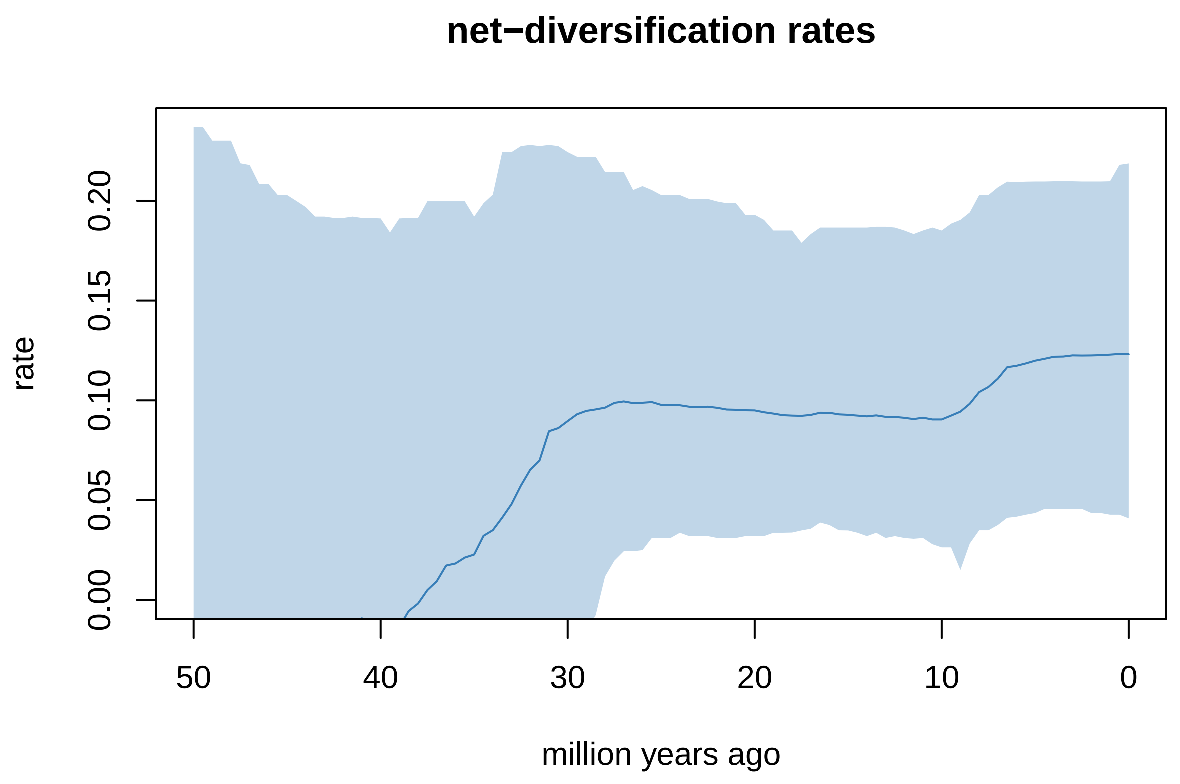


```r
tess.plot.output(output3, "net-diversification rates")
```

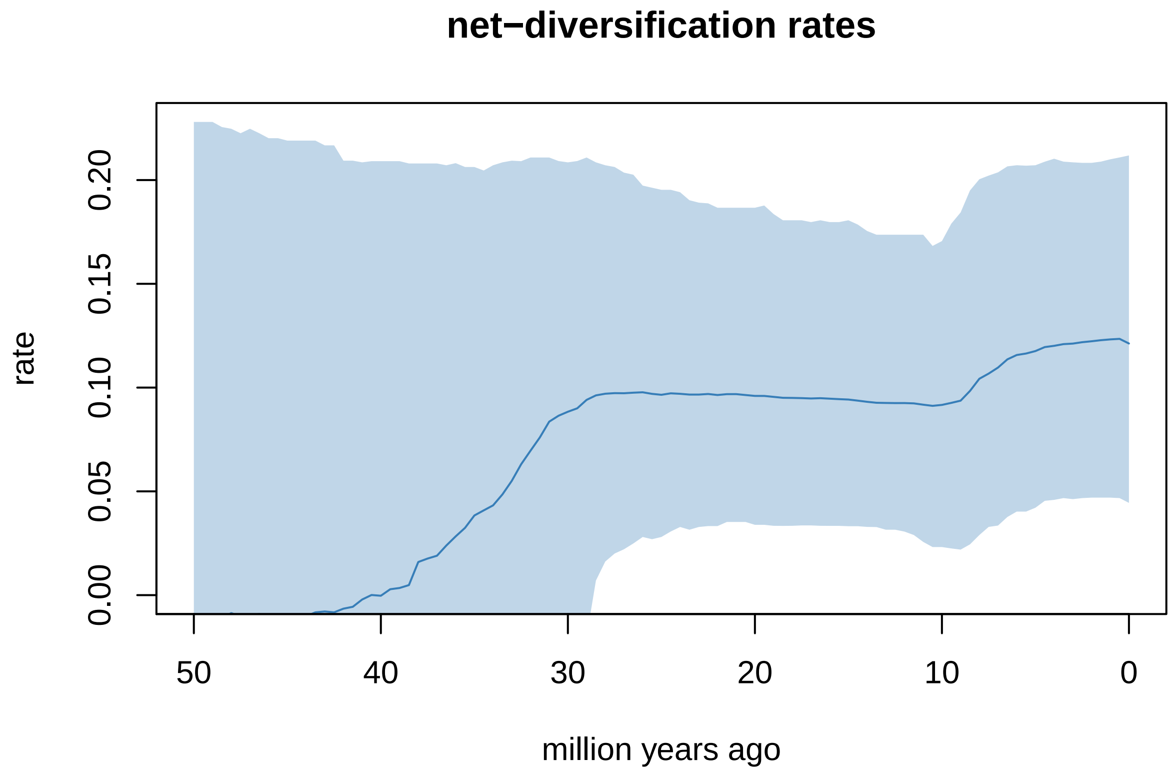


```
tess.plot.output(output4, "net-diversification rates")
```

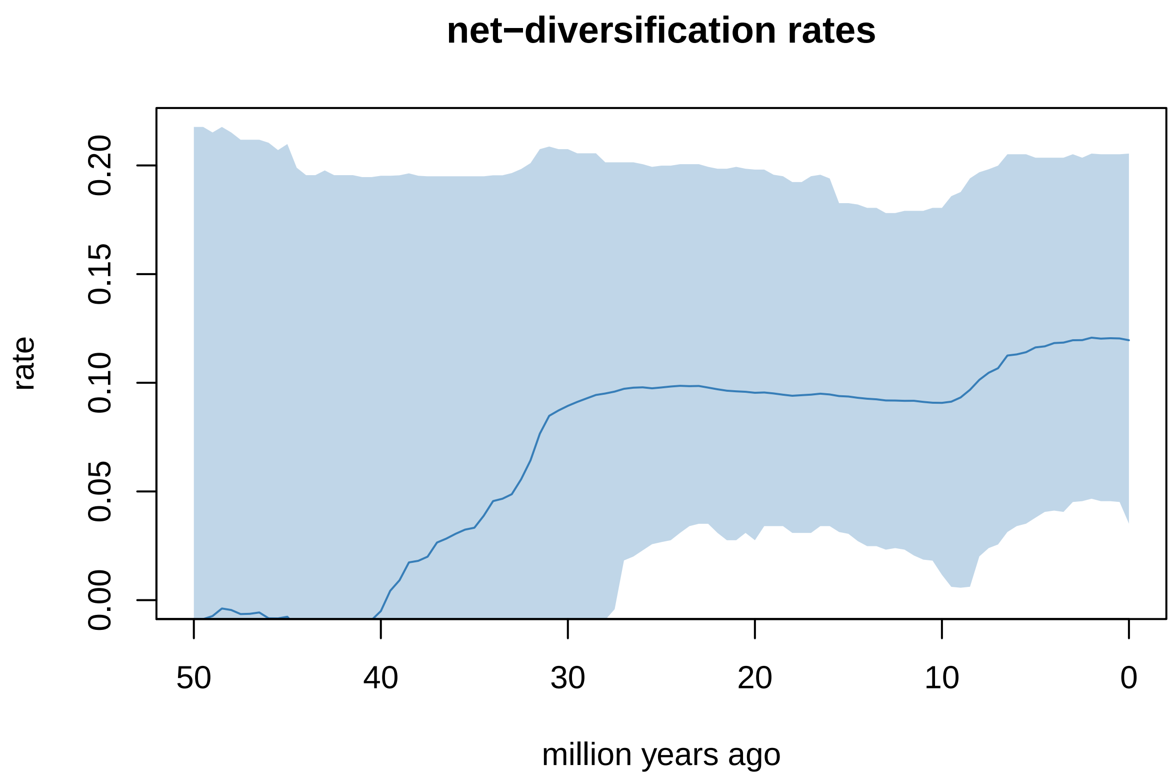

We conclude that the choice of chain does not matter much in the inference. We choose chain 3 to work with, as this was one of the two chains above that showed more satisfactory convergence. Now we can customise the plot from the diversification rate plot from chain 3. And display the point estimates over time.

```
col <- c(`speciation rates` = "#984EA3",
         `speciation shift times` = "#984EA3",
            `speciation Bayes factors` = "#984EA3",
         `extinction rates` = "#E41A1C",
            `extinction shift times` = "#E41A1C",
         `extinction Bayes factors` = "#E41A1C",
            `net-diversification rates` = "#377EB8",
         `relative-extinction rates` = "#FF7F00",
            `mass extinction times` = "#4DAF4A",
         `mass extinction Bayes factors` = "#4DAF4A")
treeAge <- max(branching.times(output3$tree))
numIntervals <- length(output3$intervals) - 1
plotAt <- 0:numIntervals
intervalSize <- treeAge/numIntervals
labels <- pretty(c(0, treeAge))
labelsAt <- numIntervals - (labels/intervalSize)
type="net-diversification rates"
thisOutput <- output3[[type]]
meanThisOutput <- colMeans(thisOutput)
quantilesThisOutput <- apply(thisOutput, 2, quantile, prob = c(0.025, 0.975))
ylim <- c(-0.8, max(quantilesThisOutput))

plot.new()
```

```
plot.window(xlim = range((plotAt-100)/2), ylim = ylim)
lines(x = (plotAt-100)/2, y = c(meanThisOutput[1], meanThisOutput),
      col = col[type],  lwd=3)
polygon(x = c(c(0:ncol(quantilesThisOutput)-100)/2,
              c(c(ncol(quantilesThisOutput):0)-100)/2),
  y = c(c(quantilesThisOutput[1, 1], quantilesThisOutput[1, ]),
      rev(c(quantilesThisOutput[2, 1], quantilesThisOutput[2,]))),
  border = NA, col = paste(col[type], col.alpha=50,
  sep = ""))
axis(at = c(round(1.2 * 50), axTicks(1)), side = 1)
axis(at = round(c(-1, axTicks(2)),2), las = 1, side = 2)
mtext(side = 1, text = "Time",line = 3)
mtext(side = 2, text = "Diversification Rate", line = 3)
```

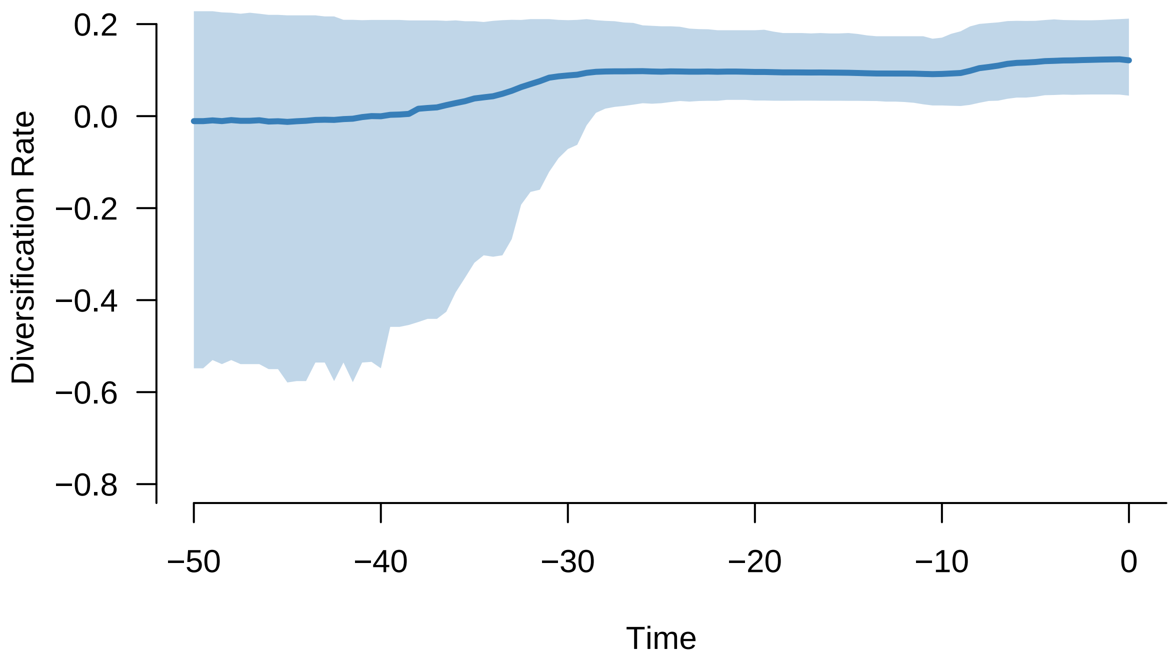


```
cbind((plotAt-100)/2,
      c(meanThisOutput[1], meanThisOutput))
```

```
##         [,1]          [,2]
##   [1,] -50.0 -1.090961e-02
##   [2,] -49.5 -1.090961e-02
##   [3,] -49.0 -9.306996e-03
##   [4,] -48.5 -1.087969e-02
##   [5,] -48.0 -8.676070e-03
##   [6,] -47.5 -1.022319e-02
##   [7,] -47.0 -1.018382e-02
##   [8,] -46.5 -9.000862e-03
```

```
##   [9,] -46.0 -1.178980e-02
##  [10,] -45.5 -1.118263e-02
##  [11,] -45.0 -1.255643e-02
##  [12,] -44.5 -1.116730e-02
##  [13,] -44.0 -1.015784e-02
##  [14,] -43.5 -8.293733e-03
##  [15,] -43.0 -7.872477e-03
##  [16,] -42.5 -8.281220e-03
##  [17,] -42.0 -6.521469e-03
##  [18,] -41.5 -5.622172e-03
##  [19,] -41.0 -2.106956e-03
##  [20,] -40.5  5.816768e-05
##  [21,] -40.0 -2.981205e-04
##  [22,] -39.5  2.835504e-03
##  [23,] -39.0  3.435245e-03
##  [24,] -38.5  4.826497e-03
##  [25,] -38.0  1.596544e-02
##  [26,] -37.5  1.762938e-02
##  [27,] -37.0  1.898910e-02
##  [28,] -36.5  2.390210e-02
##  [29,] -36.0  2.829508e-02
##  [30,] -35.5  3.241020e-02
##  [31,] -35.0  3.837023e-02
##  [32,] -34.5  4.083006e-02
##  [33,] -34.0  4.324264e-02
##  [34,] -33.5  4.853279e-02
##  [35,] -33.0  5.505340e-02
##  [36,] -32.5  6.305047e-02
##  [37,] -32.0  6.958255e-02
##  [38,] -31.5  7.596995e-02
##  [39,] -31.0  8.358055e-02
##  [40,] -30.5  8.644084e-02
##  [41,] -30.0  8.836266e-02
##  [42,] -29.5  9.001533e-02
##  [43,] -29.0  9.407348e-02
##  [44,] -28.5  9.624750e-02
##  [45,] -28.0  9.704347e-02
##  [46,] -27.5  9.732853e-02
##  [47,] -27.0  9.727674e-02
##  [48,] -26.5  9.754452e-02
##  [49,] -26.0  9.773804e-02
##  [50,] -25.5  9.698614e-02
##  [51,] -25.0  9.652744e-02
##  [52,] -24.5  9.724259e-02
##  [53,] -24.0  9.701219e-02
##  [54,] -23.5  9.663132e-02
##  [55,] -23.0  9.662974e-02
##  [56,] -22.5  9.691248e-02
##  [57,] -22.0  9.642700e-02
##  [58,] -21.5  9.684229e-02
##  [59,] -21.0  9.685330e-02
##  [60,] -20.5  9.640945e-02
##  [61,] -20.0  9.599602e-02
##  [62,] -19.5  9.597335e-02
```

```
##  [63,] -19.0  9.553266e-02
##  [64,] -18.5  9.507256e-02
##  [65,] -18.0  9.502212e-02
##  [66,] -17.5  9.492085e-02
##  [67,] -17.0  9.475259e-02
##  [68,] -16.5  9.489709e-02
##  [69,] -16.0  9.466500e-02
##  [70,] -15.5  9.443581e-02
##  [71,] -15.0  9.424732e-02
##  [72,] -14.5  9.371611e-02
##  [73,] -14.0  9.315346e-02
##  [74,] -13.5  9.268546e-02
##  [75,] -13.0  9.260104e-02
##  [76,] -12.5  9.253411e-02
##  [77,] -12.0  9.254757e-02
##  [78,] -11.5  9.238572e-02
##  [79,] -11.0  9.176371e-02
##  [80,] -10.5  9.120986e-02
##  [81,] -10.0  9.165274e-02
##  [82,]  -9.5  9.264033e-02
##  [83,]  -9.0  9.370697e-02
##  [84,]  -8.5  9.838869e-02
##  [85,]  -8.0  1.042550e-01
##  [86,]  -7.5  1.067526e-01
##  [87,]  -7.0  1.096532e-01
##  [88,]  -6.5  1.136059e-01
##  [89,]  -6.0  1.156980e-01
##  [90,]  -5.5  1.164271e-01
##  [91,]  -5.0  1.176093e-01
##  [92,]  -4.5  1.195222e-01
##  [93,]  -4.0  1.201184e-01
##  [94,]  -3.5  1.209429e-01
##  [95,]  -3.0  1.212061e-01
##  [96,]  -2.5  1.218690e-01
##  [97,]  -2.0  1.223226e-01
##  [98,]  -1.5  1.228413e-01
##  [99,]  -1.0  1.232243e-01
## [100,]  -0.5  1.234792e-01
## [101,]   0.0  1.212481e-01
```

Sensitivity analysis showing how the diversification rates compare

Weaker Prior 1

We first put a weaker prior on the magnitude of extinction. In this exercise we hypothesize that the mean survival was 25% (95% CI: 0.07-0.50).

```
speciationPriorMu <- 0.2
speciationPriorSigma <- 0.5
extinctionPriorMu <- 0.15
extinctionPriorSigma <- 0.5
speciationRatePriorMean <- log((speciationPriorMu^2)/sqrt(speciationPriorSigma^2+
                                                           speciationPriorMu^2))
speciationRatePriorStDev <- sqrt( log(1+speciationPriorSigma^2/(speciationPriorMu^2)))
```

```
extinctionRatePriorMean <- log((extinctionPriorMu^2)/sqrt(extinctionPriorSigma^2+
                                                  extinctionPriorMu^2))
extinctionRatePriorStDev <- sqrt( log(1+extinctionPriorSigma^2/(extinctionPriorMu^2)))

expectedSurvivalProbability <- 0.25
pMassExtinctionPriorShape2 <- 10
pMassExtinctionPriorShape1 <- - pMassExtinctionPriorShape2 *expectedSurvivalProbability /
  (expectedSurvivalProbability - 1)
curve(dbeta(x,shape1=pMassExtinctionPriorShape1,shape2=pMassExtinctionPriorShape2),
      n=1001, xlab='survival probability',ylab='density',las=1)
abline(v = qbeta(c(0.025,0.975),shape1=pMassExtinctionPriorShape1,
                 shape2=pMassExtinctionPriorShape2),lty=2)
```

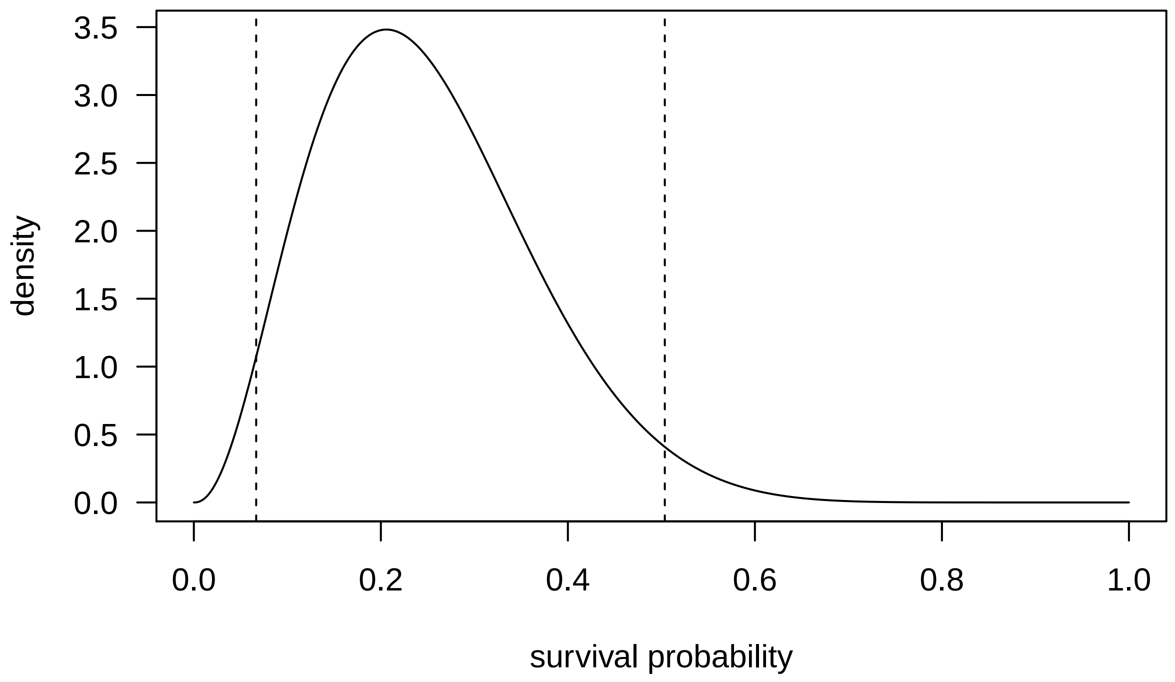


```
samplingFraction=0.74
numExpectedMassExtinctions=1
numExpectedRateChanges=1
set.seed(12345)
nnls<-nnls.tree(cophenetic(lemurs),lemurs,rooted=TRUE)
```

```
## [1] "RSS: 6.76176366918855e-08"
```

Now we run the model.

```
tess.analysis(nnls,
empiricalHyperPriors = FALSE,
initialSpeciationRate = speciationPriorMu,
speciationRatePriorMean = speciationRatePriorMean,
```

```
speciationRatePriorStDev = speciationRatePriorStDev,
initialExtinctionRate = extinctionPriorMu,
extinctionRatePriorMean = extinctionRatePriorMean,
extinctionRatePriorStDev = extinctionRatePriorStDev,
samplingProbability = samplingFraction,
numExpectedRateChanges = numExpectedRateChanges,
numExpectedMassExtinctions = numExpectedMassExtinctions,
pMassExtinctionPriorShape1 = pMassExtinctionPriorShape1,
pMassExtinctionPriorShape2 = pMassExtinctionPriorShape2,
MAX_ITERATIONS = 10e+06,
BURNIN = 2e+06,
dir = "tess_analysis_thinpriors_mu_lambda_weakextinction1")
```

```
output <- tess.process.output("tess_analysis_thinpriors_mu_lambda_weakextinction1",
numExpectedRateChanges = numExpectedRateChanges,
numExpectedMassExtinctions = numExpectedMassExtinctions)
tess.plot.singlechain.diagnostics(output,parameters = c("net-diversification rates"),las=2)
```

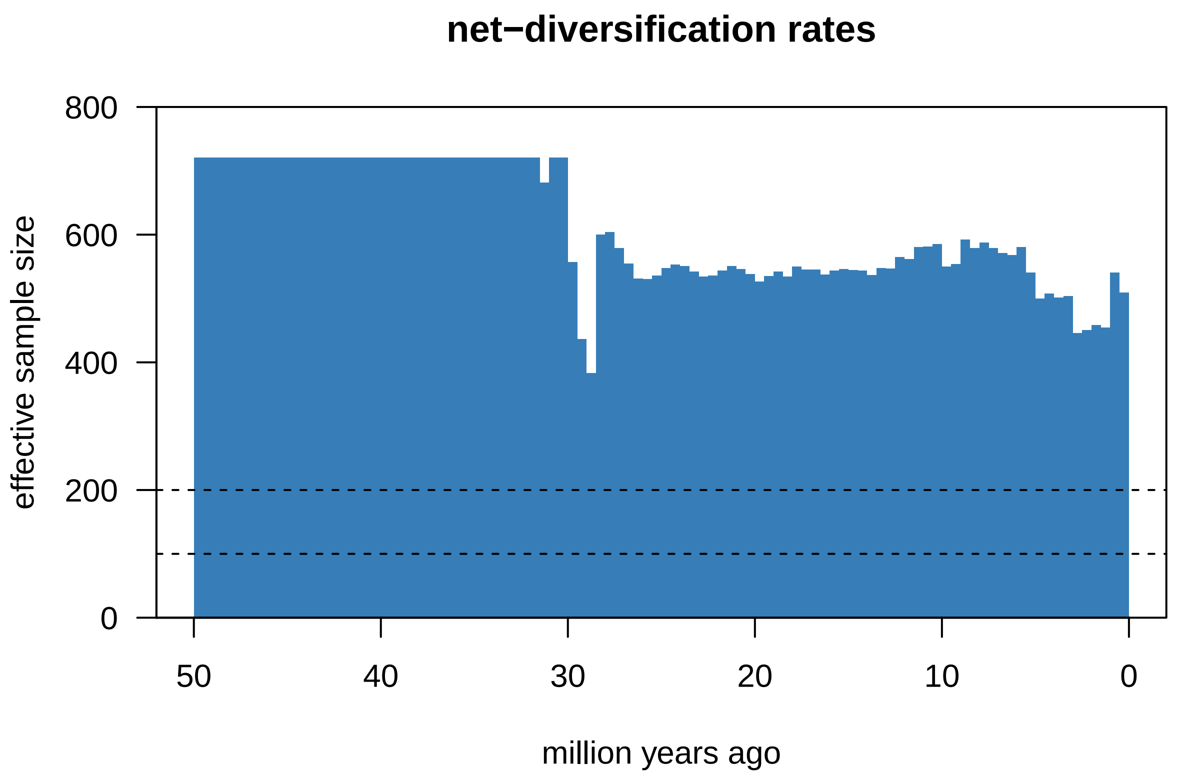

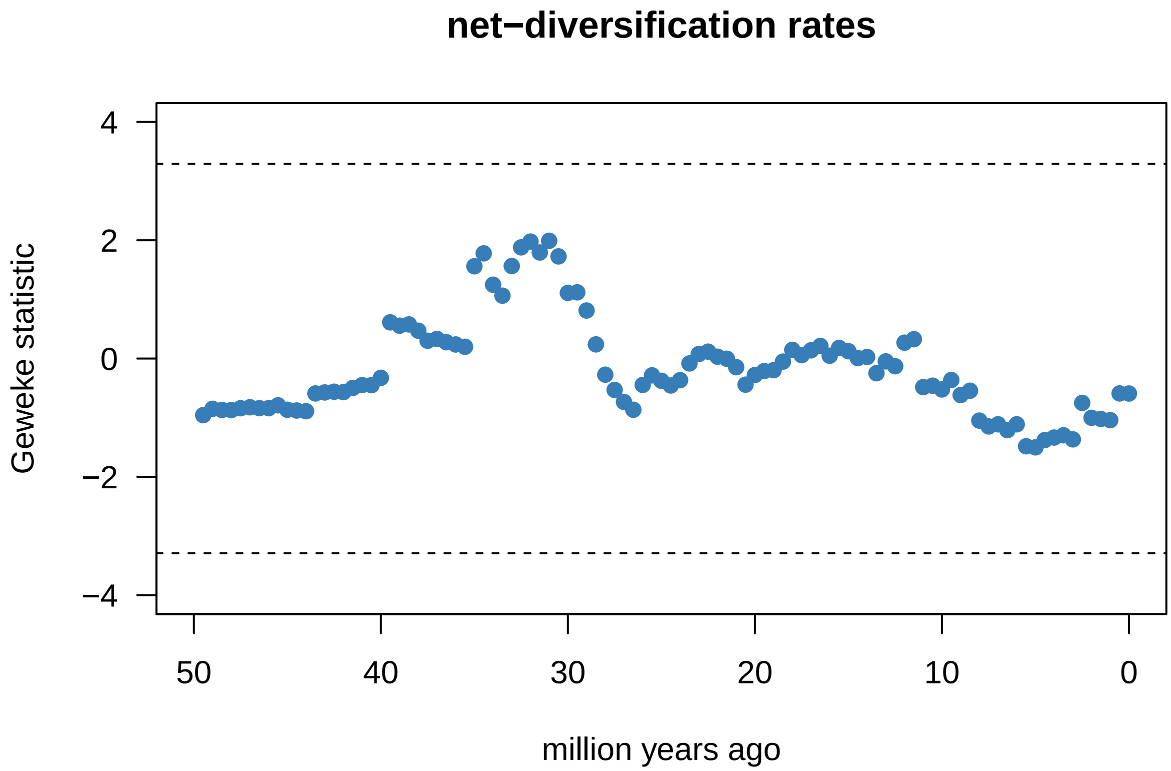


```r
tess.plot.output(output,fig.types =  c("net-diversification rates"),las=2)
```

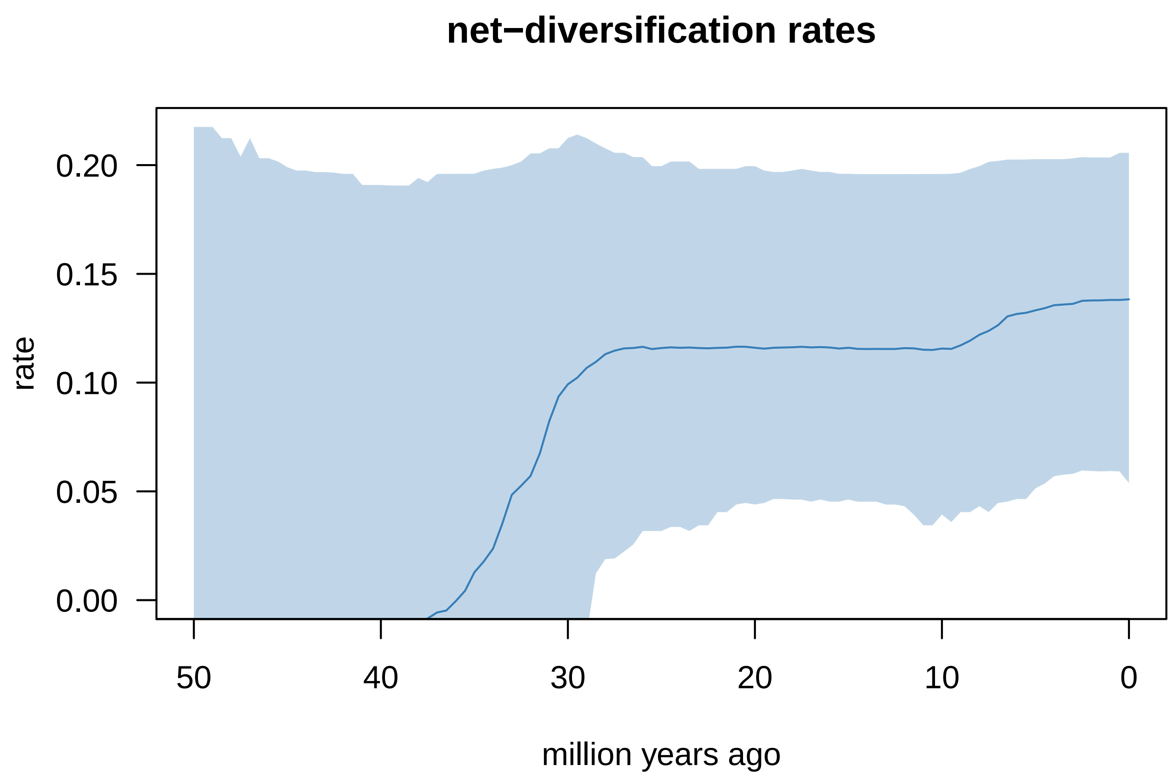

This looks similar to what the analysis with the strong prior indicates.

Weak Prior 2

Now we hypothesize that the magnitude of survival was 0.75 (95% CI: 0.48-0.94).

```
speciationPriorMu <- 0.2
speciationPriorSigma <- 0.5
extinctionPriorMu <- 0.15
extinctionPriorSigma <- 0.5
speciationRatePriorMean <- log((speciationPriorMu^2)/sqrt(speciationPriorSigma^2+
                                                           speciationPriorMu^2))
speciationRatePriorStDev <- sqrt( log(1+speciationPriorSigma^2/(speciationPriorMu^2)))
extinctionRatePriorMean <- log((extinctionPriorMu^2)/sqrt(extinctionPriorSigma^2+
                                                           extinctionPriorMu^2))
extinctionRatePriorStDev <- sqrt( log(1+extinctionPriorSigma^2/(extinctionPriorMu^2)))
expectedSurvivalProbability <- 0.75
pMassExtinctionPriorShape2 <- 3
pMassExtinctionPriorShape1 <- - pMassExtinctionPriorShape2 *
  expectedSurvivalProbability /(expectedSurvivalProbability - 1)
curve(dbeta(x,shape1=pMassExtinctionPriorShape1,shape2=pMassExtinctionPriorShape2),
      n=1001, xlab='survival probability',ylab='density',las=1)
abline(v = qbeta(c(0.025,0.975),shape1=pMassExtinctionPriorShape1,
                 shape2=pMassExtinctionPriorShape2),lty=2)
```

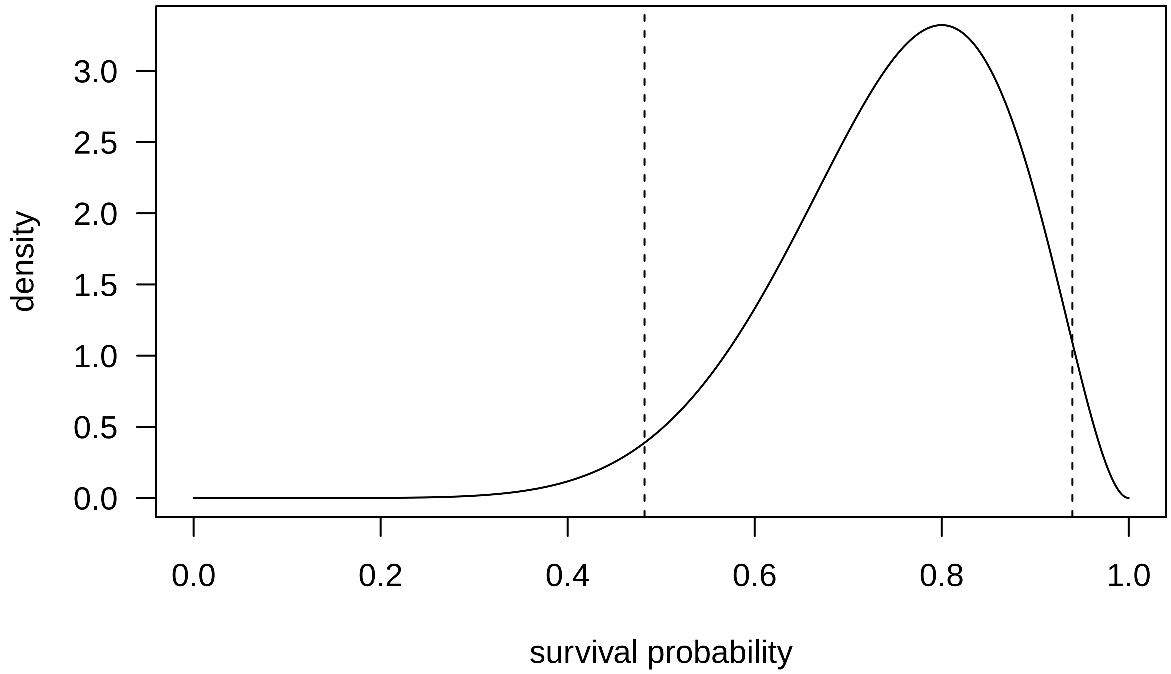


```
samplingFraction=0.74
numExpectedMassExtinctions=1
numExpectedRateChanges=1
set.seed(12345)
nnls<-nnls.tree(cophenetic(lemurs),lemurs,rooted=TRUE)
```

```
## [1] "RSS: 6.76176366918855e-08"
```

Now we run the analysis.

```
tess.analysis(nnls,
              empiricalHyperPriors = FALSE,
              initialSpeciationRate = speciationPriorMu,
              speciationRatePriorMean = speciationRatePriorMean,
              speciationRatePriorStDev = speciationRatePriorStDev,
              initialExtinctionRate = extinctionPriorMu,
              extinctionRatePriorMean = extinctionRatePriorMean,
              extinctionRatePriorStDev = extinctionRatePriorStDev,
              samplingProbability = samplingFraction,
              numExpectedRateChanges = numExpectedRateChanges,
              numExpectedMassExtinctions = numExpectedMassExtinctions,
              pMassExtinctionPriorShape1 = pMassExtinctionPriorShape1,
              pMassExtinctionPriorShape2 = pMassExtinctionPriorShape2,
              MAX_ITERATIONS = 10e+06, BURNIN = 2e+06,
              dir = "tess_analysis_thinpriors_mu_lambda_weakprior2")
```

```
output <- tess.process.output("tess_analysis_thinpriors_mu_lambda_weakprior2",
numExpectedRateChanges = numExpectedRateChanges,
```

```
numExpectedMassExtinctions = numExpectedMassExtinctions)
tess.plot.singlechain.diagnostics(output,parameters = c("net-diversification rates"),las=2)
```

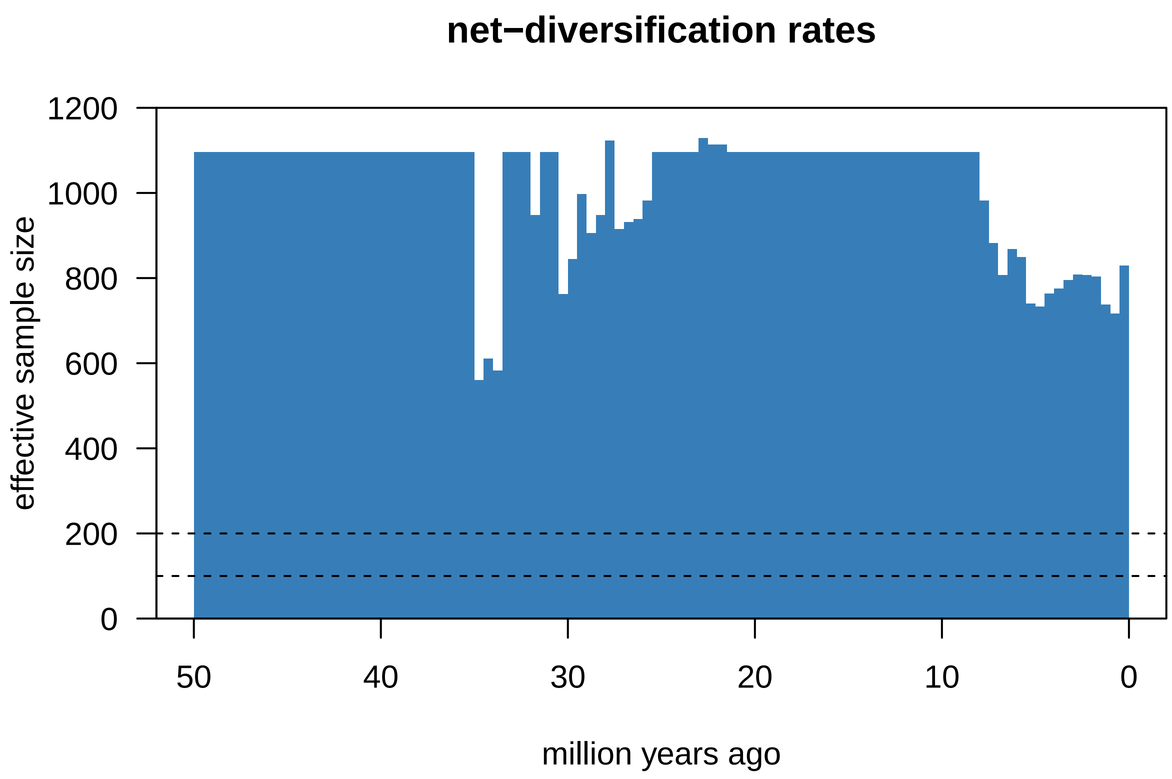

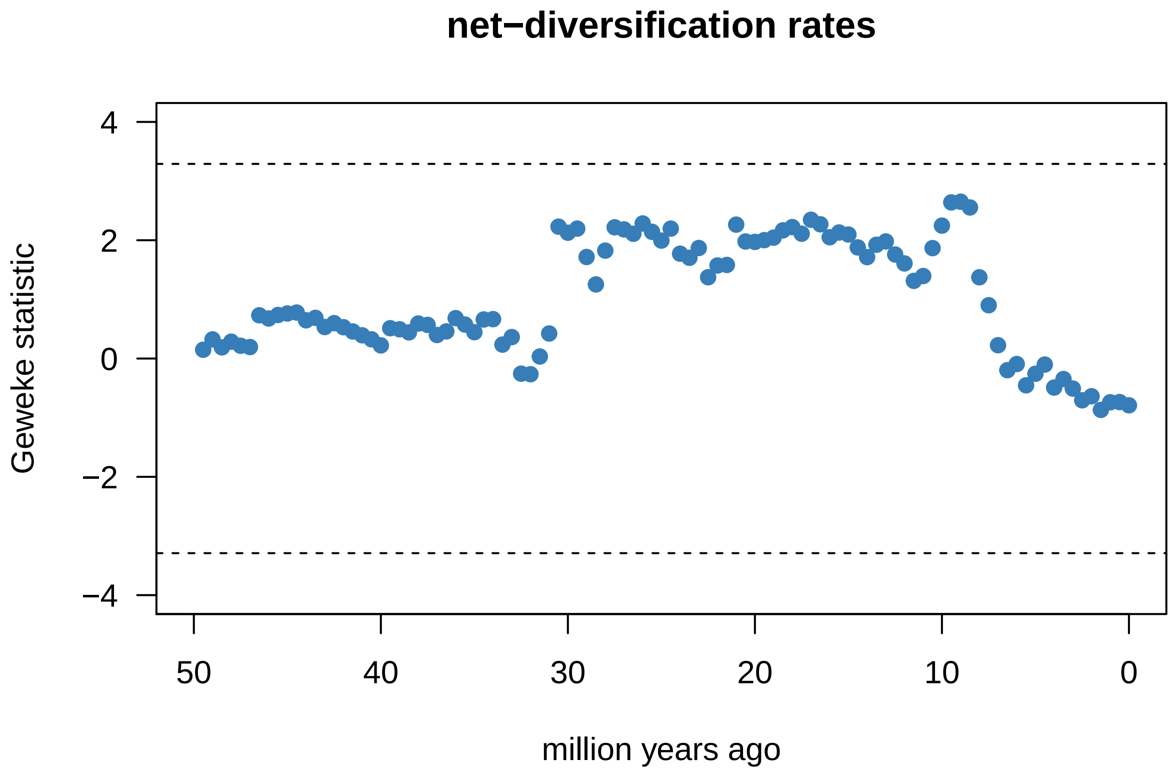


```
tess.plot.output(output,fig.types = c("net-diversification rates"),las=2)
```

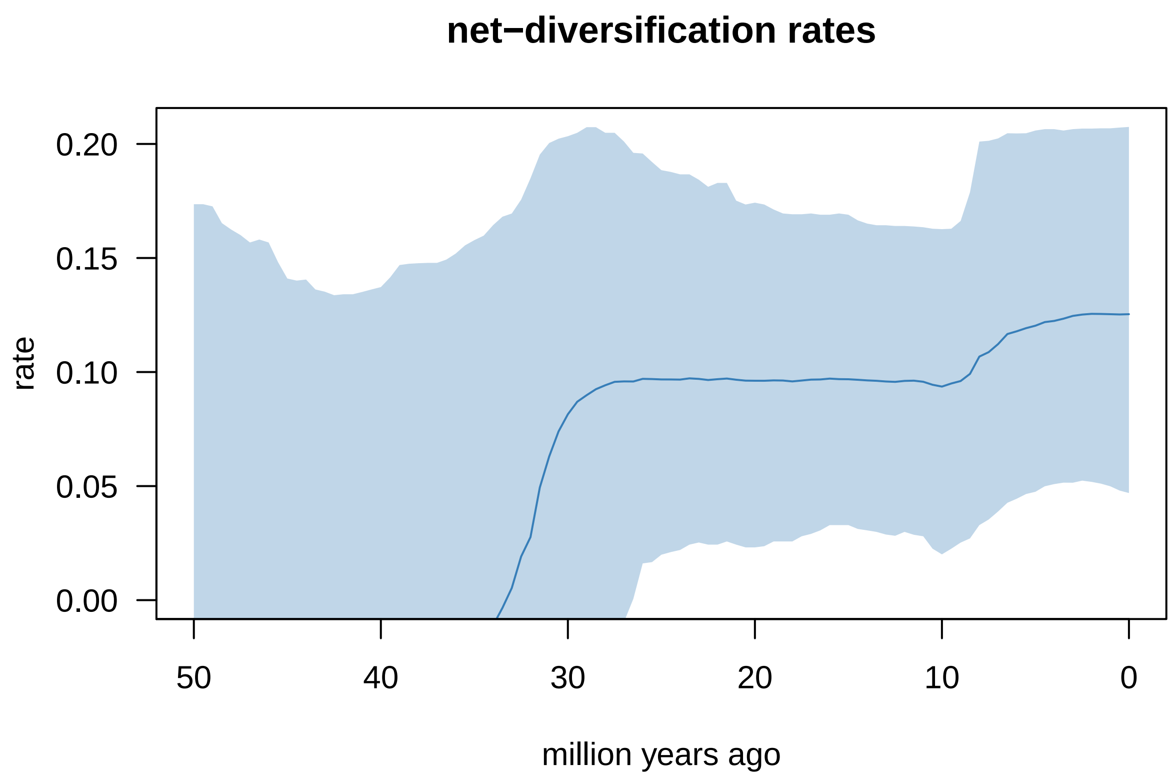

This is qualitatively similar.

2.5. Plot All Models of Diversification Rate Together

```
lthick=3
plot.new()
##
# BAMM
##
                xMin <- -maxTime
                xMax <- 0
                yMin <- -0.8
                yMax <- 0.2
            plot.window(xlim = c(xMin, xMax), ylim = c(yMin,
                yMax))
            xticks=yticks=NULL
               axis(at = c(round(1.2 * xMin), axTicks(1)), cex.axis = 1,
                  side = 1)
                axis(at = round(c(-1, axTicks(2)),2), cex.axis = 1,
                  las = 1, side = 2)
                 axis.labels=TRUE
                xline = 3
                yline = 3
            if (axis.labels) {
                mtext(side = 1, text = "Time",
```

```
                  line = xline)
                mtext(side = 2, text = "Diversification Rate", line = yline)
            }
                axis.labels=FALSE
                 intervalCol='gray80'
                opacity=1
        if (!is.null(intervals)) {
            for (i in 1:length(poly)) {
                polygon(x = -poly[[i]][, 1], y = poly[[i]][, 2],
                  col = transparentColor(intervalCol, opacity),
                  border = NA)
            }
        }
        lines(x = -rmat$time, y = avg, col = "black", lwd=lthick)
        abline(h=0, col="black", lty=2)
##
# RPANDA
##
lines(-t, r(results$lemurs.res$bcstdcst, t), col="blue", lwd=lthick)
lines(-t, r(results$lemurs.res$bcstdvar, t),  col="red", lwd=lthick)

##
# Treepar
##
lines(diversification~c(time_mya), data=treepar.df, col="purple", lwd=lthick)

####
# TESS
###
lines(x = (plotAt-100)/2, y = c(meanThisOutput[1], meanThisOutput),
      col = col[type],  lwd=3)
polygon(x = c(c(0:ncol(quantilesThisOutput)-100)/2,
              c(c(ncol(quantilesThisOutput):0)-100)/2),
  y = c(c(quantilesThisOutput[1, 1], quantilesThisOutput[1, ]),
        rev(c(quantilesThisOutput[2, 1], quantilesThisOutput[2,]))),
  border = NA, col = paste(col[type], col.alpha=50,
  sep = ""))

###
# Legend and indicator of 34 Ma
###
legend(-25,-0.025, c("BAMM","RPANDA: ScstEcst","RPANDA: ScstEvar","TreePar", "TESS"),
       lty=c(1,1,1,1,1),lwd=c(3,3,3,3,3),
       col=c("black","blue","red","purple",col[type]))
abline(h = 0.2, v = -34, col = "lightgray", lty = 3)
```

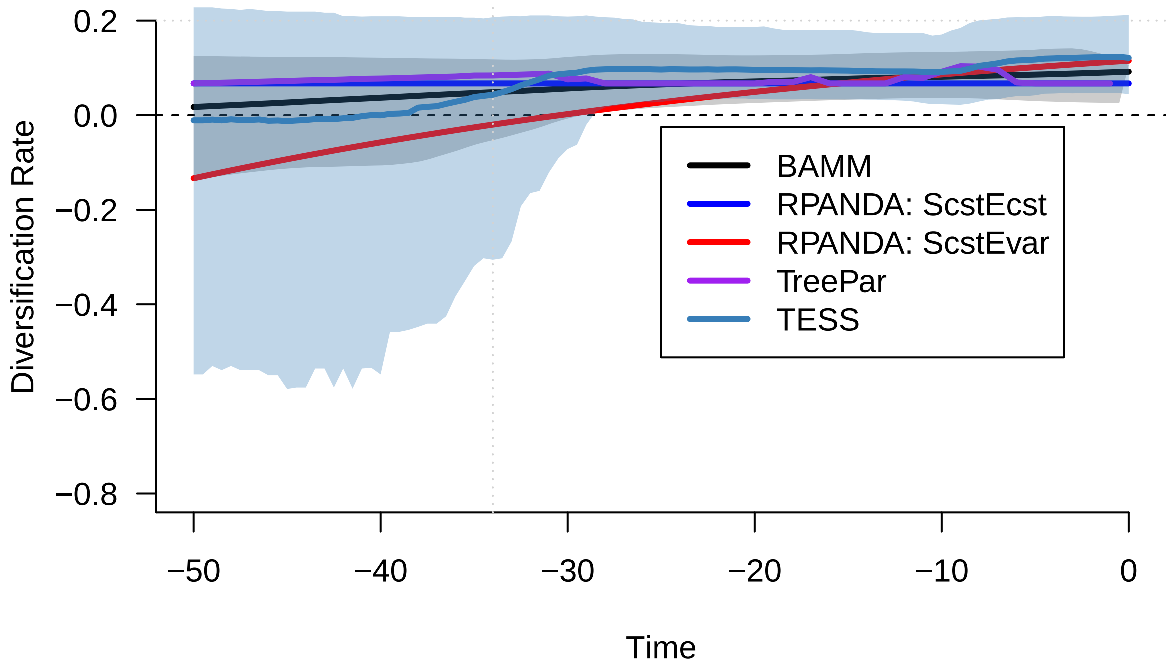

3. Test for Mass Extinction

3.1. RPANDA

With RPANDA, we construct a series of candidate “environmental variables” that we consider as predictors of extinction rate. Each variable is a flat line with a strong pulse at a different year. The variable is constructed by the probability distribution function (PDF) of a Normal distribution with variance equal to 0.25 and mean equal to the year in question. For example, the variable that corresponds to a pulsed extinction at 49 Ma is the PDF of a Normal distribution with mean 49 and variance 0.25 over the domain 0 to 50, and the variable that corresponds to a pulsed extinction at 48 Ma is the PDF of a Normal distribution with mean 48 and variance 0.25 over the domain 0 to 50 and so forth. We also construct a null model that has a flat line of 0 over the domain of 50 to 0 as a predictor of extinction rate.

```
killemall_ones <- data.frame(
  "NAge"=-seq(from=max(branching.times(lemurs))+2, to=0, length.out = 300),
  "Age"=seq(from=max(branching.times(lemurs))+2, to=0, length.out = 300))
for(i in 50:0){
  new.add=data.frame(
    "o"=c(dnorm(seq(from=max(branching.times(lemurs))+2, to=0, length.out = 300),
                mean = i, sd=0.5))
  )
  names(new.add) <- paste0("Ex_at_",i)
  killemall_ones <- cbind.data.frame(killemall_ones, new.add)
}
```

```
killemall_ones$Null <- 0
```

For example, the pulsed variable that posits an extinction at 34 Ma looks as below.

```
plot(Ex_at_34~NAge, data=killemall_ones, type="l", xlab="Years Ma")
```

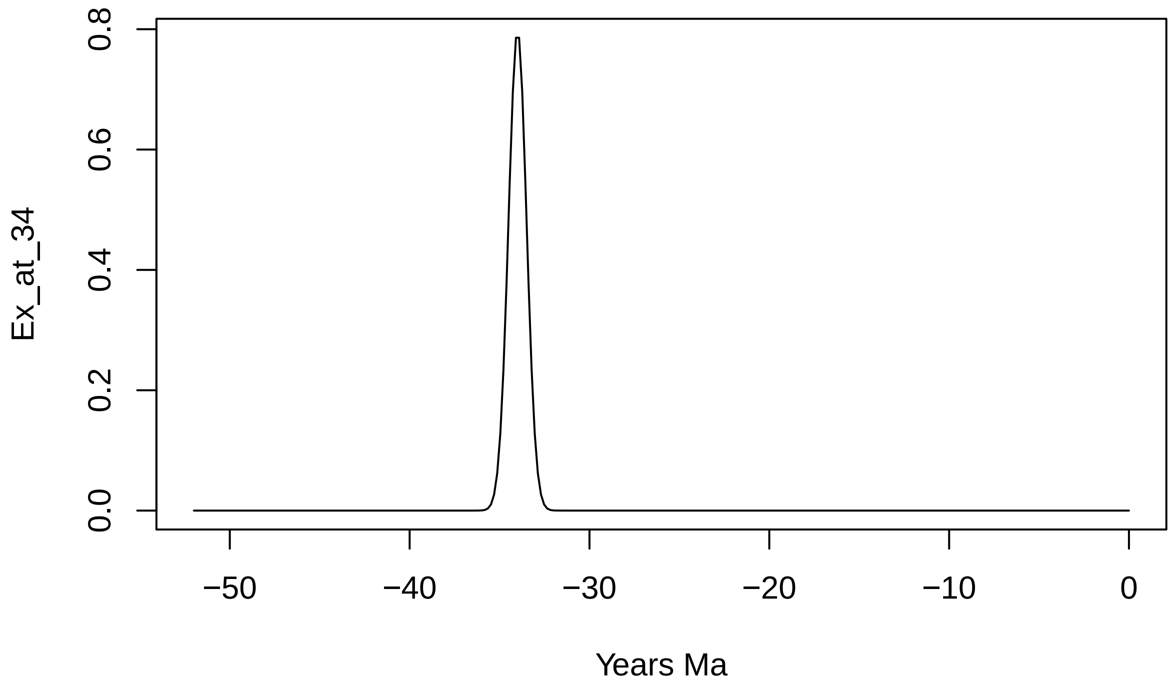

Then we fit 50 models each with the pulse variable as a predictor of extinction rate. We employ the same sampling fraction of incomplete taxon sample as above. Moreover we consider only exponential effects of the environmental pulse variable on the extinction rate as we assume $f_\mu(t, x, y) = y_1 * e^{y_2 * x}$. Herein, the extinction rate function over time is a function of two parameters y_1 and y_2 which are estimated from the data and the pulse variable x. The function must include the variable t to estimate the diversification rate over time which is the speciation rate - extinction rate. For each model we extract the AICc and likelihood. From the AICc we calculate the relative likelihood and Akaike Weight.

```
f=0.7417
f.lamb<-function(t,x,y){y[1]} #constant speciation rate,
f.mu.exp<-function(t,x,y){y[1]*exp(y[2]*x)}
f.mu.lin<-function(t,x,y){y[1]+(y[2]*x)}
lamb_par_init<-c(0.4)
mu_par_init<-c(0.1,0.05)
tot_time <- max(branching.times(lemurs))
AICcs <- c()
LHs <- c()
AICcs_lin <- c()
LHs_lin <- c()
```

```
for(i in 1:52){
```

```
env_data <- killemall_ones[nrow(killemall_ones):1,c(2,c(2+i))] #make sure first row is time zero
res.exp<-fit_env(lemurs, env_data,tot_time, f.lamb,f.mu.exp,
              lamb_par_init,mu_par_init,f=f,dt=1e-3)
res.lin<-fit_env(lemurs, env_data,tot_time, f.lamb,f.mu.lin,
              lamb_par_init,mu_par_init,f=f,dt=1e-3)
AICcs[i] <- res.exp$aicc
LHs[i] <- res.exp$LH

AICcs_lin[i] <- res.lin$aicc
LHs_lin[i] <- res.lin$LH

}
res=data.frame("AICc"=AICcs,
               "LHs"=LHs,
               "mod"=names(killemall_ones)[3:54])
res_lin=data.frame("AICc"=AICcs_lin,
               "LHs"=LHs_lin,
               "mod"=names(killemall_ones)[3:54])
```

Now we show the Akaike weight profile against the null model.

```
res$dAICc <- res$AICc-min(res$AICc)
res$RelativeLikelihood<-exp(-0.5*res$dAICc)
res$AkaikeWeights<-res$RelativeLikelihood/sum(res$RelativeLikelihood)
#Akaike Weights
plot.new()
plot.window(xlim = c(-50,0), ylim = c(0,0.2))
points(AkaikeWeights~year_of_ex, data=res[1:c(nrow(res)-1),], pch=19, col="#4DAF4A",
     ylim=c(0,0.2))
axis(at = c(round(1.2 * 50), axTicks(1)), side = 1)
axis(at = round(c(-1, axTicks(2)),2), las = 1, side = 2)
mtext(side = 1, text = "Time",line = 3)
mtext(side = 2, text ="Akaike Weights", line = 3)
```

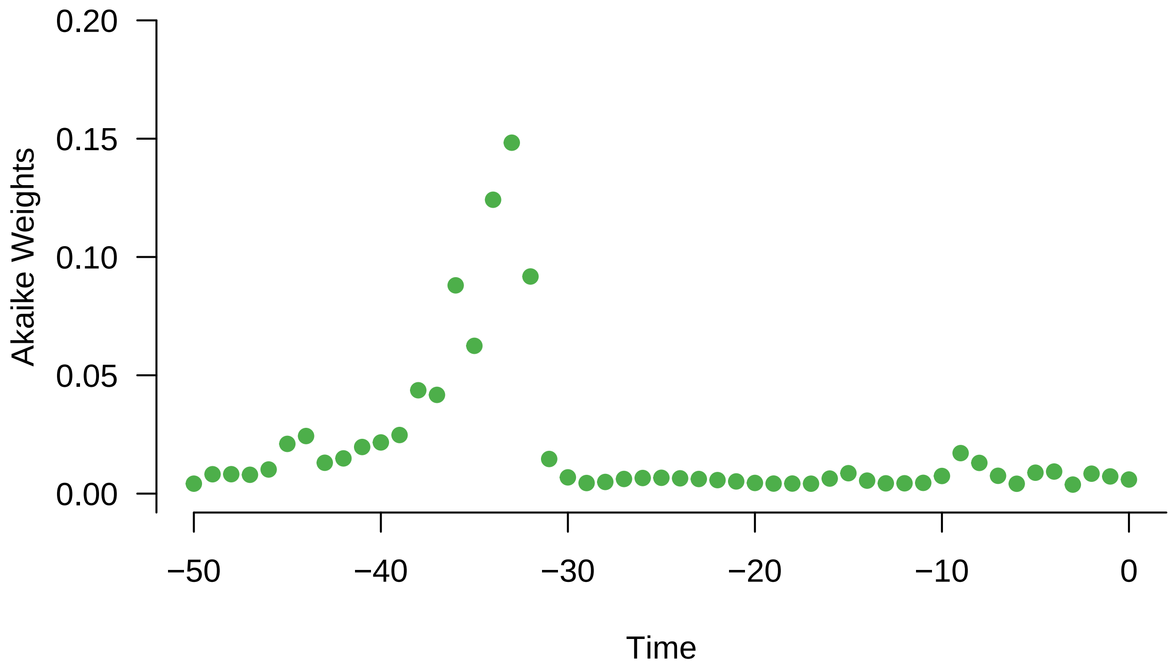

Now show the raw weights and AICc scores.

```
res
```

```
##        AICc     dAICc year_of_ex RelativeLikelihood AkaikeWeights
## 1  541.4425 7.1263667        -50         0.02834844   0.004204192
## 2  540.1108 5.7946763        -49         0.05516988   0.008181925
## 3  540.1058 5.7897152        -48         0.05530690   0.008202246
## 4  540.1660 5.8498811        -47         0.05366788   0.007959173
## 5  539.6714 5.3552531        -46         0.06872608   0.010192366
## 6  538.2235 3.9073727        -45         0.14175057   0.021022205
## 7  537.9311 3.6150274        -44         0.16406154   0.024331015
## 8  539.1796 4.8634868        -43         0.08788348   0.013033490
## 9  538.9146 4.5984762        -42         0.10033526   0.014880140
## 10 538.3530 4.0368375        -41         0.13286539   0.019704496
## 11 538.1671 3.8509450        -40         0.14580684   0.021623768
## 12 537.8963 3.5801549        -39         0.16694724   0.024758977
## 13 536.7608 2.4447241        -38         0.29453364   0.043680576
## 14 536.8525 2.5363531        -37         0.28134417   0.041724522
## 15 535.3598 1.0436406        -36         0.59343933   0.088009546
## 16 536.0458 1.7296499        -35         0.42112527   0.062454648
## 17 534.6710 0.3548612        -34         0.83741911   0.124192773
## 18 534.3161 0.0000000        -33         1.00000000   0.148304202
## 19 535.2763 0.9601513        -32         0.61873658   0.091761235
## 20 538.9482 4.6320550        -31         0.09866475   0.014632398
## 21 540.4611 6.1449581        -30         0.04630622   0.006867406
## 22 541.3157 6.9996136        -29         0.03020322   0.004479264
## 23 541.1253 6.8091650        -28         0.03322069   0.004926768
```

```
## 24 540.6701 6.3539971        -27          0.04171066   0.006185866
## 25 540.5424 6.2262980        -26          0.04446073   0.006593713
## 26 540.5152 6.1991245        -25          0.04506893   0.006683911
## 27 540.5823 6.2662204        -24          0.04358204   0.006463399
## 28 540.6742 6.3580999        -23          0.04162518   0.006173189
## 29 540.8310 6.5148448        -22          0.03848748   0.005707854
## 30 541.0379 6.7217561        -21          0.03470477   0.005146864
## 31 541.3045 6.9883355        -20          0.03037402   0.004504594
## 32 541.4291 7.1129677        -19          0.02853900   0.004232453
## 33 541.4230 7.1068552        -18          0.02862635   0.004245408
## 34 541.4460 7.1298476        -17          0.02829914   0.004196882
## 35 540.6141 6.2979535        -16          0.04289600   0.006361657
## 36 540.0055 5.6894062        -15          0.05815153   0.008624116
## 37 540.9110 6.5948660        -14          0.03697797   0.005483988
## 38 541.3787 7.0625314        -13          0.02926785   0.004340545
## 39 541.3692 7.0530463        -12          0.02940698   0.004361179
## 40 541.2864 6.9703251        -11          0.03064878   0.004545342
## 41 540.2985 5.9823328        -10          0.05022882   0.007449144
## 42 538.6348 4.3186407         -9          0.11540353   0.017114828
## 43 539.1878 4.8716783         -8          0.08752427   0.012980217
## 44 540.2776 5.9615113         -7          0.05075447   0.007527101
## 45 541.4601 7.1439974         -6          0.02809963   0.004167294
## 46 539.9576 5.6415128         -5          0.05956087   0.008833128
## 47 539.8507 5.5345318         -4          0.06283356   0.009318481
## 48 541.6377 7.3216269         -3          0.02571159   0.003813137
## 49 540.0544 5.7382479         -2          0.05674862   0.008416059
## 50 540.3515 6.0353310         -1          0.04891528   0.007254341
## 51 540.7491 6.4330082          0          0.04009498   0.005946254
## 52 541.4437 7.1275412          0          0.02833180   0.004201724
```

We see evidence for the models that posit an extinction happening near 36 to 32 Ma.

Using the Akaike Weights above the best model with the extinction at 34 Ma is `0.148304202/0.004201724= 35.3` times more likely than the null model. And the probability that the model with the extinction at 34 Ma is preferred over the null model is equal to `0.148304202/(0.148304202+0.004201724) = 0.97`.

Likewise, the models that posit an extinction occurring between 36 and 32 Ma are collectively 122.5 more likely than the null model (`sum(res$AkaikeWeights[15:19])/res$AkaikeWeights[nrow(res)]`). The probability that the models positing extinctions to occur between 36 and 32 are preferred over the null model is 0.991903 (`sum(res$AkaikeWeights[15:19])/sum(sum(res$AkaikeWeights[15:19]), res$AkaikeWeights[nrow(res)])`).

We can also consider linear effects of the pulse variables. We can show the Akaike weight profile against the null model, using the linear effect.

```
res_lin$dAICc <- res_lin$AICc-min(res_lin$AICc)
res_lin$RelativeLikelihood<-exp(-0.5*res_lin$dAICc)
res_lin$AkaikeWeights<-res_lin$RelativeLikelihood/sum(res_lin$RelativeLikelihood)
res_lin$year_of_ex<--50:1
#Akaike Weights
plot.new()
plot.window(xlim = c(-50,0), ylim = c(0,0.2))
points(AkaikeWeights~year_of_ex, data=res_lin[1:c(nrow(res_lin)-1),], pch=19, col="#4DAF4A",
     ylim=c(0,0.2))
axis(at = c(round(1.2 * 50), axTicks(1)), side = 1)
axis(at = round(c(-1, axTicks(2)),2), las = 1, side = 2)
```

```r
mtext(side = 1, text = "Time",line = 3)
mtext(side = 2, text ="Akaike Weights", line = 3)
title("Linear effect")
```

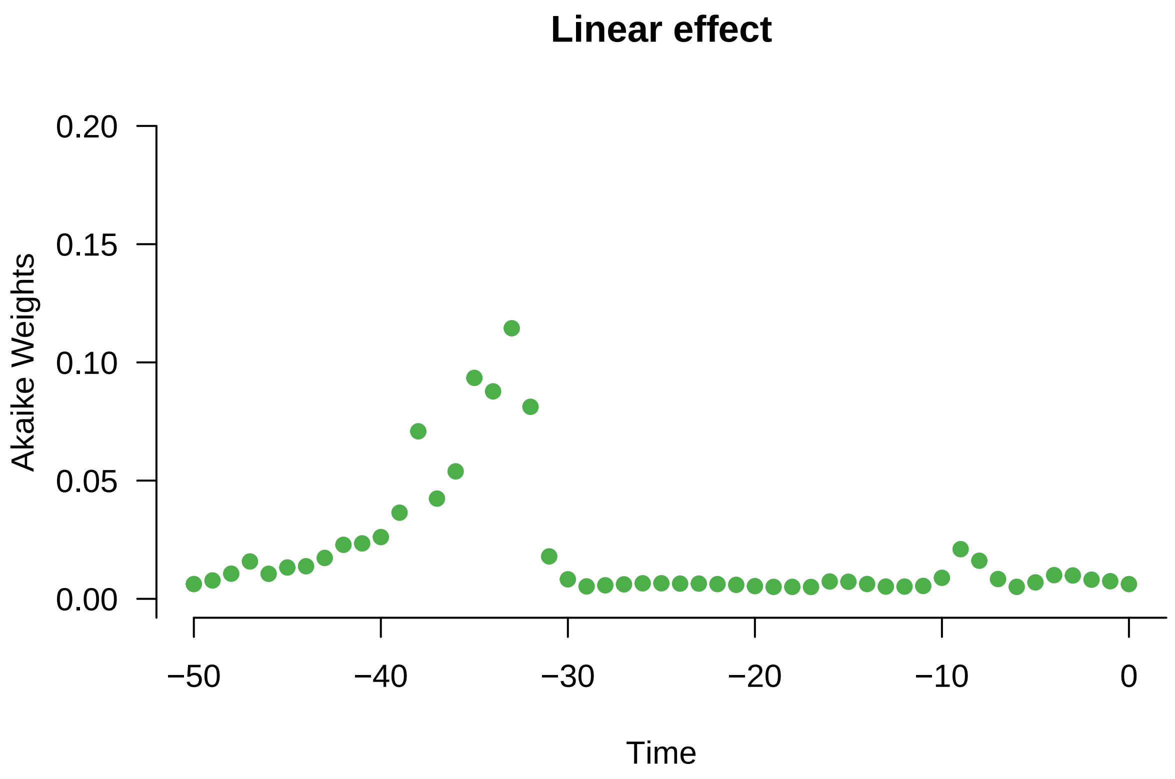

Now show the raw weights and AICc scores.

```r
res_lin
```

```
##        AICc       LHs       mod     dAICc RelativeLikelihood AkaikeWeights
## 1  541.0020 -267.3598 Ex_at_50 5.8167713         0.05456374   0.006244815
## 2  540.5659 -267.1418 Ex_at_49 5.3806585         0.06785859   0.007766410
## 3  539.9369 -266.8272 Ex_at_48 4.7516019         0.09294002   0.010636977
## 4  539.1450 -266.4313 Ex_at_47 3.9596993         0.13809000   0.015804388
## 5  539.9466 -266.8321 Ex_at_46 4.7613320         0.09248896   0.010585353
## 6  539.4969 -266.6073 Ex_at_45 4.3116262         0.11580899   0.013254328
## 7  539.4186 -266.5681 Ex_at_44 4.2333051         0.12043410   0.013783672
## 8  538.9645 -266.3411 Ex_at_43 3.7792384         0.15112935   0.017296740
## 9  538.4075 -266.0626 Ex_at_42 3.2222466         0.19966321   0.022851436
## 10 538.3554 -266.0365 Ex_at_41 3.1701580         0.20493160   0.023454403
## 11 538.1402 -265.9289 Ex_at_40 2.9549879         0.22820887   0.026118485
## 12 537.4737 -265.5957 Ex_at_39 2.2884409         0.31847209   0.036449102
## 13 536.1444 -264.9310 Ex_at_38 0.9591012         0.61906154   0.070851537
## 14 537.1717 -265.4447 Ex_at_37 1.9864821         0.37037434   0.042389309
## 15 536.6909 -265.2043 Ex_at_36 1.5056813         0.47102663   0.053908955
## 16 535.5908 -264.6542 Ex_at_35 0.4055128         0.81647712   0.093445732
## 17 535.7169 -264.7173 Ex_at_34 0.5316311         0.76658050   0.087735068
## 18 535.1853 -264.4514 Ex_at_33 0.0000000         1.00000000   0.114449909
```

```
## 19 535.8711 -264.7944 Ex_at_32 0.6858988         0.70967412   0.081222138
## 20 538.8899 -266.3038 Ex_at_31 3.7046672         0.15687067   0.017953834
## 21 540.4432 -267.0804 Ex_at_30 5.2579149         0.07215365   0.008257978
## 22 541.3437 -267.5307 Ex_at_29 6.1584224         0.04599552   0.005264183
## 23 541.1738 -267.4457 Ex_at_28 5.9885079         0.05007397   0.005730961
## 24 541.0384 -267.3780 Ex_at_27 5.8531434         0.05358041   0.006132273
## 25 540.8994 -267.3085 Ex_at_26 5.7141820         0.05743560   0.006573499
## 26 540.8962 -267.3069 Ex_at_25 5.7109877         0.05752740   0.006584006
## 27 540.9432 -267.3304 Ex_at_24 5.7579517         0.05619228   0.006431202
## 28 540.9366 -267.3271 Ex_at_23 5.7513337         0.05637853   0.006452518
## 29 541.0047 -267.3612 Ex_at_22 5.8194301         0.05449125   0.006236519
## 30 541.1095 -267.4136 Ex_at_21 5.9242885         0.05170792   0.005917967
## 31 541.3022 -267.5099 Ex_at_20 6.1169249         0.04695984   0.005374550
## 32 541.4290 -267.5733 Ex_at_19 6.2437614         0.04407420   0.005044288
## 33 541.4231 -267.5704 Ex_at_18 6.2378437         0.04420480   0.005059236
## 34 541.4428 -267.5802 Ex_at_17 6.2575799         0.04377073   0.005009556
## 35 540.6779 -267.1978 Ex_at_16 5.4926548         0.06416307   0.007343458
## 36 540.7143 -267.2160 Ex_at_15 5.5290163         0.06300708   0.007211155
## 37 540.9932 -267.3554 Ex_at_14 5.8079428         0.05480513   0.006272443
## 38 541.3747 -267.5462 Ex_at_13 6.1894434         0.04528761   0.005183163
## 39 541.3735 -267.5456 Ex_at_12 6.1882257         0.04531520   0.005186320
## 40 541.2710 -267.4943 Ex_at_11 6.0857667         0.04769716   0.005458936
## 41 540.2972 -267.0074 Ex_at_10 5.1119151         0.07761787   0.008883358
## 42 538.5758 -266.1467  Ex_at_9 3.3905401         0.18354966   0.021007241
## 43 539.1066 -266.4121  Ex_at_8 3.9213708         0.14076191   0.016110188
## 44 540.4172 -267.0674  Ex_at_7 5.2319679         0.07309583   0.008365811
## 45 541.4177 -267.5677  Ex_at_6 6.2324515         0.04432414   0.005072894
## 46 540.7859 -267.2518  Ex_at_5 5.6006481         0.06079036   0.006957451
## 47 540.0561 -266.8869  Ex_at_4 4.8708235         0.08756169   0.010021427
## 48 540.0863 -266.9020  Ex_at_3 4.9010356         0.08624892   0.009871181
## 49 540.4846 -267.1011  Ex_at_2 5.2993254         0.07067505   0.008088753
## 50 540.6473 -267.1825  Ex_at_1 5.4620537         0.06515235   0.007456681
## 51 541.0065 -267.3621  Ex_at_0 5.8212838         0.05444077   0.006230742
## 52 541.4437 -267.5807     Null 6.2584128         0.04375251   0.005007470
##    year_of_ex
## 1         -50
## 2         -49
## 3         -48
## 4         -47
## 5         -46
## 6         -45
## 7         -44
## 8         -43
## 9         -42
## 10        -41
## 11        -40
## 12        -39
## 13        -38
## 14        -37
## 15        -36
## 16        -35
## 17        -34
## 18        -33
## 19        -32
```

```
## 20         -31
## 21         -30
## 22         -29
## 23         -28
## 24         -27
## 25         -26
## 26         -25
## 27         -24
## 28         -23
## 29         -22
## 30         -21
## 31         -20
## 32         -19
## 33         -18
## 34         -17
## 35         -16
## 36         -15
## 37         -14
## 38         -13
## 39         -12
## 40         -11
## 41         -10
## 42          -9
## 43          -8
## 44          -7
## 45          -6
## 46          -5
## 47          -4
## 48          -3
## 49          -2
## 50          -1
## 51           0
## 52           1
```

We see that the inference from linear and exponential effects is qualitatively similar. We continue with the exponential effects.

Now to make it more clear what the model that posits the pulsed environmental variable with a peak at 34 Ma is actually predicting, we can make use of the `plot_fit_env()` function as in Morlon et al. 2016 (Fig. 6). This will clarify what the model is actually saying, and it shall become clear that while this model fares better than other models (as shown above), it is unlikely to be right. So, as they say, all models are wrong, but some are useful.

Because the looping process above overwrote the models, we must re-create the best model with a pulse at 34 being an exponential predictor of the extinction rate.

```
env_data_34 <- killemall_ones[nrow(killemall_ones):1,c("Age","Ex_at_34")]
res.exp_34<-fit_env(lemurs, env_data_34,tot_time, f.lamb,f.mu.exp,
              lamb_par_init,mu_par_init,f=f,dt=1e-3)
#plot_fit_env(res.exp_34, env_data_34, 55)
#minor changes necessary because speciation rate is constant
t <- seq(0, 55, length.out = 100)

    plot(-t, rep(res.exp_34$f.lamb(t), length(t)), type = "l", xlab = "time",
        ylab = "speciation rate", main = "Fitted speciation rate")
```

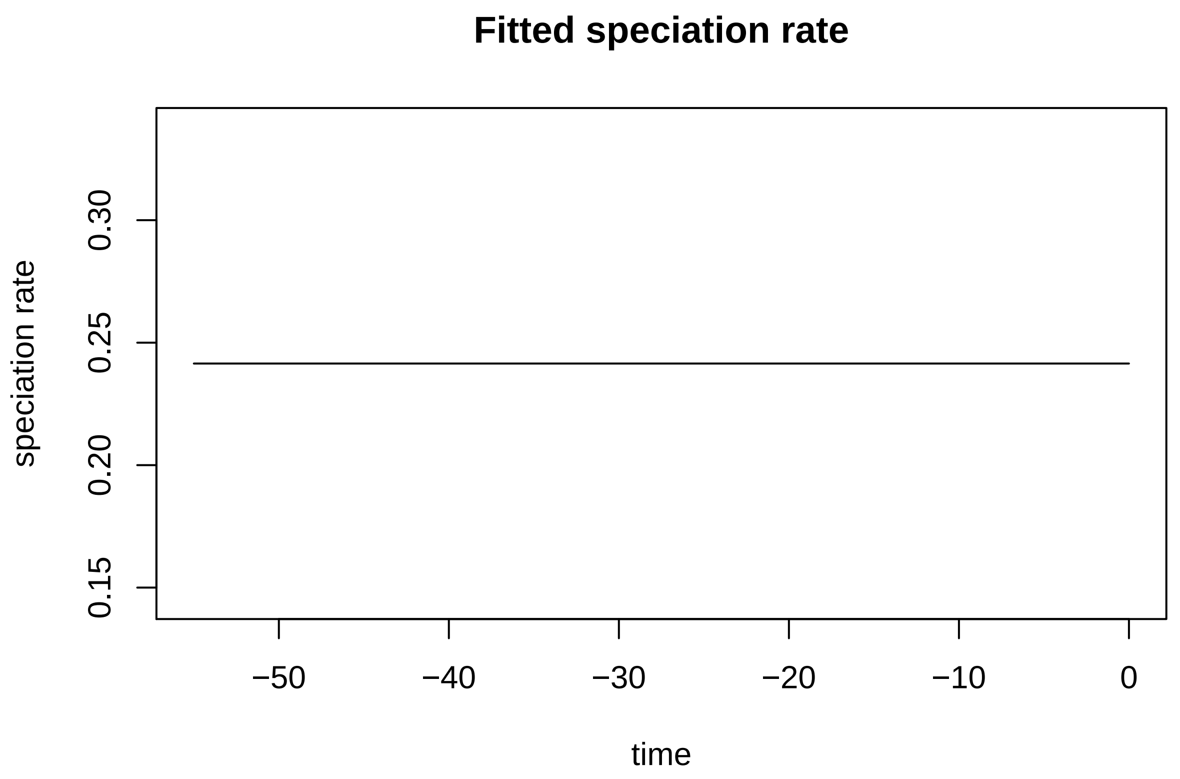


```
df <- smooth.spline(env_data_34[, 1], env_data_34[, 2])$df
spline_result <- pspline::sm.spline(env_data_34[, 1], env_data_34[, 2],
    df = df)
env_func <- function(t) {
    predict(spline_result, t)
}

plot(env_func(t), rep(res.exp_34$f.lamb(t), length(t)), type = "l", xlab = "Environmental data",
    ylab = "speciation rate", main = "Fitted speciation rate")
```

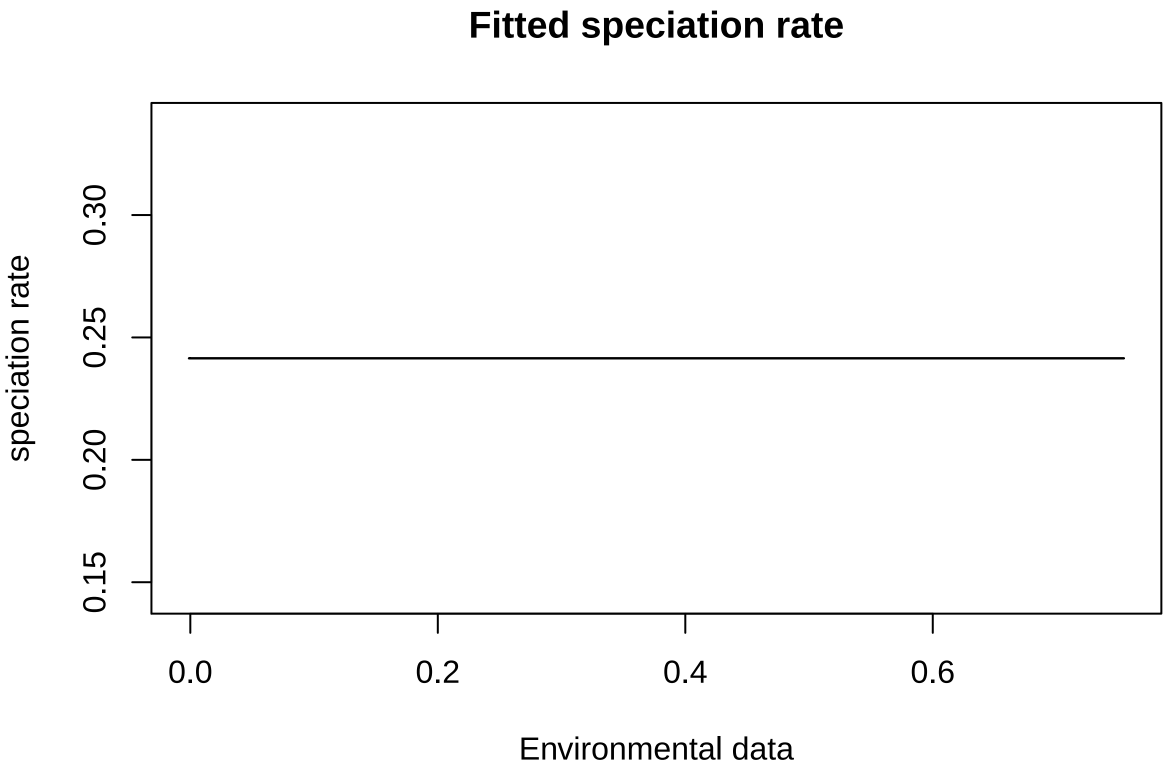


```
#    if ("f.mu" %in% attributes(fit.env)$names) {

        plot(-t, res.exp_34$f.mu(t), type = "l", xlab = "time",
            ylab = "extinction rate", main = "Fitted extinction rate")
```

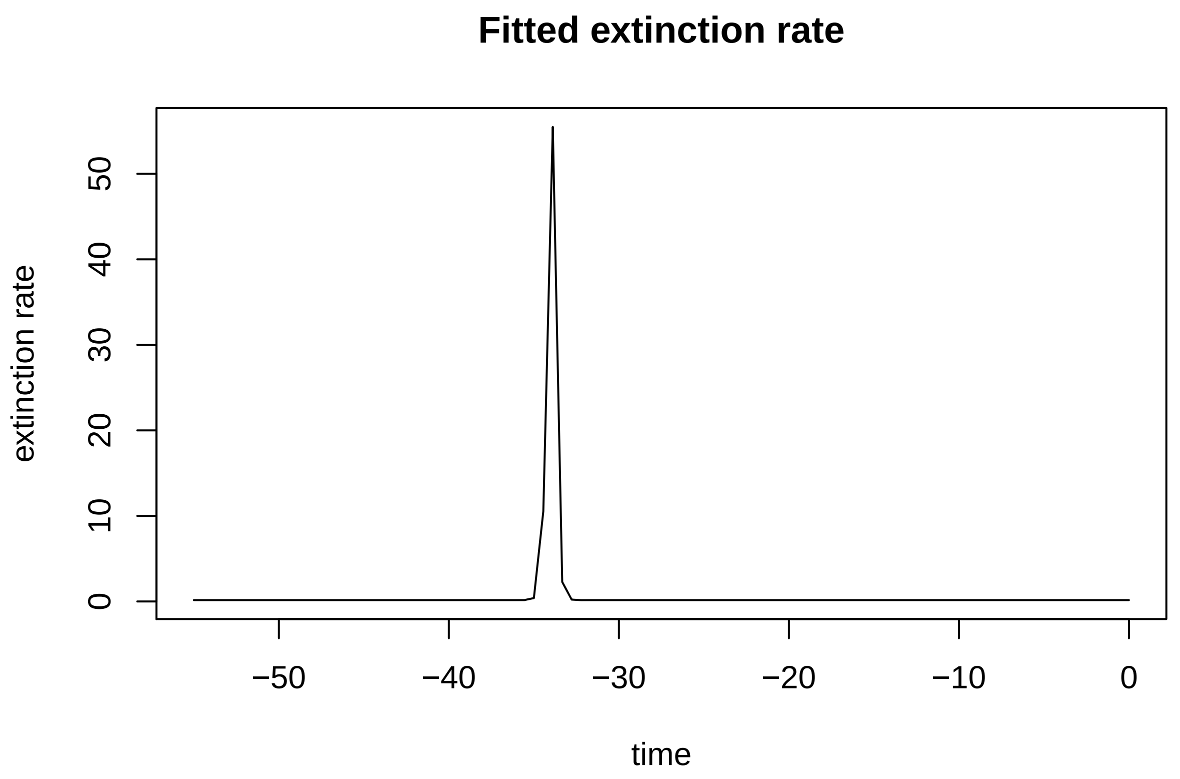


```
plot(env_func(t), res.exp_34$f.mu(t), type = "l",
    xlab = "Environmental data", ylab = "extinction rate",
    main = "Fitted extinction rate")
```

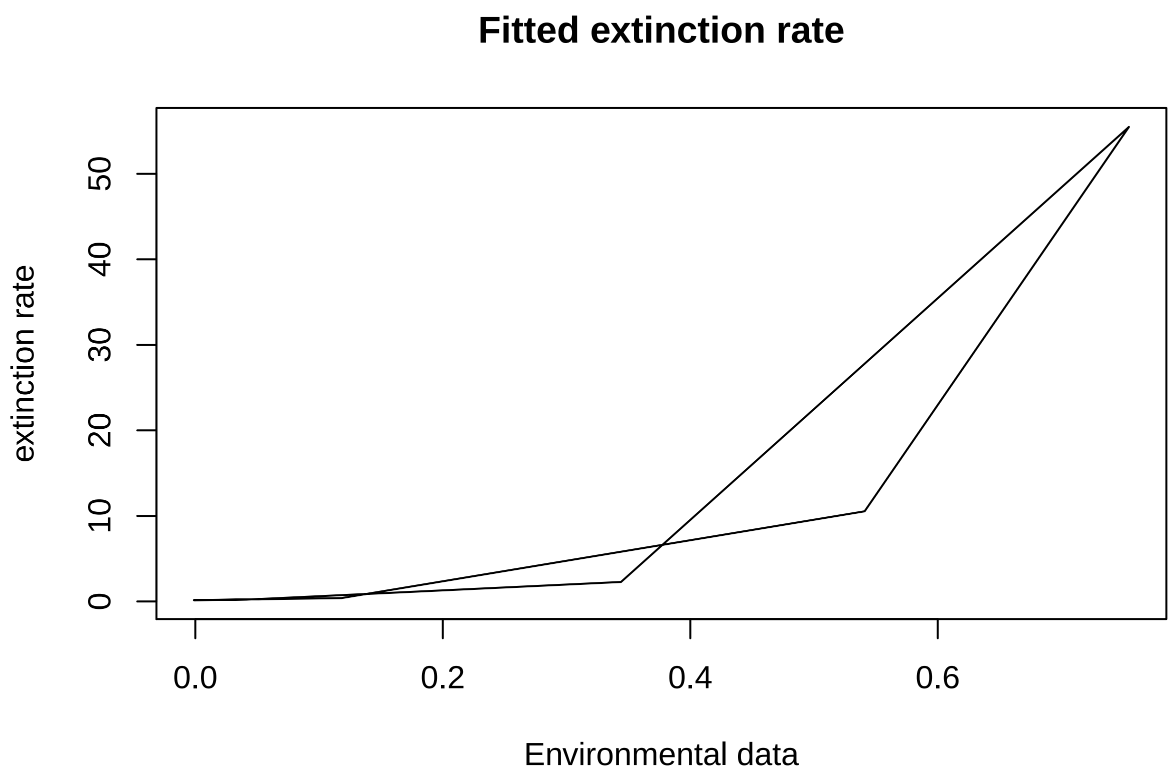


```
r <- function(t) {
    res.exp_34$f.lamb(t) - res.exp_34$f.mu(t)
}

plot(-t, r(t), type = "l", xlab = "time",
    ylab = "net diversification rate", main = "Fitted net diversification rate")
```

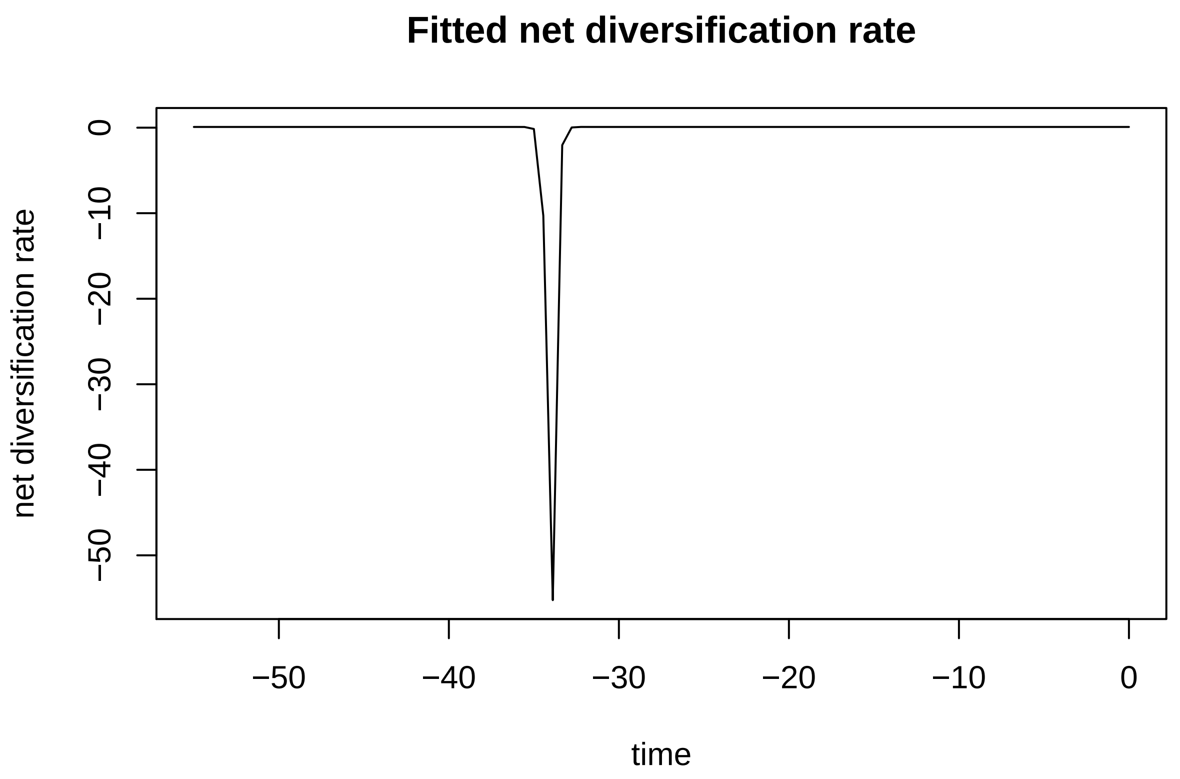


```r
plot(env_func(t), r(t), type = "l", xlab = "Environmental data",
    ylab = "net diversification rate", main = "Fitted net diversification rate")
```

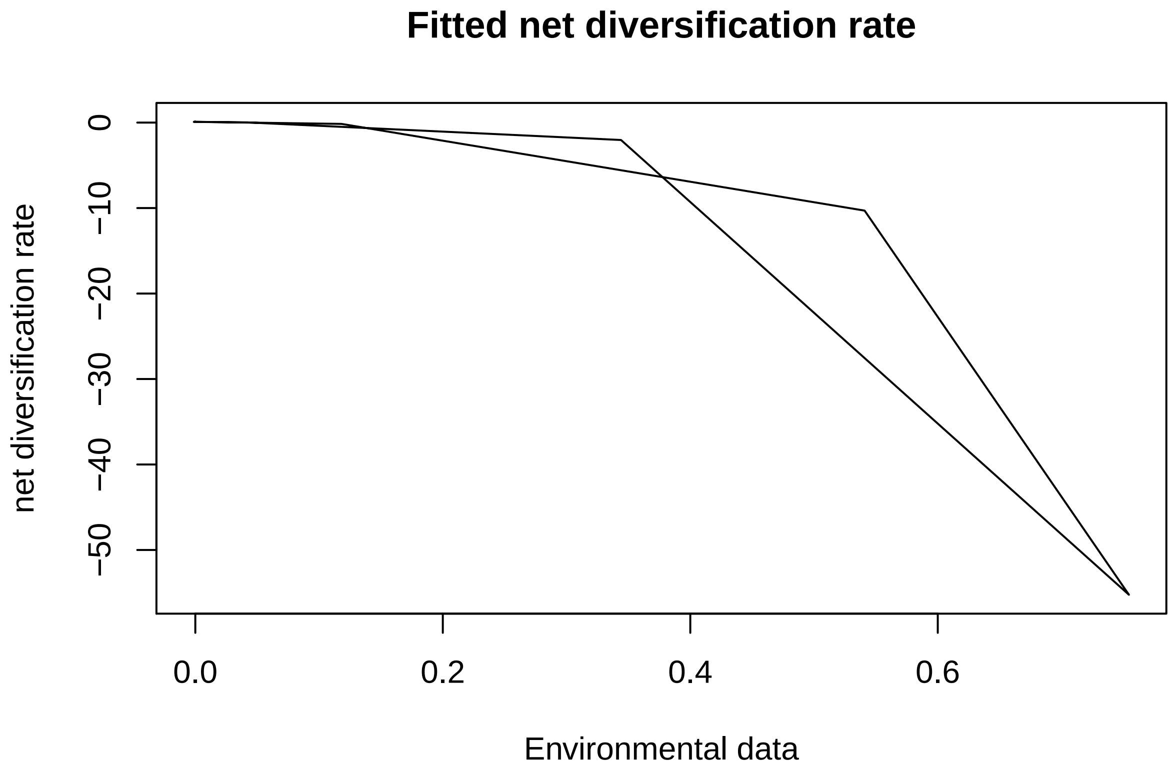


```
#    }
```

The plots above describe exactly what we would expect, given the way we set up the model.

We see that 1) diversification rate is constant 2) diversification rate is not correlated with the environmental pulse 3) We are saying that the extinction rate spiked extremely high at 34 Ma where 4) high values of the pulse variable are found - the fourth plot shows just that the few high values of environmental pulse variable correspond to high values of extinction rate 5) the net diversification rate plummeted at the time when the spiking extinction rate was observed and 6) the high values of environmental variable show when net diversification rates are low.

Consider Temperature and Rate of Change as Predictor of Extinction Rate

We also consider temperature and its rate of change as predictor variables for extinction rate. We consider both linear and exponential functions of each.

```
lamb_par_init<-c(0.4) #use the previous inits for the constant rate of lambda and varying rate of mu
mu_par_init<-c(0.1,0.05)
f.mu.lin<-function(t,x,y){y[1]+y[2]*x} #linear dependence of extinction rate on P(Mass Extinction)
f.lamb<-function(t,x,y){y[1]} #constant speciation rate,
f.mu.exp<-function(t,x,y){y[1]*exp(y[2]*x)}

newdata=data.frame("Age"=seq(0, 70 ,length.out = 700))
newdata$predY <- predict(G1, type = "response", newdata = newdata)
InfTemp2$Nage=-InfTemp2$Age
newdata$Nage=-newdata$Age
newdata$predY_diff <- c(0,diff(newdata$predY)/diff(newdata$Nage))
```

```
newdata$Null=c(rep(0.001,nrow(newdata)))
#use raw temp
env_data=newdata[,which(names(newdata) %in% c("Age", "predY"))]
res1.lin<-fit_env(lemurs, env_data,tot_time, f.lamb,f.mu.lin,
              lamb_par_init,mu_par_init,f=f,dt=1e-3)
res1.exp<-fit_env(lemurs, env_data,tot_time, f.lamb,f.mu.exp,
              lamb_par_init,mu_par_init,f=f,dt=1e-3)
#use rate
env_data=newdata[,which(names(newdata) %in% c("Age", "predY_diff"))]
res2.lin<-fit_env(lemurs, env_data,tot_time, f.lamb,f.mu.lin,
              lamb_par_init,mu_par_init,f=f,dt=1e-3)
res2.exp<-fit_env(lemurs, env_data,tot_time, f.lamb,f.mu.exp,
              lamb_par_init,mu_par_init,f=f,dt=1e-3)
#the models with nothing
env_data <- newdata[,which(names(newdata) %in% c("Age", "Null"))]
res3.lin<-fit_env(lemurs, env_data,tot_time, f.lamb,f.mu.lin,
              lamb_par_init,mu_par_init,f=f,dt=1e-3)

mods <- ls()[grep(pattern = "res[1-3].[lin|exp]",x=ls())]
mods<- mods[!grepl(mods, pattern = "spec")]
aicc.table3 <- c()
for(i in 1:length(mods)){
  g=get(mods[i])
  aicc.table3 <- rbind(aicc.table3, (cbind(mods[i], g$aicc)))
}
aicc.table3 <- as.data.frame(aicc.table3)
aicc.table3$Extinctions <- c("Temperature", "Temperature",
                             "Rate of Temperature Change", "Rate of Temperature Change",
                             "Null Model")
aicc.table3$Effect <- c(rep(c("Linear", "Exponential"), 2), "")
aicc.table3 <- aicc.table3[,c(4,3,2)]
aicc.table3$V2<-as.numeric(as.character(aicc.table3$V2))
aicc.table3$delta_null<-aicc.table3$V2-aicc.table3$V2[5]
```

Effect	Model	AICc	delta AICc
Linear	Temperature	540.1130	-1.3307
Exponential	Temperature	540.2153	-1.2283
Linear	Rate of Temperature Change	538.5663	-2.8774
Exponential	Rate of Temperature Change	539.0208	-2.4229
	Null Model	541.4437	0.0000

The models with temperature influencing extinction rate are not well supported.

In order to add all RPANDA results together, we need a plot that shows the raw AICc scores.

```
aicc.table3$year_of_ex<-5:1
aicc.table$year_of_ex<-6:9
expression(paste(Delta," Temp. (Lin.)"))
```

```
## expression(paste(Delta, " Temp. (Lin.)"))
```

```
xlabels<-c("Null", " ", " ",
                "Temp. (Exp)", "Temp. (Lin)",
```

```
            "ScstEcst","SvarEcst", "ScstEvar", "SvarEvar"
            )
xlabelspositions<-c(1:9)

par(mar=c(5.5,4.1,1,2))

plot.new()


plot.window(xlim = c(-50,18), ylim = c(533,543))


polygon(c(-31.5, -31.5,-40.5,-40.5), c(533,543,543,533), col = "#DDDDDD", border = "#DDDDDD")
polygon(c(-43.5, -43.5,-45.5,-45.5), c(533,543,543,533), col = "#DDDDDD", border = "#DDDDDD")

points(AICc~year_of_ex, data=res[1:c(nrow(res)-1),], pch=19, col="black")
aicc.table3$year_of_ex<-c(10, 8, 6, 4, 2)
points(AICc~year_of_ex, data=aicc.table3, pch=19, col="gray80")
aicc.table$year_of_ex<-c(12, 14, 16, 18)
points(AICc~year_of_ex, data=aicc.table, pch=1, col="black")

axis(at = c(-50, -40, -30, -20, -10, 0), side = 1)
xlabelspositions<-seq(from=2, to=18, by=2)
text(x=xlabelspositions, y=par()$usr[3]-0.125,labels=xlabels, srt=90, adj=1, xpd=TRUE)
text(x=xlabelspositions[2], y=par()$usr[3]-0.125,labels=expression(paste(Delta," Temp. (Exp)")),
     srt=90, adj=1, xpd=TRUE)
text(x=xlabelspositions[3], y=par()$usr[3]-0.125,labels=expression(paste(Delta," Temp. (Lin)")),
     srt=90, adj=1, xpd=TRUE)

####r right here tight now
axis(at = round(c(-1, axTicks(2)),2), las = 1, side = 2)
mtext(side = 1, text = "Time",line = 3)
mtext(side = 2, text ="AICc", line = 3)
text(-47.5,542,labels="a")

abline(h=min(res$AICc), lty=2)
abline(h=min(res$AICc)+4, lty=2)
```

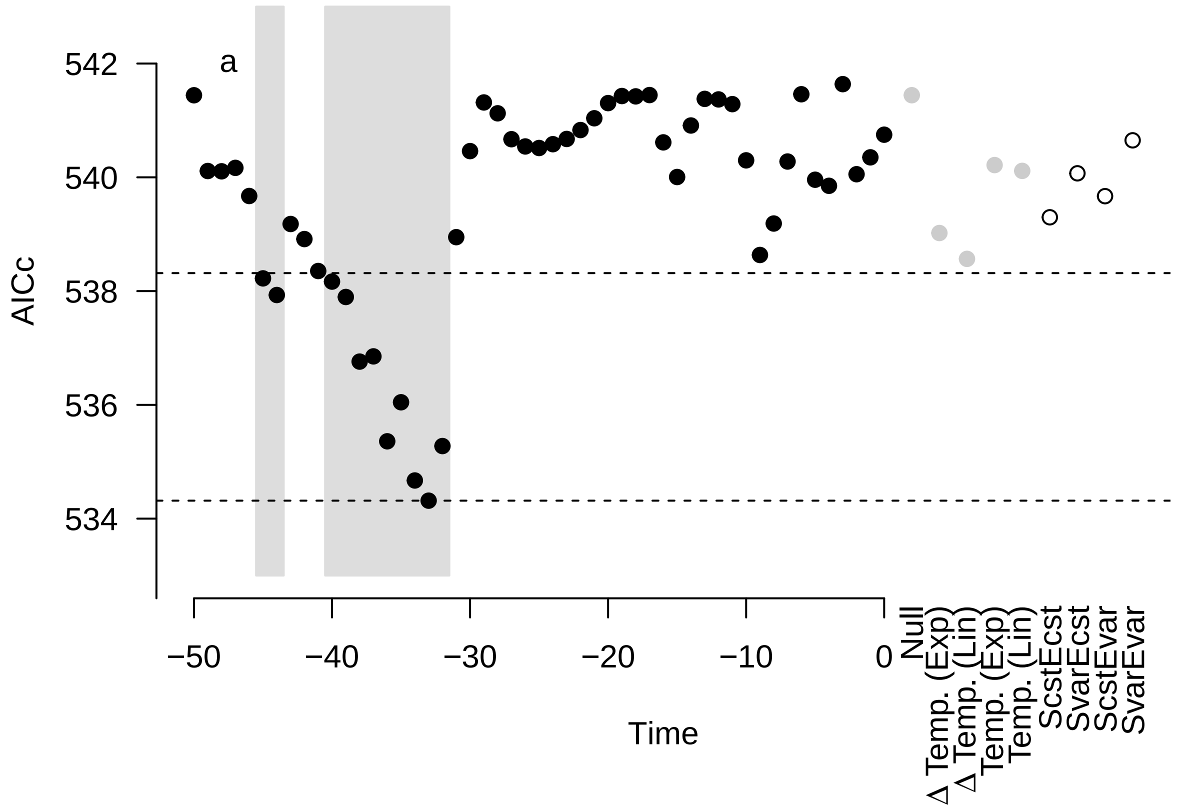


```
ftw<-dev.off()
```

From this it is abundantly clear that the model with an environmental pulse variable with a peak at near 33 Ma is better than most models. The models that posit time-variant speciation and or extinction rates (points with white fill), as well as the models that posit temperature or the rate of change of temperature affecting extinction rates in linear or logarithmic fashions (gray points) fall within the range of poor model fits that are beset by model uncertainty. Dashed lines indicate the range of model AICc scores that fall between 0 and 4 AICc scores from the best-fit model. Models within that range are the best fit models, but likely indistinguishable (gray shading). Models above that range are moderately worse than the best model. The best model posits a pulse variable with a peak at 33 Ma affecting the extinction rate in an exponential manner.

Now finally because some authors have wondered if not perhaps the extinction rate experienced a pulse-like change at some year but rather the speciation rate experienced a pulse-like change, we now repeat our analysis above considering an effect of our simulated environmental pulse variables on the speciation rate. In keeping the speciation rate dependent on the environmental variable, we keep the extinction rate constant. For completeness, we also consider the identical true environmental variables (Temperature and its rate of change) above as predictors of speciation rate.

```
f.lamb.exp2<-function(t,x,y){y[1]*exp(y[2]*x)}
f.lamb.lin2<-function(t,x,y){y[1]*(y[2]*x)}
f.mu2<-function(t,x,y){y[1]}
lamb_par_init2<-c(0.4, 0.05)
mu_par_init2<-c(0.1)

newdata=data.frame("Age"=seq(0, 70 ,length.out = 700))
newdata$predY <- predict(G1, type = "response", newdata = newdata)
```

```
InfTemp2$Nage=-InfTemp2$Age
newdata$Nage=-newdata$Age
newdata$predY_diff <- c(0,diff(newdata$predY)/diff(newdata$Nage))

newdata$Null=c(rep(0.001,nrow(newdata)))
#use raw temp

#start here###################

env_data=newdata[,which(names(newdata) %in% c("Age", "predY"))]
res1.lin_spec<-fit_env(lemurs, env_data,tot_time, f.lamb.lin2,f.mu2,
              lamb_par_init2,mu_par_init2,f=f,dt=1e-3)
res1.exp_spec<-fit_env(lemurs, env_data,tot_time, f.lamb.exp2,f.mu2,
              lamb_par_init2,mu_par_init2,f=f,dt=1e-3)
#use rate
env_data=newdata[,which(names(newdata) %in% c("Age", "predY_diff"))]
res2.lin_spec<-fit_env(lemurs, env_data,tot_time, f.lamb.lin2,f.mu2,
              lamb_par_init2,mu_par_init2,f=f,dt=1e-3)
res2.exp_spec<-fit_env(lemurs, env_data,tot_time, f.lamb.exp2,f.mu2,
              lamb_par_init2,mu_par_init2,f=f,dt=1e-3)
#the models with nothing
env_data <- newdata[,which(names(newdata) %in% c("Age", "Null"))]
res3.lin_spec<-fit_env(lemurs, env_data,tot_time, f.lamb.lin2,f.mu2,
              lamb_par_init2,mu_par_init2,f=f,dt=1e-3)

mods2 <- ls()[grep(pattern = "res[1-3].[lin|exp]",x=ls())]
mods2<-mods2[grepl(pattern = "spec", x=mods2)]
aicc.table3_spec <- c()
for(i in 1:length(mods2)){
  g=get(mods2[i])
  aicc.table3_spec <- rbind(aicc.table3_spec, (cbind(mods2[i], g$aicc)))
}
aicc.table3_spec <- as.data.frame(aicc.table3_spec)
aicc.table3_spec$Speciation <- c("Temperature", "Temperature",
                             "Rate of Temperature Change", "Rate of Temperature Change",
                             "Null Model")
aicc.table3_spec$Effect <- c(rep(c("Linear", "Exponential"), 2), "")
aicc.table3_spec <- aicc.table3_spec[,c(4,3,2)]
aicc.table3_spec$V2<-as.numeric(as.character(aicc.table3_spec$V2))
aicc.table3_spec$delta_null<-aicc.table3_spec$V2-aicc.table3_spec$V2[5]
```

Effect	Model	AICc	Delta Null
Linear	Temperature	540.3802	-1.0635
Exponential	Temperature	588.9298	47.4861
Linear	Rate of Temperature Change	539.5100	-1.9337
Exponential	Rate of Temperature Change	545.9239	4.4802
	Null Model	541.4437	0.0000

```
f.lamb2<-function(t,x,y){y[1]*exp(y[2]*x)}
f.mu.exp2<-function(t,x,y){y[1]}
lamb_par_init2<-c(0.4, 0.05)
```

```
mu_par_init2<-c(0.1)
AICcs2 <- c()
LHs2 <- c()
```

```
for(i in 1:52){
env_data <- killemall_ones[nrow(killemall_ones):1,c(2,c(2+i))] #make sure first row is time zero
res.exp2<-fit_env(lemurs, env_data,tot_time, f.lamb2,f.mu.exp2,
              lamb_par_init2,mu_par_init2,f=f,dt=1e-3)
AICcs2[i] <- res.exp2$aicc
LHs2[i] <- res.exp2$LH
}
res2=data.frame("AICc"=AICcs2,
               "LHs"=LHs2,
               "mod"=names(killemall_ones)[3:54])
```

```
res2$dAICc <- res2$AICc-min(res2$AICc)
res2$RelativeLikelihood<-exp(-0.5*res2$dAICc)
res2$AkaikeWeights<-res2$RelativeLikelihood/sum(res2$RelativeLikelihood)
res2$year_of_ex<-c(-50:0, NA)
aicc.table3_spec$year_of_ex<-c(10,8,6,4,2)
#Akaike


plot.new()
plot.window(xlim = c(-50,9), ylim = c(533,590))
points(AICc~year_of_ex, data=res2[1:c(nrow(res2)-1),], pch=19, col="#4DAF4A")
points(AICc~year_of_ex, data=aicc.table3_spec, pch=19, col="red")
title("Variables Influencing Speciation Rate")

axis(at = c(-50, -40, -30, -20, -10, 0), side = 1)
xlabelspositions<-seq(from=2, to=18, by=2)
text(x=xlabelspositions[1:5], y=par()$usr[3]-0.125,labels=xlabels[1:5], srt=90, adj=1, xpd=TRUE)
text(x=xlabelspositions[2], y=par()$usr[3]-0.125,labels=expression(paste(Delta," Temp. (Exp)")),
     srt=90, adj=1, xpd=TRUE)
text(x=xlabelspositions[3], y=par()$usr[3]-0.125,labels=expression(paste(Delta," Temp. (Lin)")),
     srt=90, adj=1, xpd=TRUE)

axis(at = round(c(-1, axTicks(2)),2), las = 1, side = 2)
mtext(side = 1, text = "Time",line = 3)
mtext(side = 2, text ="AICc", line = 3)

abline(h=min(res2$AICc), lty=2)
abline(h=min(res2$AICc)+4, lty=2)
```

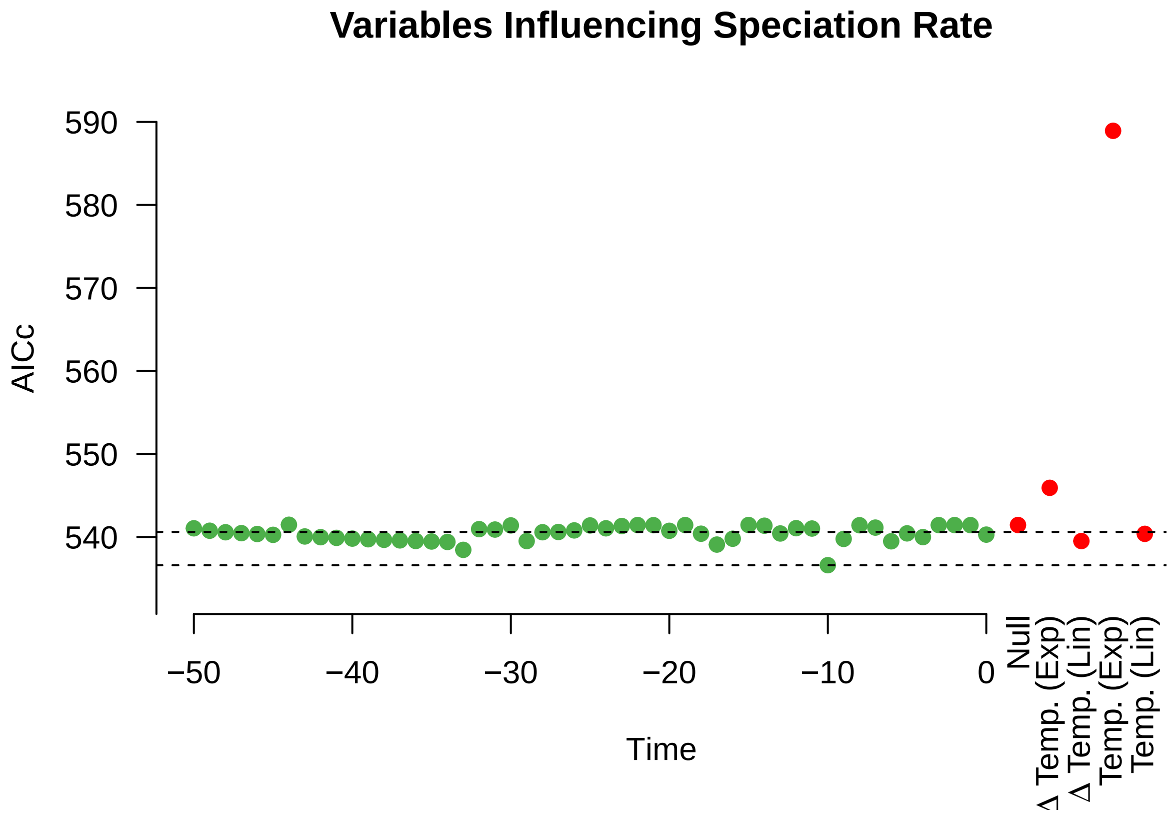


```r
dev.off()
```

```
## null device
##           1
```

We see that model uncertainty is large amongst all candidate models. The best model posits a change in speciation rate at 10 Ma, but it is indistinguishable from models with changes in speciation rates at 50-45, 44-34, 29-26, 18-17 and most models positing an environmental effect of temperature or its rate of change on speciation rates. Thus we conclude that there is negligible evidence to support the hypothesis that speciation rate shifted anywhere over the tree or was significantly influenced by environmental variables.

3.2. TESS

For the tests for mass extinctions with TESS we first consider the convergence of the chains.

```r
tess.plot.multichain.diagnostics(l, parameters = "mass extinction times")
```

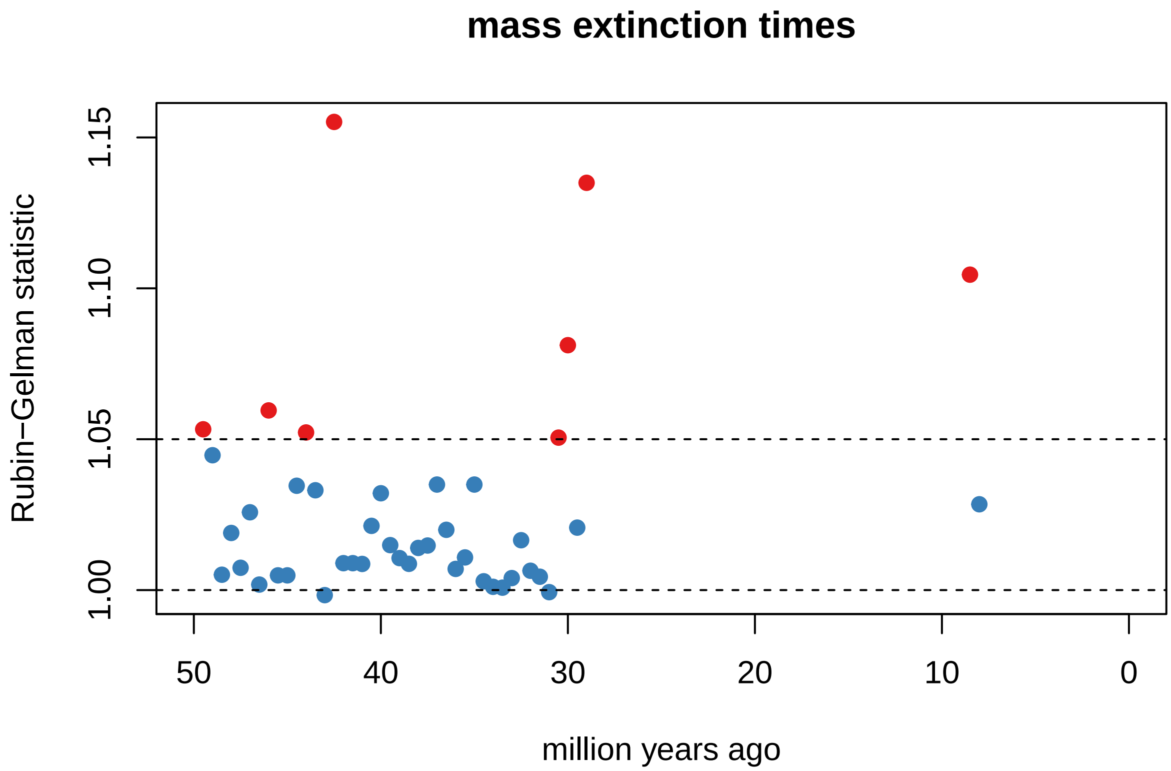

Two of those times seem to show borderline convergence errors with convergence above 1.1. But a few more chains could bring them down, as these are measures of between-chain to within-chain variation. We continue with single chain convergence diagnostics.

```
tess.plot.singlechain.diagnostics(l[[1]], parameters = "mass extinction times")
```

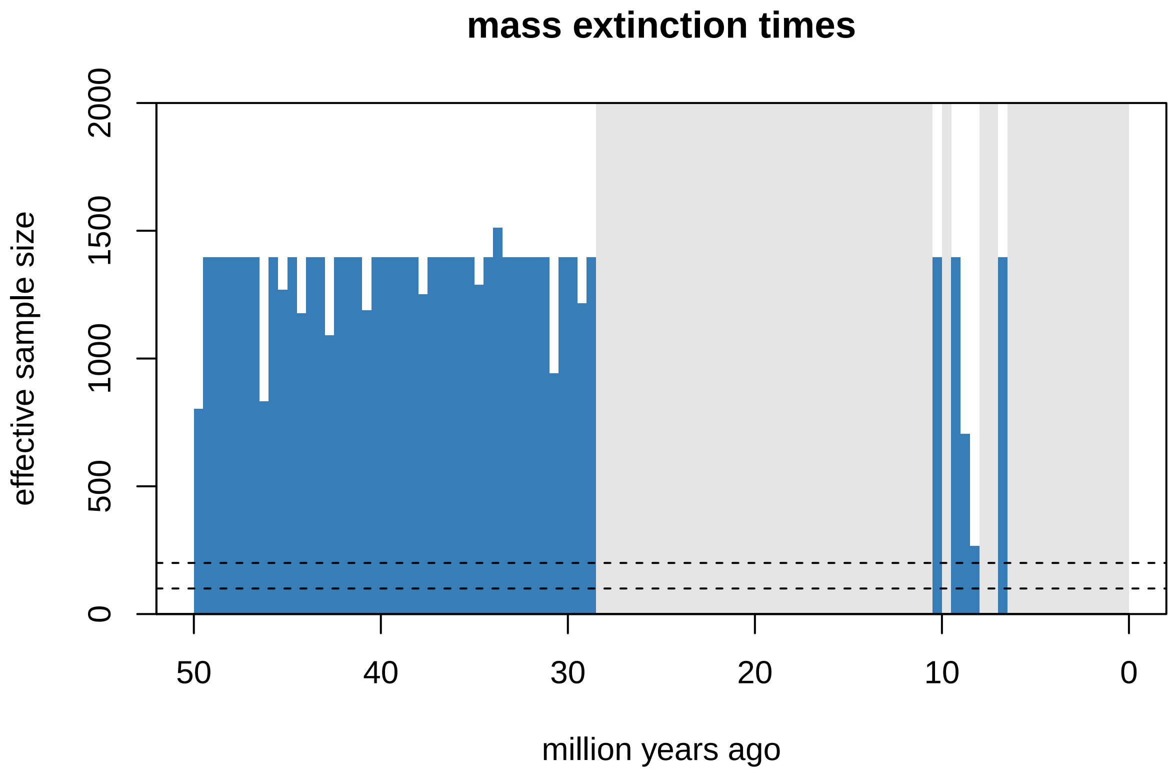
mass extinction times
effective sample size
0
500
1000
1500
2000
50
40
30
20
10
0
million years ago

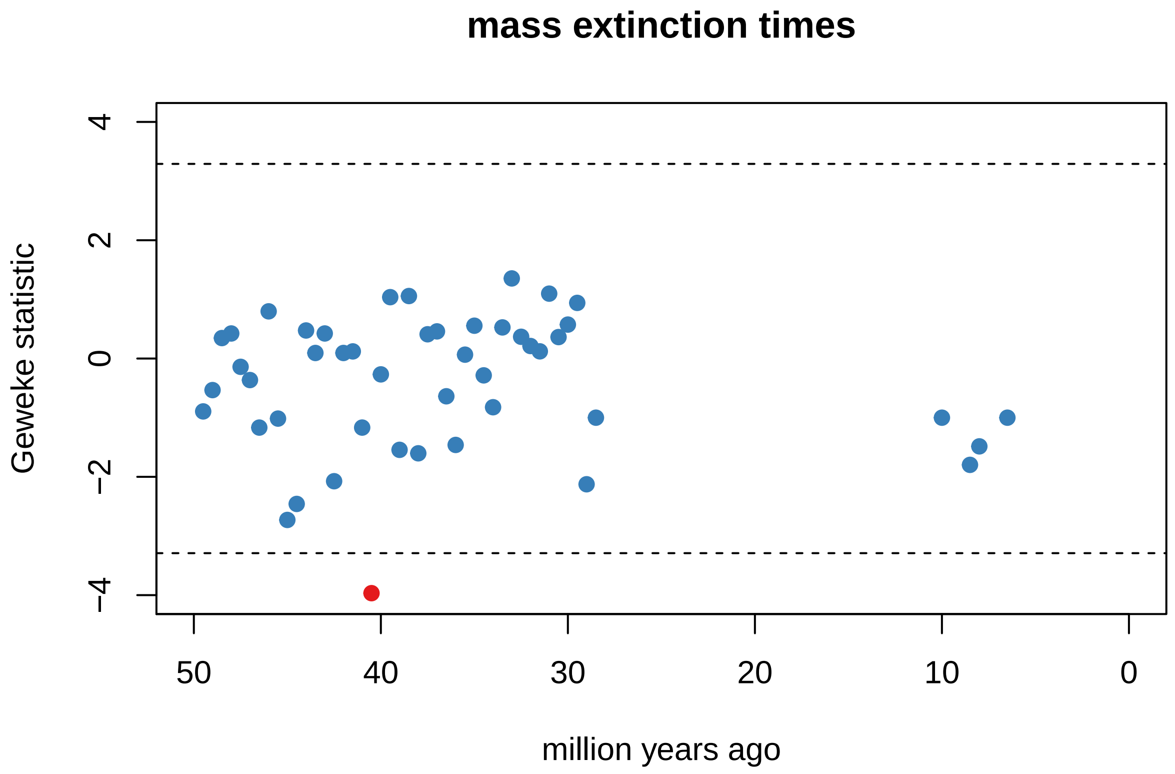


```
tess.plot.singlechain.diagnostics(l[[2]], parameters = "mass extinction times")
```

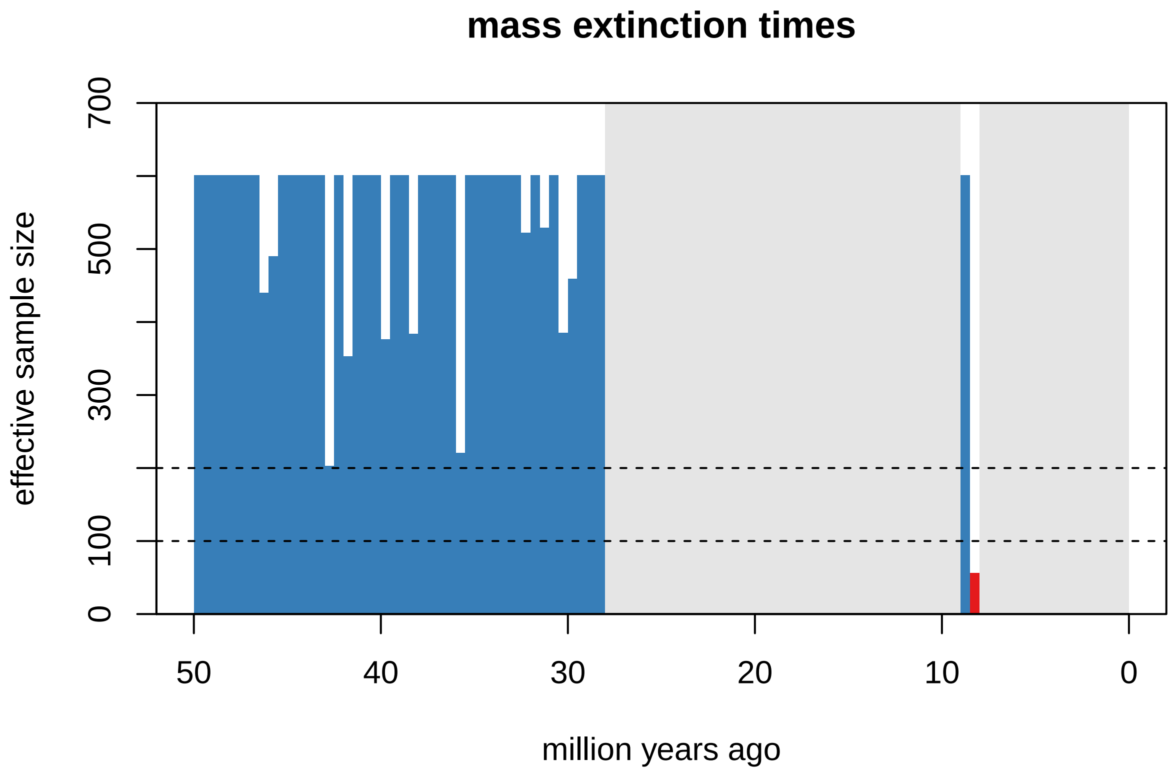
mass extinction times
effective sample size
0
100
300
500
700
50
40
30
20
10
0
million years ago

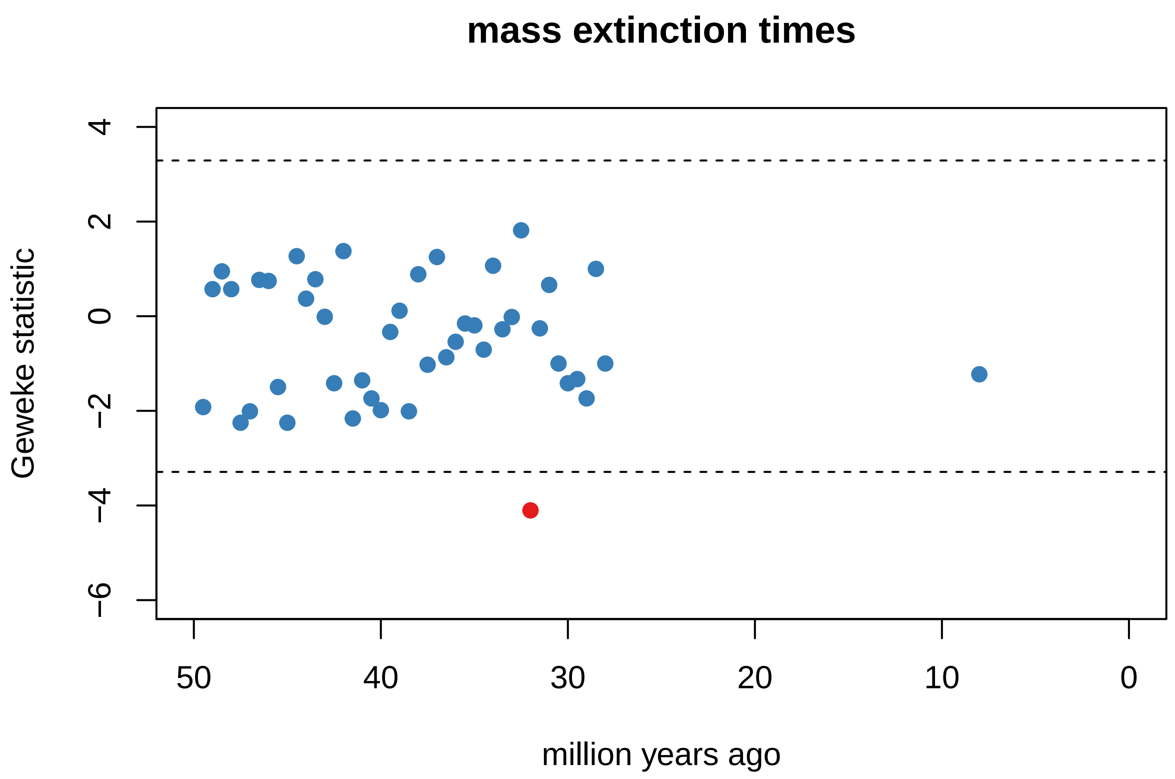


```
tess.plot.singlechain.diagnostics(l[[3]], parameters = "mass extinction times")
```

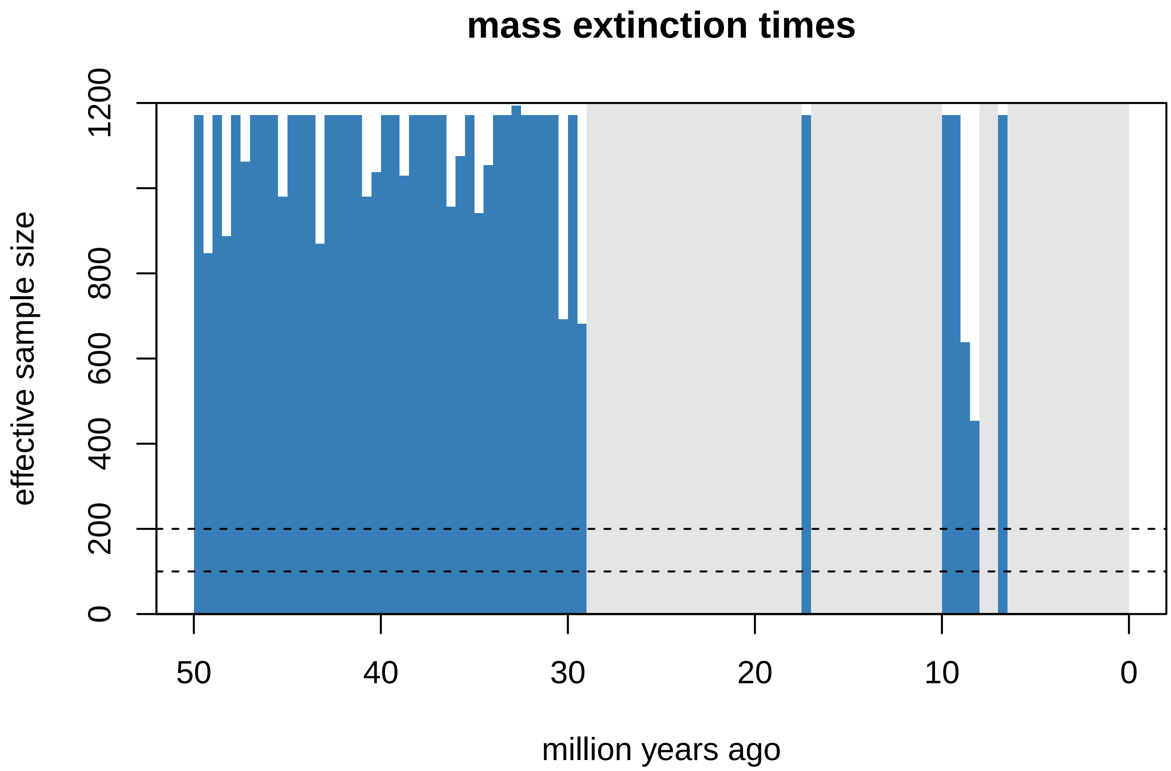
mass extinction times
effective sample size
0
200
400
600
800
1200
50
40
30
20
10
0
million years ago

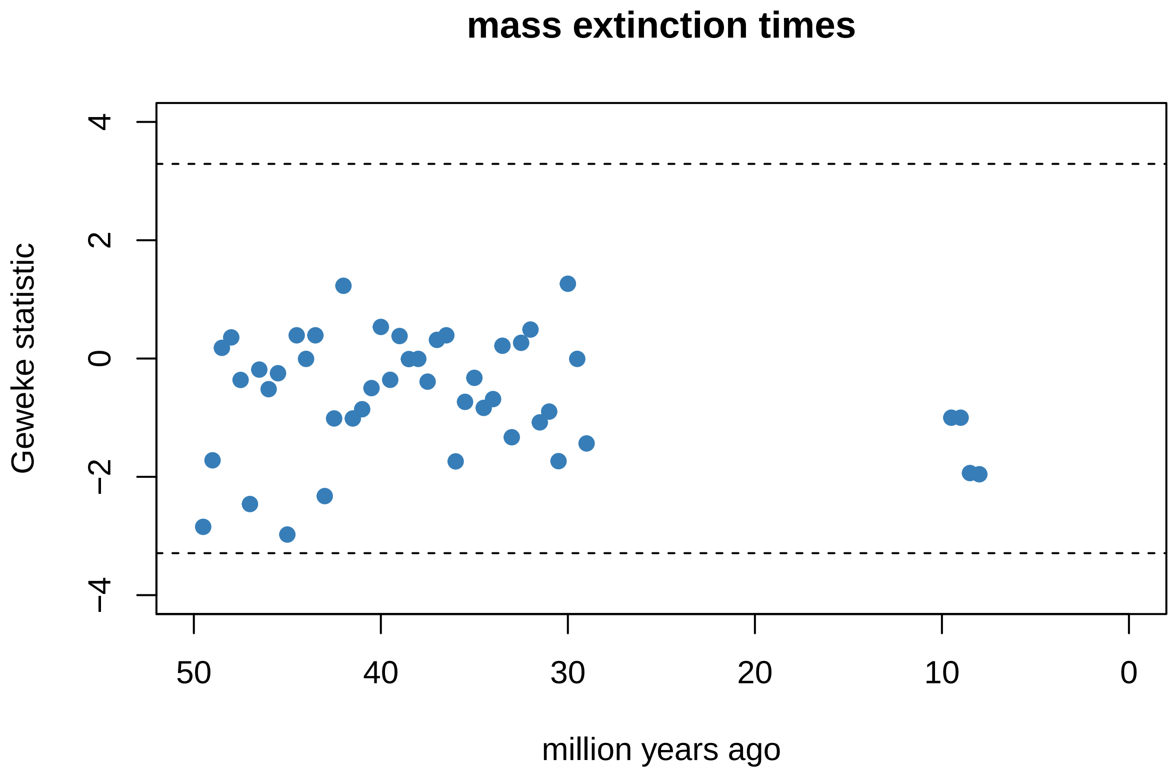


```
tess.plot.singlechain.diagnostics(l[[4]], parameters = "mass extinction times")
```

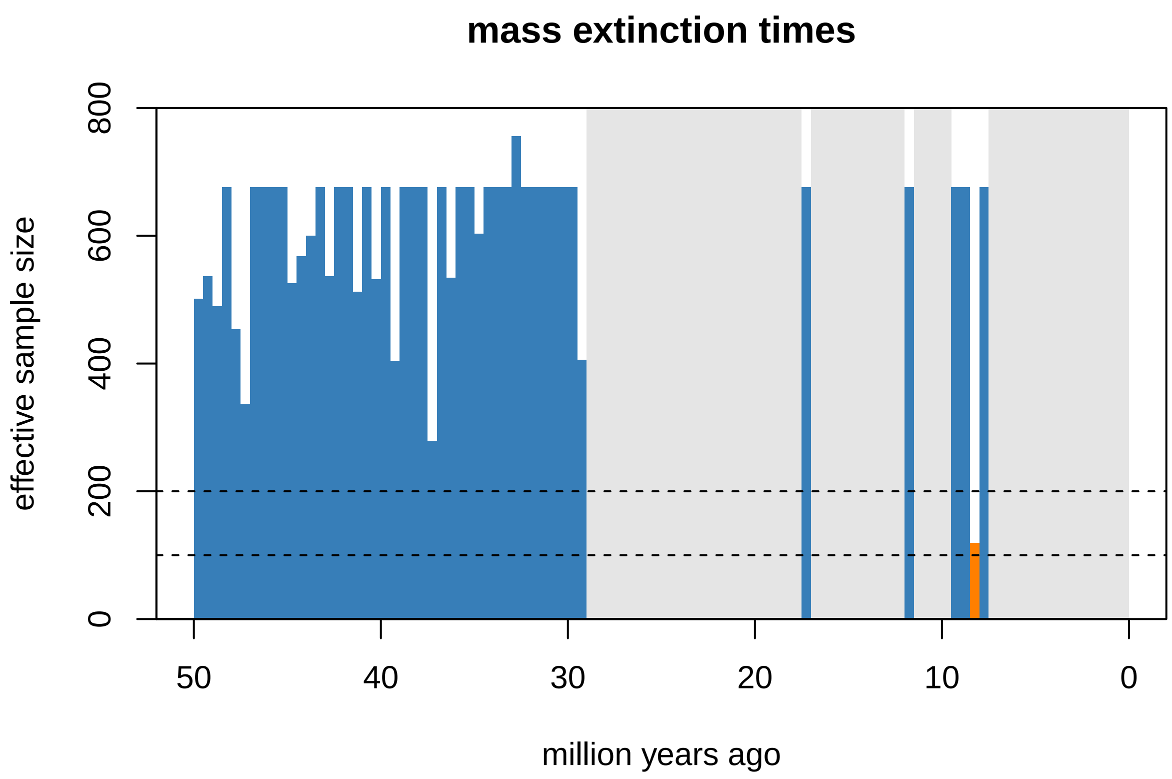

mass extinction times
effective sample size
0
200
400
600
800
50
40
30
20
10
0
million years ago

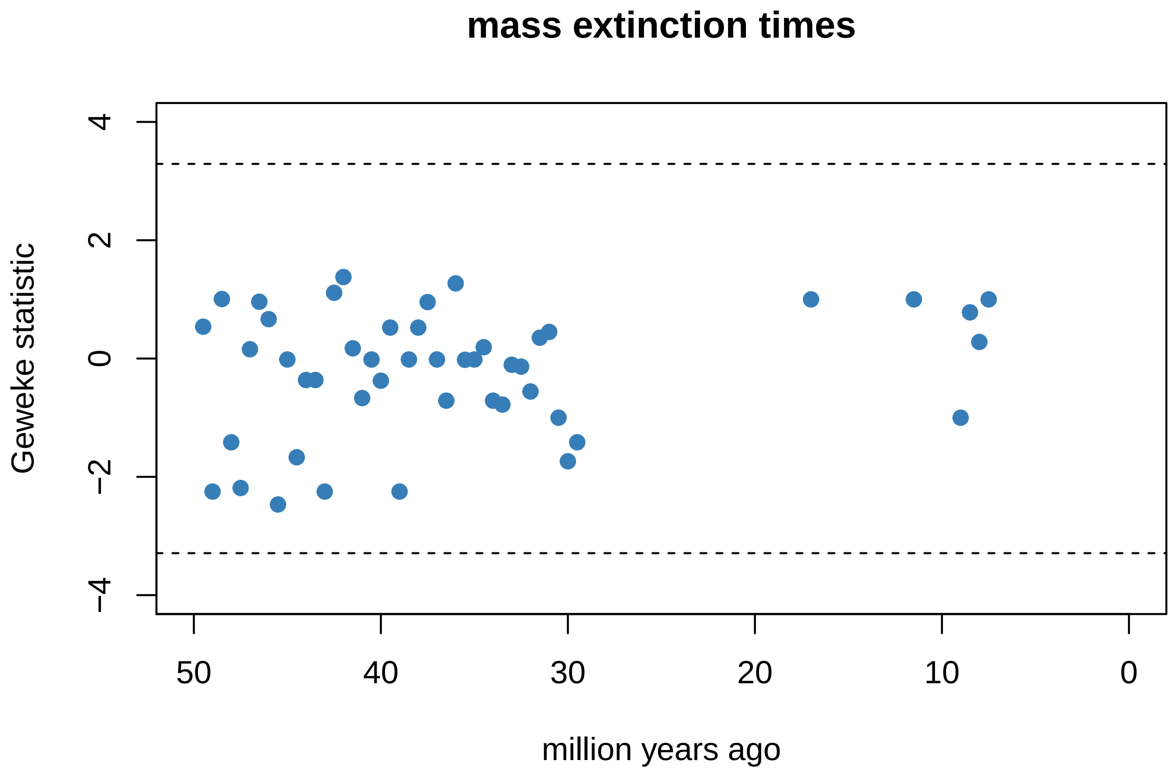

Effective sample size is sufficient and burn-in has been sufficient for chains 3 and 4, and almost with chains 1 and 2, which each have one almost-converged point.

Do the inferences change when considering each chain individually?

```
tess.plot.output(l[[1]], "mass extinction times")
```

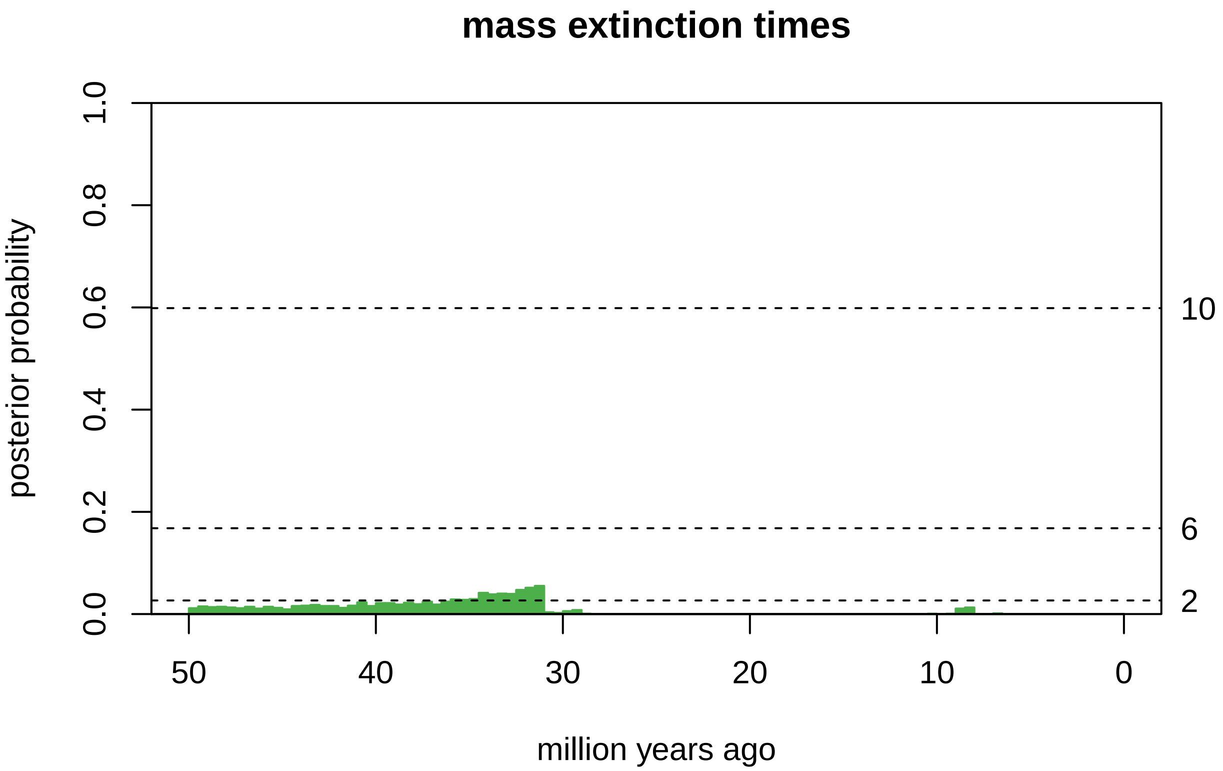


```
tess.plot.output(l[[2]], "mass extinction times")
```

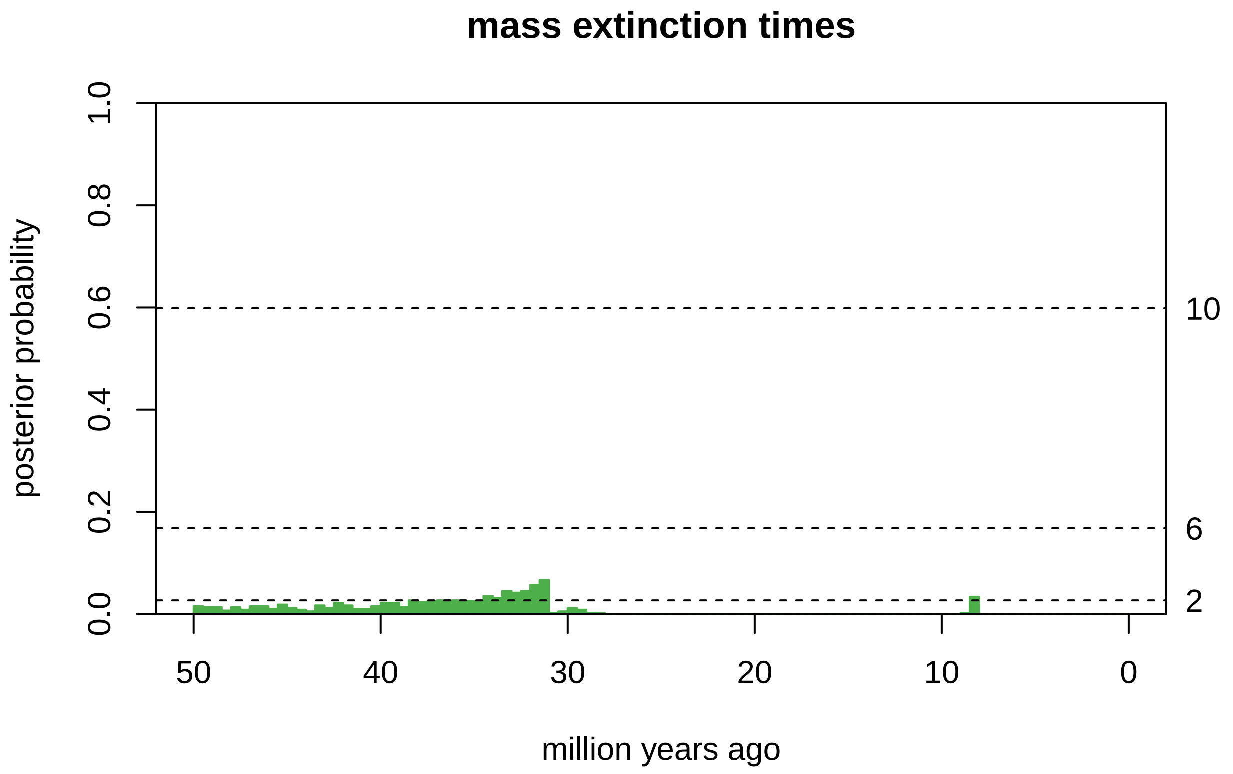


```r
tess.plot.output(l[[3]], "mass extinction times")
```

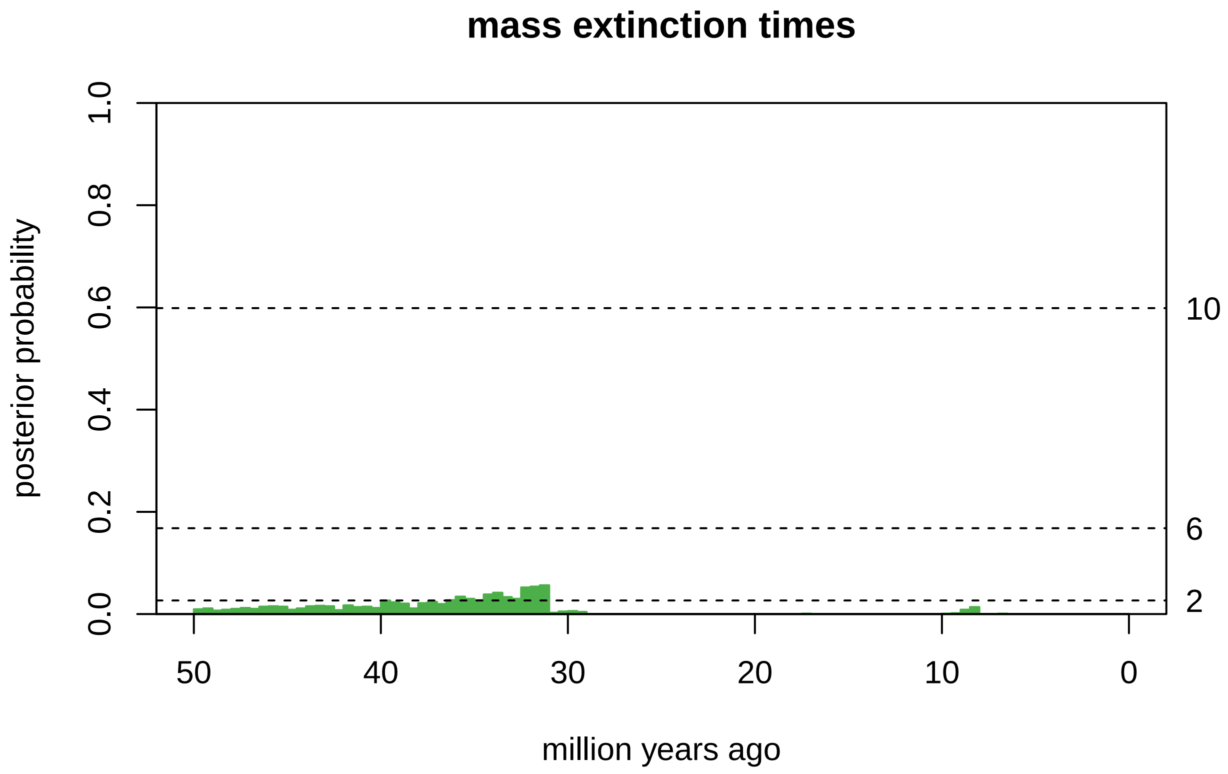


```
tess.plot.output(l[[4]], "mass extinction times")
```

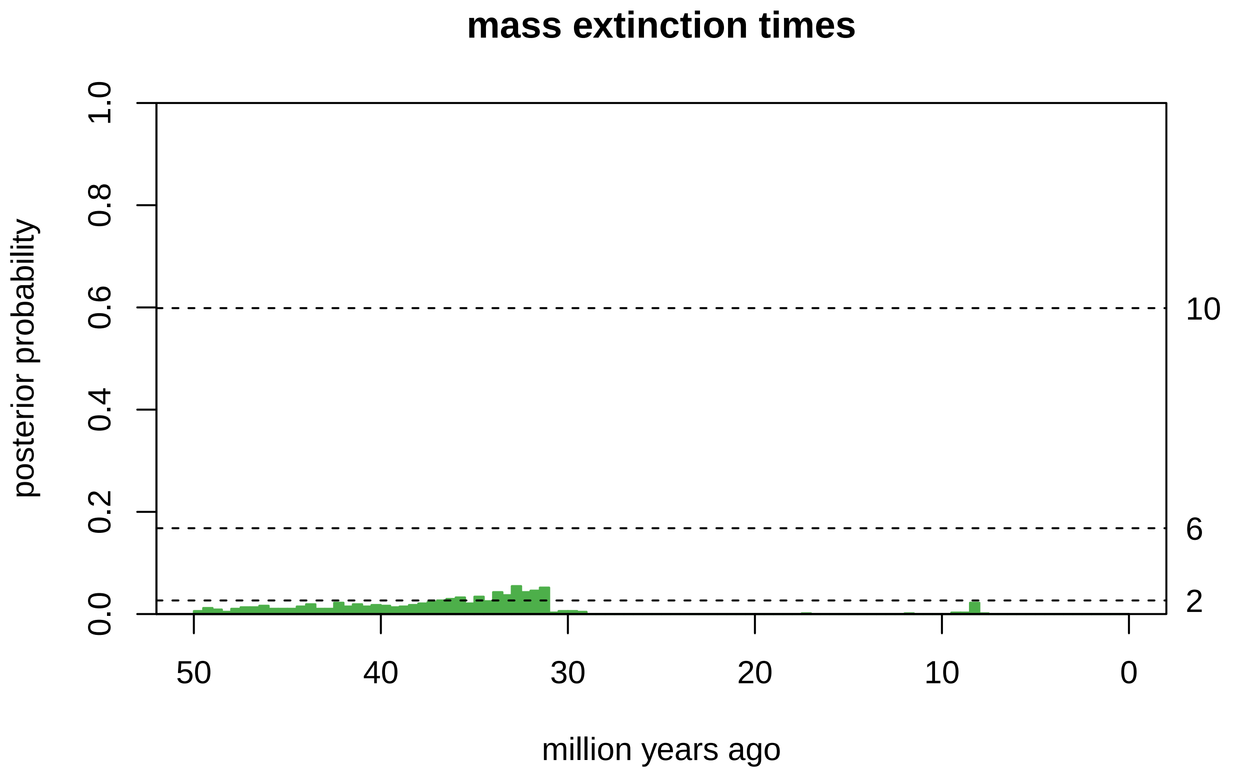

All chains show moderate evidence for a mass extinction happening near the EO boundary, and some chains show almost moderate for the extinction happening near 9 Ma. We choose chain 3 as above.

```
thisOutput <- output3[["mass extinction Bayes factors"]]
ylim <- range(c(thisOutput, -10, 10), finite = TRUE)
col <- c(`speciation rates` = "#984EA3",
         `speciation shift times` = "#984EA3",
            `speciation Bayes factors` = "#984EA3",
         `extinction rates` = "#E41A1C",
            `extinction shift times` = "#E41A1C",
         `extinction Bayes factors` = "#E41A1C",
            `net-diversification rates` = "#377EB8",
         `relative-extinction rates` = "#FF7F00",
            `mass extinction times` = "#4DAF4A",
         `mass extinction Bayes factors` = "#4DAF4A")
treeAge <- max(branching.times(output$tree))
numIntervals <- length(output$intervals) - 1
plotAt <- 0:numIntervals
intervalSize <- treeAge/numIntervals
labels <- pretty(c(0, treeAge))
labelsAt <- numIntervals - (labels/intervalSize)
type="mass extinction Bayes factors"
thisOutput <- output3[[type]]


#BF
plot.new()
```

```r
plot.window(xlim = range((plotAt-100)/2), ylim = c(0,10))
points(x = c(plotAt[-1] - diff(plotAt[1:2])/2-100)/2, y = thisOutput,
       col = col["mass extinction Bayes factors"],pch=19)
abline(h = 2 * log(output$criticalBayesFactors[1:2]), lty = 2)
axis(at = c(round(1.2 * 50), axTicks(1)), side = 1)
axis(at = round(c(-1, axTicks(2)),2), las = 1, side = 2)
mtext(side = 1, text = "Time",line = 3)
mtext(side = 2, text = "Bayes Factors", line = 3)
text(-45,10,labels="b")
```

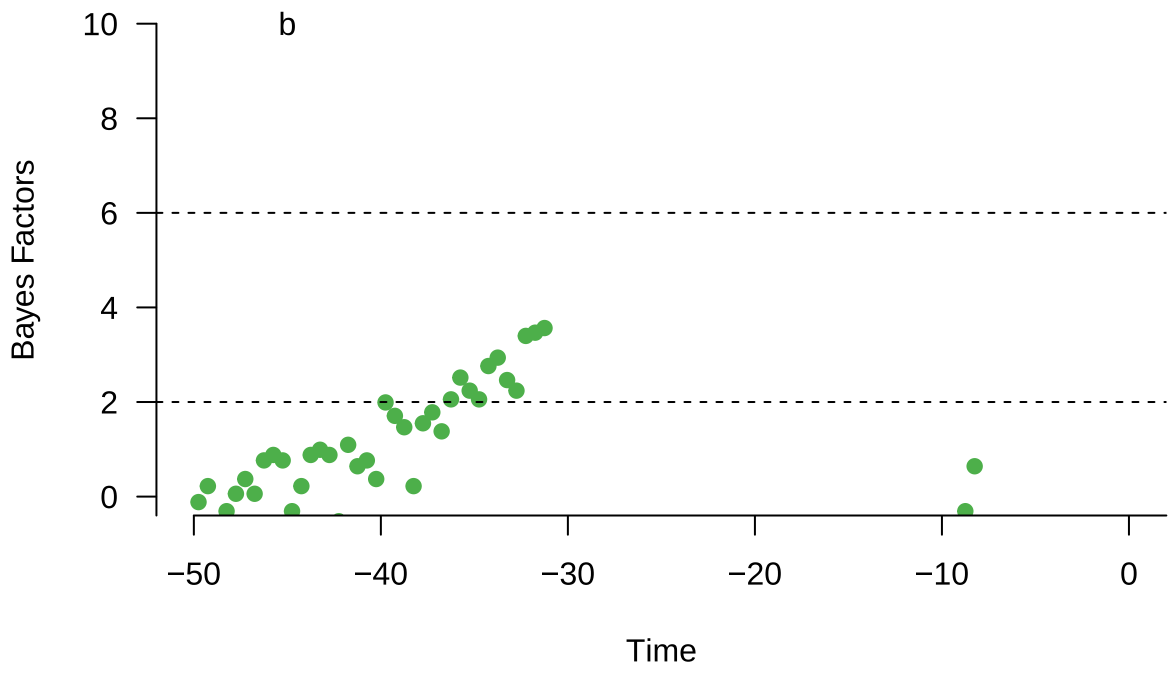


```r
#report raw values
cbind(c(plotAt[-1] - diff(plotAt[1:2])/2-100)/2,
      thisOutput
      )
```

```
##                 thisOutput
##    [1,] -49.75 -0.11622798
##    [2,] -49.25  0.22133144
##    [3,] -48.75 -0.75830118
##    [4,] -48.25 -0.30857174
##    [5,] -47.75  0.05951965
##    [6,] -47.25  0.37127524
##    [7,] -46.75  0.05951965
##    [8,] -46.25  0.76477983
##    [9,] -45.75  0.88083051
##   [10,] -45.25  0.76477983
```

```
##  [11,] -44.75 -0.30857174
##  [12,] -44.25  0.22133144
##  [13,] -43.75  0.88083051
##  [14,] -43.25  0.99070031
##  [15,] -42.75  0.88083051
##  [16,] -42.25 -0.52101468
##  [17,] -41.75  1.09502377
##  [18,] -41.25  0.64179824
##  [19,] -40.75  0.76477983
##  [20,] -40.25  0.37127524
##  [21,] -39.75  1.99073936
##  [22,] -39.25  1.70743343
##  [23,] -38.75  1.46662947
##  [24,] -38.25  0.22133144
##  [25,] -37.75  1.55001789
##  [26,] -37.25  1.78191773
##  [27,] -36.75  1.37976732
##  [28,] -36.25  2.05599192
##  [29,] -35.75  2.51637599
##  [30,] -35.25  2.24049096
##  [31,] -34.75  2.05599192
##  [32,] -34.25  2.76080340
##  [33,] -33.75  2.93823646
##  [34,] -33.25  2.46397281
##  [35,] -32.75  2.24049096
##  [36,] -32.25  3.39784918
##  [37,] -31.75  3.46597776
##  [38,] -31.25  3.56444029
##  [39,] -30.75 -2.72853971
##  [40,] -30.25 -1.33710175
##  [41,] -29.75 -1.02708292
##  [42,] -29.25 -1.70346087
##  [43,] -28.75        -Inf
##  [44,] -28.25        -Inf
##  [45,] -27.75        -Inf
##  [46,] -27.25        -Inf
##  [47,] -26.75        -Inf
##  [48,] -26.25        -Inf
##  [49,] -25.75        -Inf
##  [50,] -25.25        -Inf
##  [51,] -24.75        -Inf
##  [52,] -24.25        -Inf
##  [53,] -23.75        -Inf
##  [54,] -23.25        -Inf
##  [55,] -22.75        -Inf
##  [56,] -22.25        -Inf
##  [57,] -21.75        -Inf
##  [58,] -21.25        -Inf
##  [59,] -20.75        -Inf
##  [60,] -20.25        -Inf
##  [61,] -19.75        -Inf
##  [62,] -19.25        -Inf
##  [63,] -18.75        -Inf
##  [64,] -18.25        -Inf
```

```
## [65,] -17.75        -Inf
## [66,] -17.25 -4.92918602
## [67,] -16.75        -Inf
## [68,] -16.25        -Inf
## [69,] -15.75        -Inf
## [70,] -15.25        -Inf
## [71,] -14.75        -Inf
## [72,] -14.25        -Inf
## [73,] -13.75        -Inf
## [74,] -13.25        -Inf
## [75,] -12.75        -Inf
## [76,] -12.25        -Inf
## [77,] -11.75        -Inf
## [78,] -11.25        -Inf
## [79,] -10.75        -Inf
## [80,] -10.25        -Inf
## [81,]  -9.75 -4.92918602
## [82,]  -9.25 -3.54118152
## [83,]  -8.75 -0.30857174
## [84,]  -8.25  0.64179824
## [85,]  -7.75        -Inf
## [86,]  -7.25        -Inf
## [87,]  -6.75 -4.92918602
## [88,]  -6.25        -Inf
## [89,]  -5.75        -Inf
## [90,]  -5.25        -Inf
## [91,]  -4.75        -Inf
## [92,]  -4.25        -Inf
## [93,]  -3.75        -Inf
## [94,]  -3.25        -Inf
## [95,]  -2.75        -Inf
## [96,]  -2.25        -Inf
## [97,]  -1.75        -Inf
## [98,]  -1.25        -Inf
## [99,]  -0.75        -Inf
## [100,]  -0.25       -Inf
```

Now we look at where the sensitivity analysis places the extinctions.

Weak Prior 1

```
output_2 <- tess.process.output("tess_analysis_thinpriors_mu_lambda_weakextinction1",
numExpectedRateChanges = numExpectedRateChanges,
numExpectedMassExtinctions = numExpectedMassExtinctions)
tess.plot.singlechain.diagnostics(output_2,parameters = c("mass extinction times"),las=2)
```

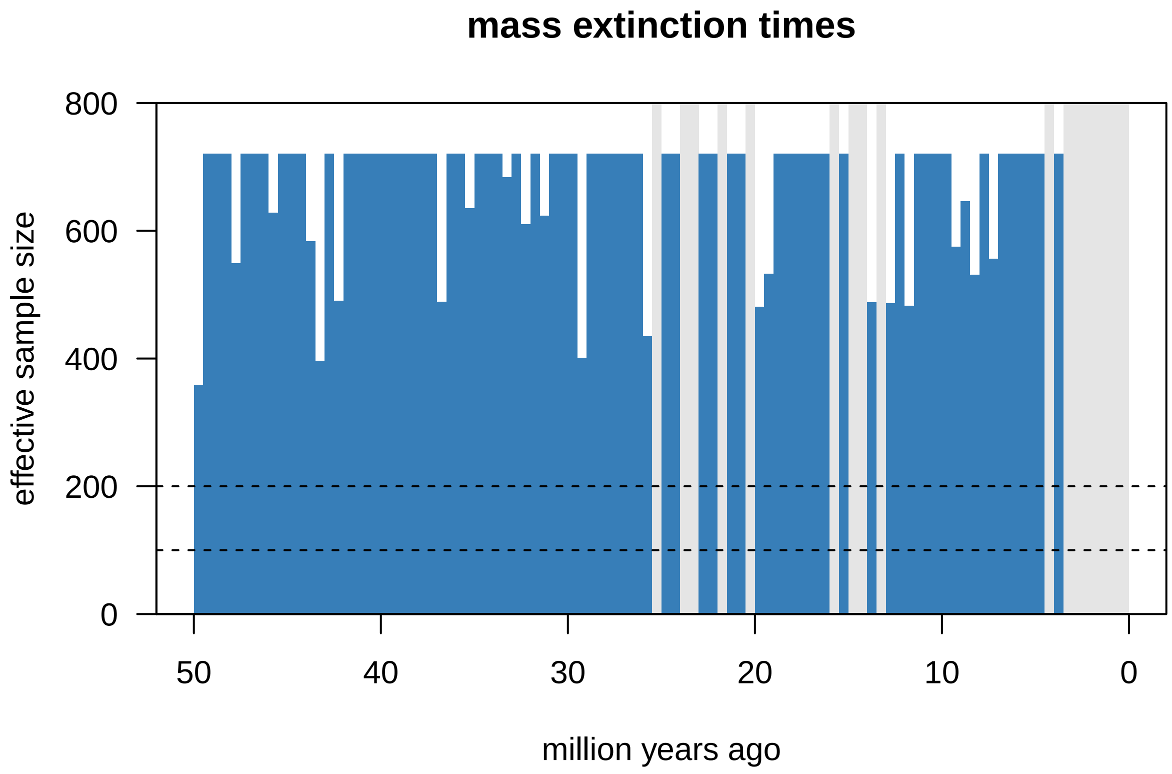
mass extinction times
effective sample size
800
600
400
200
0
50
40
30
20
10
0
million years ago

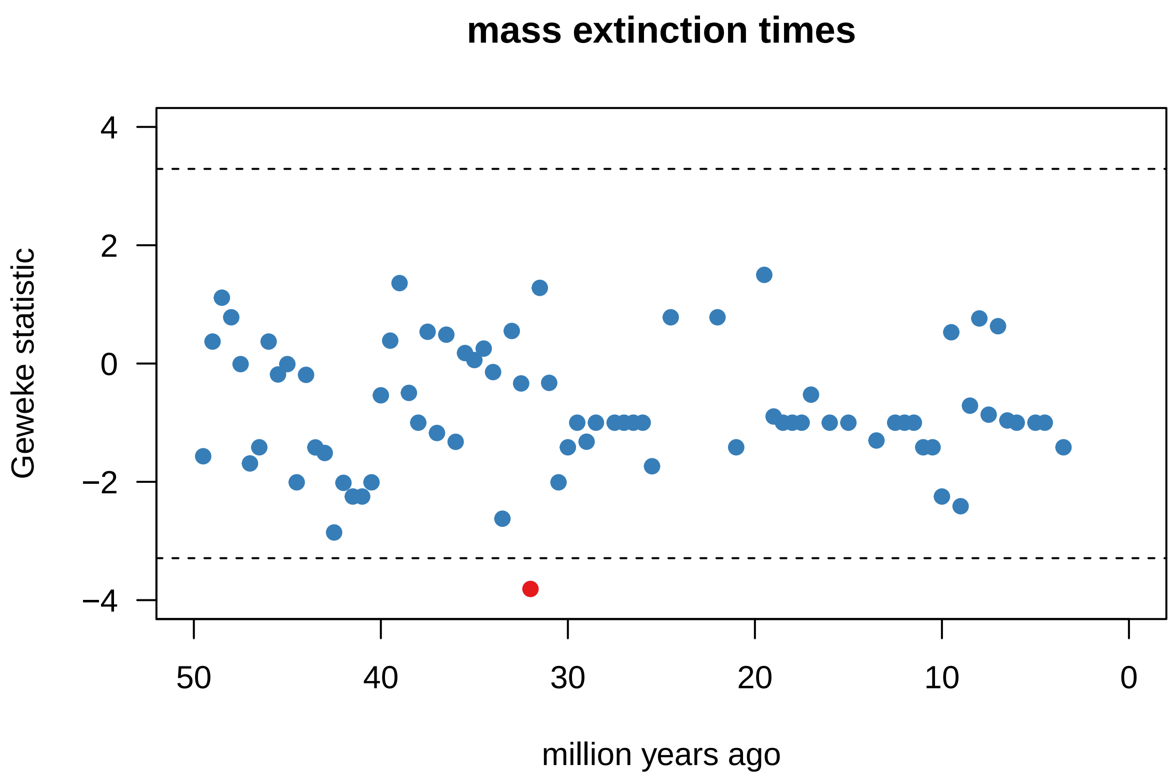


```
tess.plot.output(output_2,fig.types =  c("mass extinction times"),las=2)
```

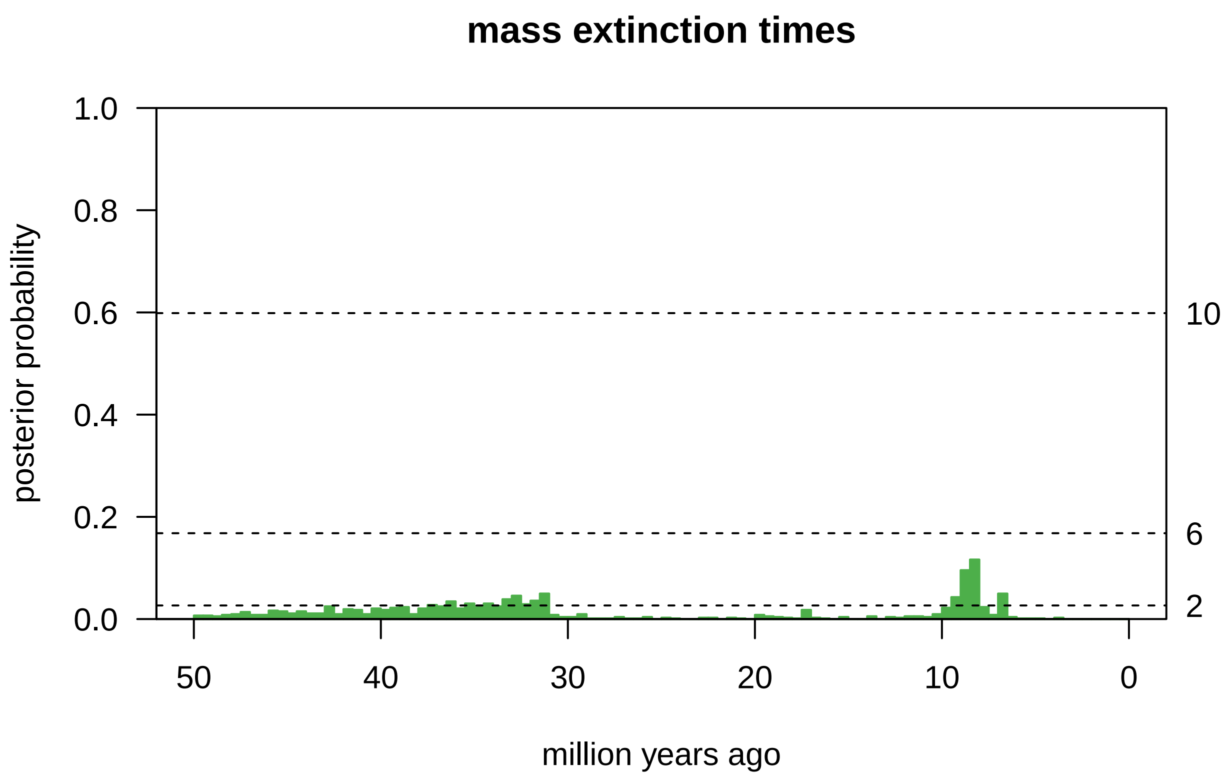


```
op2<-cbind(output_2$`mass extinction Bayes factors`,
      output_2$intervals)
op2
```

```
##                [,1] [,2]
##   [1,] -0.72815247 50.0
##   [2,] -0.72815247 49.5
##   [3,] -1.17723092 49.0
##   [4,] -0.36071411 48.5
##   [5,] -0.04961359 48.0
##   [6,]  0.67215737 47.5
##   [7,] -0.36071411 47.0
##   [8,] -0.36071411 46.5
##   [9,]  1.04243429 46.0
##  [10,]  0.86559264 45.5
##  [11,]  0.22025228 45.0
##  [12,]  0.86559264 44.5
##  [13,]  0.22025228 44.0
##  [14,]  0.22025228 43.5
##  [15,]  1.87036177 43.0
##  [16,] -0.04961359 42.5
##  [17,]  1.35638537 42.0
##  [18,]  1.20534257 41.5
##  [19,] -0.04961359 41.0
##  [20,]  1.49720197 40.5
##  [21,]  1.20534257 40.0
```

```
##  [22,]  1.62911388 39.5
##  [23,]  1.75320201 39.0
##  [24,] -0.04961359 38.5
##  [25,]  1.49720197 38.0
##  [26,]  2.08678081 37.5
##  [27,]  1.87036177 37.0
##  [28,]  2.54738437 36.5
##  [29,]  1.49720197 36.0
##  [30,]  2.28311546 35.5
##  [31,]  1.75320201 35.0
##  [32,]  2.28311546 34.5
##  [33,]  1.87036177 34.0
##  [34,]  2.78268106 33.5
##  [35,]  3.12576949 33.0
##  [36,]  2.18721624 32.5
##  [37,]  2.62870142 32.0
##  [38,]  3.30853224 31.5
##  [39,] -0.36071411 31.0
##  [40,] -1.75538252 30.5
##  [41,] -1.75538252 30.0
##  [42,] -0.04961359 29.5
##  [43,] -3.95817039 29.0
##  [44,] -3.95817039 28.5
##  [45,] -3.95817039 28.0
##  [46,] -1.75538252 27.5
##  [47,] -3.95817039 27.0
##  [48,] -3.95817039 26.5
##  [49,] -1.75538252 26.0
##  [50,]        -Inf 25.5
##  [51,] -2.56909632 25.0
##  [52,] -3.95817039 24.5
##  [53,]        -Inf 24.0
##  [54,]        -Inf 23.5
##  [55,] -2.56909632 23.0
##  [56,] -2.56909632 22.5
##  [57,]        -Inf 22.0
##  [58,] -2.56909632 21.5
##  [59,] -3.95817039 21.0
##  [60,]        -Inf 20.5
##  [61,] -0.36071411 20.0
##  [62,] -1.17723092 19.5
##  [63,] -1.75538252 19.0
##  [64,] -2.56909632 18.5
##  [65,] -3.95817039 18.0
##  [66,]  1.20534257 17.5
##  [67,] -2.56909632 17.0
##  [68,] -3.95817039 16.5
##  [69,]        -Inf 16.0
##  [70,] -1.75538252 15.5
##  [71,]        -Inf 15.0
##  [72,]        -Inf 14.5
##  [73,] -1.17723092 14.0
##  [74,]        -Inf 13.5
##  [75,] -1.75538252 13.0
```

```
##  [76,] -2.56909632 12.5
##  [77,] -1.17723092 12.0
##  [78,] -1.17723092 11.5
##  [79,] -1.75538252 11.0
##  [80,] -0.04961359 10.5
##  [81,]  1.62911388 10.0
##  [82,]  2.99492325  9.5
##  [83,]  4.70845592  9.0
##  [84,]  5.14842633  8.5
##  [85,]  1.75320201  8.0
##  [86,] -0.36071411  7.5
##  [87,]  3.30853224  7.0
##  [88,] -1.75538252  6.5
##  [89,] -3.95817039  6.0
##  [90,] -3.95817039  5.5
##  [91,] -3.95817039  5.0
##  [92,]        -Inf  4.5
##  [93,] -2.56909632  4.0
##  [94,]        -Inf  3.5
##  [95,]        -Inf  3.0
##  [96,]        -Inf  2.5
##  [97,]        -Inf  2.0
##  [98,]        -Inf  1.5
##  [99,]        -Inf  1.0
## [100,]        -Inf  0.5
## [101,] -0.72815247  0.0
```

Weak Prior 2

```r
output_3 <- tess.process.output("tess_analysis_thinpriors_mu_lambda_weakprior2",
numExpectedRateChanges = numExpectedRateChanges,
numExpectedMassExtinctions = numExpectedMassExtinctions)
tess.plot.singlechain.diagnostics(output_3,parameters = c("mass extinction times"),las=2)
```

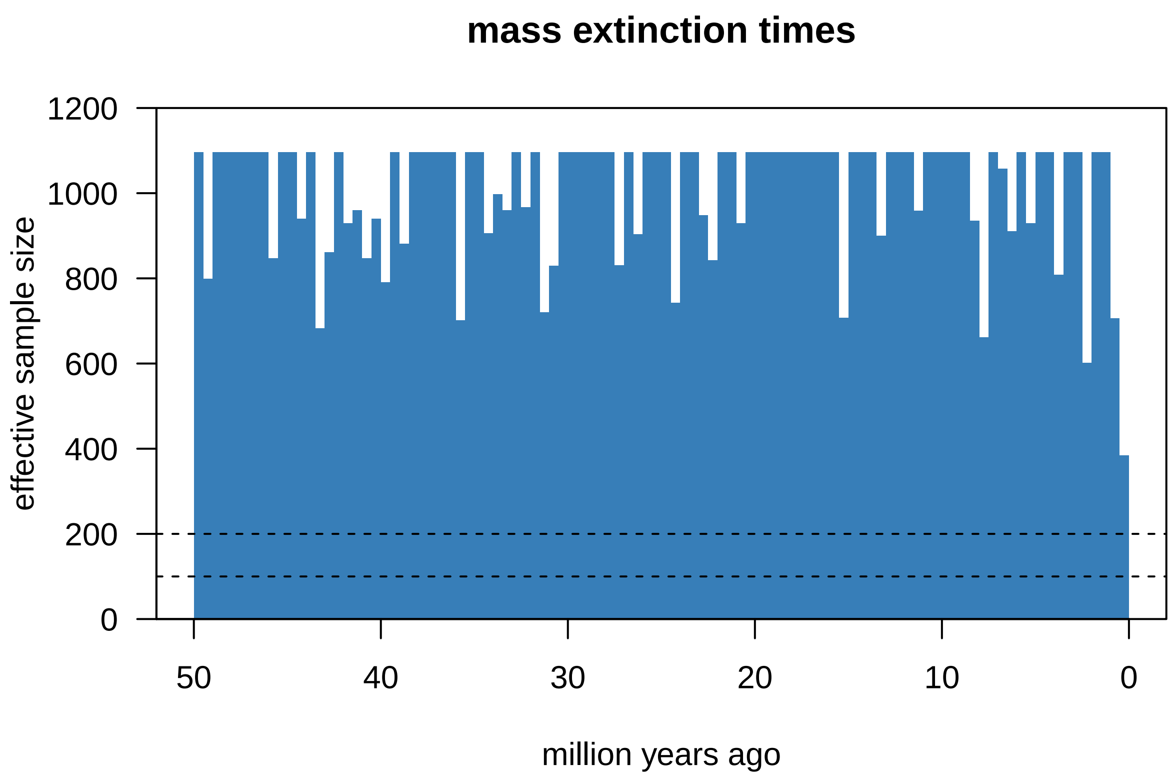

mass extinction times
effective sample size
0
200
400
600
800
1000
1200
50
40
30
20
10
0
million years ago

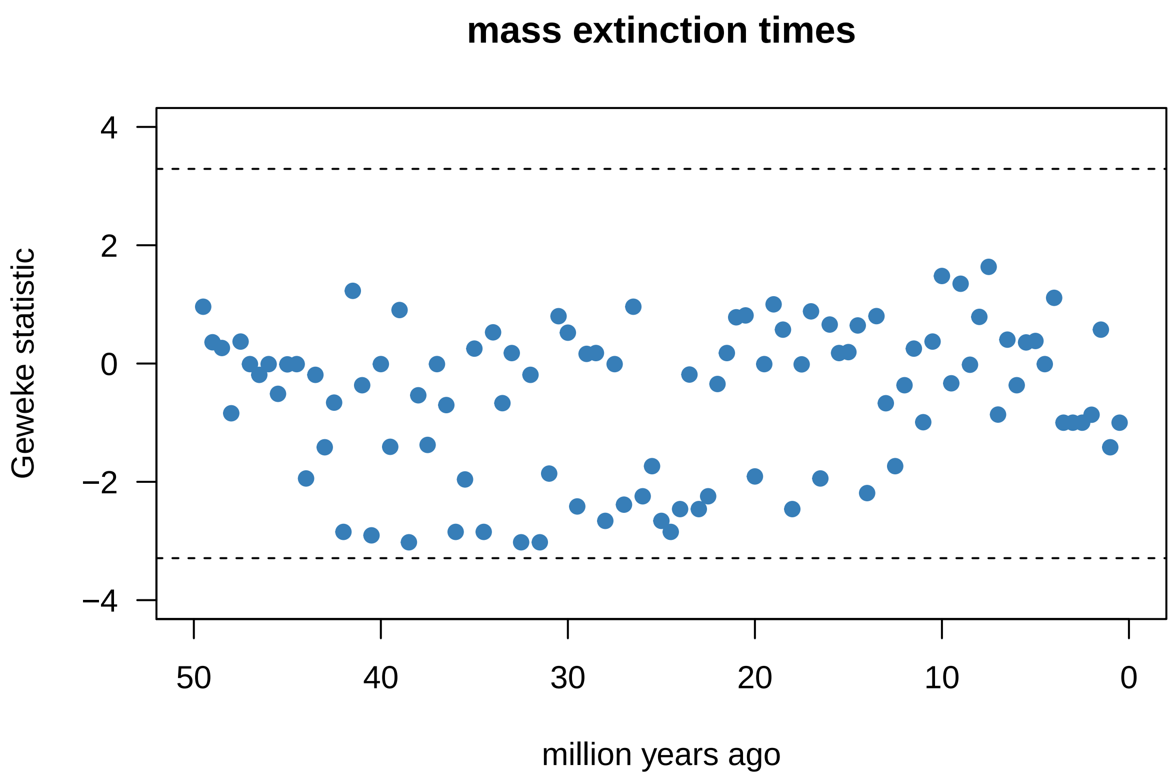


```
tess.plot.output(output_3,fig.types =  c("mass extinction times"),las=2)
```

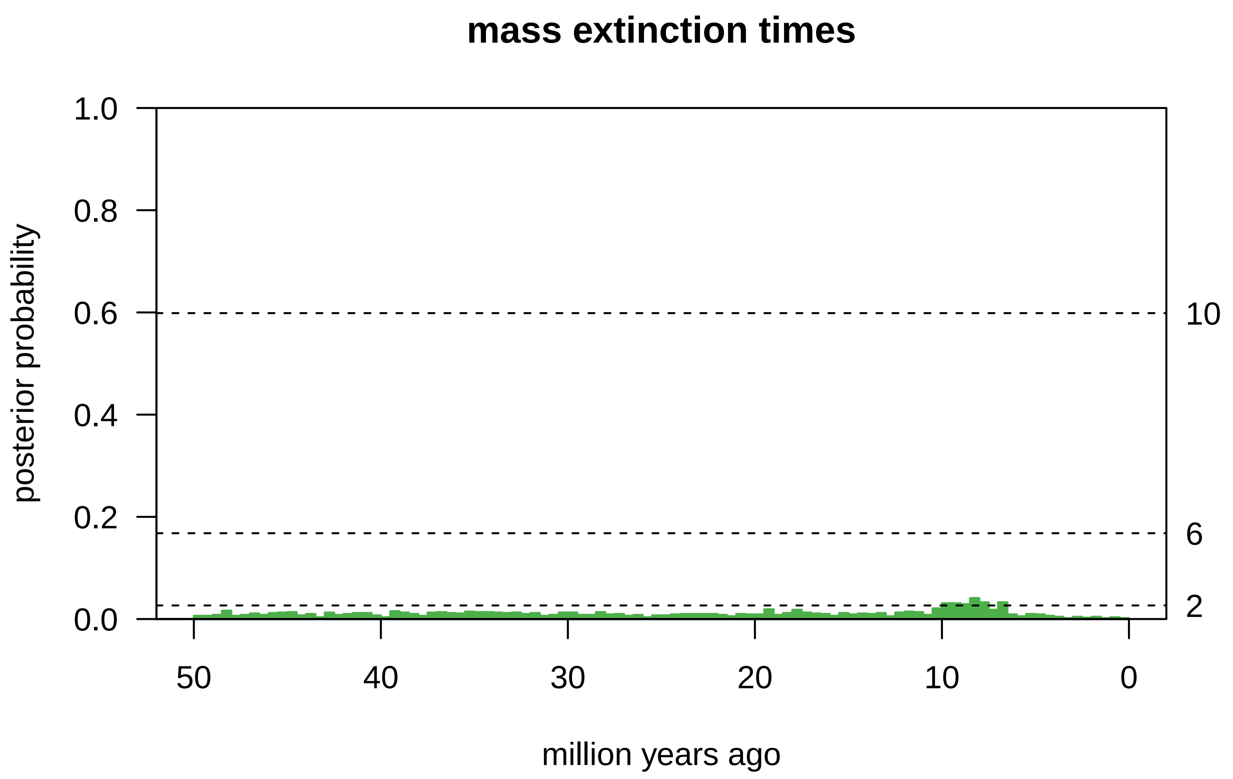


```
op3<-cbind(output_3$`mass extinction Bayes factors`,
      output_3$intervals)
op3
```

```
##                [,1] [,2]
##   [1,] -0.89387791 50.0
##   [2,] -0.89387791 49.5
##   [3,] -0.38757258 49.0
##   [4,]  1.01535005 48.5
##   [5,] -0.89387791 48.0
##   [6,] -0.38757258 47.5
##   [7,]  0.19331897 47.0
##   [8,] -0.38757258 46.5
##   [9,]  0.35525026 46.0
##  [10,]  0.50531378 45.5
##  [11,]  0.64514881 45.0
##  [12,] -0.62497773 44.5
##  [13,]  0.01745205 44.0
##  [14,] -2.01861155 43.5
##  [15,]  0.50531378 43.0
##  [16,] -0.38757258 42.5
##  [17,]  0.01745205 42.0
##  [18,]  0.35525026 41.5
##  [19,]  0.35525026 41.0
##  [20,] -0.62497773 40.5
##  [21,] -2.01861155 40.0
```

```
##  [22,]  0.89917880 39.5
##  [23,]  0.50531378 39.0
##  [24,]  0.01745205 38.5
##  [25,] -0.89387791 38.0
##  [26,]  0.50531378 37.5
##  [27,]  0.64514881 37.0
##  [28,]  0.35525026 36.5
##  [29,]  0.19331897 36.0
##  [30,]  0.77607684 35.5
##  [31,]  0.64514881 35.0
##  [32,]  0.64514881 34.5
##  [33,]  0.50531378 34.0
##  [34,]  0.35525026 33.5
##  [35,]  0.50531378 33.0
##  [36,]  0.01745205 32.5
##  [37,]  0.35525026 32.0
##  [38,] -0.89387791 31.5
##  [39,] -0.38757258 31.0
##  [40,]  0.50531378 30.5
##  [41,]  0.50531378 30.0
##  [42,] -0.38757258 29.5
##  [43,] -0.38757258 29.0
##  [44,]  0.64514881 28.5
##  [45,] -0.17501078 28.0
##  [46,]  0.01745205 27.5
##  [47,] -0.89387791 27.0
##  [48,] -0.38757258 26.5
##  [49,] -2.01861155 26.0
##  [50,] -0.62497773 25.5
##  [51,] -0.62497773 25.0
##  [52,] -0.17501078 24.5
##  [53,]  0.01745205 24.0
##  [54,]  0.01745205 23.5
##  [55,]  0.01745205 23.0
##  [56,]  0.01745205 22.5
##  [57,] -0.38757258 22.0
##  [58,] -1.20401497 21.5
##  [59,]  0.01745205 21.0
##  [60,] -0.17501078 20.5
##  [61,] -0.17501078 20.0
##  [62,]  1.32922503 19.5
##  [63,] -0.38757258 19.0
##  [64,]  0.35525026 18.5
##  [65,]  1.22978510 18.0
##  [66,]  0.50531378 17.5
##  [67,]  0.19331897 17.0
##  [68,]  0.01745205 16.5
##  [69,] -0.89387791 16.0
##  [70,]  0.35525026 15.5
##  [71,] -0.17501078 15.0
##  [72,]  0.19331897 14.5
##  [73,]  0.01745205 14.0
##  [74,]  0.35525026 13.5
##  [75,] -1.20401497 13.0
```

```
## [76,]  0.50531378 12.5
## [77,]  0.77607684 12.0
## [78,]  0.64514881 11.5
## [79,] -0.38757258 11.0
## [80,]  1.51489299 10.5
## [81,]  2.31723468 10.0
## [82,]  2.31723468  9.5
## [83,]  2.19222250  9.0
## [84,]  2.89866228  8.5
## [85,]  2.43532154  8.0
## [86,]  1.22978510  7.5
## [87,]  2.43532154  7.0
## [88,] -0.17501078  6.5
## [89,] -1.20401497  6.0
## [90,]  0.01745205  5.5
## [91,] -0.17501078  5.0
## [92,] -0.89387791  4.5
## [93,] -1.57049211  4.0
## [94,] -3.40856557  3.5
## [95,] -1.57049211  3.0
## [96,] -2.59580636  2.5
## [97,] -1.57049211  2.0
## [98,] -3.40856557  1.5
## [99,] -2.01861155  1.0
## [100,] -3.40856557  0.5
## [101,] -0.89387791  0.0
```

Relaxing the magnitude of extinction places the evidence for extinction more towards 9 Ma than 34 Ma.

Now plot all tests together.

```
par(mar=c(5.5,4.1,1,2))
layout(matrix(c(1,1,2,3,4,5), 3, 2, byrow = TRUE))


plot.new()


plot.window(xlim = c(-50,18), ylim = c(533,543))


polygon(c(-31.5, -31.5,-40.5,-40.5), c(533,543,543,533), col = "#DDDDDD", border = "#DDDDDD")
polygon(c(-43.5, -43.5,-45.5,-45.5), c(533,543,543,533), col = "#DDDDDD", border = "#DDDDDD")

points(AICc~year_of_ex, data=res[1:c(nrow(res)-1),], pch=19, col="black")
aicc.table3$year_of_ex<-c(10, 8, 6, 4, 2)
points(AICc~year_of_ex, data=aicc.table3, pch=19, col="gray80")
aicc.table$year_of_ex<-c(12, 14, 16, 18)
points(AICc~year_of_ex, data=aicc.table, pch=1, col="black")

axis(at = c(-50, -40, -30, -20, -10, 0), side = 1)
xlabelspositions<-seq(from=2, to=18, by=2)
text(x=xlabelspositions, y=par()$usr[3]-0.125,labels=xlabels, srt=90, adj=1, xpd=TRUE)
text(x=xlabelspositions[2], y=par()$usr[3]-0.125,labels=expression(paste(Delta," Temp. (Exp)")),
     srt=90, adj=1, xpd=TRUE)
```

```r
text(x=xlabelspositions[3], y=par()$usr[3]-0.125,labels=expression(paste(Delta," Temp. (Lin)")),
     srt=90, adj=1, xpd=TRUE)

####r right here tight now
axis(at = round(c(-1, axTicks(2)),2), las = 1, side = 2)
mtext(side = 1, text = "Time",line = 3)
mtext(side = 2, text ="AICc", line = 3)
text(-47.5,542,labels="a")

abline(h=min(res$AICc), lty=2)
abline(h=min(res$AICc)+4, lty=2)


##############
# B
 #Akaike Weights
plot.new()
plot.window(xlim = c(-50,0), ylim = c(0,0.2))
points(AkaikeWeights~year_of_ex, data=res[1:c(nrow(res)-1),], pch=19, col="black",
     ylim=c(0,0.2))
axis(at = c(round(1.2 * 50), axTicks(1)), side = 1)
axis(at = round(c(-1, axTicks(2)),2), las = 1, side = 2)
mtext(side = 1, text = "Time",line = 3)
mtext(side = 2, text ="Akaike Weights", line = 3)
text(-45,0.2,labels="b")


######################
#C
plot.new()
plot.window(xlim = range((plotAt-100)/2), ylim = c(0,10))
polygon(c(-31, -31,-36.5,-36.5), c(-1,9,9,-1), col = "#DDDDDD", border = "#DDDDDD")

points(x = c(plotAt[-1] - diff(plotAt[1:2])/2-100)/2, y = thisOutput,
       col = "black",pch=19)
#points(x = c(plotAt[-1] - diff(plotAt[1:2])/2-100)/2, y = output_2$`mass extinction Bayes factors`,
#       col = "gray80",pch=19)
#points(x = c(plotAt[-1] - diff(plotAt[1:2])/2-100)/2, y = output_3$`mass extinction Bayes factors`,
#       col = "black",pch=1)

#legend(x=c(-30,-20), y=c(7,10), col=c("black", "grey80", "black"), pch=c(19, 19, 1),
#       legend = c("95% (91-99%)", "75% (50-93%)", "25% (6-52%)"),
#       cex=1,yjust=-5,
#       x.intersp = 0.25, y.intersp = 0.2, inset = -1, box.lty = 0)

abline(h = 2 * log(output$criticalBayesFactors[1:2]), lty = 2)
axis(at = c(round(1.2 * 50), axTicks(1)), side = 1)
axis(at = round(c(-1, axTicks(2)),2), las = 1, side = 2)
mtext(side = 1, text = "Time",line = 3)
mtext(side = 2, text = "Bayes Factors", line = 3)
text(-45,10,labels="c")
text(-25, 10, "95% (91-99%) Extinction", font=1, cex=1)
```

```
#########
# D
plot.new()
plot.window(xlim = range((plotAt-100)/2), ylim = c(0,10))
#points(x = c(plotAt[-1] - diff(plotAt[1:2])/2-100)/2, y = thisOutput,
#       col = "black",pch=19)
polygon(c(-9.5, -9.5,-8,-8), c(-1,9,9,-1), col = "#DDDDDD", border = "#DDDDDD")
polygon(c(-31, -31,-33.5,-33.5), c(-1,9,9,-1), col = "#DDDDDD", border = "#DDDDDD")
polygon(c(-34, -34,-34.5,-34.5), c(-1,9,9,-1), col = "#DDDDDD", border = "#DDDDDD")
polygon(c(-35, -35,-35.5,-35.5), c(-1,9,9,-1), col = "#DDDDDD", border = "#DDDDDD")
polygon(c(-36, -36,-36.5,-36.5), c(-1,9,9,-1), col = "#DDDDDD", border = "#DDDDDD")
polygon(c(-37, -37,-37.5,-37.5), c(-1,9,9,-1), col = "#DDDDDD", border = "#DDDDDD")

points(x = c(plotAt[-1] - diff(plotAt[1:2])/2-100)/2, y = output_2$`mass extinction Bayes factors`,
       col = "black",pch=19)
#points(x = c(plotAt[-1] - diff(plotAt[1:2])/2-100)/2, y = output_3$`mass extinction Bayes factors`,
#       col = "black",pch=1)

#legend(x=c(-30,-20), y=c(7,10), col=c("black", "grey80", "black"), pch=c(19, 19, 1),
#       legend = c("95% (91-99%)", "75% (50-93%)", "25% (6-52%)"),
#       cex=1,yjust=-5,
#       x.intersp = 0.25, y.intersp = 0.2, inset = -1, box.lty = 0)

abline(h = 2 * log(output_2$criticalBayesFactors[1:2]), lty = 2)
axis(at = c(round(1.2 * 50), axTicks(1)), side = 1)
axis(at = round(c(-1, axTicks(2)),2), las = 1, side = 2)
mtext(side = 1, text = "Time",line = 3)
mtext(side = 2, text = "Bayes Factors", line = 3)
text(-45,10,labels="d")
text(-25, 10, "75% (50-93%) Extinction")

#########
# E
plot.new()
plot.window(xlim = range((plotAt-100)/2), ylim = c(0,10))
#points(x = c(plotAt[-1] - diff(plotAt[1:2])/2-100)/2, y = thisOutput,
#       col = "black",pch=19)
#points(x = c(plotAt[-1] - diff(plotAt[1:2])/2-100)/2, y = output_2$`mass extinction Bayes factors`,
#       col = "black",pch=19)
polygon(c(-7.5, -7.5,-10,-10), c(-1,9,9,-1), col = "#DDDDDD", border = "#DDDDDD")
polygon(c(-6.5, -6.5,-7,-7), c(-1,9,9,-1), col = "#DDDDDD", border = "#DDDDDD")

points(x = c(plotAt[-1] - diff(plotAt[1:2])/2-100)/2, y = output_3$`mass extinction Bayes factors`,
       col = "black",pch=19)

#legend(x=c(-30,-20), y=c(7,10), col=c("black", "grey80", "black"), pch=c(19, 19, 1),
#       legend = c("95% (91-99%)", "75% (50-93%)", "25% (6-52%)"),
#       cex=1,yjust=-5,
#       x.intersp = 0.25, y.intersp = 0.2, inset = -1, box.lty = 0)

abline(h = 2 * log(output_3$criticalBayesFactors[1:2]), lty = 2)
axis(at = c(round(1.2 * 50), axTicks(1)), side = 1)
axis(at = round(c(-1, axTicks(2)),2), las = 1, side = 2)
```

```
mtext(side = 1, text = "Time",line = 3)
mtext(side = 2, text = "Bayes Factors", line = 3)
text(-45,10,labels="e")
text(-25, 10, "25% (6-52%) Extinction")
```

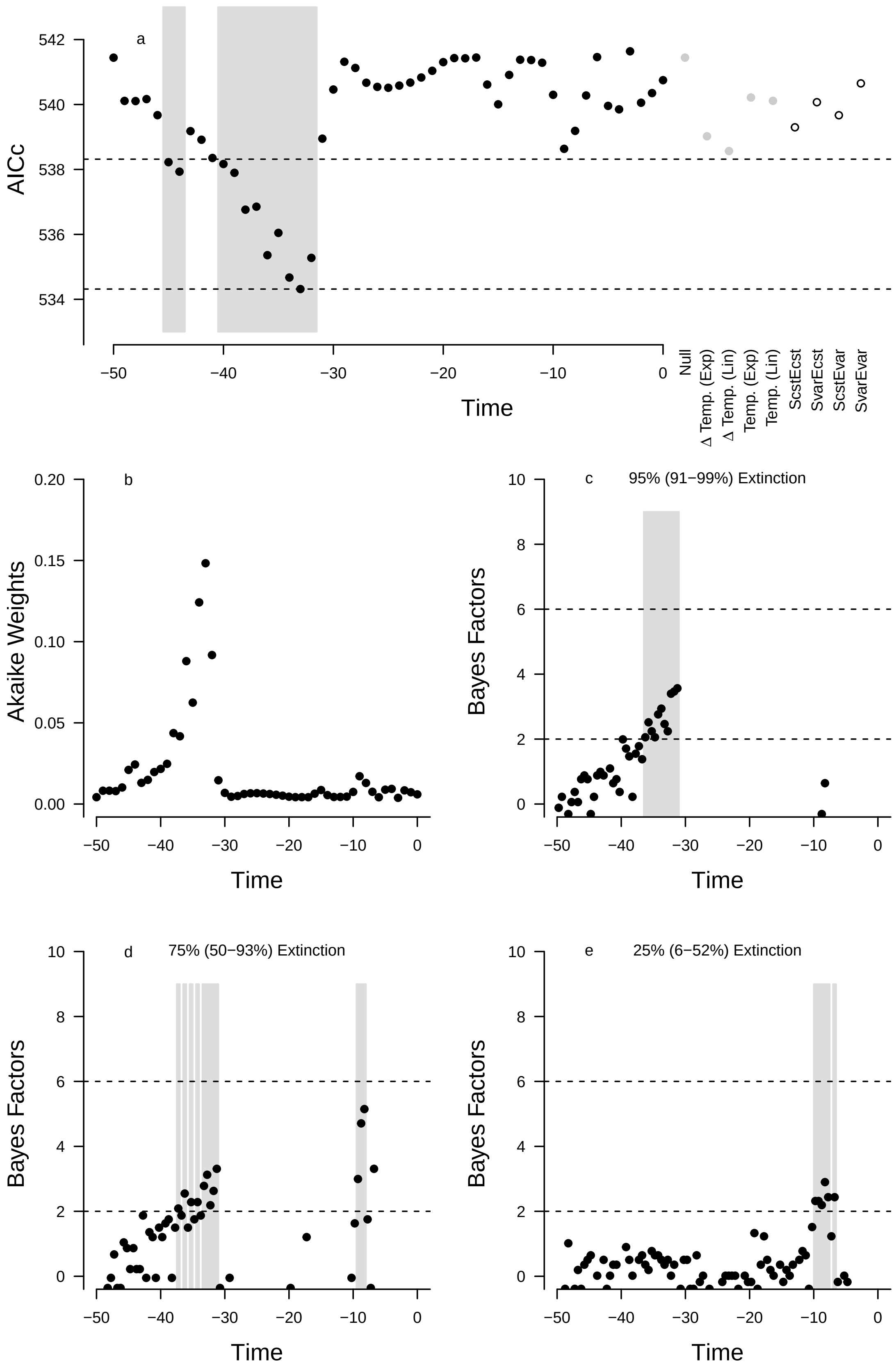

a
AICc
542
540
538
536
534
−50
−40
−30
−20
−10
0
Time
Null
Δ Temp. (Exp)
Δ Temp. (Lin)
Temp. (Exp)
Temp. (Lin)
ScstEcst
SvarEcst
ScstEvar
SvarEvar
b
Akaike Weights
0.20
0.15
0.10
0.05
0.00
Time
c
95% (91−99%) Extinction
Bayes Factors
Time
d
75% (50−93%) Extinction
Bayes Factors
Time
e
25% (6−52%) Extinction
Bayes Factors
Time
