# Supplementary material for: Mid-Cenozoic climate change, extinction, and faunal turnover in Madagascar, and their bearing on the evolution of lemurs
Source: BMC Evol Biol. 2020 Aug 8;20:97. doi: 10.1186/s12862-020-01628-1 (PMC7414565; doi:10.1186/s12862-020-01628-1)
Supplement: Supplementary file 4 — Additional file 4. Supplementary R Code and Output (Based on Herrera, 2017). R Code for modeling lemur diversification using phylogeny from Herrera. [file 12862_2020_1628_MOESM4_ESM.pdf]

BMC Evolutionary Biology
Mid-Cenozoic climate change, extinction, and faunal turnover in Madagascar, and their bearing on the evolution of lemurs

Laurie R. Godfrey, Karen E. Samonds, Justin W. Baldwin,
Michael R. Sutherland, Jason M. Kamilar, Kristen L. Allfisher

Additional file 4: Supplementary R Code and Output (Based on Herrera, 2017)

R Code for modeling lemur diversification using phylogeny from Herrera

This document shows all necessary code to replicate our findings, using the evolutionary tree provided by Herrera (2017) from the supplementary file on Figshare (Word Document page 62-63, referred to in his text as File S1).

Herrera, James P. (2017): Supplementary material from "Testing the adaptive radiation hypothesis for the lemurs of Madagascar". The Royal Society. Collection. https://doi.org/10.6084/m9.figshare.c.3655697.v1

We loaded the file S1, pruned it to include only the taxa we considered in our original tree as shown in Appendix S3 and reran all analyses from appendix S3 considering the alternative tree provided herein.

```
library(BAMMtools)
library(coda)
library(mgcv)
library(phangorn)
library(phytools)
library(RPANDA)
library(scales)
library(TESS)
library(TreePar)
library(xtable)
```

1. Generate Phylogeny and Retrieve Temperature Data

Methods for phylogeny creation are explained in text.

```
lemurs <- read.tree("sub3/herrera2017_tree_S1_pruned.tre")
plot.phylo(lemurs,cex = 0.6)
axisPhylo()
```

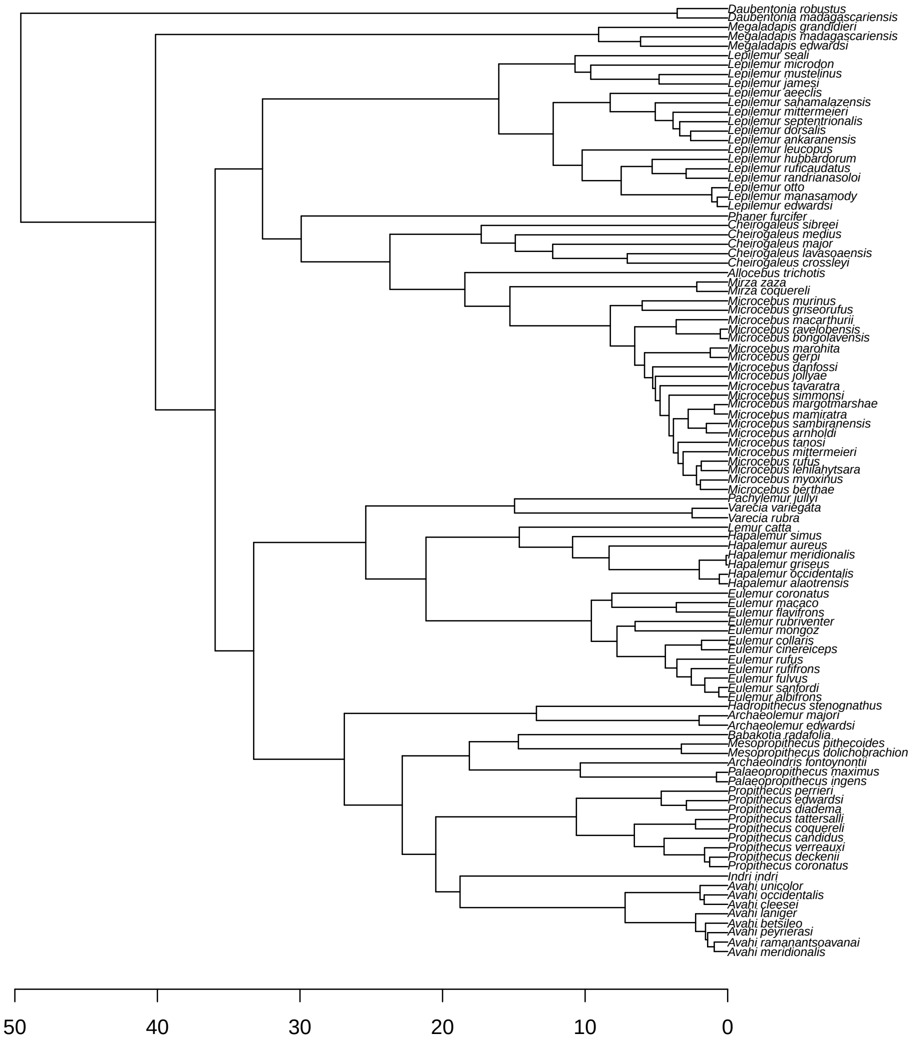

Now we will use the global temperature database from RPANDA (Zachos et al. 2008; Condamine et al. 2013).

```
data(InfTemp)
plot(InfTemp$Temperature~InfTemp$Age)
```

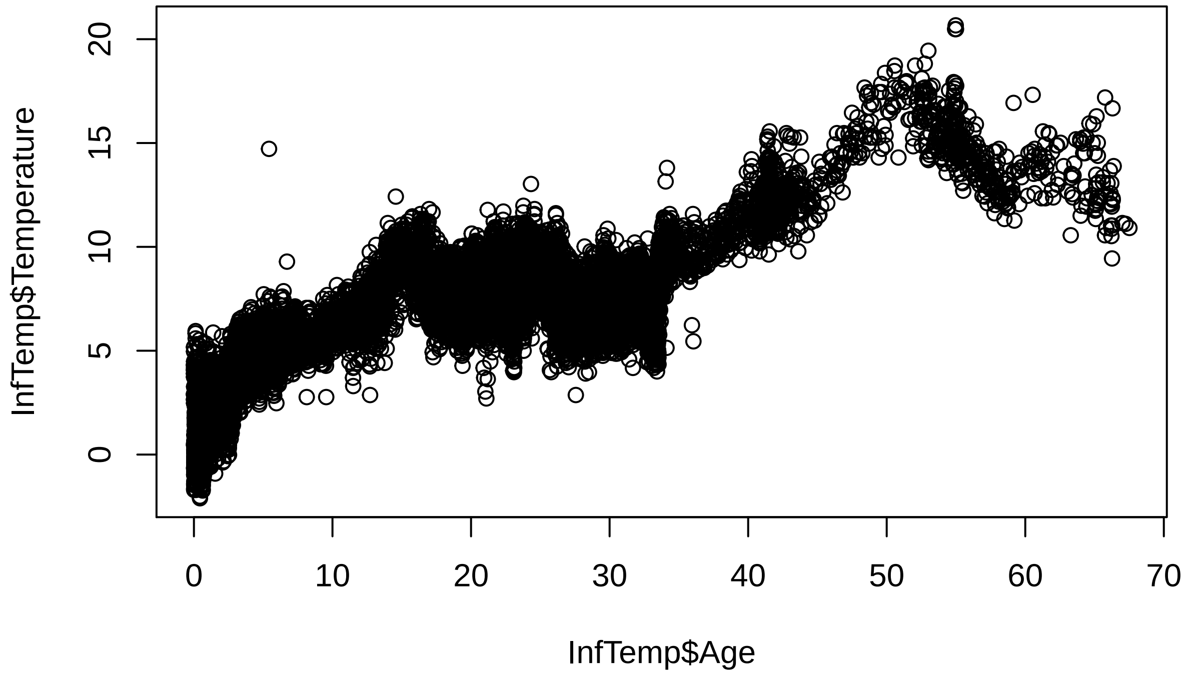

We use a generalized additive model (GAM) with 300 knots to predict the average temperature between 50 and 0 Ma. Black are the raw temperatures, red is the predicted temperature from the GAM.

```
InfTemp2=subset(InfTemp, InfTemp$Age<50)
set.seed(1)
G1 <- gam(Temperature ~ s(Age, k = 300, bs = "cs"), data = InfTemp2)
newdata=data.frame("Age"=seq(0, 70 ,length.out = 700))
newdata$predY <- predict(G1, type = "response", newdata = newdata)
InfTemp2$Nage=-InfTemp2$Age
newdata$Nage=-newdata$Age
plot(InfTemp2$Temperature~InfTemp2$Nage, xlab="Time",
     ylab=expression("Temperature ("~ degree~"C)"),
     axes=F, col=alpha("black", 0.35),ylim=c(-5,20))
axis(side=1)
axis(side=2)
lines(newdata$Nage,newdata$predY,col="red")
```

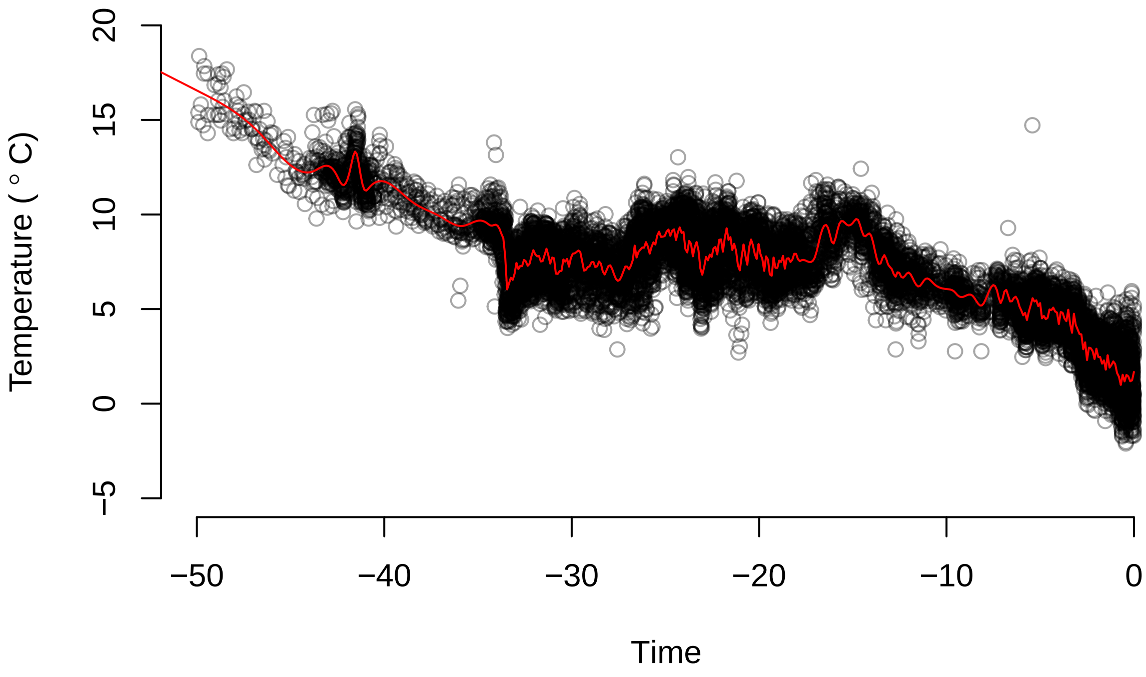

We also consider the rate of change of predicted temperature.

```
newdata$predY_diff <- c(0,diff(newdata$predY)/diff(newdata$Nage))
plot(predY_diff~Nage, data=newdata,type="l",xlim=c(-50,0), axes=F,ylim=c(-20,10),
     ylab = expression(Delta*degree ~ C ~ "/ Million Years"),
     xlab = "Time")
axis(side=1)
axis(side=2)
```

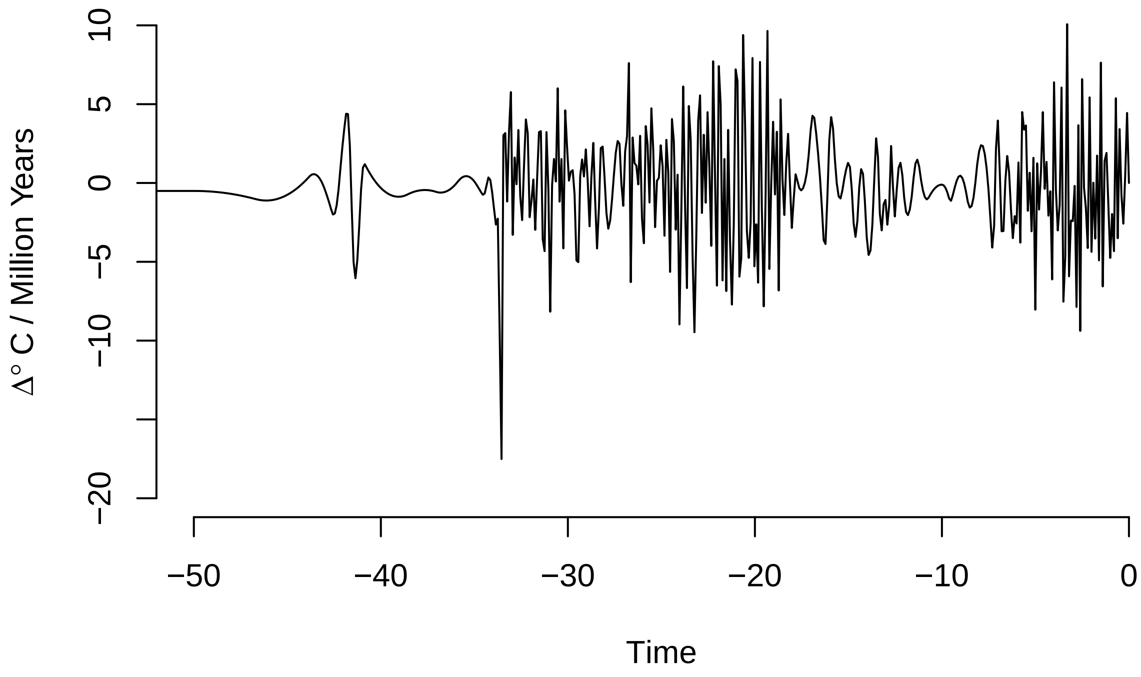

2. Estimate Diversification Rates

2.1. TreePar

We first used TreePar to estimate diversification rates over time.

```
res.shifts <- bd.shifts.optim(x,sampling=c(1,1),ME=TRUE, grid=1, start=0,end = 50)
saveRDS(res.shifts, "sub3/h1/res.shifts.rds")
```

```
x<-sort(getx(lemurs),decreasing=TRUE)
treepar.df <- data.frame()
for(i in 1:50){treepar.df <- rbind(treepar.df,
  res.shifts[[1]][[2]][[i]][[1]]$par
)}
names(treepar.df) <- c("turnover", "diversification", "probsurvive")
treepar.df$time_mya <- -1:-50
plot(diversification~time_mya, data=treepar.df, type="l", main="",
     xlab="Time", ylim=c(-0.1,0.1),
     ylab="Diversification Rate",axes=F,lwd=3)
axis(side=1)
axis(side=2)
abline(h=0, col="black", lty=2)
```

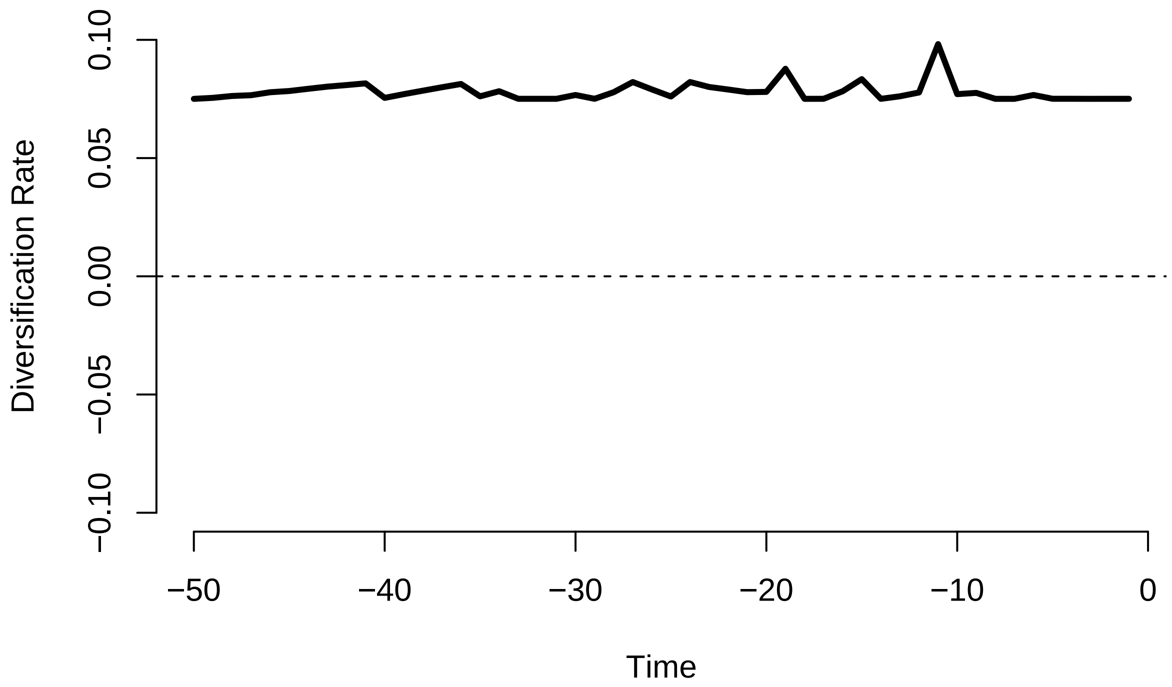

Below we report diversification rates at the beginning and end of the series.

```
fintab=data.frame(rbind(cbind(head(-treepar.df$time_mya,1),
                              head(treepar.df$diversification,1)),
             cbind(tail(-treepar.df$time_mya,1),
                   tail(treepar.df$diversification,1))))
names(fintab)=c("time", "div.rate")
fintab
```

```
##   time   div.rate
## 1    1 0.07506759
## 2   50 0.07504958
```

We also estimate survival rates over time.

```
plot(probsurvive~time_mya, data=treepar.df, type="l", main="",
     xlab="Time", ylim=c(0,1),
     ylab="P(Survival)",axes=F, lwd=3)
axis(side=1)
axis(side=2)
```

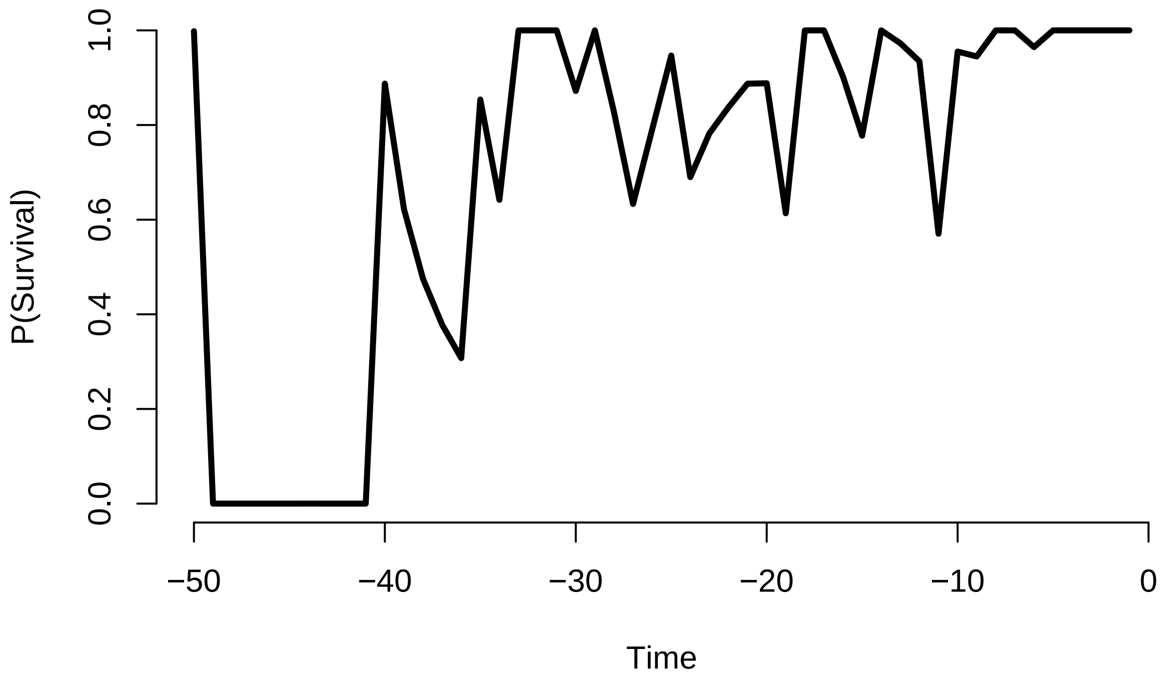

2.2. RPANDA

Morlon et al.'s package fits time-dependent diversification models that ignore enviromental variables. There are 4 choices: time-invariant speciation and extinction rates, time-invariance of either speciation or extinction rates and variable speciation and extinction rates. In all cases, we must provide the fraction of species of the clade that are included in the phylogeny to account for incomplete taxon sampling. The fraction we use is `0.8890909`, stemming from averaging the fractions Herrera 2017 reported in the supplementary material page 2, Table S1 (second column, for all italicized rows).

```
f=0.8890909
lambda.cst <- function(x,y){y}
lambda.var <- function(x,y){y[1]*exp(y[2]*x)}
mu.cst <- function(x,y){y}
mu.var <- function(x,y){y[1]*exp(y[2]*x)}
fit.multi.rpanda <- function(tree,par)
    {
        bcstdcst <- fit_bd(tree, max(branching.times(tree)), f.lamb=lambda.cst, f.mu=mu.cst,
                           lamb_par=par[[1]][1],mu_par=par[[1]][2],
                           cst.lamb=TRUE,cst.mu=TRUE,cond="crown",f=f,dt=1e-3)
        bvardcst <- fit_bd(tree, max(branching.times(tree)), f.lamb=lambda.var, f.mu=mu.cst,
                           lamb_par=par[[2]][c(1,2)],mu_par=par[[2]][3],expo.lamb=TRUE,
                           cst.mu=TRUE,cond="crown",f=f,dt=1e-3)
        bcstdvar <- fit_bd(tree, max(branching.times(tree)), f.lamb=lambda.cst, f.mu=mu.var,
                           lamb_par=par[[3]][1],mu_par=par[[3]][c(2,3)],
                           cst.lamb=TRUE,expo.mu=TRUE,cond="crown",f=f,dt=1e-3)
```

```
        bvardvar <- fit_bd(tree, max(branching.times(tree)), f.lamb=lambda.var, f.mu=mu.var,
                           lamb_par=par[[4]][c(1,2)],mu_par=par[[4]][c(3,4)],expo.lamb=TRUE,
                           expo.mu=TRUE,cond="crown",f=f,dt=1e-3)
        return(list("bcstdcst"=bcstdcst,"bvardcst"=bvardcst,
                    "bcstdvar"=bcstdvar,"bvardvar"=bvardvar))
}
lemurs.par <- list(c(0.4,0),c(0.4,-0.05,0),c(0.4,0.1,0.05),c(0.4,-0.05,0.1,0.05))
results <- list("lemurs.res"=fit.multi.rpanda(lemurs,lemurs.par))
aicc.table <- matrix(nrow=4,ncol=1,NA)
for(i in 1:1)
    {
        for(j in 1:4)
            {
                aicc.table[j,i] <- results[[i]][[j]]$aicc
            }
}
aicc.table <- data.frame("AICc"=c(aicc.table))
aicc.table$'Speciation Rate' <- c("Constant","Time-varying","Constant","Time-varying")
aicc.table$'Extinction Rate' <- c("Constant","Constant","Time-varying","Time-varying")

par.table <- data.frame(
  "ConstantSpeciation_ConstantExtinction"=c(results[[1]]$bcstdcst$lamb_par[1:2],
                                             results[[1]]$bcstdcst$mu_par[1:2]),
  "VaryingSpeciation_ConstantExtinction"=c(results[[1]]$bvardcst$lamb_par[1:2],
                                             results[[1]]$bvardcst$mu_par[1:2]),
  "ConstantSpeciation_VaryingExtinction"=c(results[[1]]$bcstdvar$lamb_par[1:2],
                                             results[[1]]$bcstdvar$mu_par[1:2]),
  "VaryingSpeciation_VaryingExtinction"=c(results[[1]]$bvardvar$lamb_par[1:2],
                                             results[[1]]$bvardvar$mu_par[1:2]))
row.names(par.table) <- c("$\\lambda_1$","$\\lambda_2$","$\\mu_1$","$\\mu_2$")
aicc.table <- cbind(aicc.table, t(par.table))
aicc.table <- aicc.table[,c(2,3,1,4:7)]
```

Speciation Rate	Extinction Rate	AICc	λ_1	λ_2	μ_1	μ_2
Constant	Constant	642.71	0.16		0.08	
Time-varying	Constant	643.82	0.14	-0.03	-0.00	
Constant	Time-varying	643.90	0.15		0.04	0.03
Time-varying	Time-varying	645.99	0.14	-0.03	-0.00	-0.12

The models are characterized by the following functions. $\mu(.)$ describes the extinction rate function and $\lambda(.)$ describes the speciation rate function. Depending on the choice of model, the functions are parameterized by either one or two parameters. The table below shows the estimated parameter values for each model and its Akaike Information Criterion with finite size correction (AICc).

$$\lambda_{constant}(\lambda_1) = \lambda_1$$

$$\lambda_{variable}(x, \lambda_1, \lambda_2) = \lambda_1 * e^{x\lambda_2}$$

$$\mu_{constant}(\mu_1) = \mu_1$$

$$\mu_{variable}(x, \mu_1, \mu_2) = \mu_1 * e^{x\mu_2}$$

The four model predictions are shown below. Allowing extinction rate to vary over time allows for high extinction rates before 30 Ma (red and green lines). The strongest increase in diversification rate is observed

for the model in which both rates are allowed to vary over time (green) with a rapid change in slope near 30 Ma. Allowing only the extinction rate to vary over time (red) shows a weaker increase in diversification rate. It is important to note that these models are all supported equally well/poorly by the data, according to AICc scores (above: they are all within 2 scores of each other).

```
div.time <- c(max(branching.times(lemurs)))
tot_time <- max(branching.times(lemurs))
t <- seq(tot_time-div.time, tot_time, 0.01)
r <- function(fit.bd, t) {fit.bd$f.lamb(t) - fit.bd$f.mu(t)}

plot(-t, r(results$lemurs.res$bcstdcst, t), type = "l", xlab = "Time",
     ylab = "Diversification Rate", ylim=c(-1, 0.2),
             main = "", col="blue",axes=F,lwd=3)
axis(side=1)
axis(side=2)
lines(-t, r(results$lemurs.res$bvardcst, t), col="black",lwd=3)
lines(-t, r(results$lemurs.res$bcstdvar, t),  col="red",lwd=3)
lines(-t, r(results$lemurs.res$bvardvar, t),  col="green",lwd=3)
abline(h=0, col="black", lty=2)
legend(-25,-0.2, c("ScstEcst","SvarEcst","ScstEvar","SvarEvar"),
       lty=c(1,1,1,1),lwd=c(3,3,3,3),col=c("blue","black","red","green"))
```

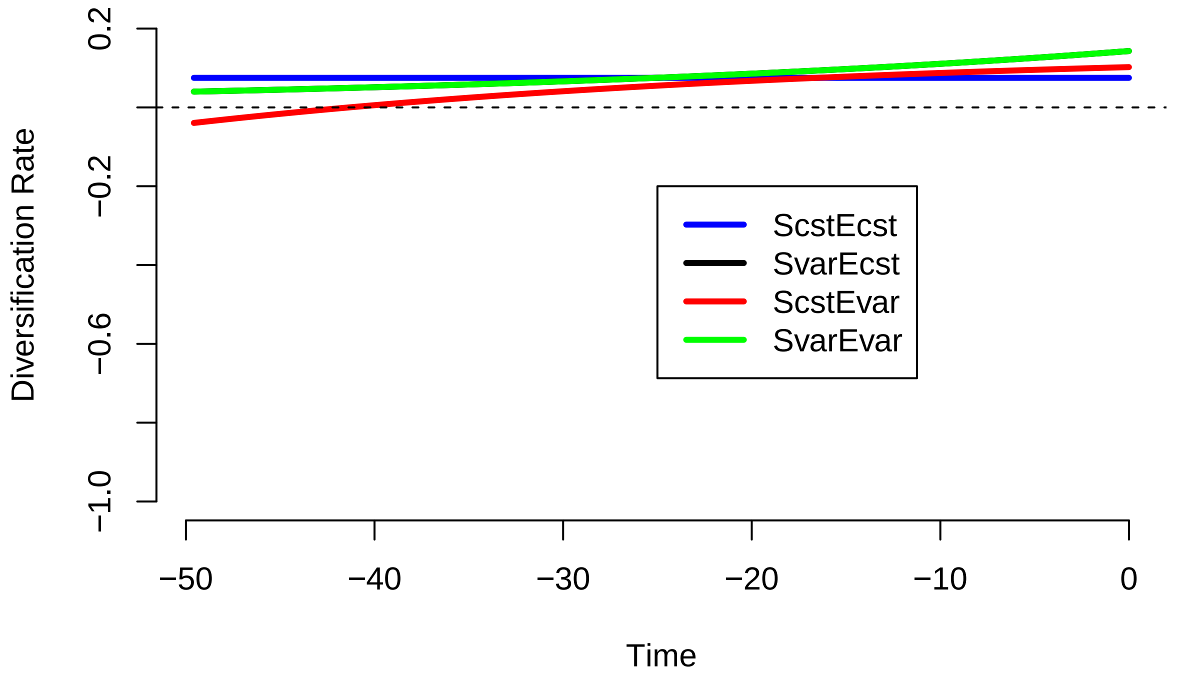

Again we report diversification rates at the beginning and end of the series.

```
fintab=data.frame(rbind(
                  cbind(rbind(cbind(head(-t,1),head(r(results$lemurs.res$bcstdcst, t),1)),
                  cbind(tail(-t,1),tail(r(results$lemurs.res$bcstdcst, t),1))),
```

```r
                    rep("black",2)),
                    cbind(rbind(cbind(head(-t,1),head(r(results$lemurs.res$bvardcst, t),1)),
                    cbind(tail(-t,1),tail(r(results$lemurs.res$bvardcst, t),1))),
                    rep("blue",2)),
                    cbind(rbind(cbind(head(-t,1),head(r(results$lemurs.res$bcstdvar, t),1)),
                    cbind(tail(-t,1),tail(r(results$lemurs.res$bcstdvar, t),1))),
                    rep("red",2)),
                    cbind(rbind(cbind(head(-t,1),head(r(results$lemurs.res$bvardvar, t),1)),
                    cbind(tail(-t,1),tail(r(results$lemurs.res$bvardvar, t),1))),
                    rep("green",2))
                    )
)
names(fintab)=c("time", "div.rate", "color")
fintab
```

```
##     time             div.rate color
## 1      0    0.075013231071332 black
## 2 -49.58    0.075013231071332 black
## 3      0    0.143018027368821  blue
## 4 -49.58   0.0400405211117664  blue
## 5      0    0.102240166197917   red
## 6 -49.58  -0.0393735426127133   red
## 7      0    0.143052016816767 green
## 8 -49.58   0.0400162746431274 green
```

2.3. BAMM

This is the text of the BAMM control file we used. We use the sampling fraction of 0.8890909 as above (details in text).

NOPE

```
# BAMM configuration file for speciation/extinction analysis
# ==========================================================
#
# Format
# ------
#
#     - Each option is specified as: option_name = option_value
#     - Comments start with # and go to the end of the line
#     - True is specified with "1" and False with "0"


################################################################################
# GENERAL SETUP AND DATA INPUT
################################################################################

modeltype = speciationextinction
# Specify "speciationextinction" or "trait" analysis

treefile = herrera2017_tree_S1_pruned.tre
# File name of the phylogenetic tree to be analyzed

runInfoFilename = run_info.txt
```

```
# File name to output general information about this run

sampleFromPriorOnly = 0
# Whether to perform analysis sampling from prior only (no likelihoods computed)

runMCMC = 1
# Whether to perform the MCMC simulation. If runMCMC = 0, the program will only
# check whether the data file can be read and the initial likelihood computed

simulatePriorShifts = 1
# Whether to simulate the prior distribution of the number of shift events,
# given the hyperprior on the Poisson rate parameter. This is necessary to
# compute Bayes factors

loadEventData = 0
# Whether to load a previous event data file

eventDataInfile = event_data_in.txt
# File name of the event data file to load, used only if loadEventData = 1

initializeModel = 1
# Whether to initialize (but not run) the MCMC. If initializeModel = 0, the
# program will only ensure that the data files (e.g., treefile) can be read

useGlobalSamplingProbability = 1
# Whether to use a "global" sampling probability. If False (0), expects a file
# name for species-specific sampling probabilities (see sampleProbsFilename)

globalSamplingFraction = 0.8890909
# The sampling probability. If useGlobalSamplingFraction = 0, this is ignored
# and BAMM looks for a file name with species-specific sampling fractions

sampleProbsFilename = sample_probs.txt
# File name containing species-specific sampling fractions

# seed = -1
# Seed for the random number generator.
# If not specified (or is -1), a seed is obtained from the system clock

overwrite = 1
# If True (1), the program will overwrite any output files in the current
# directory (if present)


################################################################################
# PRIORS
################################################################################

expectedNumberOfShifts = 1.0
# prior on the number of shifts in diversification
# Suggested values:
#     expectedNumberOfShifts = 1.0 for small trees (< 500 tips)
#     expectedNumberOfShifts = 10 or even 50 for large trees (> 5000 tips)
```

```
# Prior block chosen by BAMMtools::setBAMMpriors

lambdaInitPrior = 2.63470649014838
# Prior (rate parameter of exponential) on the initial lambda value for rate
# regimes

lambdaShiftPrior = 0.0230258509299405
# Prior (std dev of normal) on lambda shift parameter for rate regimes
# You cannot adjust the mean of this distribution (fixed at zero, which is
# equal to a constant rate diversification process)

muInitPrior = 2.63470649014838
# Prior (rate parameter of exponential) on extinction rates

lambdaIsTimeVariablePrior = 1
# Prior (probability) of the time mode being time-variable (vs. time-constant)


################################################################################
# MCMC SIMULATION SETTINGS & OUTPUT OPTIONS
################################################################################

numberOfGenerations = 10000000
# Number of generations to perform MCMC simulation

mcmcOutfile = mcmc_out.txt
# File name for the MCMC output, which only includes summary information about
# MCMC simulation (e.g., log-likelihoods, log-prior, number of processes)

mcmcWriteFreq = 10000
# Frequency in which to write the MCMC output to a file

eventDataOutfile = event_data.txt
# The raw event data (these are the main results). ALL of the results are
# contained in this file, and all branch-specific speciation rates, shift
# positions, marginal distributions etc can be reconstructed from this output.
# See R package BAMMtools for working with this output

eventDataWriteFreq = 10000
# Frequency in which to write the event data to a file

printFreq = 10000
# Frequency in which to print MCMC status to the screen

acceptanceResetFreq = 10000
# Frequency in which to reset the acceptance rate calculation
# The acceptance rate is output to both the MCMC data file and the screen

# outName = BAMM
# Optional name that will be prefixed on all output files (separated with "_")
# If commented out, no prefix will be used
```

```
################################################################################
# OPERATORS: MCMC SCALING OPERATORS
################################################################################

updateLambdaInitScale = 2.0
# Scale parameter for updating the initial speciation rate for each process

updateLambdaShiftScale = 0.1
# Scale parameter for the exponential change parameter for speciation

updateMuInitScale = 2.0
# Scale parameter for updating initial extinction rate for each process

updateEventLocationScale = 0.05
# Scale parameter for updating LOCAL moves of events on the tree
# This defines the width of the sliding window proposal

updateEventRateScale = 4.0
# Scale parameter (proportional shrinking/expanding) for updating
# the rate parameter of the Poisson process


################################################################################
# OPERATORS: MCMC MOVE FREQUENCIES
################################################################################

updateRateEventNumber = 0.1
# Relative frequency of MCMC moves that change the number of events

updateRateEventPosition = 1
# Relative frequency of MCMC moves that change the location of an event on the
# tree

updateRateEventRate = 1
# Relative frequency of MCMC moves that change the rate at which events occur

updateRateLambda0 = 1
# Relative frequency of MCMC moves that change the initial speciation rate
# associated with an event

updateRateLambdaShift = 1
# Relative frequency of MCMC moves that change the exponential shift parameter
# of the speciation rate associated with an event

updateRateMu0 = 1
# Relative frequency of MCMC moves that change the extinction rate for a given
# event

updateRateLambdaTimeMode = 0
# Relative frequency of MCMC moves that flip the time mode
# (time-constant <=> time-variable)

localGlobalMoveRatio = 10.0
```

```
# Ratio of local to global moves of events


################################################################################
# INITIAL PARAMETER VALUES
################################################################################

lambdaInit0 = 0.032
# Initial speciation rate (at the root of the tree)

lambdaShift0 = 0
# Initial shift parameter for the root process

muInit0 = 0.005
# Initial value of extinction (at the root)

initialNumberEvents = 0
# Initial number of non-root processes


################################################################################
# METROPOLIS COUPLED MCMC
################################################################################

numberOfChains = 4
# Number of Markov chains to run

deltaT = 0.01
# Temperature increment parameter. This value should be > 0
# The temperature for the i-th chain is computed as 1 / [1 + deltaT * (i - 1)]

swapPeriod = 1000
# Number of generations in which to propose a chain swap

chainSwapFileName = chain_swap.txt
# File name in which to output data about each chain swap proposal.
# The format of each line is [generation],[rank_1],[rank_2],[swap_accepted]
# where [generation] is the generation in which the swap proposal was made,
# [rank_1] and [rank_2] are the chains that were chosen, and [swap_accepted] is
# whether the swap was made. The cold chain has a rank of 1.


################################################################################
# NUMERICAL AND OTHER PARAMETERS
################################################################################

minCladeSizeForShift = 1
# Allows you to constrain location of possible rate-change events to occur
# only on branches with at least this many descendant tips. A value of 1
# allows shifts to occur on all branches.

segLength = 0.02
# Controls the "grain" of the likelihood calculations. Approximates the
```

```
# continuous-time change in diversification rates by breaking each branch into
# a constant-rate diversification segments, with each segment given a length
# determined by segLength. segLength is in units of the root-to-tip distance of
# the tree. So, if the segLength parameter is 0.01, and the crown age of your
# tree is 50, the "step size" of the constant rate approximation will be 0.5.
# If the value is greater than the branch length (e.g., you have a branch of
# length < 0.5 in the preceding example) BAMM will not break the branch into
# segments but use the mean rate across the entire branch.
```

We then inspect the chain for adequate mixing.

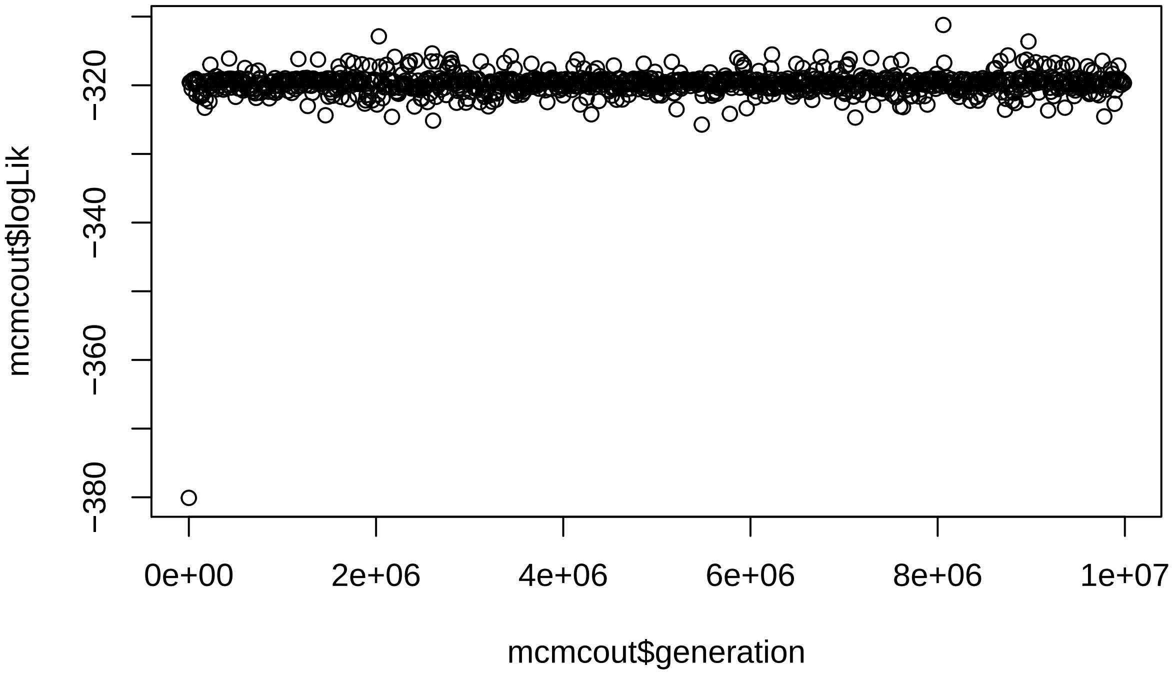

This looks like adequate mixing, so we conclude we have run it for a sufficient number of generations. We then consider the effective sample size.

```
burnstart <- floor(0.1 * nrow(mcmcout))
postburn <- mcmcout[burnstart:nrow(mcmcout), ]
effectiveSize(postburn$N_shifts)
```

```
## var1
##  901
```

```
effectiveSize(postburn$logLik)
```

```
## var1
##  901
```

The effective sample size is well over the recommended cutoff of 200. We conclude that convergence has been achieved. Code below is modified lightly from BAMMtools package functions.

```r
events.lemurs <- read.csv("BAMM runs\\9_19_2018_LemursH1\\event_data.txt")
ed <- getEventData(lemurs, events.lemurs, burnin = 0.1)
```

```
## Processing event data from data.frame
##
## Discarded as burnin: GENERATIONS <  990000
## Analyzing  901  samples from posterior
##
## Setting recursive sequence on tree...
##
## Done with recursive sequence
```

```r
rmat <- getRateThroughTimeMatrix(ed, nslices = 100,
            nodetype = "include")
        rate <- rmat$lambda - rmat$mu
        ratelabel <- "Diversification Rate"
    maxTime <- max(rmat$times)
    nanCol <- apply(rate, 2, function(x) any(is.nan(x)))
    rate <- rate[, which(nanCol == FALSE)]
    rmat$times <- rmat$times[which(nanCol == FALSE)]
    rmat$times <- max(rmat$times) - rmat$times

    #intervals is CI
intervals=c(0.025, 0.975)
    if (!is.null(intervals)) {
        mm <- apply(rate, MARGIN = 2, quantile, intervals)
        poly <- list()
        q1 <- 1
        q2 <- nrow(mm)
        repeat {
            if (q1 >= q2) {
                break
            }
            a <- as.data.frame(cbind(rmat$times, mm[q1, ]))
            b <- as.data.frame(cbind(rmat$times, mm[q2, ]))
            b <- b[rev(rownames(b)), ]
            colnames(a) <- colnames(b) <- c("x", "y")
            poly[[q1]] <- rbind(a, b)
            q1 <- q1 + 1
            q2 <- q2 - 1
        }
    }
    useMedian=TRUE
    avg <- unlist(apply(rate, 2, median))
    smooth=TRUE
    smoothParam = 0.2
    if (smooth) {
        for (i in 1:length(poly)) {
            p <- poly[[i]]
            rows <- nrow(p)
            p[1:rows/2, 2] <- loess(p[1:rows/2, 2] ~ p[1:rows/2,
                1], span = smoothParam)$fitted
            p[(rows/2):rows, 2] <- loess(p[(rows/2):rows, 2] ~
                p[(rows/2):rows, 1], span = smoothParam)$fitted
```

```
            poly[[i]] <- p
        }
        avg <- loess(avg ~ rmat$time, span = smoothParam)$fitted
    }
  ylim=xlim="auto"
        ylim=c(-1,0.2)
  plot.new()
  xMin <- maxTime
                xMax <- 0
                yMin <- ylim[1]
                yMax <- ylim[2]
  plot.window(xlim = c(xMin, xMax), ylim = c(yMin,
                yMax))
            xticks=yticks=NULL
                axis(at = c(round(1.2 * xMin), axTicks(1)), cex.axis = 1,
                  side = 1)
                axis(at = round(c(-1, axTicks(2)),2), cex.axis = 1,
                  las = 1, side = 2)
                axis.labels=TRUE
                xline = 3
                yline = 3
            if (axis.labels) {
                mtext(side = 1, text = "Time",
                  line = xline)
                mtext(side = 2, text = "Diversification Rate", line = yline)
            }
                 intervalCol='gray80'
                opacity=1
        if (!is.null(intervals)) {
            for (i in 1:length(poly)) {
                polygon(x = poly[[i]][, 1], y = poly[[i]][, 2],
                  col = transparentColor(intervalCol, opacity),
                  border = NA)
            }
        }
        lines(x = rmat$time, y = avg, lwd = 3, col = "black")
        abline(h=avg[length(avg)],col="blue",lwd=3)
        legend(25,-0.2, c("Constant Rate","BAMM"),lty=c(1,1),lwd=c(3,3),
               col=c("blue","black"))
```

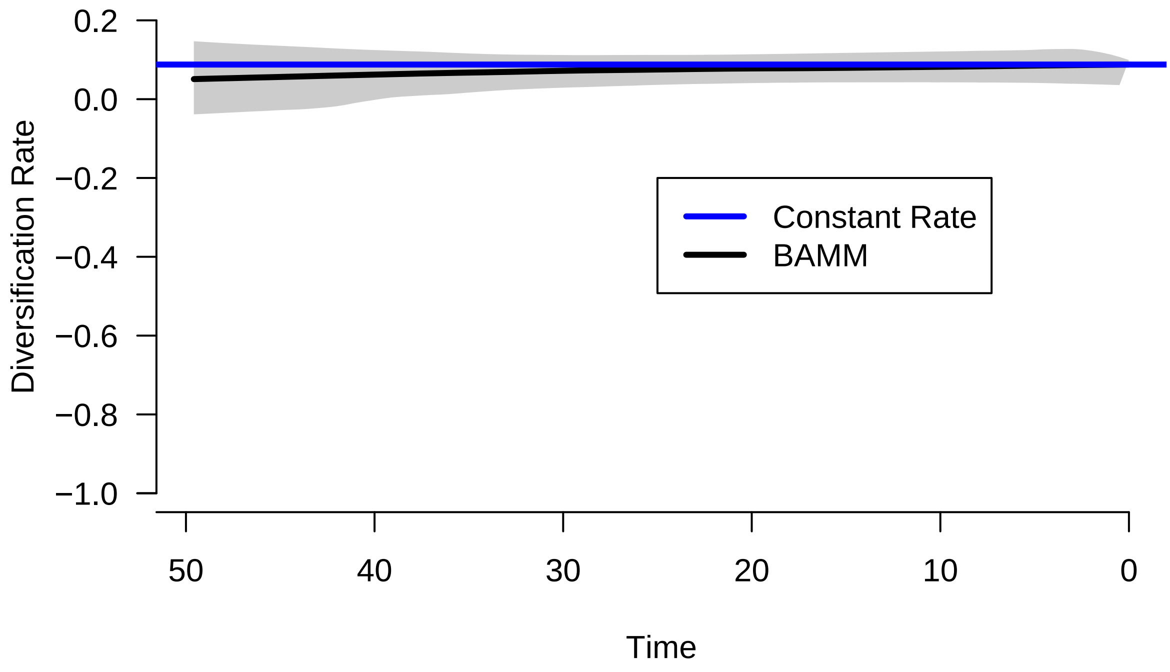

Now we report the diversification rates at the beginning and end of the series.

```
fintab=data.frame(rbind(cbind(head(-rmat$times,1),head(avg,1)),
             cbind(tail(-rmat$times,1),tail(avg,1))))
names(fintab)=c("time", "div.rate")
fintab
```

```
##         time   div.rate
## 1 -49.58148 0.05097435
## 2   0.00000 0.08782602
```

2.4. TESS

The model we run has a strong prior on the magnitude of the single mass extinction. We hypothesize that the mean survival was 5% (95% CI: 0.01-0.10).

```
# Specify the mean and standard deviation of the lognormal
# prior on the speciation rate in real space
speciationPriorMu <- 0.2
#speciationPriorSigma <- 10
speciationPriorSigma <- 0.5
# Specify the mean and standard deviation of the lognormal
# prior on the extinction rate in real space

extinctionPriorMu <- 0.15
extinctionPriorSigma <- 0.5
#We then transform the hyperparameters to reflect the mean and standard of the
```

```r
#log-transformed speciation and extinction rates. These are the mu and sigma parameters
#of the lognormal priors on speciation and extinction rates.
# Transform the priors on the speciation rate into log space.
speciationRatePriorMean <- log((speciationPriorMu^2)/sqrt(speciationPriorSigma^2+
                                                              speciationPriorMu^2))
speciationRatePriorStDev <- sqrt( log(1+speciationPriorSigma^2/(speciationPriorMu^2)))
# Transform the priors on the extinction rate into log space.
extinctionRatePriorMean <- log((extinctionPriorMu^2)/sqrt(extinctionPriorSigma^2+
                                                              extinctionPriorMu^2))
extinctionRatePriorStDev <- sqrt( log(1+extinctionPriorSigma^2/(extinctionPriorMu^2)))

# Finally, we need to specify the prior density on the survival probability of a massextinction
# event. This value reflects the probability that a lineage survives a
# particular mass-extinction event, and therefore must be between 0 (each lineage
# will always go extinct) and 1 (each lineage will always survive). A convenient prior
# density for this parameter is the beta distribution, which has two shape parameters,
# alpha and beta. We use the definition of what constitutes to a mass extinction to inform
# our prior density on this parameter.
# We begin parameterizing this distribution by specifying the expected survival
# probability.
expectedSurvivalProbability <- 0.05

# Using the expected survival probability, we compute the alpha and beta parameters of
# the beta distribution. We set the value of beta to be large, which focuses the prior
# density more tightly around the expected survival probability. Then, we compute
# alpha based on the expected survival probability and the specified beta value.
pMassExtinctionPriorShape2 <- 100
pMassExtinctionPriorShape1 <- - pMassExtinctionPriorShape2 *expectedSurvivalProbability /
  (expectedSurvivalProbability - 1)
# We can inspect this beta distribution to confirm that it accurately reflects our
# prior belief regarding the survival probability
# Plot the density function of our beta distribution.
curve(dbeta(x,shape1=pMassExtinctionPriorShape1,shape2=pMassExtinctionPriorShape2),
      n=1001, xlab='survival probability',ylab='density',las=1)
# Plot the 95% prior interval on the survival probability.
abline(v = qbeta(c(0.025,0.975),shape1=pMassExtinctionPriorShape1,
                 shape2=pMassExtinctionPriorShape2),lty=2)
```

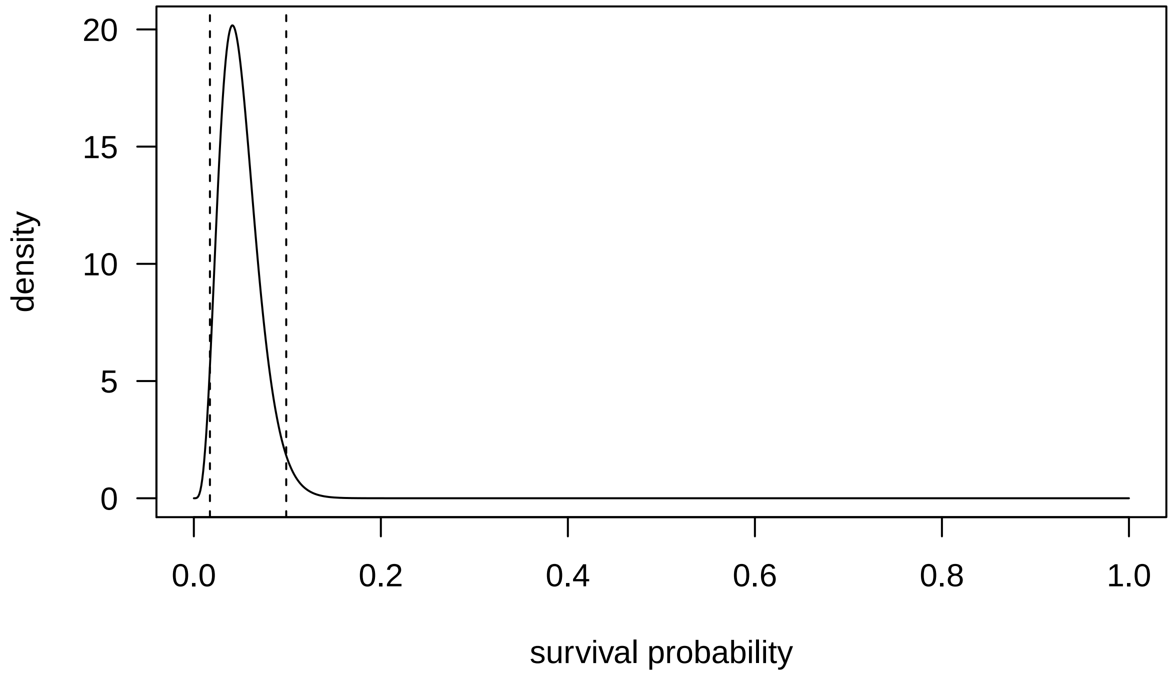


```
# This beta distribution seems to reflect our prior belief that the survival probability
# is expected to be ca. 5%, but can range from about 1% to about 10%.
# Having specified prior distributions for all of the parameters of the CoMET model,
# we can now perform an analysis.

samplingFraction=0.8890909
numExpectedMassExtinctions=1
numExpectedRateChanges=1
set.seed(12345)

#make ultrametric
nnls<-nnls.tree(cophenetic(lemurs),lemurs,rooted=TRUE)
```

```
## [1] "RSS: 4.47340907347883e-08"
```

```
tess.analysis(nnls,
empiricalHyperPriors = FALSE,
initialSpeciationRate = speciationPriorMu,
speciationRatePriorMean = speciationRatePriorMean,
speciationRatePriorStDev = speciationRatePriorStDev,
initialExtinctionRate = extinctionPriorMu,
extinctionRatePriorMean = extinctionRatePriorMean,
extinctionRatePriorStDev = extinctionRatePriorStDev,
samplingProbability = samplingFraction,
numExpectedRateChanges = numExpectedRateChanges,
numExpectedMassExtinctions = numExpectedMassExtinctions,
pMassExtinctionPriorShape1 = pMassExtinctionPriorShape1,
```

```r
pMassExtinctionPriorShape2 = pMassExtinctionPriorShape2,
MAX_ITERATIONS = 10e+06, BURNIN = 1e+05,
dir = "tess_analysis_thinpriors_mu_lambda")
```

```r
tess.analysis(nnls,
empiricalHyperPriors = FALSE,
initialSpeciationRate = speciationPriorMu,
speciationRatePriorMean = speciationRatePriorMean,
speciationRatePriorStDev = speciationRatePriorStDev,
initialExtinctionRate = extinctionPriorMu,
extinctionRatePriorMean = extinctionRatePriorMean,
extinctionRatePriorStDev = extinctionRatePriorStDev,
samplingProbability = samplingFraction,
numExpectedRateChanges = numExpectedRateChanges,
numExpectedMassExtinctions = numExpectedMassExtinctions,
pMassExtinctionPriorShape1 = pMassExtinctionPriorShape1,
pMassExtinctionPriorShape2 = pMassExtinctionPriorShape2,
MAX_ITERATIONS = 10e+06, BURNIN = 1e+05,
dir = "tess_analysis_thinpriors_mu_lambda_run2")
```

```r
tess.analysis(nnls,
empiricalHyperPriors = FALSE,
initialSpeciationRate = speciationPriorMu,
speciationRatePriorMean = speciationRatePriorMean,
speciationRatePriorStDev = speciationRatePriorStDev,
initialExtinctionRate = extinctionPriorMu,
extinctionRatePriorMean = extinctionRatePriorMean,
extinctionRatePriorStDev = extinctionRatePriorStDev,
samplingProbability = samplingFraction,
numExpectedRateChanges = numExpectedRateChanges,
numExpectedMassExtinctions = numExpectedMassExtinctions,
pMassExtinctionPriorShape1 = pMassExtinctionPriorShape1,
pMassExtinctionPriorShape2 = pMassExtinctionPriorShape2,
MAX_ITERATIONS = 10e+06, BURNIN = 1e+05,
dir = "tess_analysis_thinpriors_mu_lambda_run3")
```

```r
tess.analysis(nnls,
empiricalHyperPriors = FALSE,
initialSpeciationRate = speciationPriorMu,
speciationRatePriorMean = speciationRatePriorMean,
speciationRatePriorStDev = speciationRatePriorStDev,
initialExtinctionRate = extinctionPriorMu,
extinctionRatePriorMean = extinctionRatePriorMean,
extinctionRatePriorStDev = extinctionRatePriorStDev,
samplingProbability = samplingFraction,
numExpectedRateChanges = numExpectedRateChanges,
numExpectedMassExtinctions = numExpectedMassExtinctions,
pMassExtinctionPriorShape1 = pMassExtinctionPriorShape1,
pMassExtinctionPriorShape2 = pMassExtinctionPriorShape2,
MAX_ITERATIONS = 10e+06, BURNIN = 1e+05,
dir = "tess_analysis_thinpriors_mu_lambda_run4")
```

First we diagnose the convergence.

```
numExpectedRateChanges=1
numExpectedMassExtinctions=1
output <- tess.process.output("sub3/h1/tess/tess_analysis_thinpriors_mu_lambda",
                              numExpectedRateChanges = numExpectedRateChanges,
                              numExpectedMassExtinctions = numExpectedMassExtinctions,
                              criticalBayesFactors = c(2,6,10))

output2 <- tess.process.output("sub3/h1//tess/tess_analysis_thinpriors_mu_lambda_run2",
                              numExpectedRateChanges = numExpectedRateChanges,
                              numExpectedMassExtinctions = numExpectedMassExtinctions,
                              criticalBayesFactors = c(2,6,10))

output3 <- tess.process.output("sub3/h1//tess/tess_analysis_thinpriors_mu_lambda_run3",
                              numExpectedRateChanges = numExpectedRateChanges,
                              numExpectedMassExtinctions = numExpectedMassExtinctions,
                              criticalBayesFactors = c(2,6,10))

output4 <- tess.process.output("sub3/h1//tess/tess_analysis_thinpriors_mu_lambda_run4",
                              numExpectedRateChanges = numExpectedRateChanges,
                              numExpectedMassExtinctions = numExpectedMassExtinctions,
                              criticalBayesFactors = c(2,6,10))

l=list(output, output2, output3, output4)
tess.plot.multichain.diagnostics(l,parameters="net-diversification rates")
```

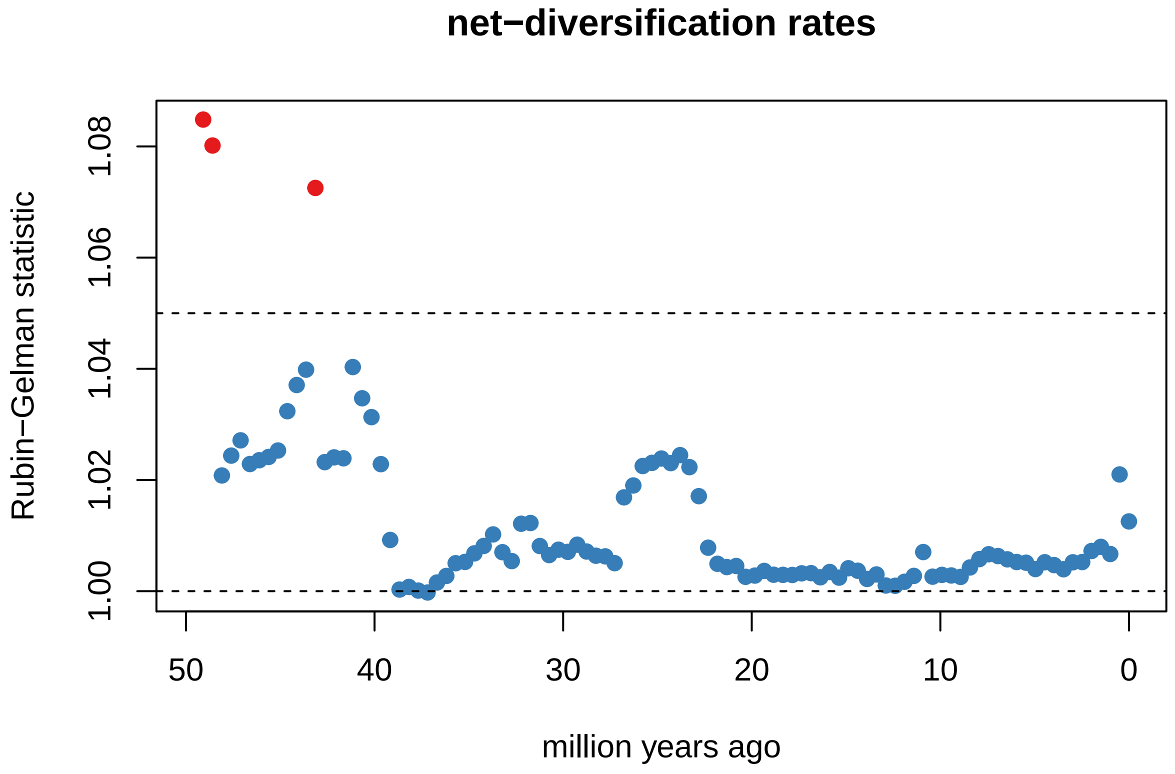

Gelman-Rubin statistics are a measure of within-chain variation to between-chain variation. Borderline

unsatisfactory convergence of Rhat values near 1.05 for pre 40 Ma diversification rates would likely become satisfactory with a few more chains added. This makes sense too, the part of the tree before 40 Ma is poorly parameterized so that is likely where convergence errors would show up. In any case, we decide to use the more lenient threshold of Rhat values of 1.1 to identify converged parameters. Thus, we can look at this plot and say that there is no drastically problematic convergence.

We must select one chain to continute with to use the `tess.plot.output` functions and the next convergence diagnostics. We now look at each chain individually.

```
tess.plot.singlechain.diagnostics(output, parameters = "net-diversification rates")
```

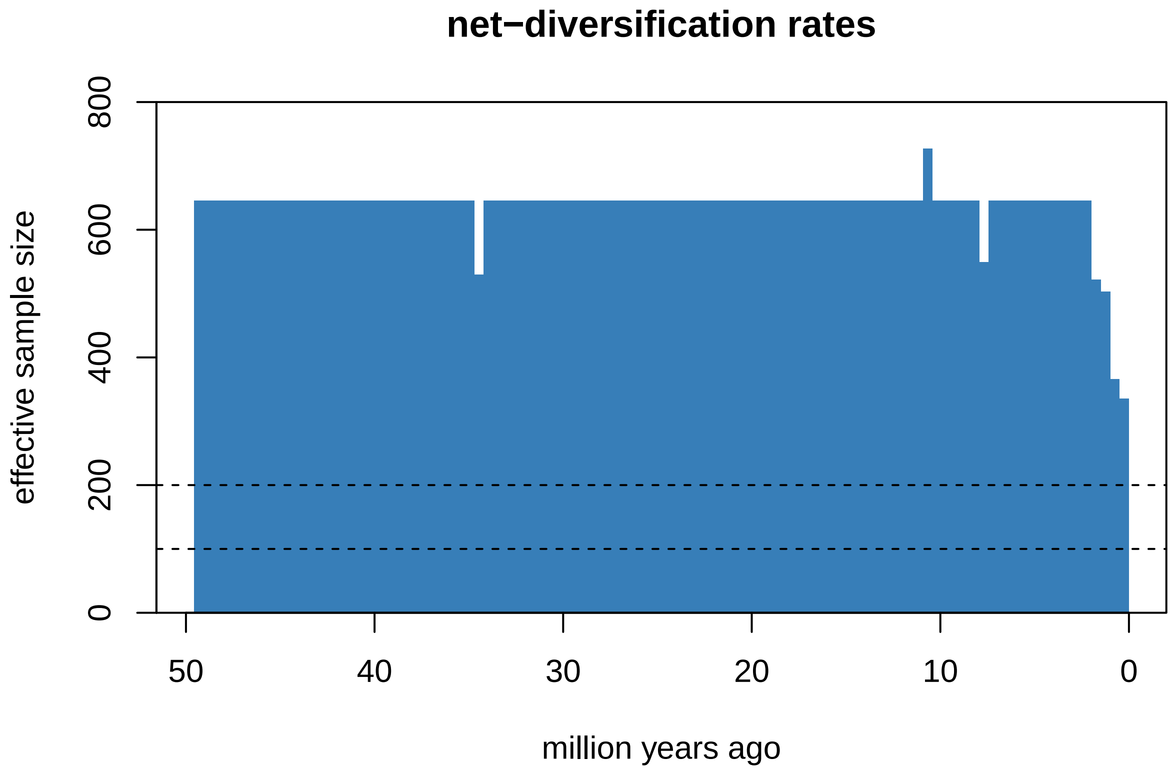

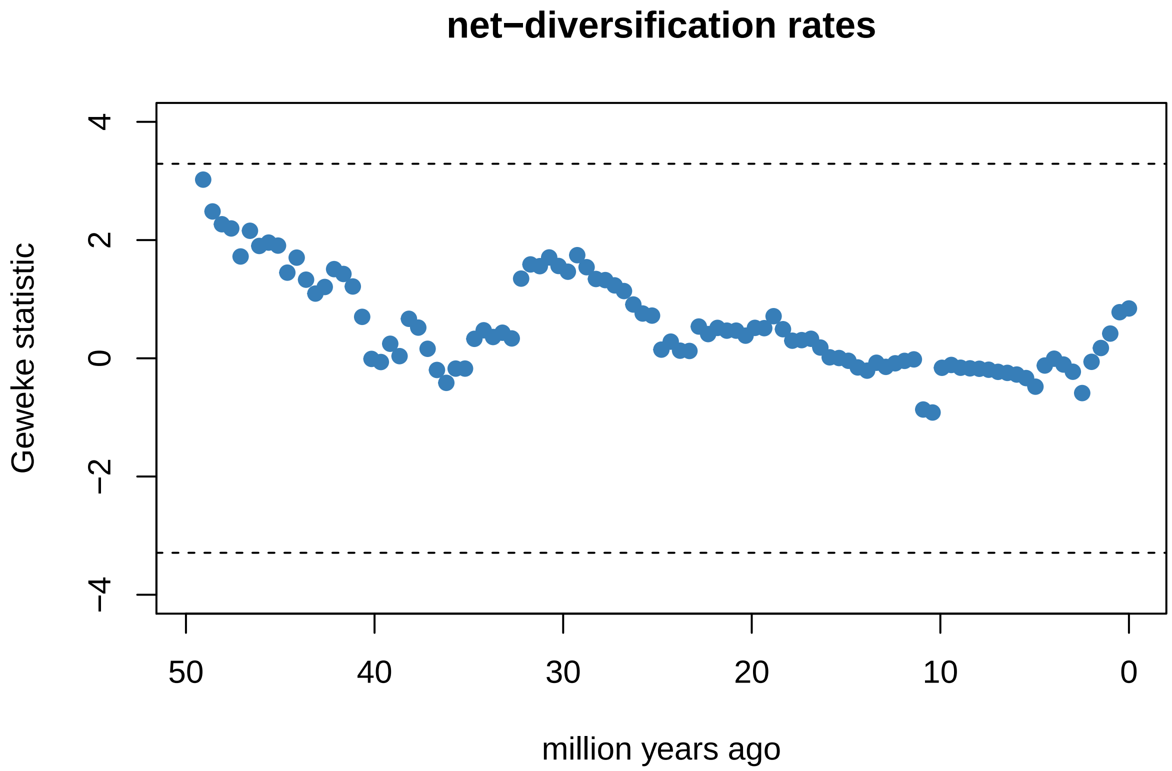


```r
tess.plot.singlechain.diagnostics(output2, parameters = "net-diversification rates")
```

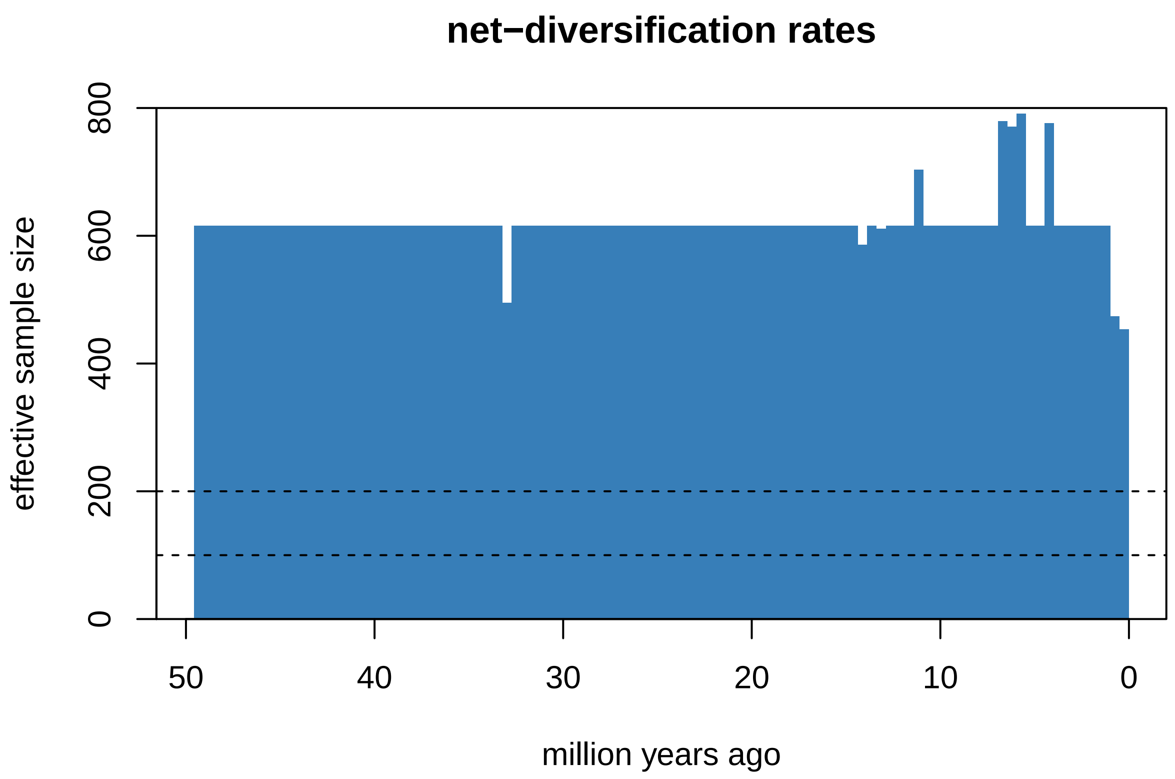
net−diversification rates
effective sample size
800
600
400
200
0
50
40
30
20
10
0
million years ago

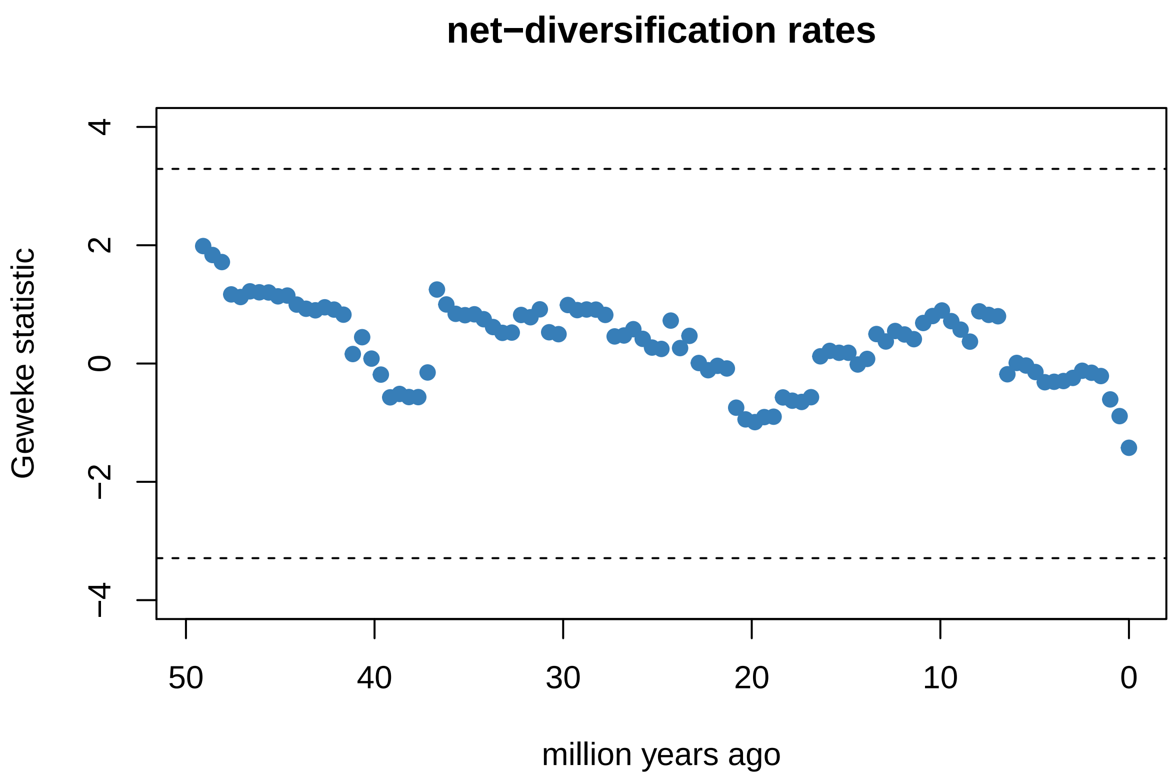


```
tess.plot.singlechain.diagnostics(output3, parameters = "net-diversification rates")
```

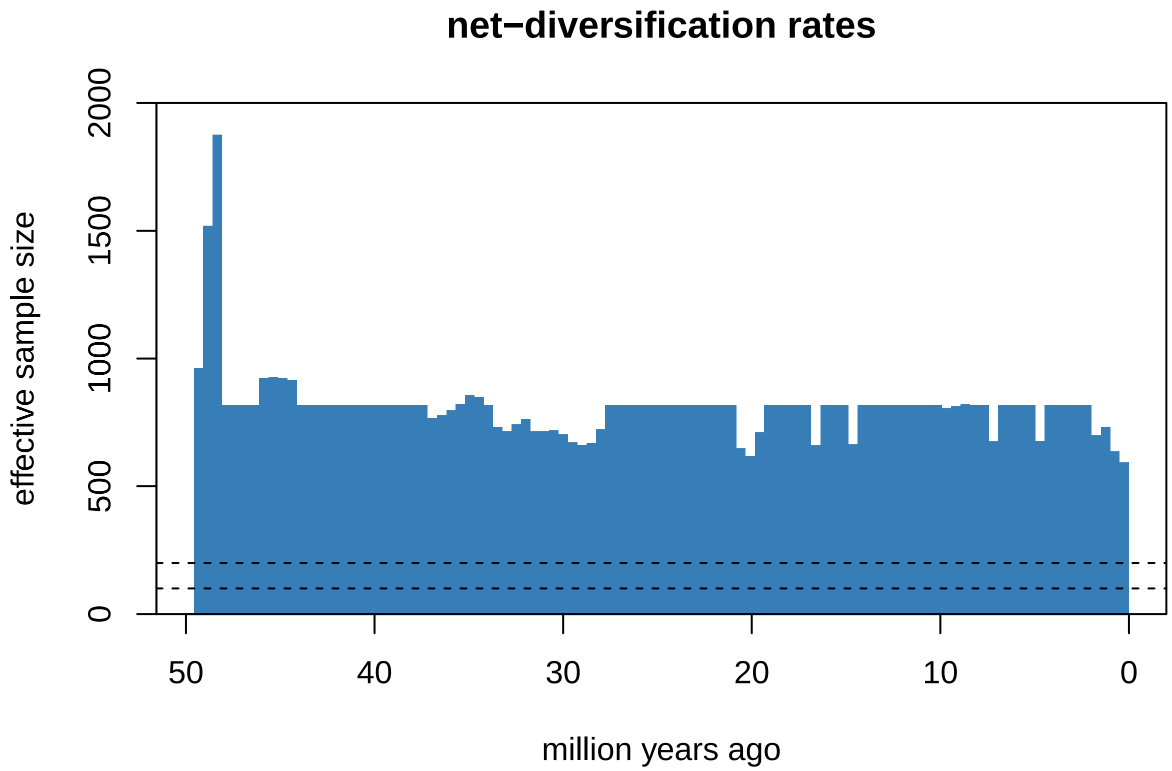
net−diversification rates
effective sample size
0
500
1000
1500
2000
50
40
30
20
10
0
million years ago

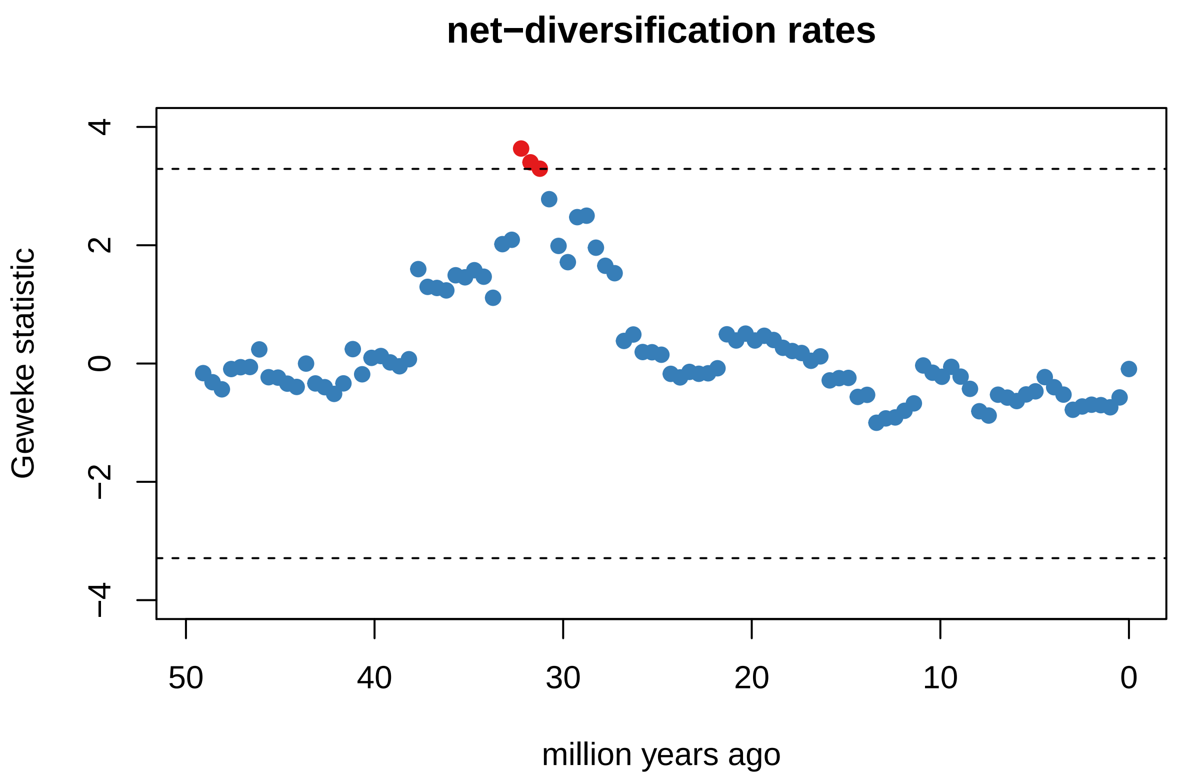


```
tess.plot.singlechain.diagnostics(output4, parameters = "net-diversification rates")
```

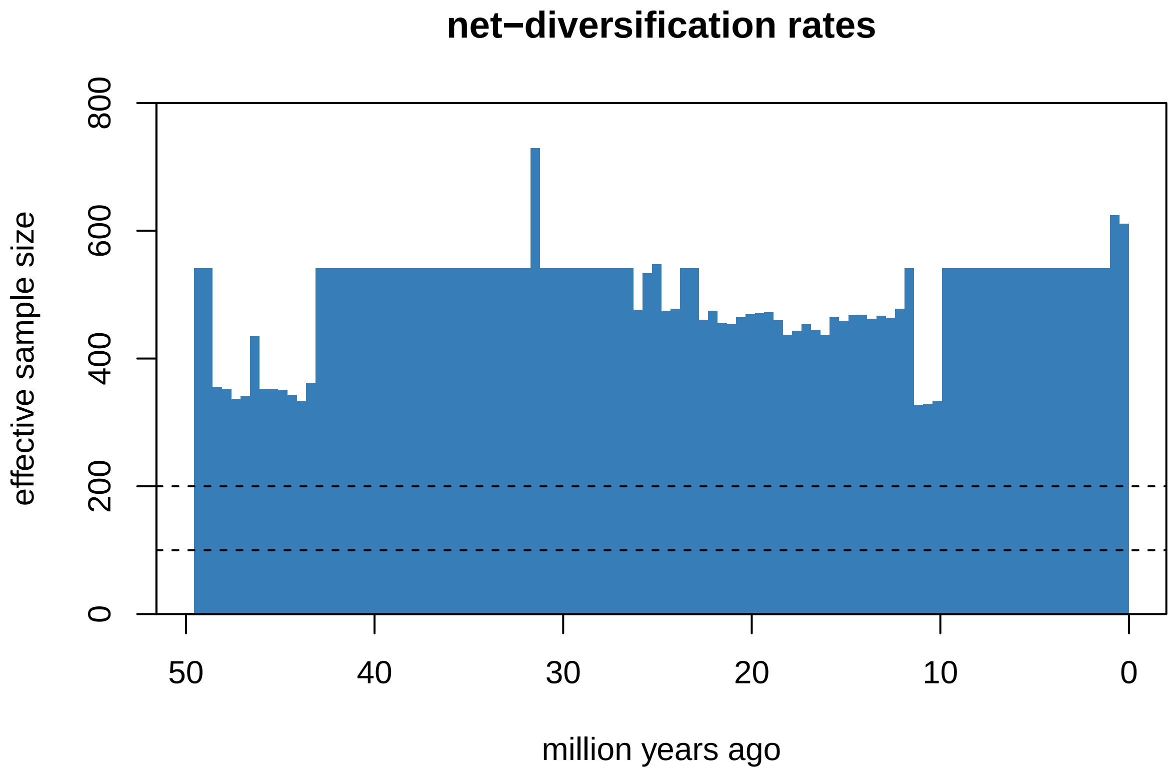
net−diversification rates
effective sample size
0
200
400
600
800
50
40
30
20
10
0
million years ago

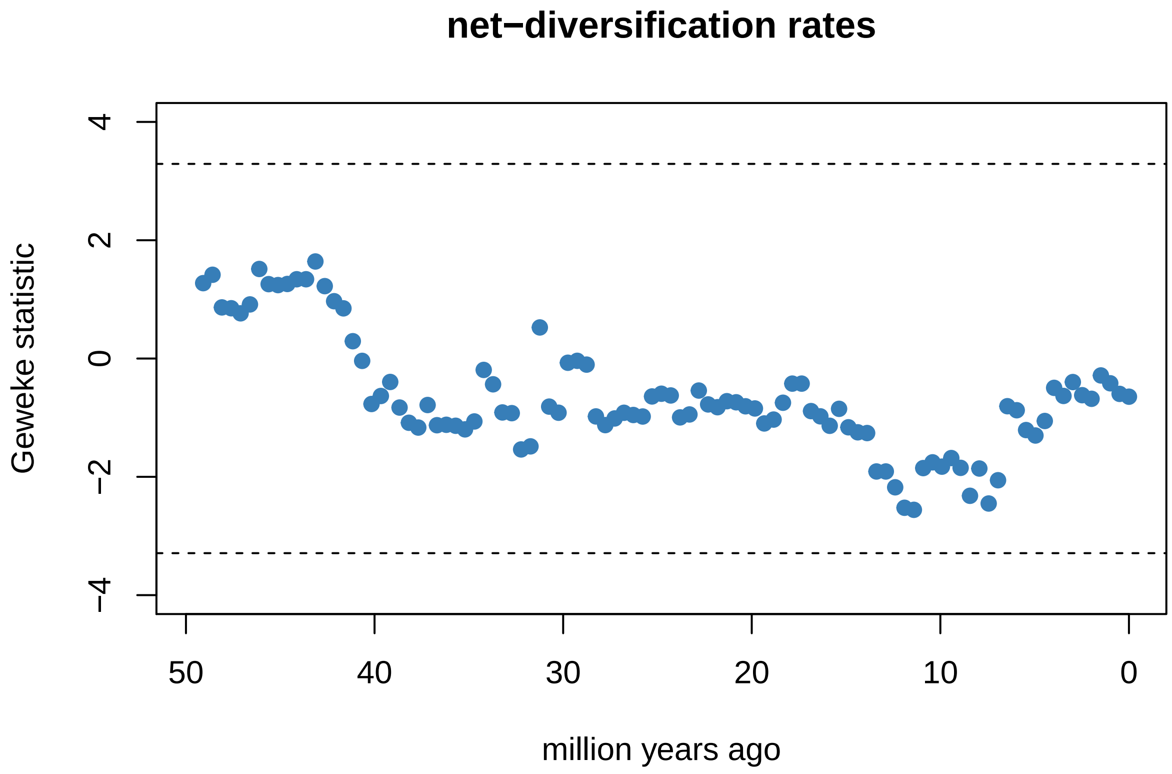

Geweke statistics compare pre burn-in parameter estimates to post-burnin samples with a 2 sample mean test. We thus infer that the burn-in has gone on sufficiently long enough for chains 3 and 4, but not 1 and 2. The effective sample size is sufficiently high for chains 3 and 4 but not 1 and 2.

We now can look if the output from the single chains are different.

```
tess.plot.output(output, "net-diversification rates")
```

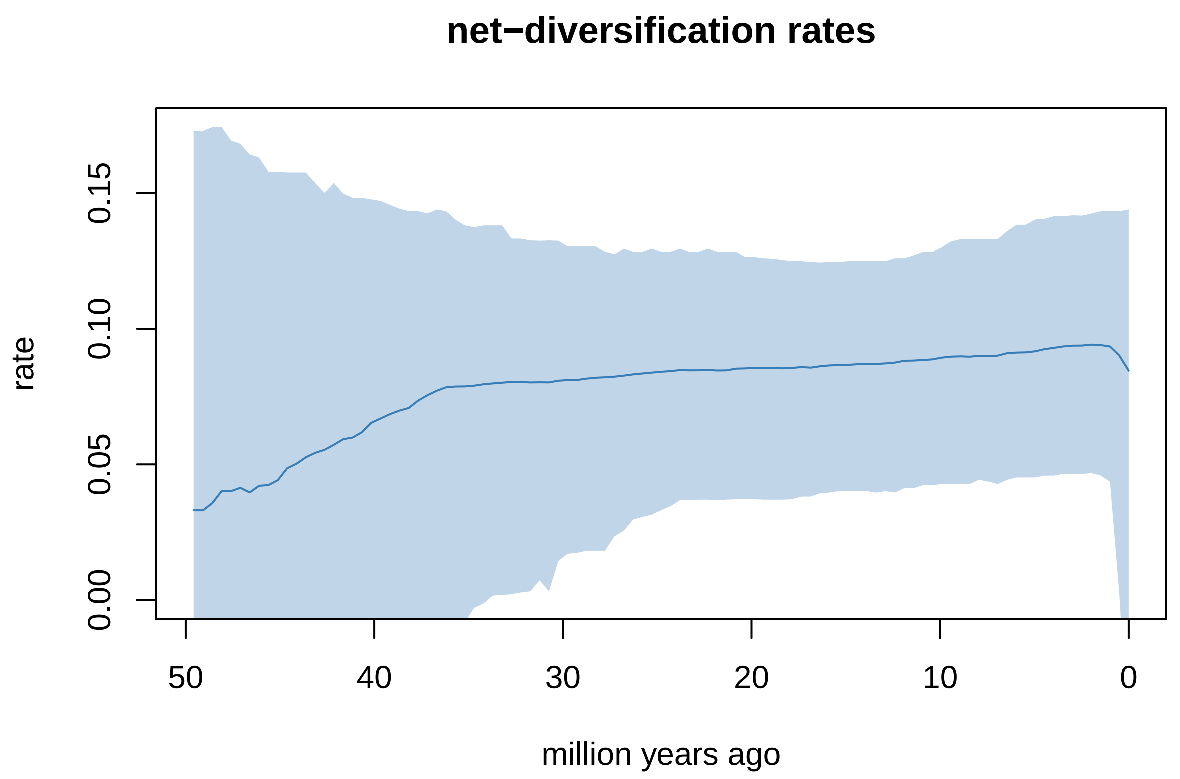


```
tess.plot.output(output2, "net-diversification rates")
```

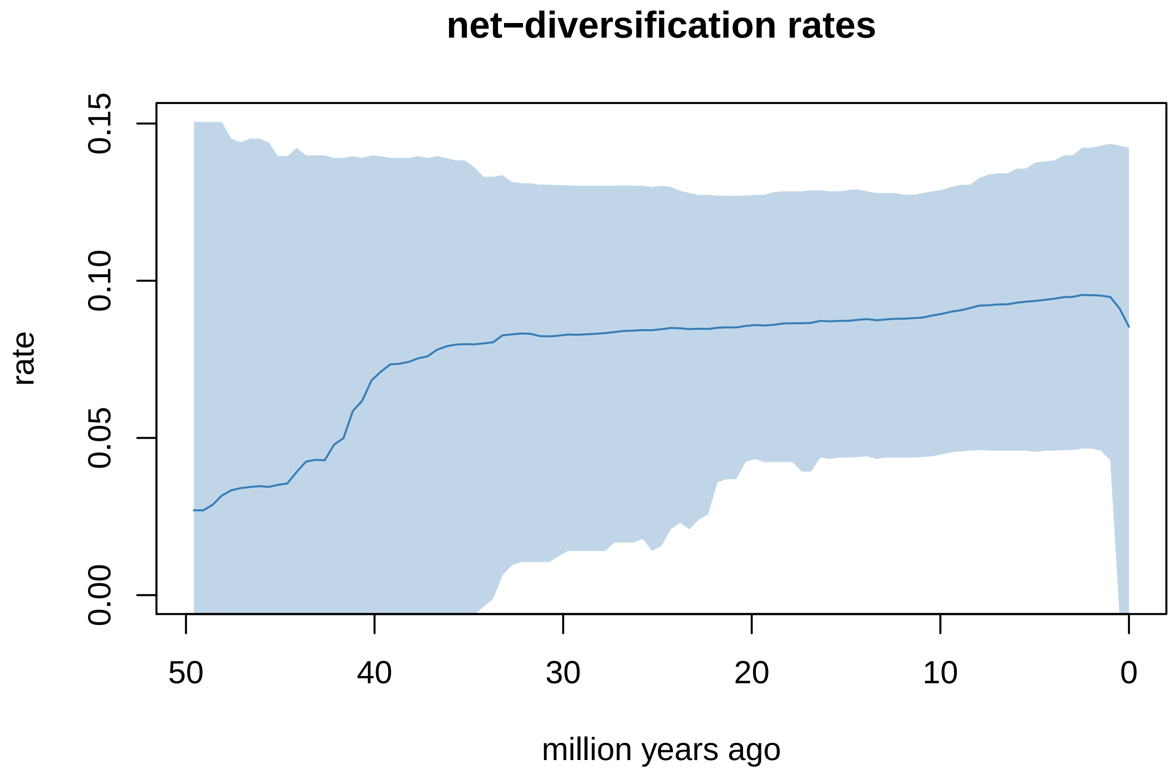


```
tess.plot.output(output3, "net-diversification rates")
```

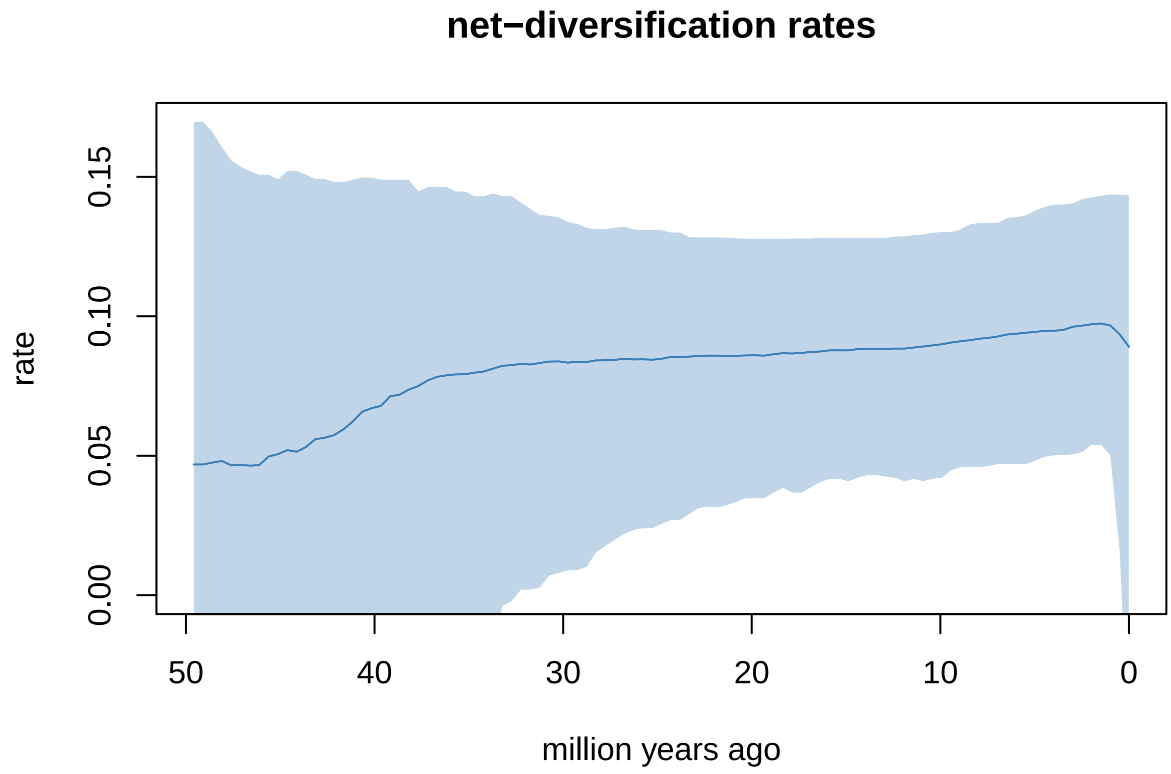


```
tess.plot.output(output4, "net-diversification rates")
```

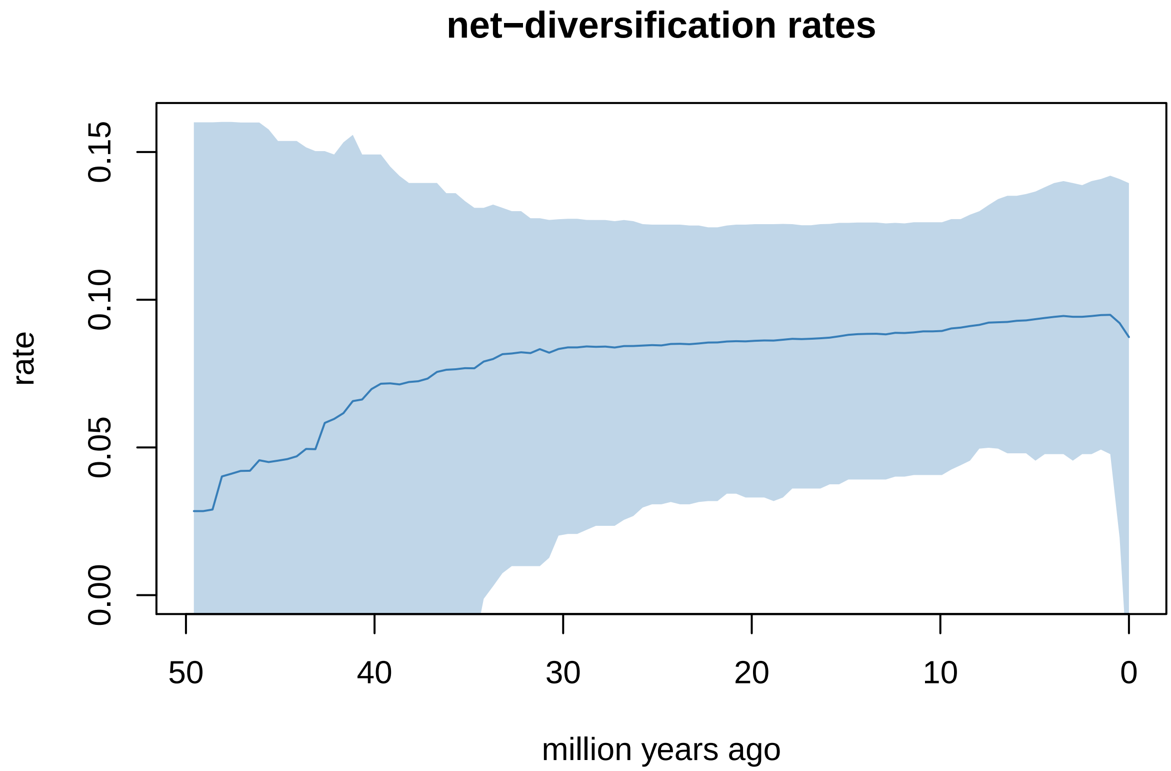

We conclude that the choice of chain does not matter much in the inference. We choose chain 3 to work with, as this was one of the two chains above that showed more satisfactory convergence. Now we can customise the plot from the diversification rate plot from chain 3. And display the point estimates over time.

```
col <- c(`speciation rates` = "#984EA3",
         `speciation shift times` = "#984EA3",
            `speciation Bayes factors` = "#984EA3",
         `extinction rates` = "#E41A1C",
            `extinction shift times` = "#E41A1C",
         `extinction Bayes factors` = "#E41A1C",
            `net-diversification rates` = "#377EB8",
         `relative-extinction rates` = "#FF7F00",
            `mass extinction times` = "#4DAF4A",
         `mass extinction Bayes factors` = "#4DAF4A")
treeAge <- max(branching.times(output3$tree))
numIntervals <- length(output3$intervals) - 1
plotAt <- 0:numIntervals
intervalSize <- treeAge/numIntervals
labels <- pretty(c(0, treeAge))
labelsAt <- numIntervals - (labels/intervalSize)
type="net-diversification rates"
thisOutput <- output3[[type]]
meanThisOutput <- colMeans(thisOutput)
quantilesThisOutput <- apply(thisOutput, 2, quantile, prob = c(0.025, 0.975))
ylim <- c(-0.8, max(quantilesThisOutput))

plot.new()
```

```r
plot.window(xlim = range((plotAt-100)/2), ylim = ylim)
lines(x = (plotAt-100)/2, y = c(meanThisOutput[1], meanThisOutput),
      col = col[type],  lwd=3)
polygon(x = c(c(0:ncol(quantilesThisOutput)-100)/2,
              c(c(ncol(quantilesThisOutput):0)-100)/2),
  y = c(c(quantilesThisOutput[1, 1], quantilesThisOutput[1, ]),
        rev(c(quantilesThisOutput[2, 1], quantilesThisOutput[2,]))),
  border = NA, col = paste(col[type], col.alpha=50,
  sep = ""))
axis(at = c(round(1.2 * 50), axTicks(1)), side = 1)
axis(at = round(c(-1, axTicks(2)),2), las = 1, side = 2)
mtext(side = 1, text = "Time",line = 3)
mtext(side = 2, text = "Diversification Rate", line = 3)
```

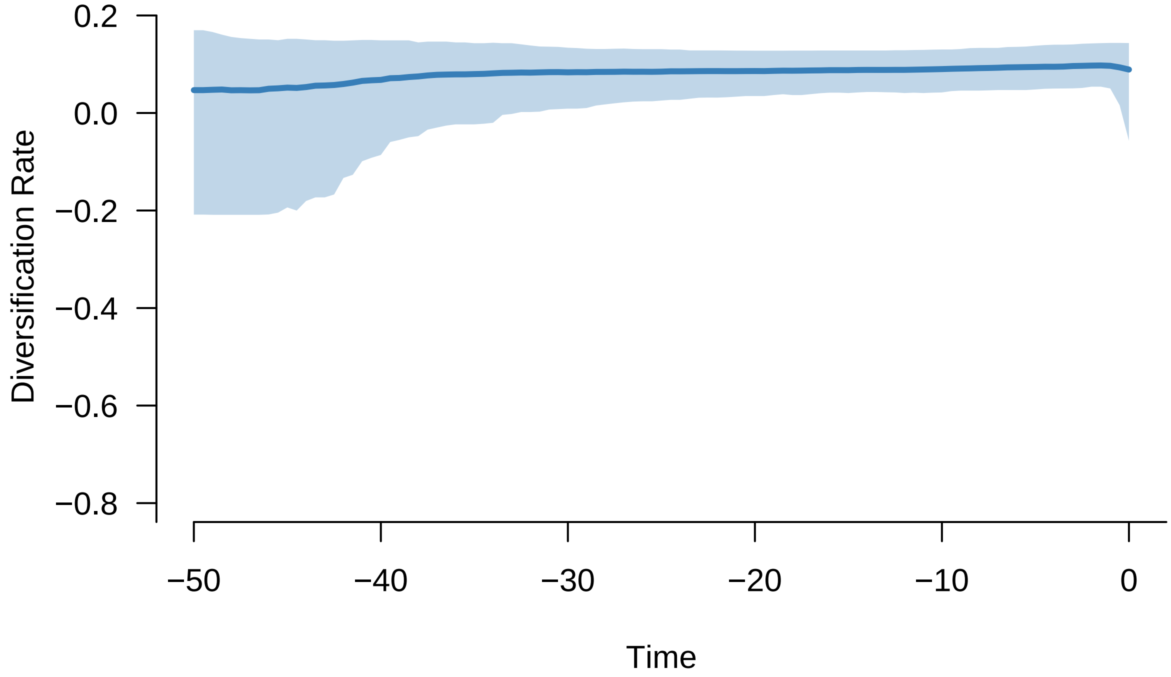


```r
cbind((plotAt-100)/2,
      c(meanThisOutput[1], meanThisOutput))
```

```
##         [,1]       [,2]
##   [1,] -50.0 0.04686094
##   [2,] -49.5 0.04686094
##   [3,] -49.0 0.04756339
##   [4,] -48.5 0.04810721
##   [5,] -48.0 0.04655589
##   [6,] -47.5 0.04674832
##   [7,] -47.0 0.04642556
##   [8,] -46.5 0.04667030
```

```
##   [9,] -46.0 0.04970537
##  [10,] -45.5 0.05056379
##  [11,] -45.0 0.05199394
##  [12,] -44.5 0.05142636
##  [13,] -44.0 0.05314819
##  [14,] -43.5 0.05593315
##  [15,] -43.0 0.05644983
##  [16,] -42.5 0.05737634
##  [17,] -42.0 0.05946320
##  [18,] -41.5 0.06225166
##  [19,] -41.0 0.06577075
##  [20,] -40.5 0.06703555
##  [21,] -40.0 0.06787426
##  [22,] -39.5 0.07131163
##  [23,] -39.0 0.07187869
##  [24,] -38.5 0.07373642
##  [25,] -38.0 0.07498541
##  [26,] -37.5 0.07697770
##  [27,] -37.0 0.07828507
##  [28,] -36.5 0.07881887
##  [29,] -36.0 0.07916611
##  [30,] -35.5 0.07923202
##  [31,] -35.0 0.07977727
##  [32,] -34.5 0.08019878
##  [33,] -34.0 0.08124710
##  [34,] -33.5 0.08225605
##  [35,] -33.0 0.08250303
##  [36,] -32.5 0.08293365
##  [37,] -32.0 0.08272660
##  [38,] -31.5 0.08325529
##  [39,] -31.0 0.08376910
##  [40,] -30.5 0.08384482
##  [41,] -30.0 0.08338453
##  [42,] -29.5 0.08370451
##  [43,] -29.0 0.08360811
##  [44,] -28.5 0.08418738
##  [45,] -28.0 0.08424737
##  [46,] -27.5 0.08437265
##  [47,] -27.0 0.08478021
##  [48,] -26.5 0.08452811
##  [49,] -26.0 0.08459902
##  [50,] -25.5 0.08442482
##  [51,] -25.0 0.08473697
##  [52,] -24.5 0.08546907
##  [53,] -24.0 0.08543296
##  [54,] -23.5 0.08555094
##  [55,] -23.0 0.08580044
##  [56,] -22.5 0.08590255
##  [57,] -22.0 0.08588025
##  [58,] -21.5 0.08581550
##  [59,] -21.0 0.08581116
##  [60,] -20.5 0.08597869
##  [61,] -20.0 0.08603742
##  [62,] -19.5 0.08589167
```

```
##  [63,] -19.0 0.08641133
##  [64,] -18.5 0.08678117
##  [65,] -18.0 0.08667482
##  [66,] -17.5 0.08688781
##  [67,] -17.0 0.08721156
##  [68,] -16.5 0.08737078
##  [69,] -16.0 0.08779793
##  [70,] -15.5 0.08780244
##  [71,] -15.0 0.08779043
##  [72,] -14.5 0.08825699
##  [73,] -14.0 0.08835326
##  [74,] -13.5 0.08833855
##  [75,] -13.0 0.08829144
##  [76,] -12.5 0.08841894
##  [77,] -12.0 0.08841481
##  [78,] -11.5 0.08880892
##  [79,] -11.0 0.08917028
##  [80,] -10.5 0.08957584
##  [81,] -10.0 0.08999759
##  [82,]  -9.5 0.09058955
##  [83,]  -9.0 0.09105700
##  [84,]  -8.5 0.09144372
##  [85,]  -8.0 0.09195485
##  [86,]  -7.5 0.09228081
##  [87,]  -7.0 0.09279233
##  [88,]  -6.5 0.09348624
##  [89,]  -6.0 0.09376662
##  [90,]  -5.5 0.09411765
##  [91,]  -5.0 0.09440651
##  [92,]  -4.5 0.09484499
##  [93,]  -4.0 0.09478871
##  [94,]  -3.5 0.09512440
##  [95,]  -3.0 0.09626950
##  [96,]  -2.5 0.09666277
##  [97,]  -2.0 0.09715108
##  [98,]  -1.5 0.09742904
##  [99,]  -1.0 0.09671640
## [100,]  -0.5 0.09356837
## [101,]   0.0 0.08907954
```

Sensitivity analysis showing how the diversification rates compare

Weaker Prior 1

We first put a weaker prior on the magnitude of extinction. In this exercise we hypothesize that the mean survival was 25% (95% CI: 0.07-0.50).

```
speciationPriorMu <- 0.2
speciationPriorSigma <- 0.5
extinctionPriorMu <- 0.15
extinctionPriorSigma <- 0.5
speciationRatePriorMean <- log((speciationPriorMu^2)/sqrt(speciationPriorSigma^2+
                                                           speciationPriorMu^2))
speciationRatePriorStDev <- sqrt( log(1+speciationPriorSigma^2/(speciationPriorMu^2)))
```

```
extinctionRatePriorMean <- log((extinctionPriorMu^2)/sqrt(extinctionPriorSigma^2+
                                                         extinctionPriorMu^2))
extinctionRatePriorStDev <- sqrt( log(1+extinctionPriorSigma^2/(extinctionPriorMu^2)))

expectedSurvivalProbability <- 0.25
pMassExtinctionPriorShape2 <- 10
pMassExtinctionPriorShape1 <- - pMassExtinctionPriorShape2 *expectedSurvivalProbability /
  (expectedSurvivalProbability - 1)
curve(dbeta(x,shape1=pMassExtinctionPriorShape1,shape2=pMassExtinctionPriorShape2),
      n=1001, xlab='survival probability',ylab='density',las=1)
abline(v = qbeta(c(0.025,0.975),shape1=pMassExtinctionPriorShape1,
                 shape2=pMassExtinctionPriorShape2),lty=2)
```

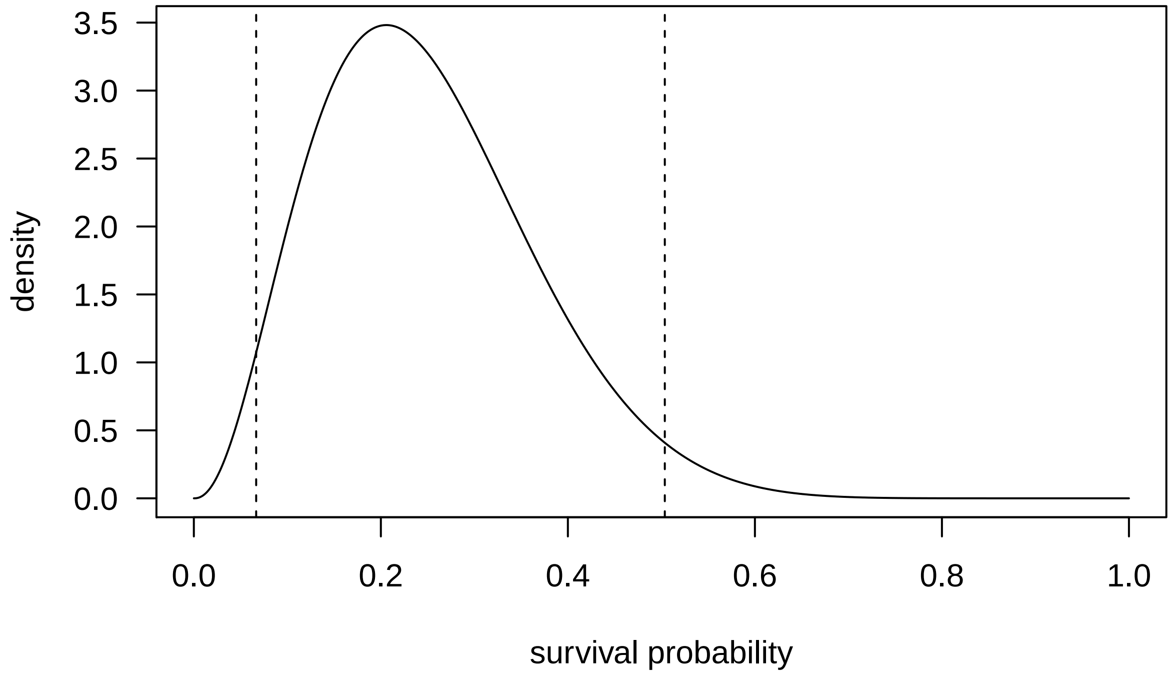


```
samplingFraction=0.8890909
numExpectedMassExtinctions=1
numExpectedRateChanges=1
set.seed(12345)
nnls<-nnls.tree(cophenetic(lemurs),lemurs,rooted=TRUE)
```

```
## [1] "RSS: 4.47340907347883e-08"
```

Now we run the model.

```
tess.analysis(nnls,
empiricalHyperPriors = FALSE,
initialSpeciationRate = speciationPriorMu,
speciationRatePriorMean = speciationRatePriorMean,
```

```
speciationRatePriorStDev = speciationRatePriorStDev,
initialExtinctionRate = extinctionPriorMu,
extinctionRatePriorMean = extinctionRatePriorMean,
extinctionRatePriorStDev = extinctionRatePriorStDev,
samplingProbability = samplingFraction,
numExpectedRateChanges = numExpectedRateChanges,
numExpectedMassExtinctions = numExpectedMassExtinctions,
pMassExtinctionPriorShape1 = pMassExtinctionPriorShape1,
pMassExtinctionPriorShape2 = pMassExtinctionPriorShape2,
MAX_ITERATIONS = 10e+06,
BURNIN = 2e+06,
dir = "tess_analysis_thinpriors_mu_lambda_weakextinction1")
```

```
output <- tess.process.output("sub3/h1/tess/tess_analysis_thinpriors_mu_lambda_weakextinction1",
numExpectedRateChanges = numExpectedRateChanges,
numExpectedMassExtinctions = numExpectedMassExtinctions)
tess.plot.singlechain.diagnostics(output,parameters = c("net-diversification rates"),las=2)
```

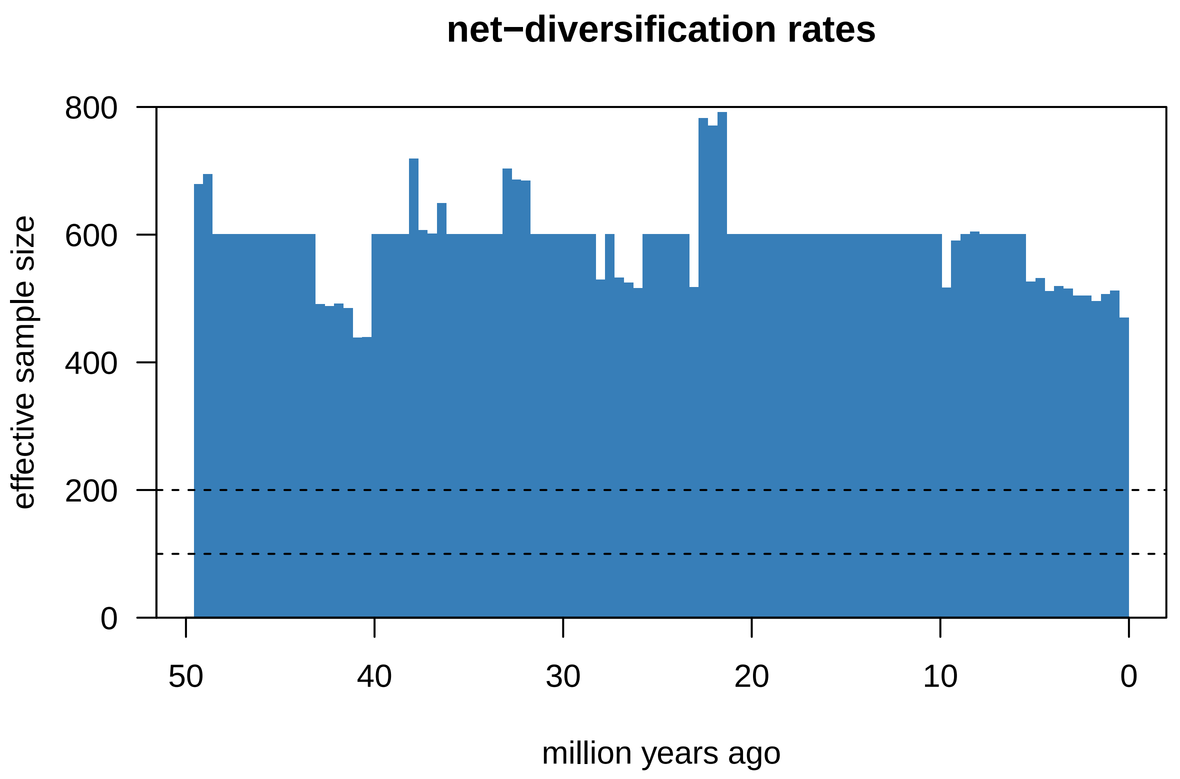

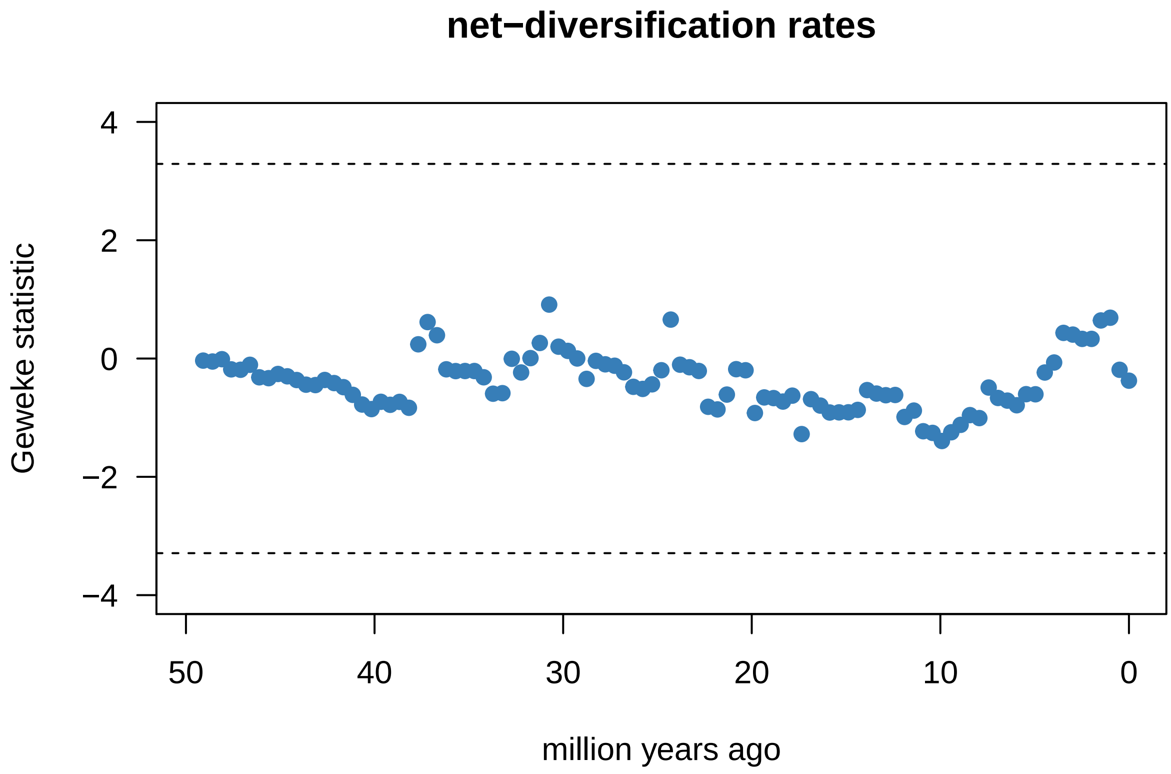


```
tess.plot.output(output,fig.types =  c("net-diversification rates"),las=2)
```

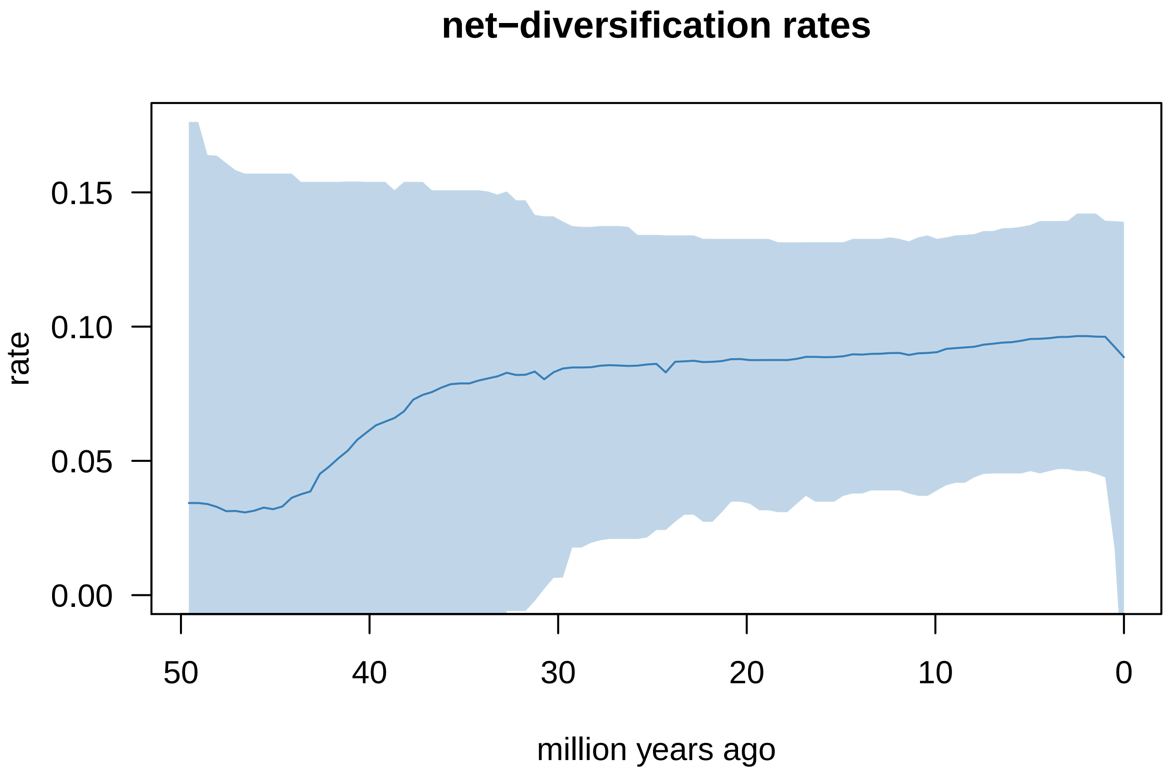

This looks similar to what the analysis with the strong prior indicates.

Weak Prior 2

Now we hypothesize that the magnitude of survival was 0.75 (95% CI: 0.48-0.94).

```
speciationPriorMu <- 0.2
speciationPriorSigma <- 0.5
extinctionPriorMu <- 0.15
extinctionPriorSigma <- 0.5
speciationRatePriorMean <- log((speciationPriorMu^2)/sqrt(speciationPriorSigma^2+
                                                            speciationPriorMu^2))
speciationRatePriorStDev <- sqrt( log(1+speciationPriorSigma^2/(speciationPriorMu^2)))
extinctionRatePriorMean <- log((extinctionPriorMu^2)/sqrt(extinctionPriorSigma^2+
                                                            extinctionPriorMu^2))
extinctionRatePriorStDev <- sqrt( log(1+extinctionPriorSigma^2/(extinctionPriorMu^2)))
expectedSurvivalProbability <- 0.75
pMassExtinctionPriorShape2 <- 3
pMassExtinctionPriorShape1 <- - pMassExtinctionPriorShape2 *
  expectedSurvivalProbability /(expectedSurvivalProbability - 1)
curve(dbeta(x,shape1=pMassExtinctionPriorShape1,shape2=pMassExtinctionPriorShape2),
      n=1001, xlab='survival probability',ylab='density',las=1)
abline(v = qbeta(c(0.025,0.975),shape1=pMassExtinctionPriorShape1,
                 shape2=pMassExtinctionPriorShape2),lty=2)
```

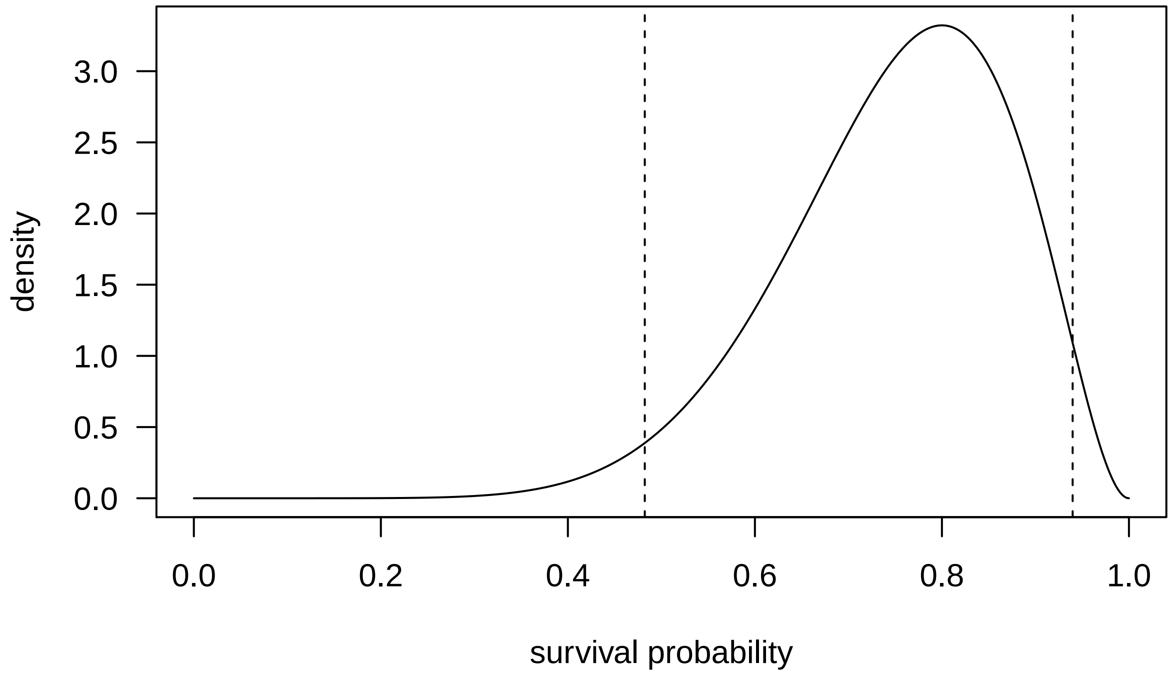


```r
samplingFraction=0.8890909
numExpectedMassExtinctions=1
numExpectedRateChanges=1
set.seed(12345)
nnls<-nnls.tree(cophenetic(lemurs),lemurs,rooted=TRUE)
```

```
## [1] "RSS: 4.47340907347883e-08"
```

Now we run the analysis.

```r
tess.analysis(nnls,
              empiricalHyperPriors = FALSE,
              initialSpeciationRate = speciationPriorMu,
              speciationRatePriorMean = speciationRatePriorMean,
              speciationRatePriorStDev = speciationRatePriorStDev,
              initialExtinctionRate = extinctionPriorMu,
              extinctionRatePriorMean = extinctionRatePriorMean,
              extinctionRatePriorStDev = extinctionRatePriorStDev,
              samplingProbability = samplingFraction,
              numExpectedRateChanges = numExpectedRateChanges,
              numExpectedMassExtinctions = numExpectedMassExtinctions,
              pMassExtinctionPriorShape1 = pMassExtinctionPriorShape1,
              pMassExtinctionPriorShape2 = pMassExtinctionPriorShape2,
              MAX_ITERATIONS = 10e+06, BURNIN = 2e+06,
              dir = "tess_analysis_thinpriors_mu_lambda_weakprior2")
```

```r
output <- tess.process.output("sub3/h1/tess/tess_analysis_thinpriors_mu_lambda_weakprior2",
numExpectedRateChanges = numExpectedRateChanges,
```

```
numExpectedMassExtinctions = numExpectedMassExtinctions)
tess.plot.singlechain.diagnostics(output,parameters = c("net-diversification rates"),las=2)
```

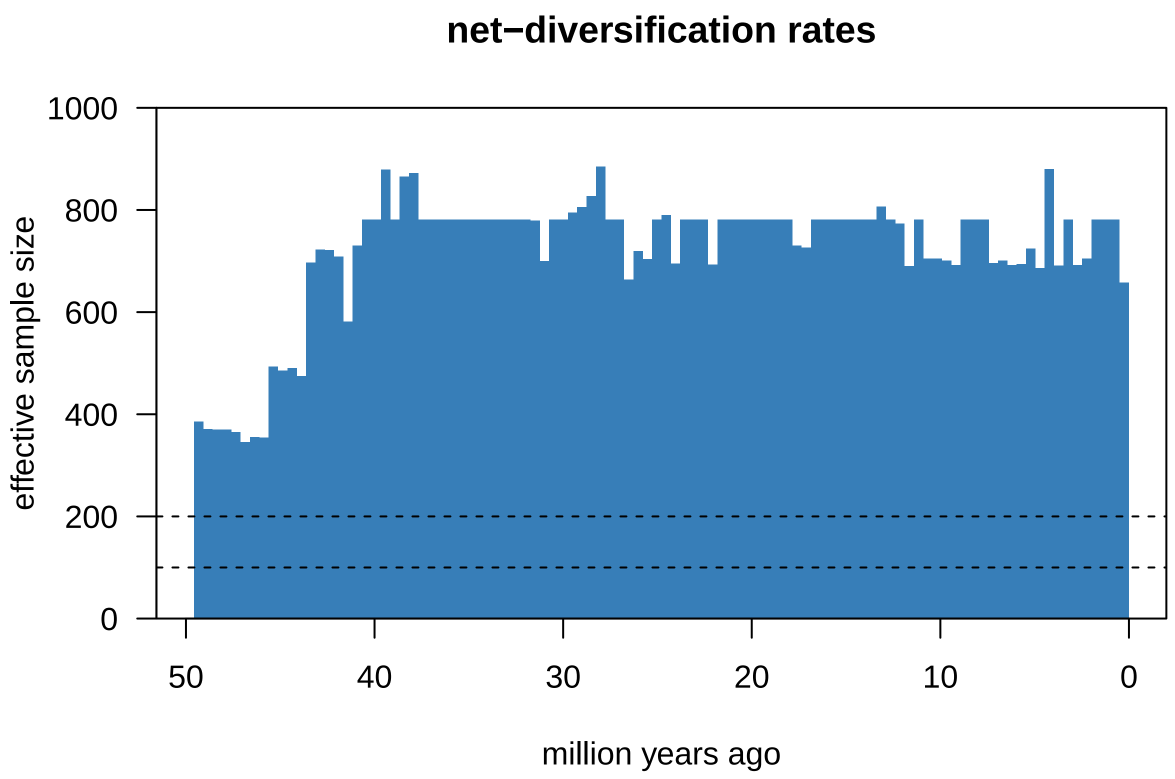

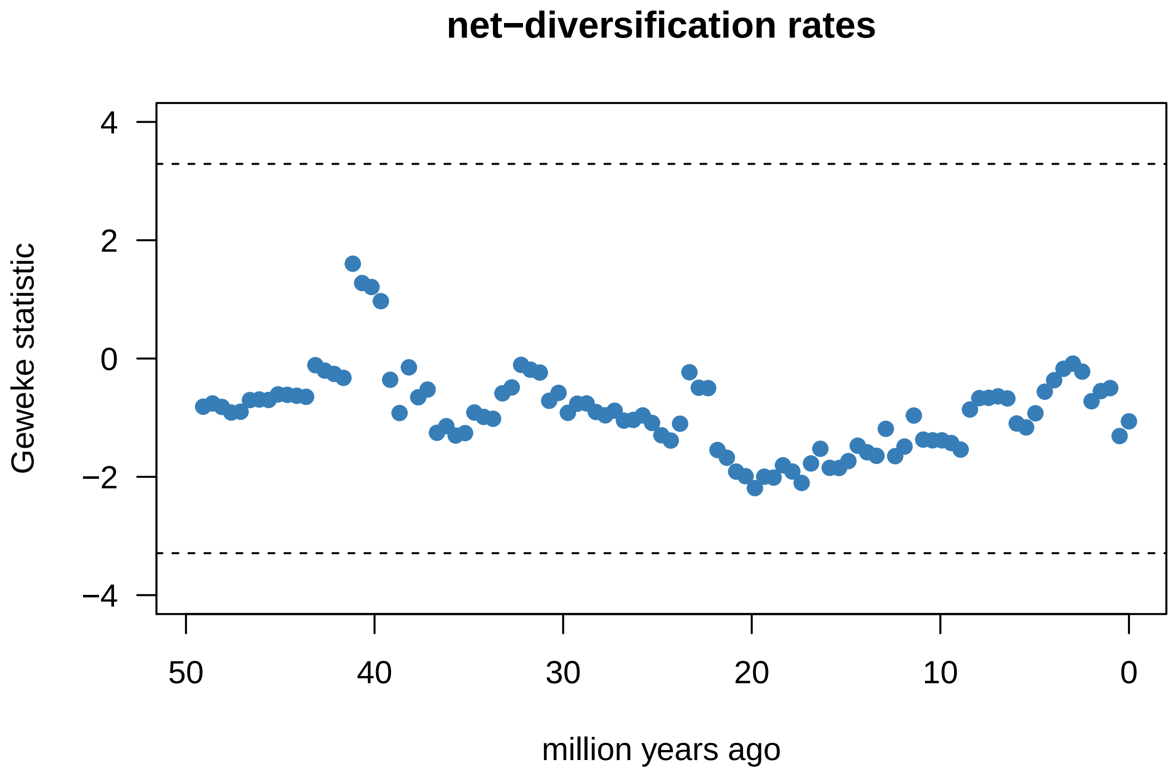


```
tess.plot.output(output,fig.types = c("net-diversification rates"),las=2)
```

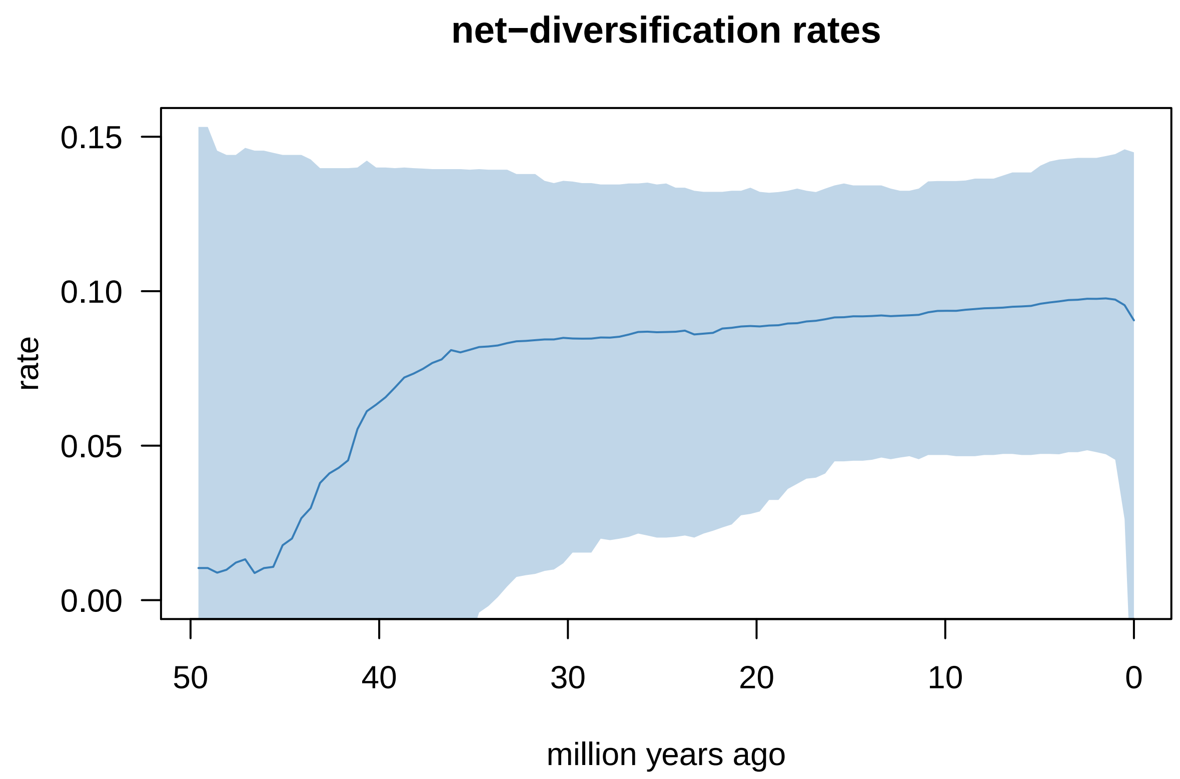

This is qualitatively similar.

2.5. Plot All Models of Diversification Rate Together

```
lthick=3
plot.new()
##
# BAMM
##
                xMin <- -maxTime
                xMax <- 0
                yMin <- -0.8
                yMax <- 0.2
            plot.window(xlim = c(xMin, xMax), ylim = c(yMin,
                yMax))
            xticks=yticks=NULL
               axis(at = c(round(1.2 * xMin), axTicks(1)), cex.axis = 1,
                  side = 1)
                axis(at = round(c(-1, axTicks(2)),2), cex.axis = 1,
                  las = 1, side = 2)
                 axis.labels=TRUE
                xline = 3
                yline = 3
            if (axis.labels) {
                mtext(side = 1, text = "Time",
```

```
                  line = xline)
                mtext(side = 2, text = "Diversification Rate", line = yline)
            }
                axis.labels=FALSE
                 intervalCol='gray80'
                opacity=1
        if (!is.null(intervals)) {
            for (i in 1:length(poly)) {
                polygon(x = -poly[[i]][, 1], y = poly[[i]][, 2],
                  col = transparentColor(intervalCol, opacity),
                  border = NA)
            }
        }
        lines(x = -rmat$time, y = avg, col = "black", lwd=lthick)
        abline(h=0, col="black", lty=2)
##
# RPANDA
##
lines(-t, r(results$lemurs.res$bcstdcst, t), col="blue", lwd=lthick)
lines(-t, r(results$lemurs.res$bcstdvar, t),  col="red", lwd=lthick)

##
# Treepar
##
lines(diversification~c(time_mya), data=treepar.df, col="purple", lwd=lthick)

####
# TESS
###
lines(x = (plotAt-100)/2, y = c(meanThisOutput[1], meanThisOutput),
      col = col[type],  lwd=3)
polygon(x = c(c(0:ncol(quantilesThisOutput)-100)/2,
              c(c(ncol(quantilesThisOutput):0)-100)/2),
  y = c(c(quantilesThisOutput[1, 1], quantilesThisOutput[1, ]),
        rev(c(quantilesThisOutput[2, 1], quantilesThisOutput[2,]))),
  border = NA, col = paste(col[type], col.alpha=50,
  sep = ""))

###
# Legend and indicator of 34 Ma
###
legend(-25,-0.025, c("BAMM","RPANDA: ScstEcst","RPANDA: ScstEvar","TreePar", "TESS"),
       lty=c(1,1,1,1,1),lwd=c(3,3,3,3,3),
       col=c("black","blue","red","purple",col[type]))
abline(h = 0.2, v = -34, col = "lightgray", lty = 3)
```

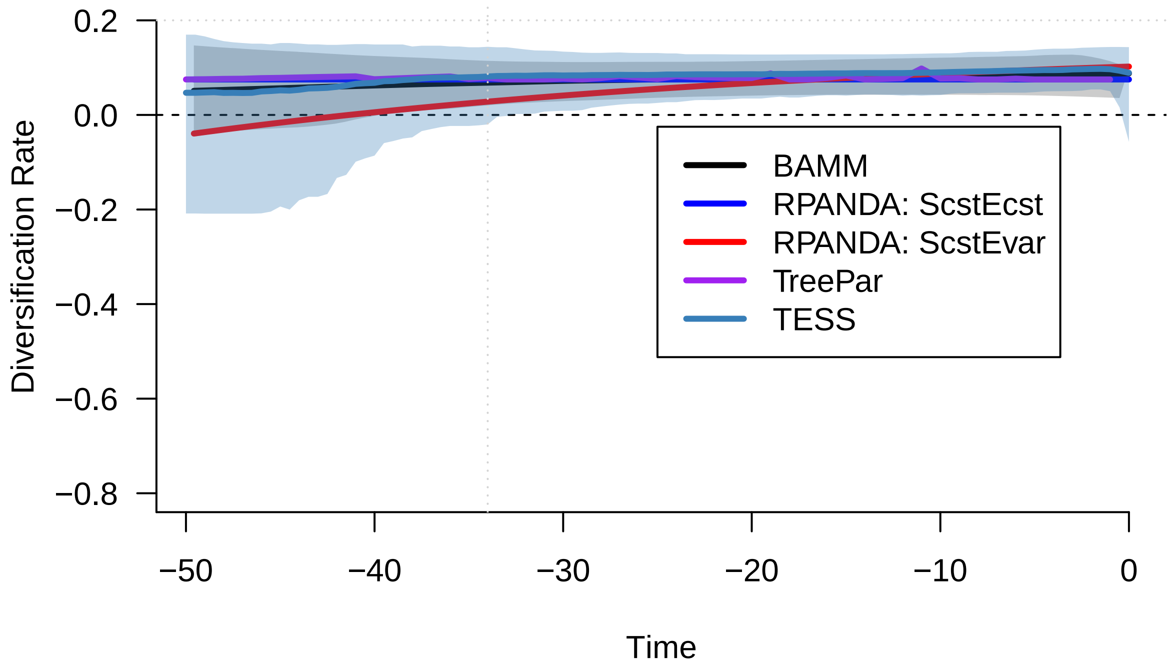

3. Test for Mass Extinction

3.1. RPANDA

With RPANDA, we construct a series of candidate "environmental variables" that we consider as predictors of extinction rate. Each variable is a flat line with a strong pulse at a different year. The variable is constructed by the probability distribution function (PDF) of a Normal distribution with variance equal to 0.25 and mean equal to the year in question. For example, the variable that corresponds to a pulsed extinction at 49 Ma is the PDF of a Normal distribution with mean 49 and variance 0.25 over the domain 0 to 50, and the variable that corresponds to a pulsed extinction at 48 Ma is the PDF of a Normal distribution with mean 48 and variance 0.25 over the domain 0 to 50 and so forth. We also construct a null model that has a flat line of 0 over the domain of 50 to 0 as a predictor of extinction rate.

```
killemall_ones <- data.frame(
  "NAge"=-seq(from=max(branching.times(lemurs))+2, to=0, length.out = 300),
  "Age"=seq(from=max(branching.times(lemurs))+2, to=0, length.out = 300))
for(i in 50:0){
  new.add=data.frame(
    "o"=c(dnorm(seq(from=max(branching.times(lemurs))+2, to=0, length.out = 300),
               mean = i, sd=0.5))
  )
  names(new.add) <- paste0("Ex_at_",i)
  killemall_ones <- cbind.data.frame(killemall_ones, new.add)
}
```

```
killemall_ones$Null <- 0
```

For example, the pulsed variable that posits an extinction at 34 Ma looks as below.

```
plot(Ex_at_34~NAge, data=killemall_ones, type="l", xlab="Years Ma")
```

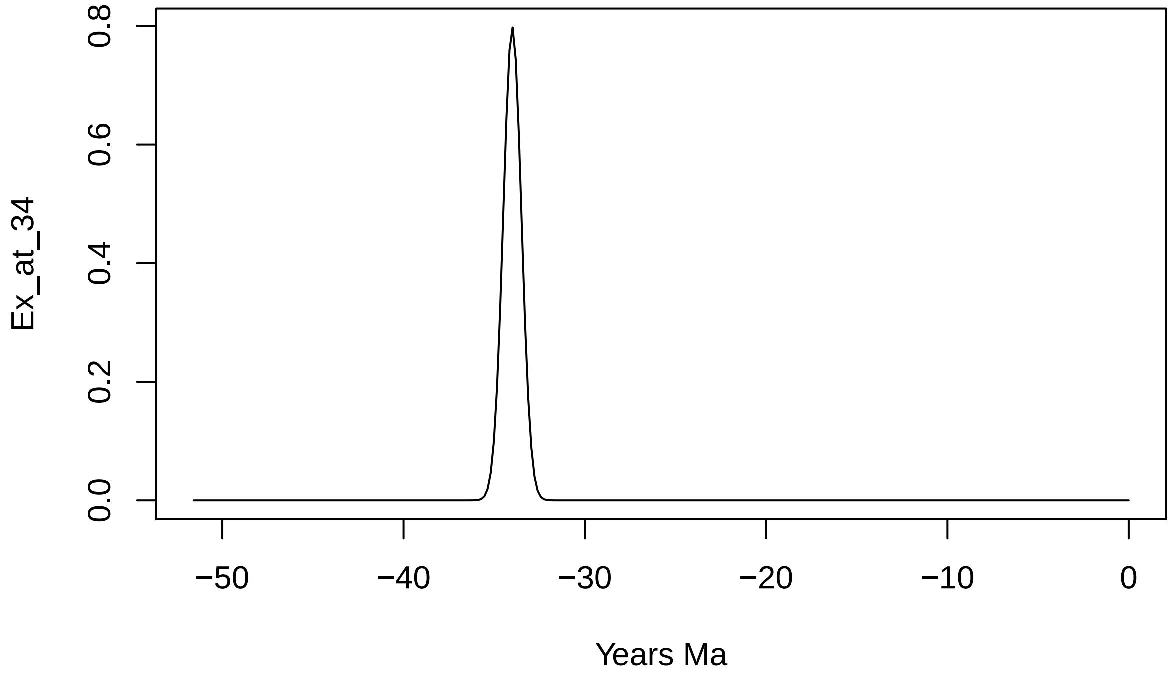

Then we fit 50 models each with the pulse variable as a predictor of extinction rate. We employ the same sampling fraction of incomplete taxon sample as above. Moreover we consider only exponential effects of the environmental pulse variable on the extinction rate as we assume $f_\mu(t, x, y) = y_1 * e^{y_2 * x}$. Herein, the extinction rate function over time is a function of two parameters y_1 and y_2 which are estimated from the data and the pulse variable x. The function must include the variable t to estimate the diversification rate over time which is the speciation rate - extinction rate. For each model we extract the AICc and likelihood. From the AICc we calculate the relative likelihood and Akaike Weight.

```
f=0.8890909
f.lamb<-function(t,x,y){y[1]} #constant speciation rate,
f.mu.exp<-function(t,x,y){y[1]*exp(y[2]*x)}
f.mu.lin<-function(t,x,y){y[1]+(y[2]*x)}
lamb_par_init<-c(0.4)
mu_par_init<-c(0.1,0.05)
tot_time <- max(branching.times(lemurs))
AICcs <- c()
LHs <- c()
AICcs_lin <- c()
LHs_lin <- c()
```

```
for(i in 1:52){
```

```
env_data <- killemall_ones[nrow(killemall_ones):1,c(2,c(2+i))] #make sure first row is time zero
res.exp<-fit_env(lemurs, env_data,tot_time, f.lamb,f.mu.exp,
              lamb_par_init,mu_par_init,f=f,dt=1e-3)
res.lin<-fit_env(lemurs, env_data,tot_time, f.lamb,f.mu.lin,
              lamb_par_init,mu_par_init,f=f,dt=1e-3)
AICcs[i] <- res.exp$aicc
LHs[i] <- res.exp$LH

AICcs_lin[i] <- res.lin$aicc
LHs_lin[i] <- res.lin$LH

}
res=data.frame("AICc"=AICcs,
               "LHs"=LHs,
               "mod"=names(killemall_ones)[3:54])
res_lin=data.frame("AICc"=AICcs_lin,
               "LHs"=LHs_lin,
               "mod"=names(killemall_ones)[3:54])
```

Now we show the Akaike weight profile against the null model.

```
res$year_of_ex<-c(-50:0,NA)
res$dAICc <- res$AICc-min(res$AICc)
res$RelativeLikelihood<-exp(-0.5*res$dAICc)
res$AkaikeWeights<-res$RelativeLikelihood/sum(res$RelativeLikelihood)
#Akaike Weights
plot.new()
plot.window(xlim = c(-50,0), ylim = c(0,0.2))
points(AkaikeWeights~year_of_ex, data=res[1:c(nrow(res)-1),], pch=19, col="#4DAF4A",
     ylim=c(0,0.2))
axis(at = c(round(1.2 * 50), axTicks(1)), side = 1)
axis(at = round(c(-1, axTicks(2)),2), las = 1, side = 2)
mtext(side = 1, text = "Time",line = 3)
mtext(side = 2, text ="Akaike Weights", line = 3)
```

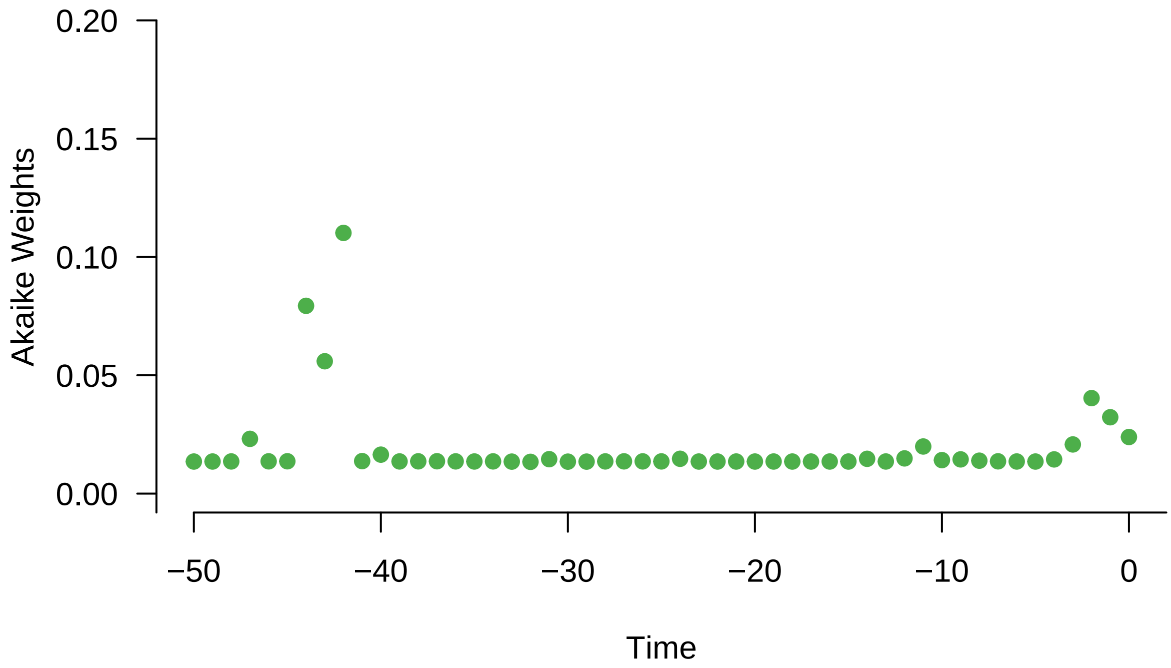

Now show the raw weights and AICc scores.

```
res
```

```
##         AICc       LHs       mod year_of_ex     dAICc RelativeLikelihood
## 1  644.8357 -319.2941 Ex_at_50        -50 4.1911111          0.1230019
## 2  644.8334 -319.2930 Ex_at_49        -49 4.1888363          0.1231419
## 3  644.8296 -319.2911 Ex_at_48        -48 4.1850546          0.1233749
## 4  643.7658 -318.7592 Ex_at_47        -47 3.1212382          0.2100060
## 5  644.8226 -319.2876 Ex_at_46        -46 4.1779985          0.1238110
## 6  644.8193 -319.2860 Ex_at_45        -45 4.1747619          0.1240115
## 7  641.3005 -317.5266 Ex_at_44        -44 0.6559632          0.7203763
## 8  642.0001 -317.8763 Ex_at_43        -43 1.3555183          0.5077535
## 9  640.6446 -317.1986 Ex_at_42        -42 0.0000000          1.0000000
## 10 644.8098 -319.2812 Ex_at_41        -41 4.1652248          0.1246043
## 11 644.4459 -319.0992 Ex_at_40        -40 3.8013323          0.1494690
## 12 644.8280 -319.2903 Ex_at_39        -39 4.1834808          0.1234721
## 13 644.8226 -319.2876 Ex_at_38        -38 4.1779905          0.1238115
## 14 644.8177 -319.2851 Ex_at_37        -37 4.1731496          0.1241115
## 15 644.8248 -319.2887 Ex_at_36        -36 4.1801906          0.1236754
## 16 644.8313 -319.2920 Ex_at_35        -35 4.1867796          0.1232686
## 17 644.8259 -319.2892 Ex_at_34        -34 4.1813202          0.1236055
## 18 644.8406 -319.2966 Ex_at_33        -33 4.1960748          0.1226970
## 19 644.8548 -319.3037 Ex_at_32        -32 4.2102163          0.1218325
## 20 644.6928 -319.2227 Ex_at_31        -31 4.0482295          0.1321107
## 21 644.8418 -319.2972 Ex_at_30        -30 4.1972271          0.1226263
## 22 644.8412 -319.2969 Ex_at_29        -29 4.1966521          0.1226616
## 23 644.8269 -319.2897 Ex_at_28        -28 4.1823334          0.1235429
```

```
## 24 644.8232 -319.2879 Ex_at_27        -27 4.1786136          0.1237729
## 25 644.8252 -319.2889 Ex_at_26        -26 4.1806194          0.1236488
## 26 644.8268 -319.2897 Ex_at_25        -25 4.1821830          0.1235522
## 27 644.6704 -319.2115 Ex_at_24        -24 4.0258427          0.1335978
## 28 644.8352 -319.2939 Ex_at_23        -23 4.1906514          0.1230302
## 29 644.8349 -319.2937 Ex_at_22        -22 4.1903264          0.1230502
## 30 644.8346 -319.2936 Ex_at_21        -21 4.1900536          0.1230669
## 31 644.8347 -319.2937 Ex_at_20        -20 4.1901748          0.1230595
## 32 644.8339 -319.2932 Ex_at_19        -19 4.1893153          0.1231124
## 33 644.8356 -319.2941 Ex_at_18        -18 4.1910540          0.1230054
## 34 644.8355 -319.2940 Ex_at_17        -17 4.1909060          0.1230145
## 35 644.8342 -319.2934 Ex_at_16        -16 4.1896342          0.1230928
## 36 644.8362 -319.2944 Ex_at_15        -15 4.1915952          0.1229721
## 37 644.6730 -319.2128 Ex_at_14        -14 4.0284621          0.1334230
## 38 644.8322 -319.2924 Ex_at_13        -13 4.1875832          0.1232191
## 39 644.6478 -319.2002 Ex_at_12        -12 4.0032499          0.1351155
## 40 644.0661 -318.9093 Ex_at_11        -11 3.4215121          0.1807291
## 41 644.7485 -319.2505 Ex_at_10        -10 4.1038924          0.1284846
## 42 644.7042 -319.2284  Ex_at_9         -9 4.0596490          0.1313586
## 43 644.7822 -319.2674  Ex_at_8         -8 4.1375867          0.1263381
## 44 644.8219 -319.2873  Ex_at_7         -7 4.1773576          0.1238507
## 45 644.8329 -319.2928  Ex_at_6         -6 4.1883734          0.1231704
## 46 644.8359 -319.2942  Ex_at_5         -5 4.1913238          0.1229888
## 47 644.7059 -319.2292  Ex_at_4         -4 4.0613073          0.1312497
## 48 643.9804 -318.8665  Ex_at_3         -3 3.3358457          0.1886385
## 49 642.6530 -318.2028  Ex_at_2         -2 2.0083947          0.3663386
## 50 643.0999 -318.4262  Ex_at_1         -1 2.4552840          0.2929826
## 51 643.6998 -318.7262  Ex_at_0          0 3.0552740          0.2170479
## 52 644.8359 -319.2942     Null         NA 4.1913488          0.1229873
##    AkaikeWeights
## 1     0.01355114
## 2     0.01356657
## 3     0.01359224
## 4     0.02313641
## 5     0.01364028
## 6     0.01366237
## 7     0.07936400
## 8     0.05593931
## 9     0.11017021
## 10    0.01372768
## 11    0.01646703
## 12    0.01360294
## 13    0.01364034
## 14    0.01367339
## 15    0.01362534
## 16    0.01358052
## 17    0.01361765
## 18    0.01351755
## 19    0.01342231
## 20    0.01455467
## 21    0.01350977
## 22    0.01351365
## 23    0.01361075
## 24    0.01363609
```

```
## 25     0.01362242
## 26     0.01361177
## 27     0.01471850
## 28     0.01355426
## 29     0.01355646
## 30     0.01355831
## 31     0.01355749
## 32     0.01356332
## 33     0.01355153
## 34     0.01355253
## 35     0.01356115
## 36     0.01354786
## 37     0.01469924
## 38     0.01357507
## 39     0.01488571
## 40     0.01991096
## 41     0.01415518
## 42     0.01447180
## 43     0.01391870
## 44     0.01364465
## 45     0.01356971
## 46     0.01354970
## 47     0.01445981
## 48     0.02078234
## 49     0.04035960
## 50     0.03227796
## 51     0.02391222
## 52     0.01354953
```

We see evidence for the models that posit an extinction happening near 42-44 Ma.

Using the Akaike Weights above the best model with the extinction at 42 Ma is `0.11017021/0.01354953= 8.13` times more likely than the null model. And the probability that the model with the extinction at 34 Ma is preferred over the null model is equal to `0.11017021/(0.11017021+0.01354953) = 0.89`.

Likewise, the models that posit an extinction occurring between 44 and 42 Ma are collectively 18.12 more likely than the null model (`sum(res$AkaikeWeights[7:9])/res$AkaikeWeights[nrow(res)]`). The probability that the models positing extinctions to occur between 44 and 42 are preferred over the null model is 0.9476899 (`sum(res$AkaikeWeights[7:9])/sum(sum(res$AkaikeWeights[7:9]), res$AkaikeWeights[nrow(res)])`).

We can also consider linear effects of the pulse variables. We can show the Akaike weight profile against the null model, using the linear effect.

```
res_lin$dAICc <- res_lin$AICc-min(res_lin$AICc)
res_lin$RelativeLikelihood<-exp(-0.5*res_lin$dAICc)
res_lin$AkaikeWeights<-res_lin$RelativeLikelihood/sum(res_lin$RelativeLikelihood)
res_lin$year_of_ex<--50:1
#Akaike Weights
plot.new()
plot.window(xlim = c(-50,0), ylim = c(0,0.2))
points(AkaikeWeights~year_of_ex, data=res_lin[1:c(nrow(res_lin)-1),], pch=19, col="#4DAF4A",
     ylim=c(0,0.2))
axis(at = c(round(1.2 * 50), axTicks(1)), side = 1)
axis(at = round(c(-1, axTicks(2)),2), las = 1, side = 2)
mtext(side = 1, text = "Time",line = 3)
```

```r
mtext(side = 2, text ="Akaike Weights", line = 3)
title("Linear effect")
```

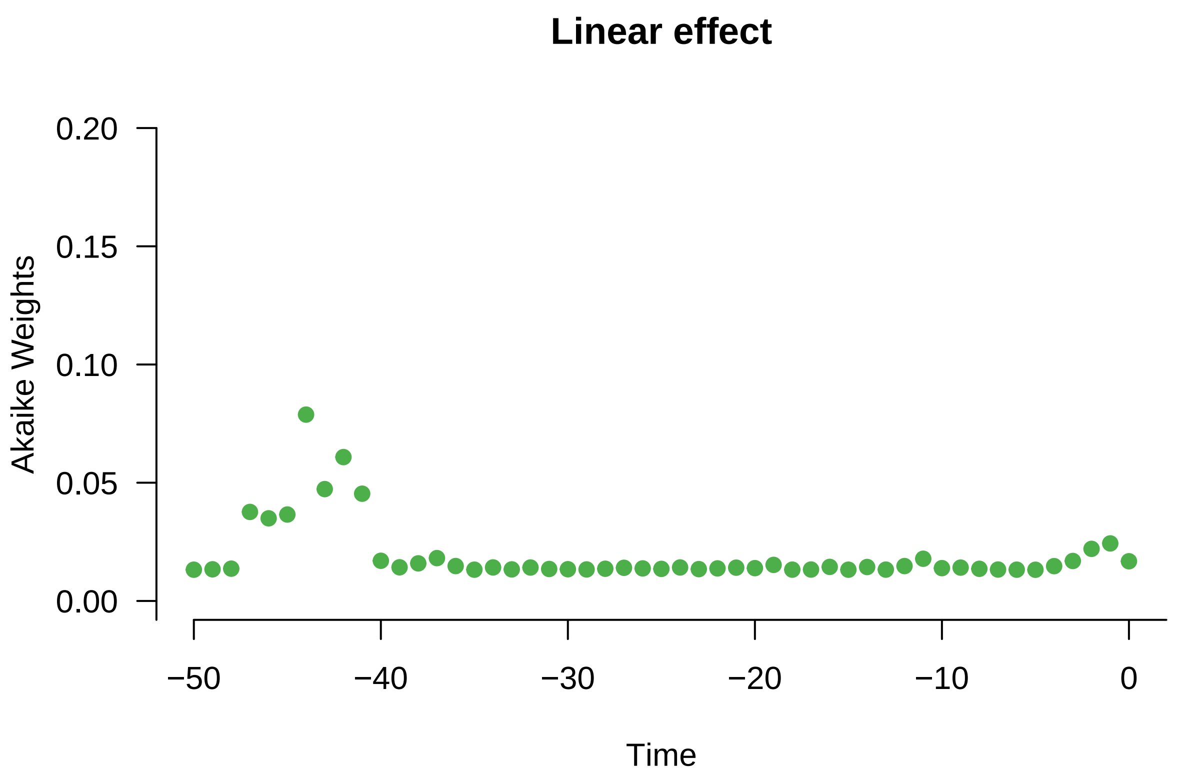

Now show the raw weights and AICc scores.

```r
res_lin
```

```
##        AICc       LHs       mod     dAICc RelativeLikelihood AkaikeWeights
## 1  644.8332 -319.2929 Ex_at_50 3.5718931          0.1676383    0.01321168
## 2  644.8080 -319.2803 Ex_at_49 3.5466510          0.1697675    0.01337949
## 3  644.7665 -319.2596 Ex_at_48 3.5052256          0.1733205    0.01365950
## 4  642.7391 -318.2458 Ex_at_47 1.4778109          0.4776364    0.03764284
## 5  642.8875 -318.3200 Ex_at_46 1.6261480          0.4434927    0.03495195
## 6  642.7981 -318.2754 Ex_at_45 1.5368389          0.4637455    0.03654808
## 7  641.2613 -317.5069 Ex_at_44 0.0000000          1.0000000    0.07881065
## 8  642.2821 -318.0173 Ex_at_43 1.0207482          0.6002710    0.04730774
## 9  641.7787 -317.7656 Ex_at_42 0.5173959          0.7720562    0.06084625
## 10 642.3660 -318.0593 Ex_at_41 1.1047173          0.5755906    0.04536267
## 11 644.3299 -319.0412 Ex_at_40 3.0686148          0.2156050    0.01699197
## 12 644.6851 -319.2188 Ex_at_39 3.4238139          0.1805212    0.01422699
## 13 644.4630 -319.1078 Ex_at_38 3.2016566          0.2017294    0.01589842
## 14 644.2015 -318.9770 Ex_at_37 2.9401506          0.2299082    0.01811921
## 15 644.6117 -319.1821 Ex_at_36 3.3503486          0.1872755    0.01475931
## 16 644.8312 -319.2919 Ex_at_35 3.5699133          0.1678043    0.01322477
## 17 644.6926 -319.2226 Ex_at_34 3.4312678          0.1798497    0.01417407
## 18 644.8127 -319.2826 Ex_at_33 3.5514181          0.1693633    0.01334763
## 19 644.6980 -319.2253 Ex_at_32 3.4366634          0.1793651    0.01413588
```

```
## 20 644.7876 -319.2701 Ex_at_31 3.5262752          0.1715059    0.01351649
## 21 644.8022 -319.2774 Ex_at_30 3.5409427          0.1702527    0.01341773
## 22 644.8164 -319.2845 Ex_at_29 3.5551216          0.1690500    0.01332294
## 23 644.7719 -319.2622 Ex_at_28 3.5105709          0.1728579    0.01362304
## 24 644.7151 -319.2339 Ex_at_27 3.4538265          0.1778325    0.01401509
## 25 644.7521 -319.2524 Ex_at_26 3.4908319          0.1745724    0.01375816
## 26 644.7811 -319.2668 Ex_at_25 3.5197484          0.1720665    0.01356067
## 27 644.6944 -319.2235 Ex_at_24 3.4331212          0.1796831    0.01416094
## 28 644.7956 -319.2741 Ex_at_23 3.5342608          0.1708225    0.01346263
## 29 644.7511 -319.2518 Ex_at_22 3.4897654          0.1746655    0.01376550
## 30 644.7062 -319.2294 Ex_at_21 3.4449360          0.1786248    0.01407753
## 31 644.7339 -319.2432 Ex_at_20 3.4725820          0.1761726    0.01388428
## 32 644.5463 -319.1494 Ex_at_19 3.2849991          0.1934958    0.01524953
## 33 644.8299 -319.2912 Ex_at_18 3.5685779          0.1679164    0.01323360
## 34 644.8233 -319.2879 Ex_at_17 3.5619919          0.1684703    0.01327725
## 35 644.6612 -319.2069 Ex_at_16 3.3998619          0.1826961    0.01439840
## 36 644.8326 -319.2926 Ex_at_15 3.5712843          0.1676893    0.01321571
## 37 644.6671 -319.2099 Ex_at_14 3.4058360          0.1821512    0.01435546
## 38 644.8272 -319.2899 Ex_at_13 3.5658623          0.1681446    0.01325158
## 39 644.6129 -319.1828 Ex_at_12 3.3516329          0.1871553    0.01474983
## 40 644.2315 -318.9921 Ex_at_11 2.9702263          0.2264767    0.01784878
## 41 644.7339 -319.2432 Ex_at_10 3.4725537          0.1761751    0.01388447
## 42 644.7027 -319.2276  Ex_at_9 3.4413993          0.1789409    0.01410245
## 43 644.7764 -319.2645  Ex_at_8 3.5150694          0.1724695    0.01359244
## 44 644.8225 -319.2875  Ex_at_7 3.5611501          0.1685412    0.01328284
## 45 644.8307 -319.2917  Ex_at_6 3.5694421          0.1678439    0.01322788
## 46 644.8359 -319.2942  Ex_at_5 3.5745940          0.1674121    0.01319385
## 47 644.6166 -319.1846  Ex_at_4 3.3553156          0.1868110    0.01472270
## 48 644.3409 -319.0468  Ex_at_3 3.0796346          0.2144203    0.01689860
## 49 643.8105 -318.7816  Ex_at_2 2.5492311          0.2795384    0.02203060
## 50 643.6114 -318.6820  Ex_at_1 2.3501201          0.3088004    0.02433676
## 51 644.3539 -319.0532  Ex_at_0 3.0925975          0.2130350    0.01678943
## 52 644.8359 -319.2942     Null 3.5746137          0.1674104    0.01319372
##    year_of_ex
## 1         -50
## 2         -49
## 3         -48
## 4         -47
## 5         -46
## 6         -45
## 7         -44
## 8         -43
## 9         -42
## 10        -41
## 11        -40
## 12        -39
## 13        -38
## 14        -37
## 15        -36
## 16        -35
## 17        -34
## 18        -33
## 19        -32
## 20        -31
```

```
## 21         -30
## 22         -29
## 23         -28
## 24         -27
## 25         -26
## 26         -25
## 27         -24
## 28         -23
## 29         -22
## 30         -21
## 31         -20
## 32         -19
## 33         -18
## 34         -17
## 35         -16
## 36         -15
## 37         -14
## 38         -13
## 39         -12
## 40         -11
## 41         -10
## 42          -9
## 43          -8
## 44          -7
## 45          -6
## 46          -5
## 47          -4
## 48          -3
## 49          -2
## 50          -1
## 51           0
## 52           1
```

We see that the inference from linear and exponential effects is qualitatively similar. We continue with the exponential effects.

Now to make it more clear what the model that posits the pulsed environmental variable with a peak at 42 Ma is actually predicting, we can make use of the `plot_fit_env()` function as in Morlon et al. 2016 (Fig. 6). This will clarify what the model is actually saying, and it shall become clear that while this model fares better than other models (as shown above), it is unlikely to be right. So, as they say, all models are wrong, but some are useful.

Because the looping process above overwrote the models, we must re-create the best model with a pulse at 42 being an exponential predictor of the extinction rate.

```
env_data_42 <- killemall_ones[nrow(killemall_ones):1,c("Age","Ex_at_42")]
res.exp_42<-fit_env(lemurs, env_data_42,tot_time, f.lamb,f.mu.exp,
              lamb_par_init,mu_par_init,f=f,dt=1e-3)
#plot_fit_env(res.exp_42, env_data_42, 55)
#minor changes necessary because speciation rate is constant
t <- seq(0, 55, length.out = 100)

    plot(-t, rep(res.exp_42$f.lamb(t), length(t)), type = "l", xlab = "time",
        ylab = "speciation rate", main = "Fitted speciation rate")
```

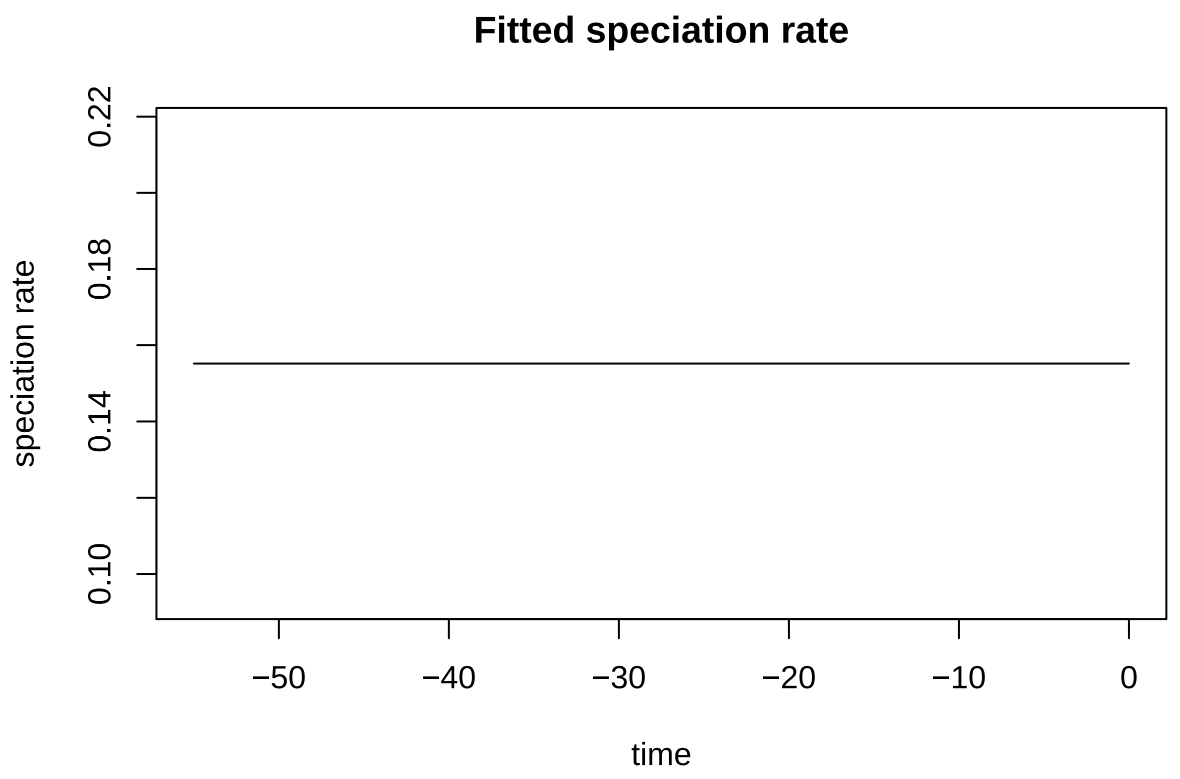


```
df <- smooth.spline(env_data_42[, 1], env_data_42[, 2])$df
spline_result <- pspline::sm.spline(env_data_42[, 1], env_data_42[, 2],
    df = df)
env_func <- function(t) {
    predict(spline_result, t)
}

plot(env_func(t), rep(res.exp_42$f.lamb(t), length(t)), type = "l", xlab = "Environmental data",
    ylab = "speciation rate", main = "Fitted speciation rate")
```

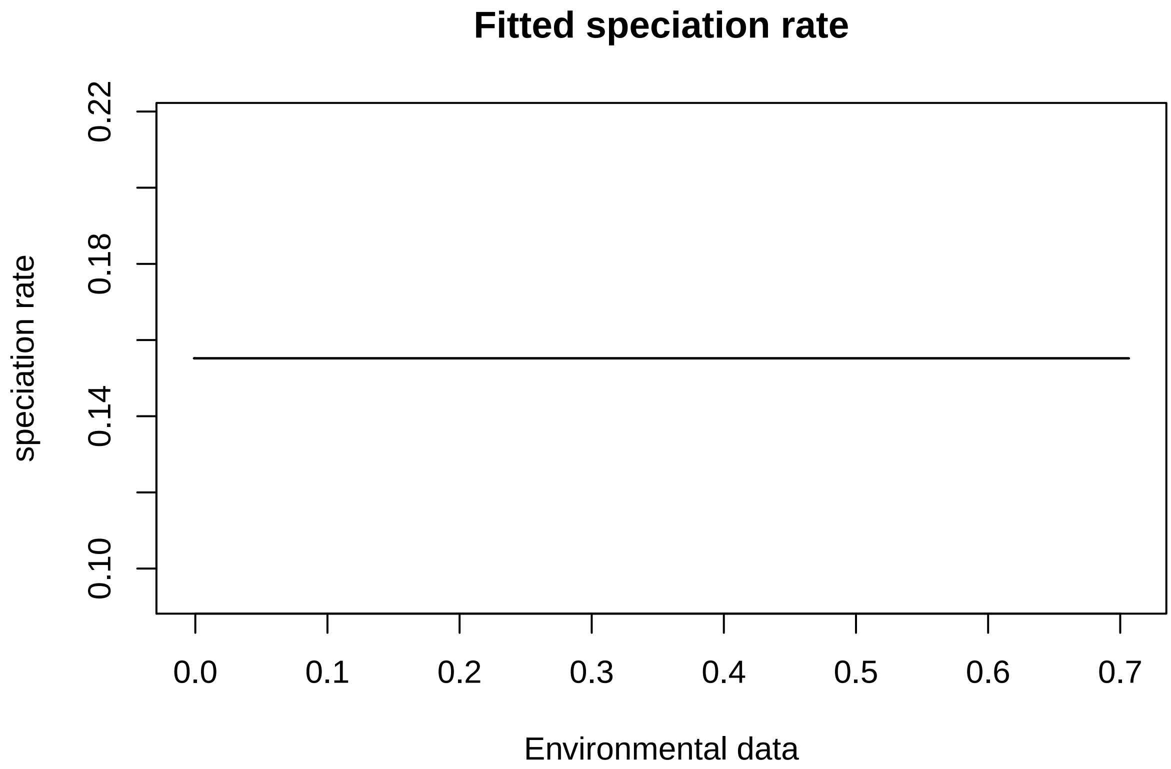


```
#    if ("f.mu" %in% attributes(fit.env)$names) {

        plot(-t, res.exp_42$f.mu(t), type = "l", xlab = "time",
            ylab = "extinction rate", main = "Fitted extinction rate")
```

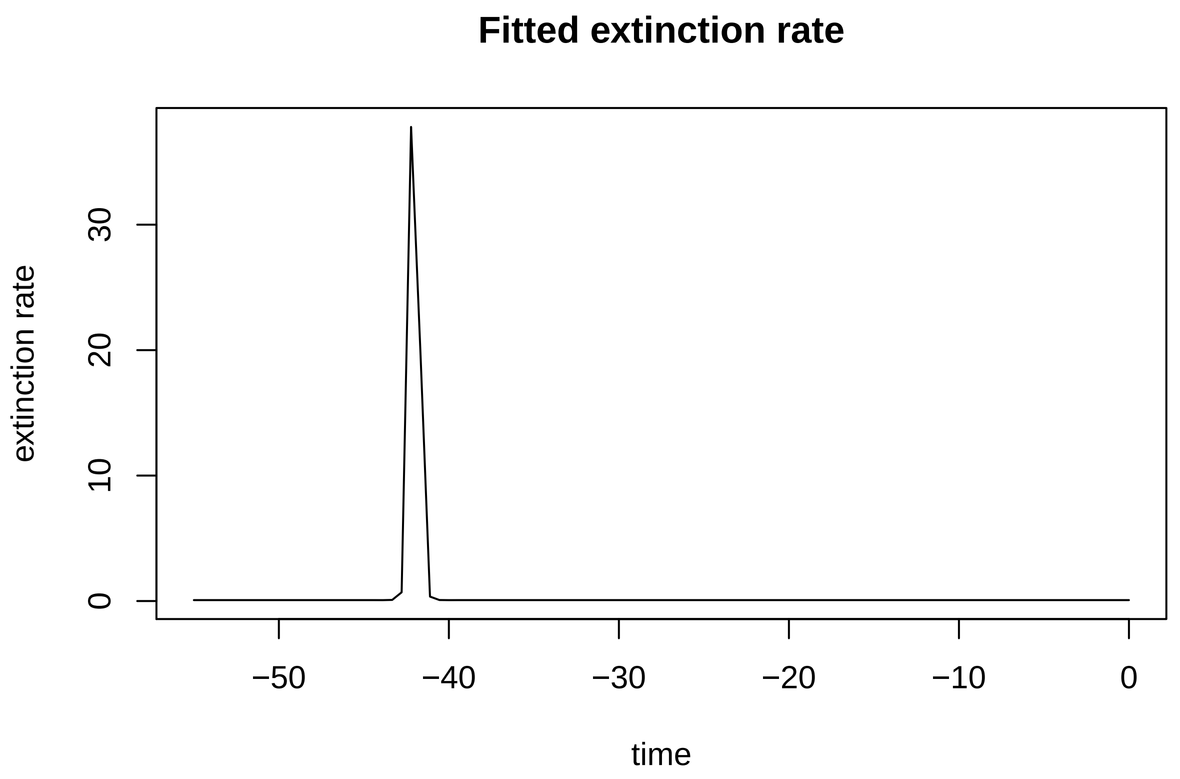


```
plot(env_func(t), res.exp_42$f.mu(t), type = "l",
     xlab = "Environmental data", ylab = "extinction rate",
     main = "Fitted extinction rate")
```

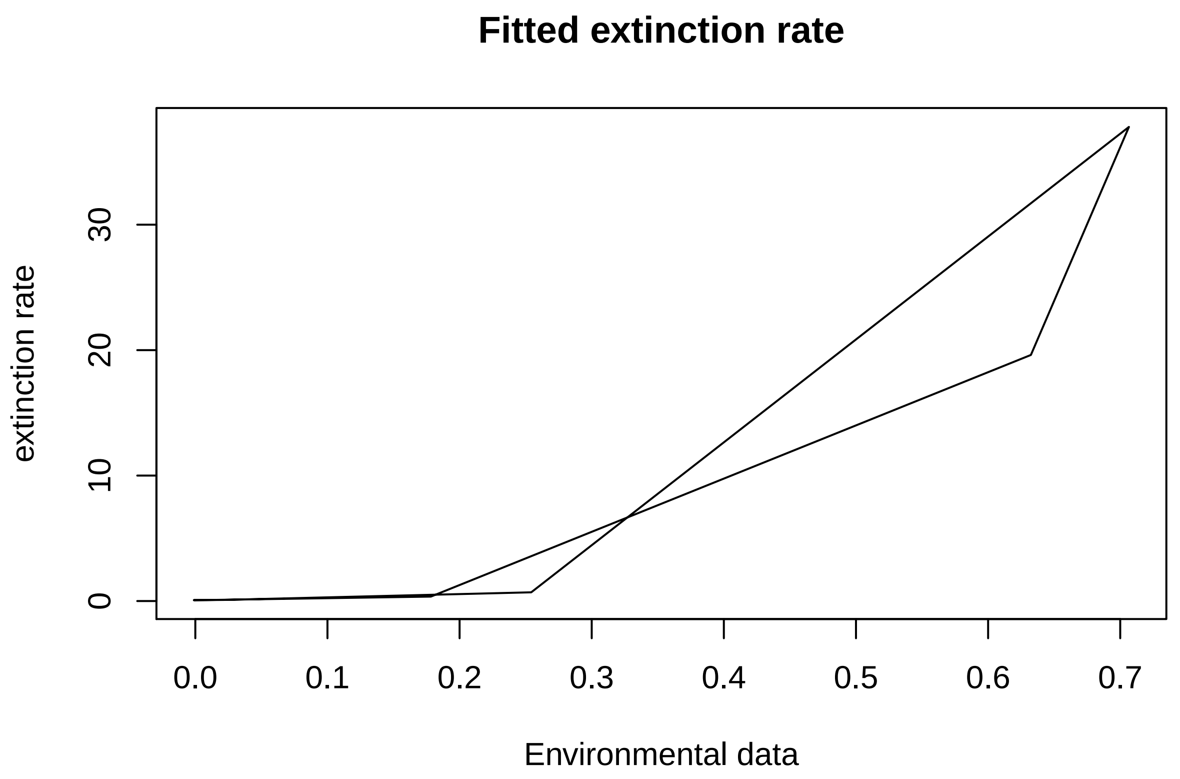


```
r <- function(t) {
    res.exp_42$f.lamb(t) - res.exp_42$f.mu(t)
}

plot(-t, r(t), type = "l", xlab = "time",
    ylab = "net diversification rate", main = "Fitted net diversification rate")
```

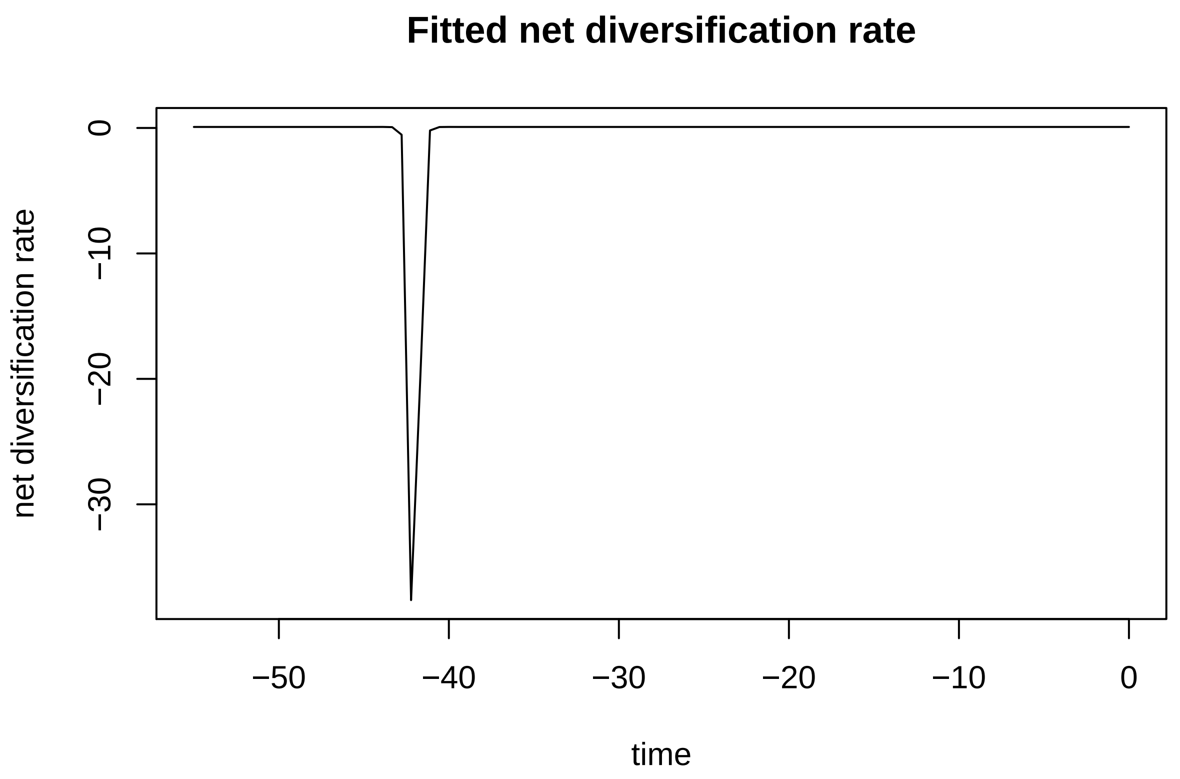


```
plot(env_func(t), r(t), type = "l", xlab = "Environmental data",
    ylab = "net diversification rate", main = "Fitted net diversification rate")
```

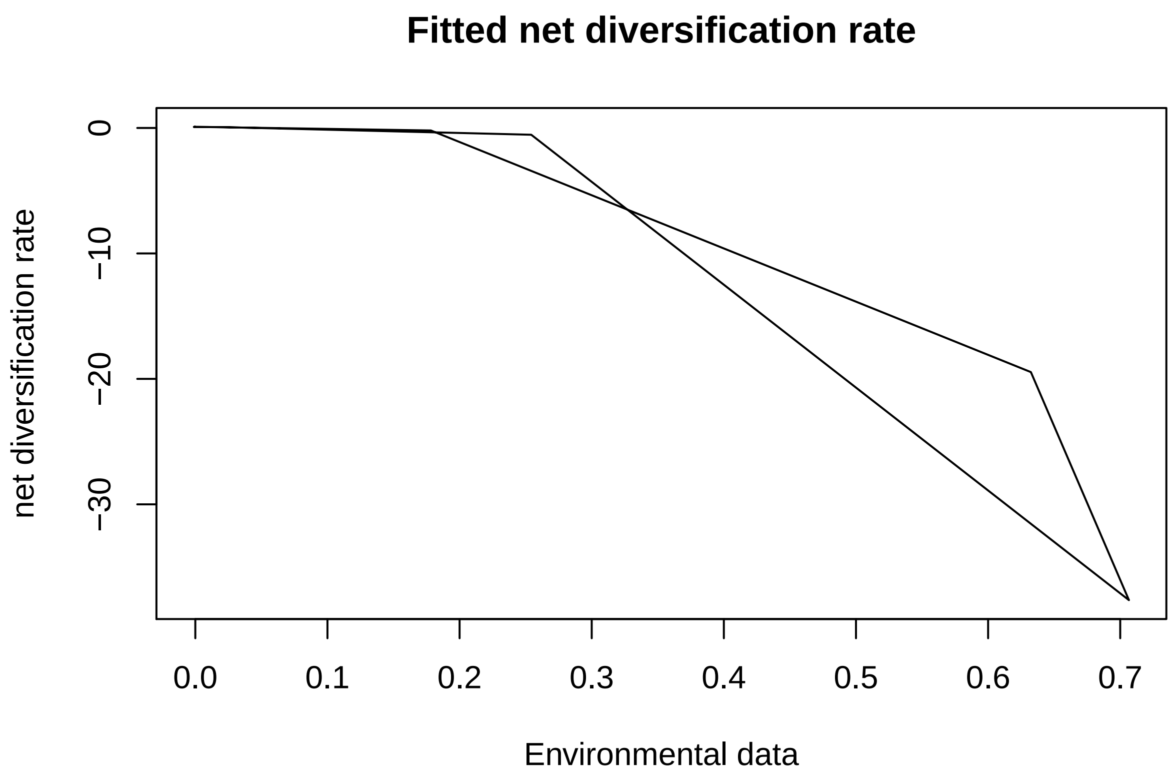


```
#    }
```

The plots above describe exactly what we would expect, given the way we set up the model.

We see that 1) diversification rate is constant 2) diversification rate is not correlated with the environmental pulse 3) We are saying that the extinction rate spiked extremely high at 42 Ma where 4) high values of the pulse variable are found - the fourth plot shows just that the few high values of environmental pulse variable correspond to high values of extinction rate 5) the net diversification rate plummeted at the time when the spiking extinction rate was observed and 6) the high values of environmental variable show when net diversification rates are low.

Consider Temperature and Rate of Change as Predictor of Extinction Rate

We also consider temperature and its rate of change as predictor variables for extinction rate. We consider both linear and exponential functions of each.

```
lamb_par_init<-c(0.4) #use the previous inits for the constant rate of lambda and varying rate of mu
mu_par_init<-c(0.1,0.05)
f.mu.lin<-function(t,x,y){y[1]+y[2]*x} #linear dependence of extinction rate on P(Mass Extinction)
f.lamb<-function(t,x,y){y[1]} #constant speciation rate,
f.mu.exp<-function(t,x,y){y[1]*exp(y[2]*x)}

newdata=data.frame("Age"=seq(0, 70 ,length.out = 700))
newdata$predY <- predict(G1, type = "response", newdata = newdata)
InfTemp2$Nage=-InfTemp2$Age
newdata$Nage=-newdata$Age
newdata$predY_diff <- c(0,diff(newdata$predY)/diff(newdata$Nage))
```

```r
newdata$Null=c(rep(0.001,nrow(newdata)))
#use raw temp
env_data=newdata[,which(names(newdata) %in% c("Age", "predY"))]
res1.lin<-fit_env(lemurs, env_data,tot_time, f.lamb,f.mu.lin,
              lamb_par_init,mu_par_init,f=f,dt=1e-3)
res1.exp<-fit_env(lemurs, env_data,tot_time, f.lamb,f.mu.exp,
              lamb_par_init,mu_par_init,f=f,dt=1e-3)
#use rate
env_data=newdata[,which(names(newdata) %in% c("Age", "predY_diff"))]
res2.lin<-fit_env(lemurs, env_data,tot_time, f.lamb,f.mu.lin,
              lamb_par_init,mu_par_init,f=f,dt=1e-3)
res2.exp<-fit_env(lemurs, env_data,tot_time, f.lamb,f.mu.exp,
              lamb_par_init,mu_par_init,f=f,dt=1e-3)
#the models with nothing
env_data <- newdata[,which(names(newdata) %in% c("Age", "Null"))]
res3.lin<-fit_env(lemurs, env_data,tot_time, f.lamb,f.mu.lin,
              lamb_par_init,mu_par_init,f=f,dt=1e-3)

mods <- ls()[grep(pattern = "res[1-3].[lin|exp]",x=ls())]
aicc.table3 <- c()
for(i in 1:length(mods)){
  g=get(mods[i])
  aicc.table3 <- rbind(aicc.table3, (cbind(mods[i], g$aicc)))
}
aicc.table3 <- as.data.frame(aicc.table3)
aicc.table3$Extinctions <- c("Temperature", "Temperature",
                             "Rate of Temperature Change", "Rate of Temperature Change",
                             "Null Model")
aicc.table3$Effect <- c(rep(c("Linear", "Exponential"), 2), "")
aicc.table3 <- aicc.table3[,c(4,3,2)]
```

Effect	Model	AICc
Linear	Temperature	642.9363
Exponential	Temperature	643.0027
Linear	Rate of Temperature Change	644.8625
Exponential	Rate of Temperature Change	644.0604
	Null Model	644.8359

The models with temperature influencing extinction rate are not well supported.

In order to add all RPANDA results together, we need a plot that shows the raw AICc scores.

```r
aicc.table3$year_of_ex<-5:1
aicc.table$year_of_ex<-6:9
expression(paste(Delta," Temp. (Lin.)"))
```

```
## expression(paste(Delta, " Temp. (Lin.)"))
```

```r
xlabels<-c("Null", " ", " ",
                "Temp. (Exp)", "Temp. (Lin)",
           "ScstEcst","SvarEcst", "ScstEvar", "SvarEvar"
           )
xlabelspositions<-c(1:9)
```

```
par(mar=c(5.5,4.1,1,2))

plot.new()


plot.window(xlim = c(-50,18), ylim = c(635,645))


#polygon(c(-31.5, -31.5,-40.5,-40.5), c(635,645,645,635), col = "#DDDDDD", border = "#DDDDDD")
#polygon(c(-43.5, -43.5,-45.5,-45.5), c(635,645,645,635), col = "#DDDDDD", border = "#DDDDDD")

points(AICc~year_of_ex, data=res[1:c(nrow(res)-1),], pch=19, col="black")
aicc.table3$year_of_ex<-c(10, 8, 6, 4, 2)
points(AICc~year_of_ex, data=aicc.table3, pch=19, col="gray80")
aicc.table$year_of_ex<-c(12, 14, 16, 18)
points(AICc~year_of_ex, data=aicc.table, pch=1, col="black")

axis(at = c(-50, -40, -30, -20, -10, 0), side = 1)
xlabelspositions<-seq(from=2, to=18, by=2)
text(x=xlabelspositions, y=par()$usr[3]-0.125,labels=xlabels, srt=90, adj=1, xpd=TRUE)
text(x=xlabelspositions[2], y=par()$usr[3]-0.125,labels=expression(paste(Delta," Temp. (Exp)")),
     srt=90, adj=1, xpd=TRUE)
text(x=xlabelspositions[3], y=par()$usr[3]-0.125,labels=expression(paste(Delta," Temp. (Lin)")),
     srt=90, adj=1, xpd=TRUE)

####r right here tight now
axis(at = round(c(-1, axTicks(2)),2), las = 1, side = 2)
mtext(side = 1, text = "Time",line = 3)
mtext(side = 2, text ="AICc", line = 3)
text(-47.5,542,labels="a")

abline(h=min(res$AICc), lty=2)
abline(h=min(res$AICc)+4, lty=2)
```

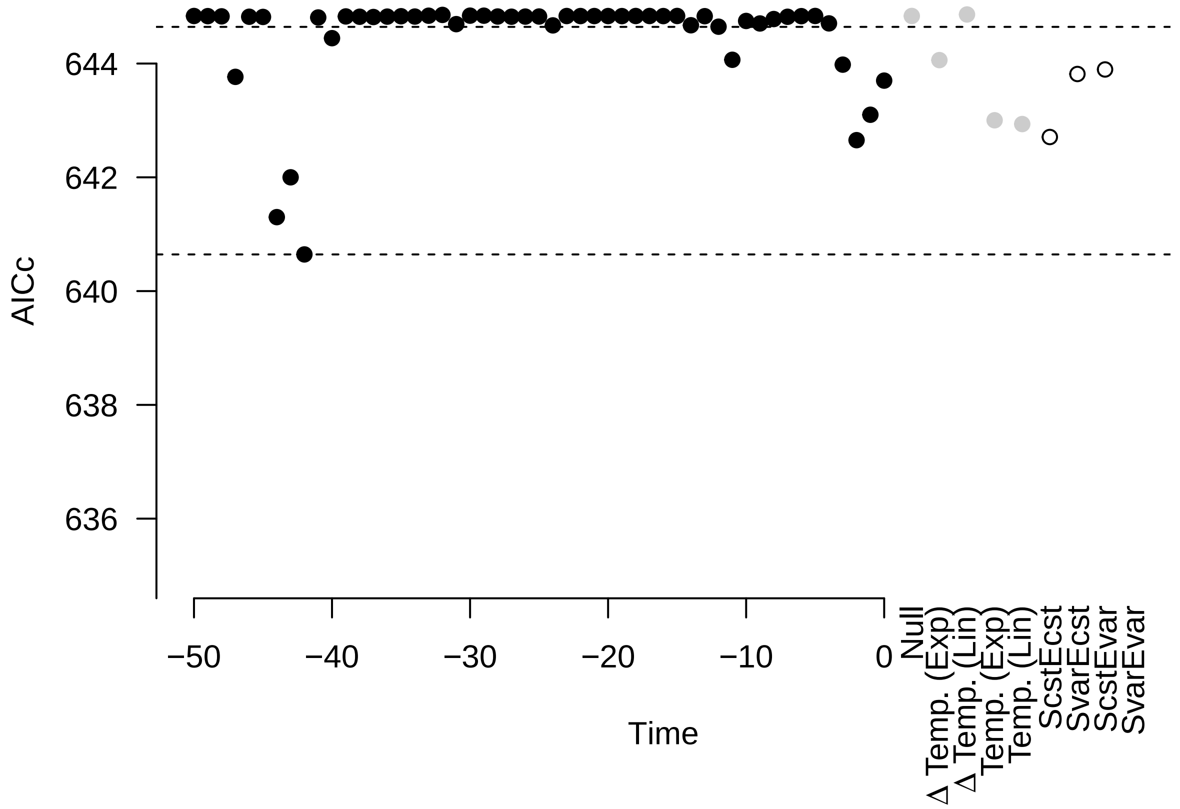


```
ftw<-dev.off()
```

From this it is abundantly clear that the model with an environmental pulse variable with a peak at near 33 Ma is better than most models. The models that posit time-variant speciation and or extinction rates (points with white fill), as well as the models that posit temperature or the rate of change of temperature affecting extinction rates in linear or logarithmic fashions (gray points) fall within the range of poor model fits that are beset by model uncertainty. Dashed lines indicate the range of model AICc scores that fall between 0 and 4 AICc scores from the best-fit model. Models within that range are the best fit models, but likely indistinguishable (gray shading). Models above that range are moderately worse than the best model. The best model posits a pulse variable with a peak at 33 Ma affecting the extinction rate in an exponential manner.

Now finally because some authors have wondered if not perhaps the extinction rate experienced a pulse-like change at some year but rather the speciation rate experienced a pulse-like change, we now repeat our analysis above considering an effect of our simulated environmental pulse variables on the speciation rate. In keeping the speciation rate dependent on the environmental variable, we keep the extinction rate constant. For completeness, we also consider the identical true environmental variables (Temperature and its rate of change) above as predictors of speciation rate.

```
f.lamb.exp2<-function(t,x,y){y[1]*exp(y[2]*x)}
f.lamb.lin2<-function(t,x,y){y[1]*(y[2]*x)}
f.mu2<-function(t,x,y){y[1]}
lamb_par_init2<-c(0.4, 0.05)
mu_par_init2<-c(0.1)

newdata=data.frame("Age"=seq(0, 70 ,length.out = 700))
newdata$predY <- predict(G1, type = "response", newdata = newdata)
```

```r
InfTemp2$Nage=-InfTemp2$Age
newdata$Nage=-newdata$Age
newdata$predY_diff <- c(0,diff(newdata$predY)/diff(newdata$Nage))

newdata$Null=c(rep(0.001,nrow(newdata)))
#use raw temp

#start here####################

env_data=newdata[,which(names(newdata) %in% c("Age", "predY"))]
res1.lin_spec<-fit_env(lemurs, env_data,tot_time, f.lamb.lin2,f.mu2,
              lamb_par_init2,mu_par_init2,f=f,dt=1e-3)
res1.exp_spec<-fit_env(lemurs, env_data,tot_time, f.lamb.exp2,f.mu2,
              lamb_par_init2,mu_par_init2,f=f,dt=1e-3)
#use rate
env_data=newdata[,which(names(newdata) %in% c("Age", "predY_diff"))]
res2.lin_spec<-fit_env(lemurs, env_data,tot_time, f.lamb.lin2,f.mu2,
              lamb_par_init2,mu_par_init2,f=f,dt=1e-3)
res2.exp_spec<-fit_env(lemurs, env_data,tot_time, f.lamb.exp2,f.mu2,
              lamb_par_init2,mu_par_init2,f=f,dt=1e-3)
#the models with nothing
env_data <- newdata[,which(names(newdata) %in% c("Age", "Null"))]
res3.lin_spec<-fit_env(lemurs, env_data,tot_time, f.lamb.lin2,f.mu2,
              lamb_par_init2,mu_par_init2,f=f,dt=1e-3)

mods2 <- ls()[grep(pattern = "res[1-3].[lin|exp]",x=ls())]
mods2<-mods2[grepl(pattern = "spec", x=mods2)]
aicc.table3_spec <- c()
for(i in 1:length(mods2)){
  g=get(mods2[i])
  aicc.table3_spec <- rbind(aicc.table3_spec, (cbind(mods2[i], g$aicc)))
}
aicc.table3_spec <- as.data.frame(aicc.table3_spec)
aicc.table3_spec$Speciation <- c("Temperature", "Temperature",
                             "Rate of Temperature Change", "Rate of Temperature Change",
                             "Null Model")
aicc.table3_spec$Effect <- c(rep(c("Linear", "Exponential"), 2), "")
aicc.table3_spec <- aicc.table3_spec[,c(4,3,2)]
```

Effect	Model	AICc
Linear	Temperature	644.5719
Exponential	Temperature	670.7991
Linear	Rate of Temperature Change	644.1648
Exponential	Rate of Temperature Change	647.1290
	Null Model	644.8359

```r
f.lamb2<-function(t,x,y){y[1]*exp(y[2]*x)}
f.mu.exp2<-function(t,x,y){y[1]}
lamb_par_init2<-c(0.4, 0.05)
mu_par_init2<-c(0.1)
AICcs2 <- c()
```

```
LHs2 <- c()
```

```
for(i in 1:52){
env_data <- killemall_ones[nrow(killemall_ones):1,c(2,c(2+i))] #make sure first row is time zero
res.exp2<-fit_env(lemurs, env_data,tot_time, f.lamb2,f.mu.exp2,
              lamb_par_init2,mu_par_init2,f=f,dt=1e-3)
AICcs2[i] <- res.exp2$aicc
LHs2[i] <- res.exp2$LH
}
res2=data.frame("AICc"=AICcs2,
               "LHs"=LHs2,
               "mod"=names(killemall_ones)[3:54])
saveRDS(res2, "sub3/h1/res_test2.RDS")
```

```
res2$dAICc <- res2$AICc-min(res2$AICc)
res2$RelativeLikelihood<-exp(-0.5*res2$dAICc)
res2$AkaikeWeights<-res2$RelativeLikelihood/sum(res2$RelativeLikelihood)
res2$year_of_ex<-c(-50:0, NA)
aicc.table3_spec$year_of_ex<-c(10,8,6,4,2)
#Akaike


plot.new()
plot.window(xlim = c(-50,9), ylim = c(635, 672))
points(AICc~year_of_ex, data=res2[1:c(nrow(res2)-1),], pch=19, col="#4DAF4A")
points(AICc~year_of_ex, data=aicc.table3_spec, pch=19, col="red")
title("Variables Influencing Speciation Rate")

axis(at = c(-50, -40, -30, -20, -10, 0), side = 1)
xlabelspositions<-seq(from=2, to=18, by=2)
text(x=xlabelspositions[1:5], y=par()$usr[3]-0.125,labels=xlabels[1:5], srt=90, adj=1, xpd=TRUE)
text(x=xlabelspositions[2], y=par()$usr[3]-0.125,labels=expression(paste(Delta," Temp. (Exp)")),
     srt=90, adj=1, xpd=TRUE)
text(x=xlabelspositions[3], y=par()$usr[3]-0.125,labels=expression(paste(Delta," Temp. (Lin)")),
     srt=90, adj=1, xpd=TRUE)

axis(at = round(c(-1, axTicks(2)),2), las = 1, side = 2)
mtext(side = 1, text = "Time",line = 3)
mtext(side = 2, text ="AICc", line = 3)

abline(h=min(res2$AICc), lty=2)
abline(h=min(res2$AICc)+4, lty=2)
```

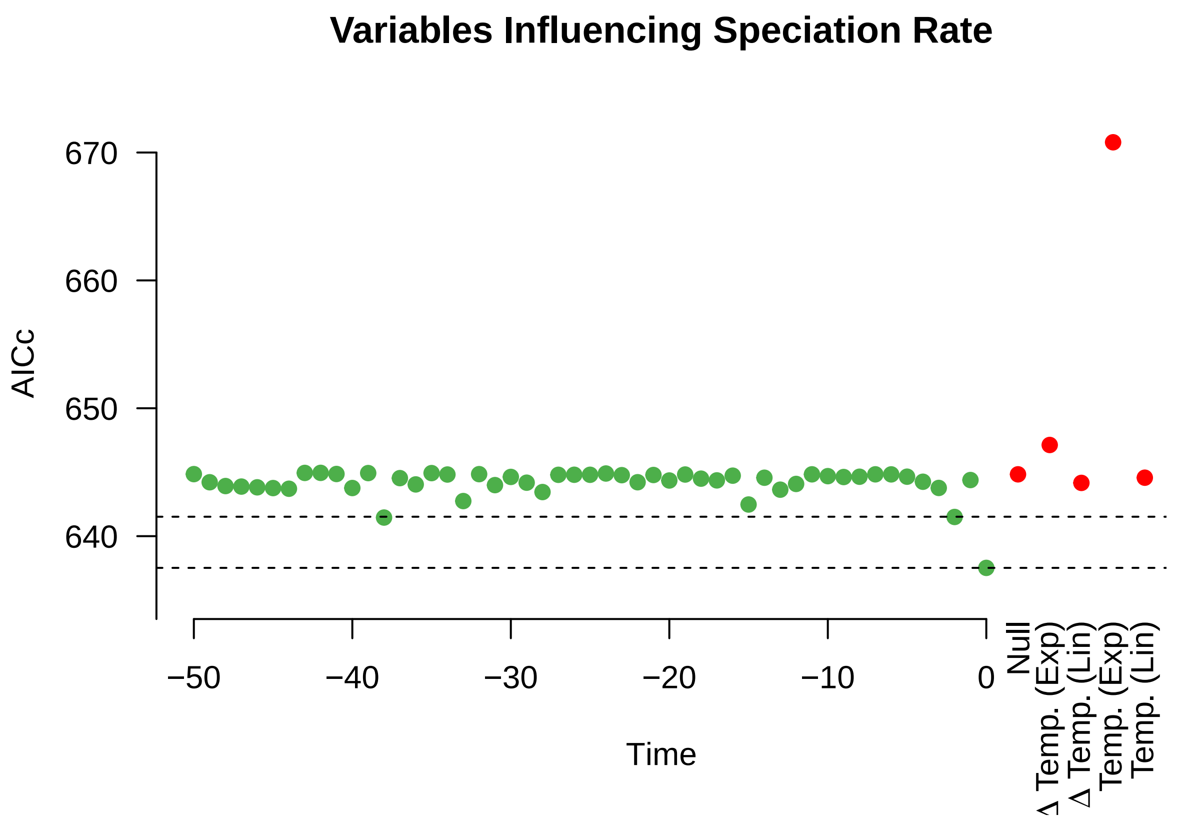


```r
dev.off()
```

```
## null device
##           1
```

We see that model uncertainty is large amongst all candidate models and that a null model with no change in speciation rate is favored. Note that the score of the model with the null peak influencing speciation rate has an AICc score of 637.52. This is within 4 delta AICc of the best-fitting extinction model which has a peak influencing extinction rate at 42 Ma and an AICc score of 640.65. Inference with this tree is wracked by model uncertainty and we cannot distinguish between a model positing a mass extinction at 42 and a model positing flat speciation and extinction rates.

3.2. TESS

For the tests for mass extinctions with TESS we first consider the convergence of the chains.

```r
tess.plot.multichain.diagnostics(l, parameters = "mass extinction times")
```

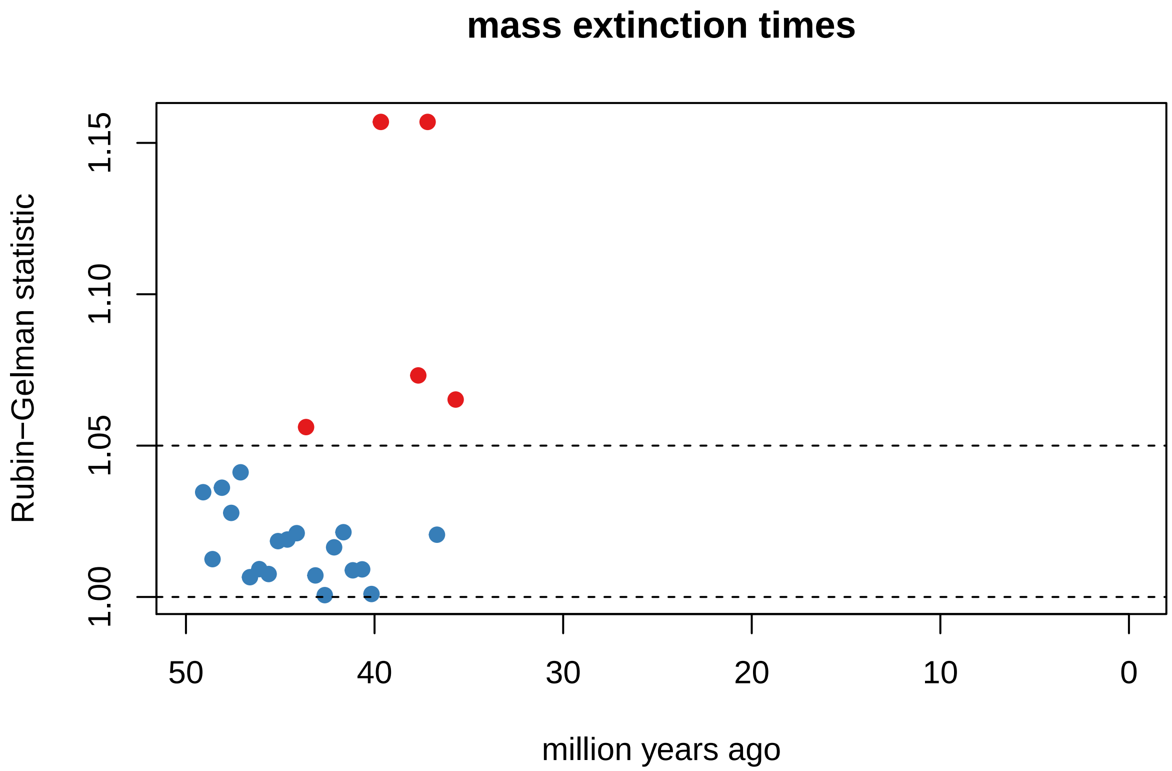

Two of those times seem to show borderline convergence errors with convergence above 1.1. But a few more chains could bring them down, as these are measures of between-chain to within-chain variation. We continue with single chain convergence diagnostics.

```
tess.plot.singlechain.diagnostics(l[[1]], parameters = "mass extinction times")
```

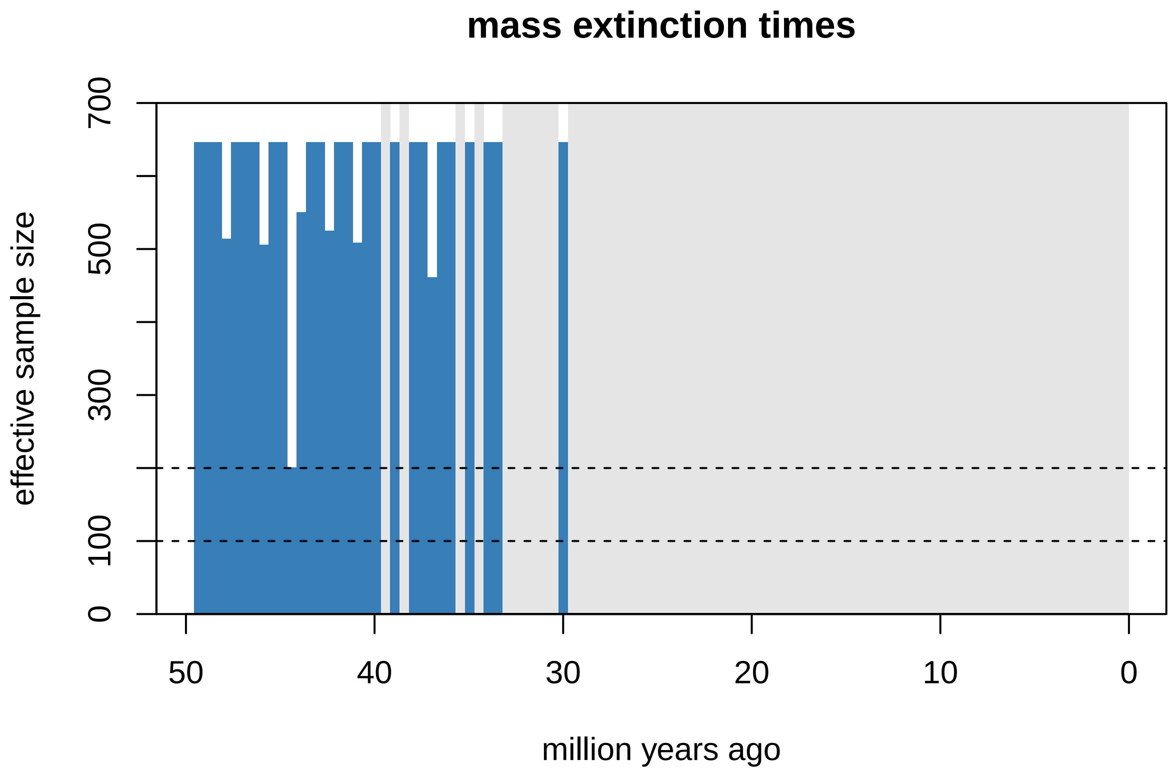
mass extinction times
effective sample size
0
100
300
500
700
50
40
30
20
10
0
million years ago

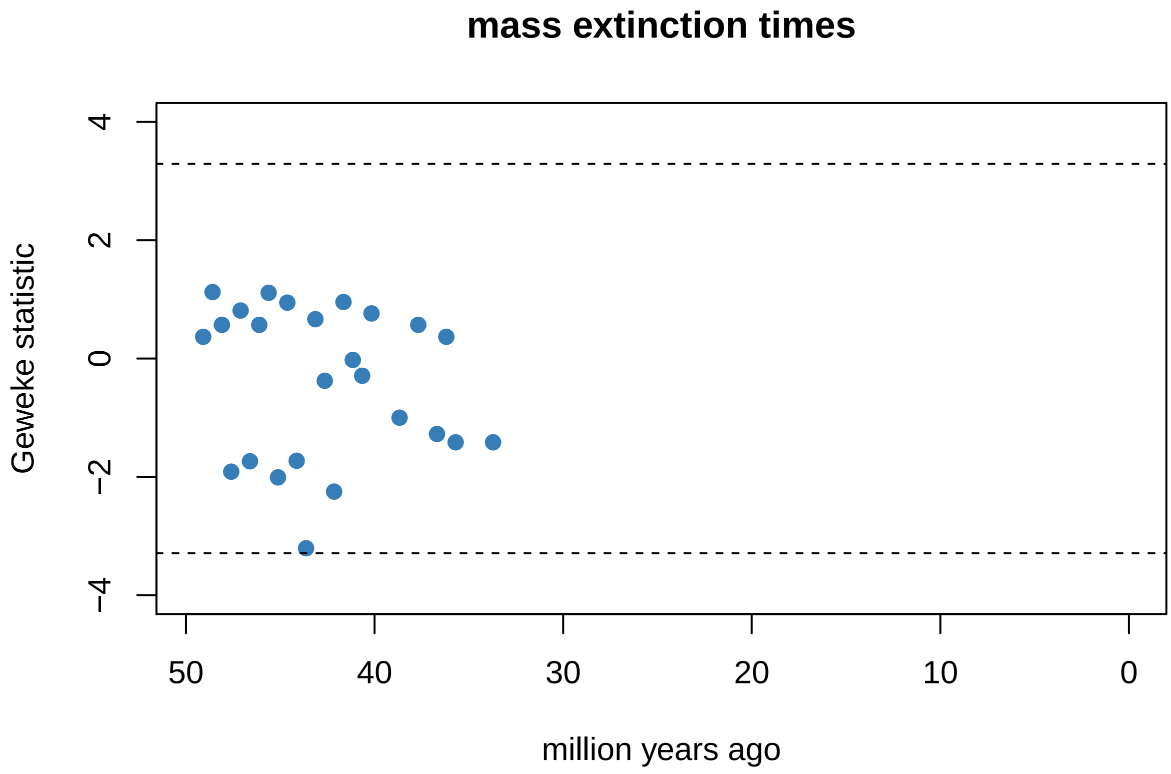


```
tess.plot.singlechain.diagnostics(l[[2]], parameters = "mass extinction times")
```

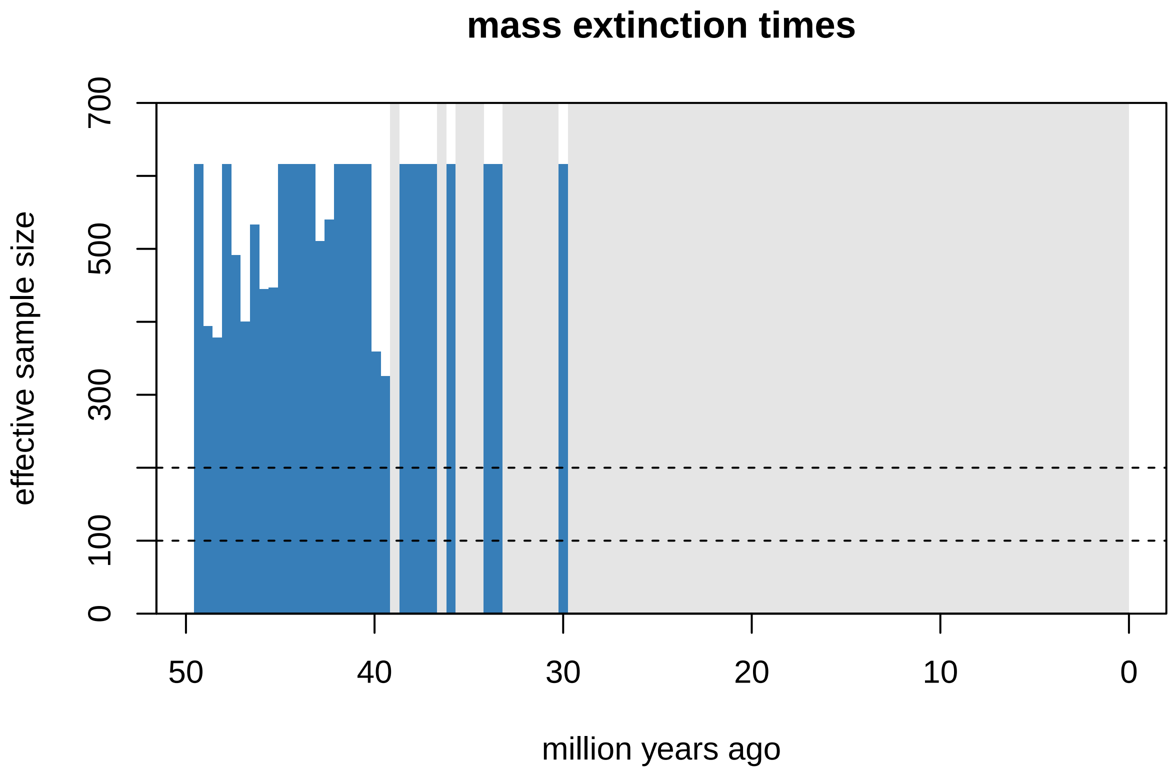
mass extinction times
effective sample size
0
100
300
500
700
50
40
30
20
10
0
million years ago

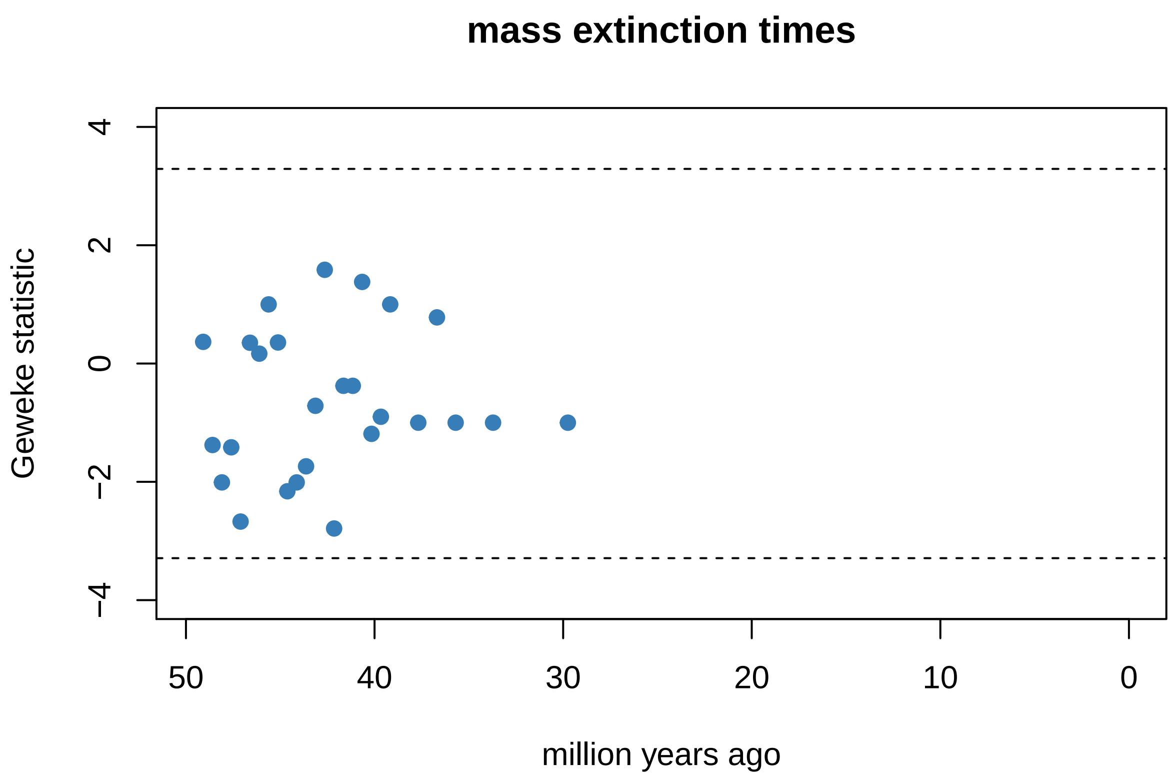


```
tess.plot.singlechain.diagnostics(l[[3]], parameters = "mass extinction times")
```

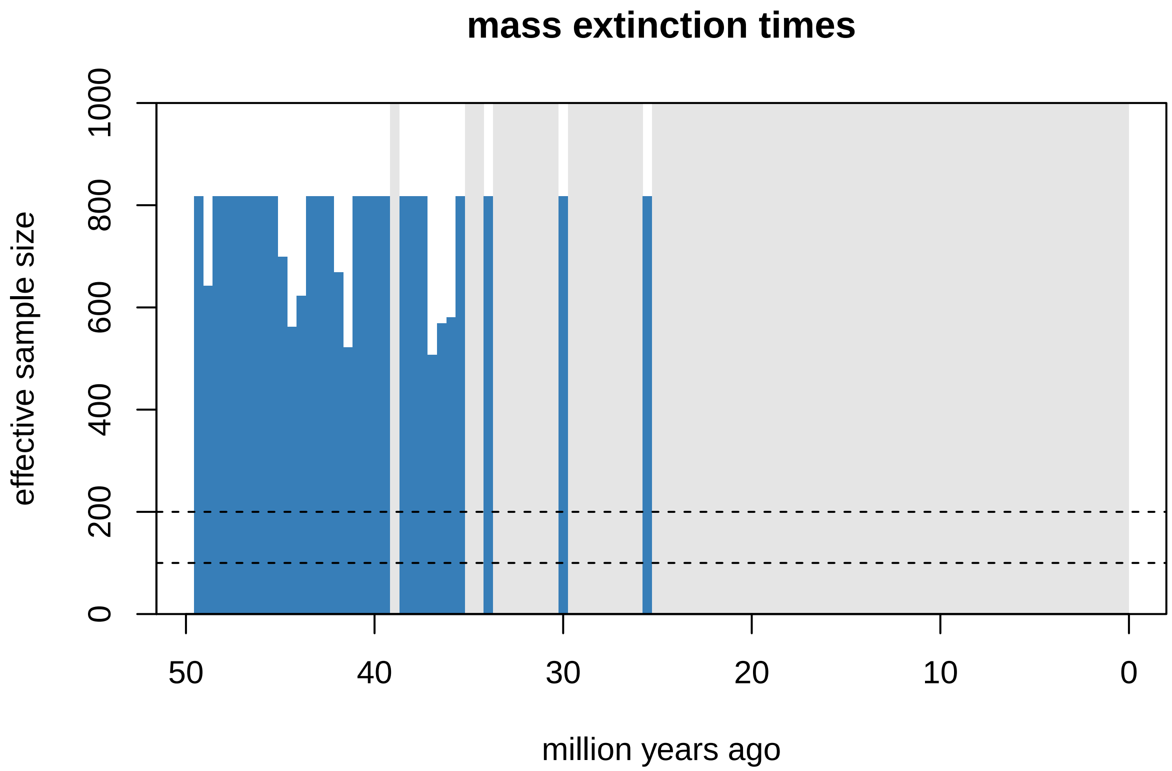
mass extinction times
effective sample size
0
200
400
600
800
1000
50
40
30
20
10
0
million years ago

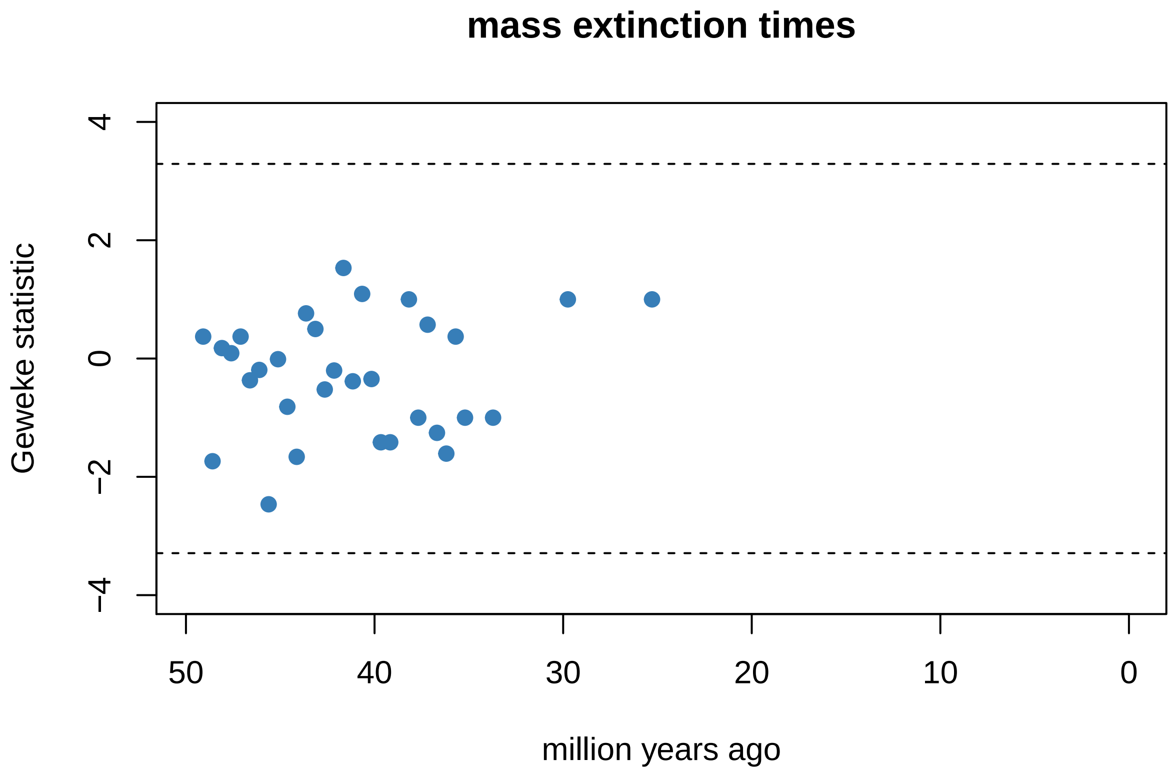


```
tess.plot.singlechain.diagnostics(l[[4]], parameters = "mass extinction times")
```

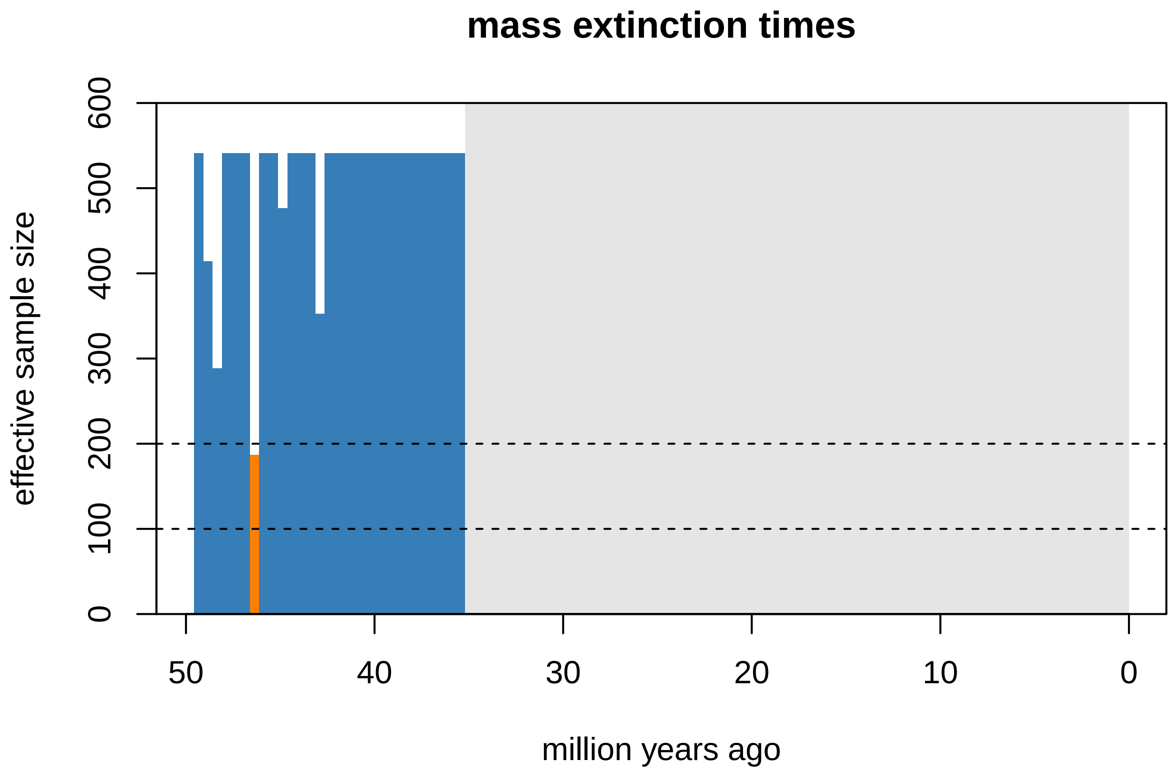
mass extinction times
effective sample size
0
100
200
300
400
500
600
50
40
30
20
10
0
million years ago

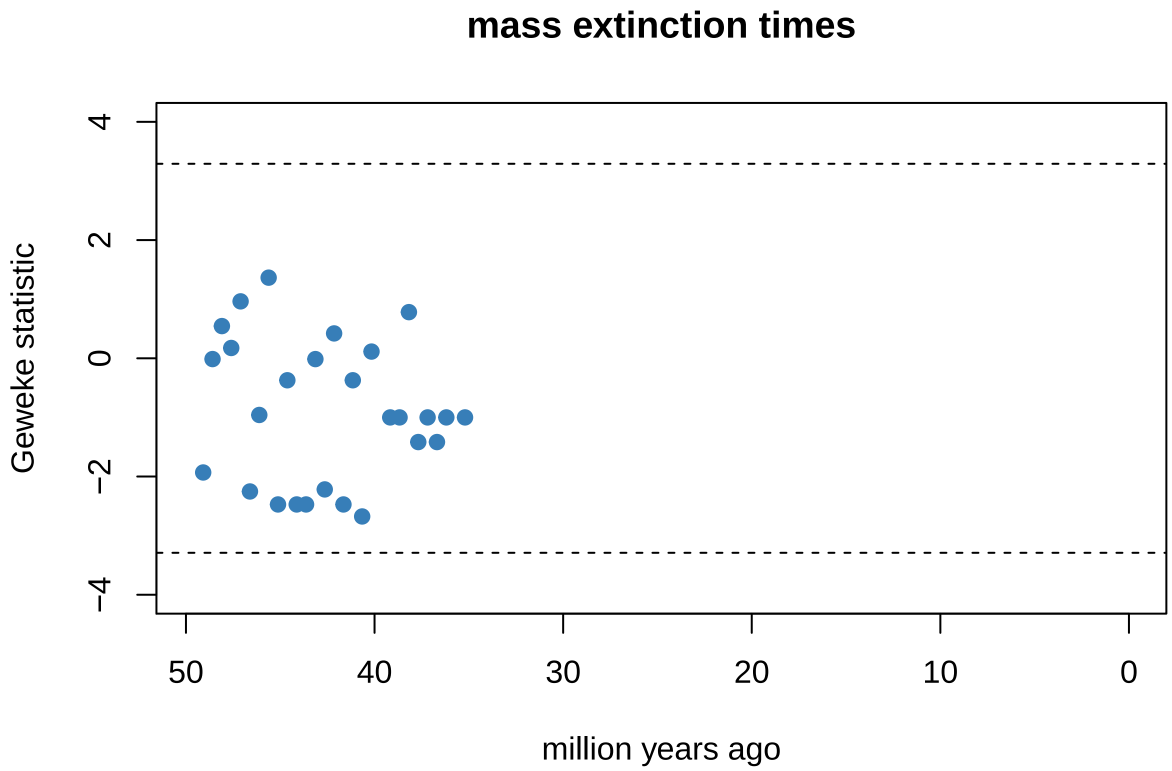

Effective sample size is sufficient and burn-in has been sufficient for all chains.

Do the inferences change when considering each chain individually?

```
tess.plot.output(l[[1]], "mass extinction times")
```

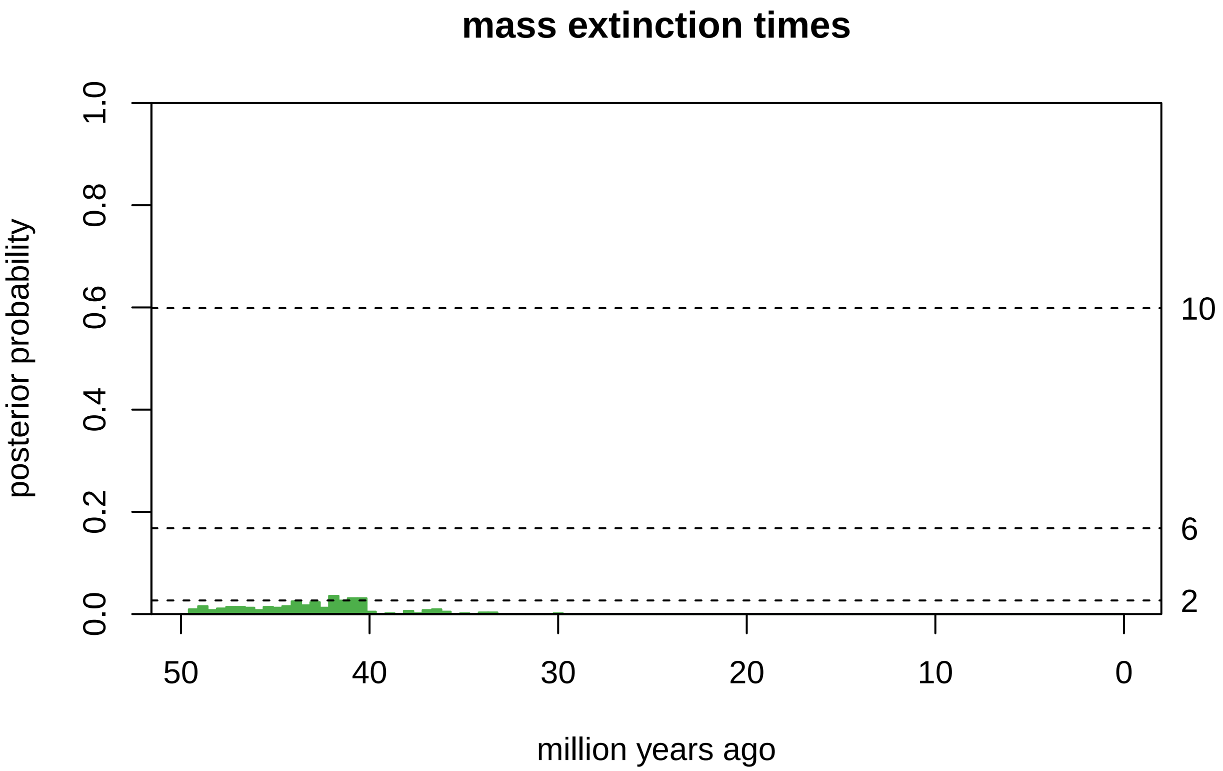


```
tess.plot.output(l[[2]], "mass extinction times")
```

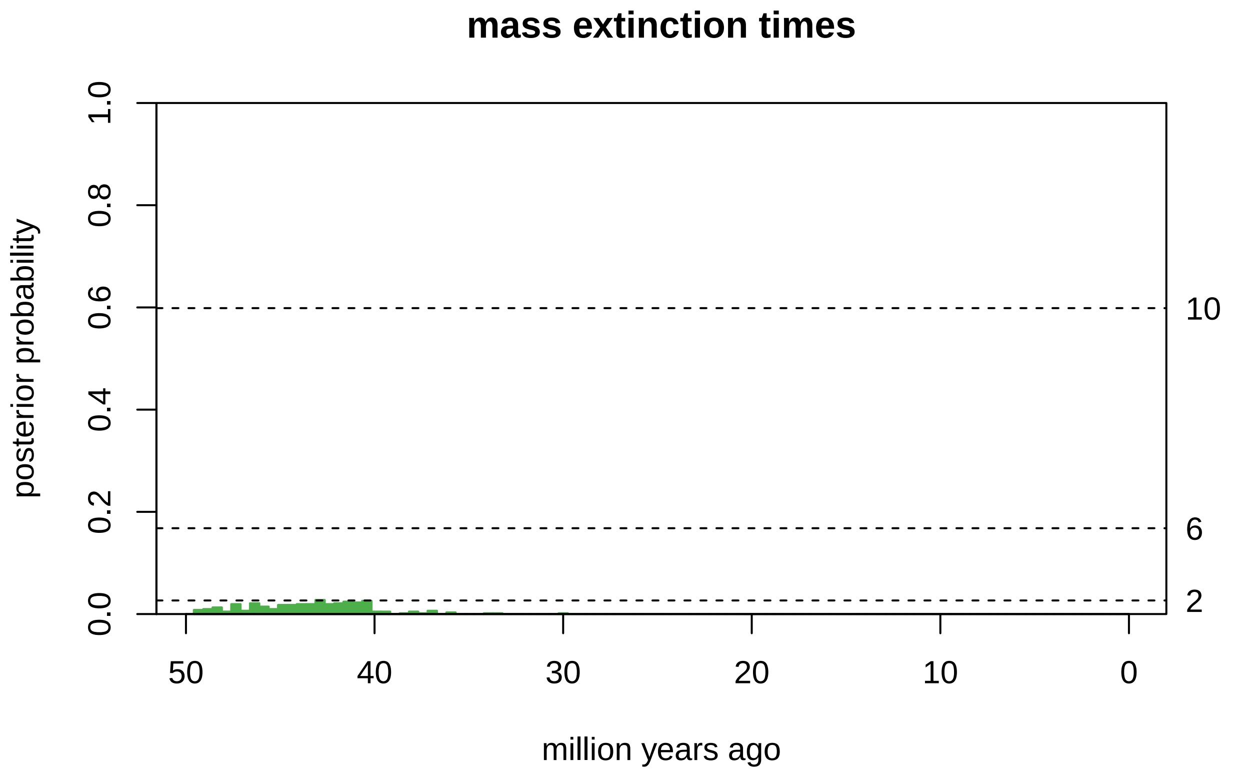


```
tess.plot.output(l[[3]], "mass extinction times")
```

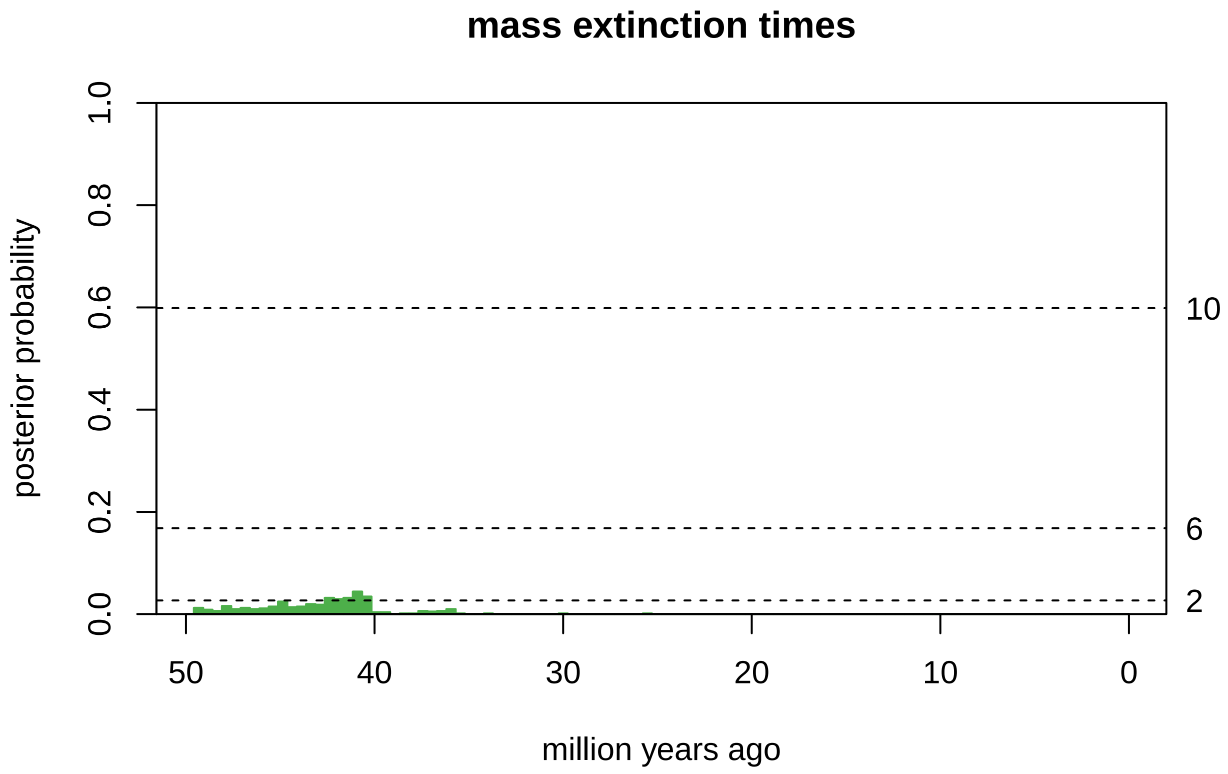


```
tess.plot.output(l[[4]], "mass extinction times")
```

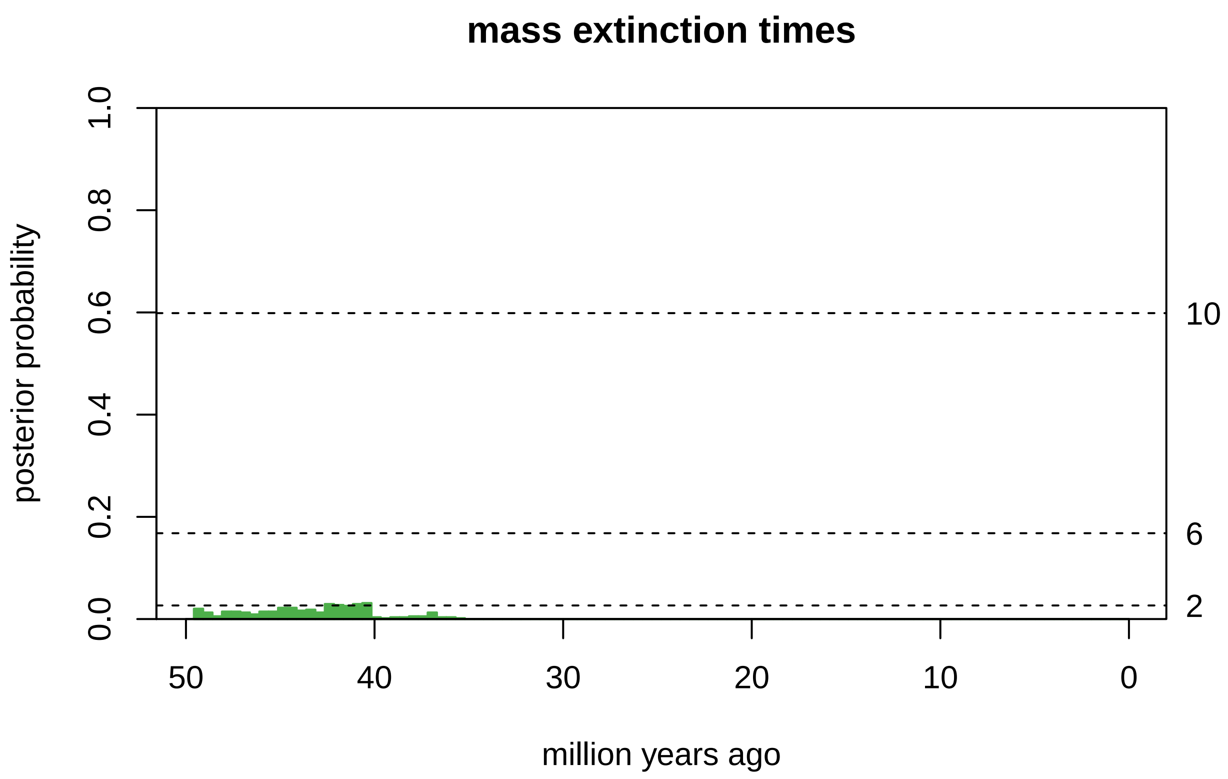

No. All models show a minor amount of evidence for an extinction happening just before 40Ma. But the amount of evidence is very weak and appears to just reach above the threshold of Bayes Factors >2. This is substantially weaker amount of evidence for the placement of an extinction event that the analysis with Kistler's modified tree.

```
thisOutput <- output3[["mass extinction Bayes factors"]]
ylim <- range(c(thisOutput, -10, 10), finite = TRUE)
col <- c(`speciation rates` = "#984EA3",
         `speciation shift times` = "#984EA3",
            `speciation Bayes factors` = "#984EA3",
         `extinction rates` = "#E41A1C",
            `extinction shift times` = "#E41A1C",
         `extinction Bayes factors` = "#E41A1C",
            `net-diversification rates` = "#377EB8",
         `relative-extinction rates` = "#FF7F00",
            `mass extinction times` = "#4DAF4A",
         `mass extinction Bayes factors` = "#4DAF4A")
treeAge <- max(branching.times(output$tree))
numIntervals <- length(output$intervals) - 1
plotAt <- 0:numIntervals
intervalSize <- treeAge/numIntervals
labels <- pretty(c(0, treeAge))
labelsAt <- numIntervals - (labels/intervalSize)
type="mass extinction Bayes factors"
thisOutput <- output3[[type]]
```

```r
#BF
plot.new()
plot.window(xlim = range((plotAt-100)/2), ylim = c(0,10))
points(x = c(plotAt[-1] - diff(plotAt[1:2])/2-100)/2, y = thisOutput,
       col = col["mass extinction Bayes factors"],pch=19)
abline(h = 2 * log(output$criticalBayesFactors[1:2]), lty = 2)
axis(at = c(round(1.2 * 50), axTicks(1)), side = 1)
axis(at = round(c(-1, axTicks(2)),2), las = 1, side = 2)
mtext(side = 1, text = "Time",line = 3)
mtext(side = 2, text = "Bayes Factors", line = 3)
text(-45,10,labels="b")
```

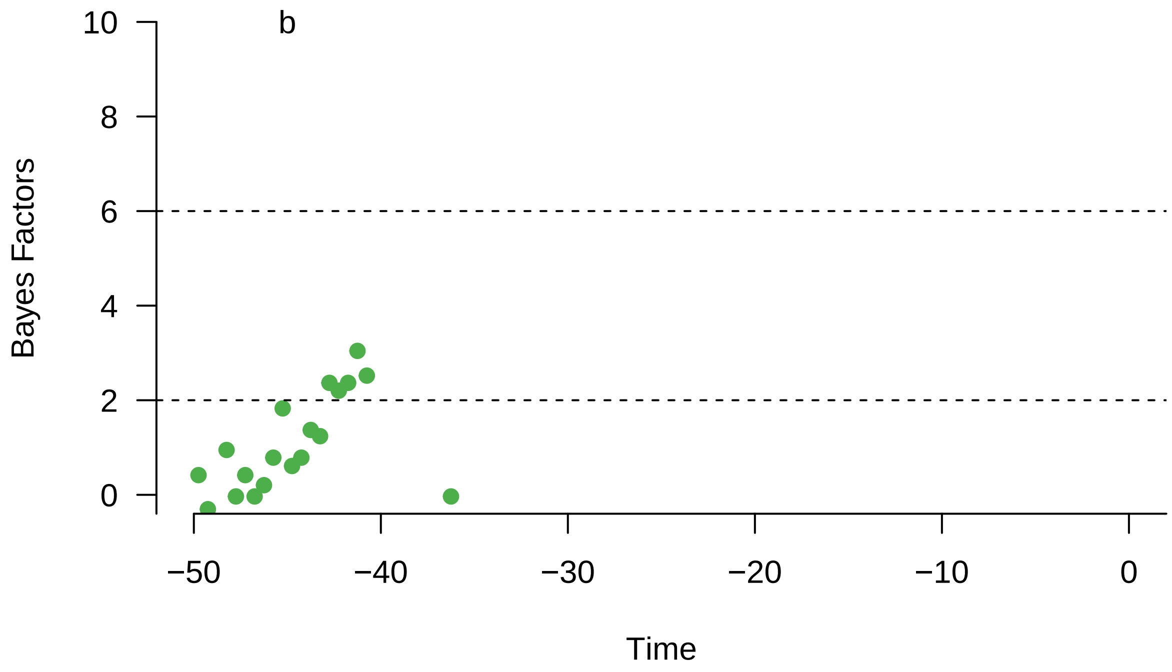


```r
#report raw values
cbind(c(plotAt[-1] - diff(plotAt[1:2])/2-100)/2,
      thisOutput
      )
```

```
##                thisOutput
##  [1,] -49.75  0.41637811
##  [2,] -49.25 -0.30438377
##  [3,] -48.75 -0.98225436
##  [4,] -48.25  0.94854620
##  [5,] -47.75 -0.03485337
##  [6,] -47.25  0.41637811
##  [7,] -46.75 -0.03485337
##  [8,] -46.25  0.20318336
##  [9,] -45.75  0.78597785
```

```
##  [10,] -45.25  1.82757939
##  [11,] -44.75  0.60947525
##  [12,] -44.25  0.78597785
##  [13,] -43.75  1.37129227
##  [14,] -43.25  1.23972301
##  [15,] -42.75  2.36740233
##  [16,] -42.25  2.20227278
##  [17,] -41.75  2.36740233
##  [18,] -41.25  3.04366043
##  [19,] -40.75  2.52067517
##  [20,] -40.25 -2.00881961
##  [21,] -39.75 -2.00881961
##  [22,] -39.25        -Inf
##  [23,] -38.75 -4.21094615
##  [24,] -38.25 -4.21094615
##  [25,] -37.75 -0.98225436
##  [26,] -37.25 -1.43099997
##  [27,] -36.75 -0.98225436
##  [28,] -36.25 -0.03485337
##  [29,] -35.75 -4.21094615
##  [30,] -35.25        -Inf
##  [31,] -34.75        -Inf
##  [32,] -34.25 -4.21094615
##  [33,] -33.75        -Inf
##  [34,] -33.25        -Inf
##  [35,] -32.75        -Inf
##  [36,] -32.25        -Inf
##  [37,] -31.75        -Inf
##  [38,] -31.25        -Inf
##  [39,] -30.75        -Inf
##  [40,] -30.25 -4.21094615
##  [41,] -29.75        -Inf
##  [42,] -29.25        -Inf
##  [43,] -28.75        -Inf
##  [44,] -28.25        -Inf
##  [45,] -27.75        -Inf
##  [46,] -27.25        -Inf
##  [47,] -26.75        -Inf
##  [48,] -26.25        -Inf
##  [49,] -25.75 -4.21094615
##  [50,] -25.25        -Inf
##  [51,] -24.75        -Inf
##  [52,] -24.25        -Inf
##  [53,] -23.75        -Inf
##  [54,] -23.25        -Inf
##  [55,] -22.75        -Inf
##  [56,] -22.25        -Inf
##  [57,] -21.75        -Inf
##  [58,] -21.25        -Inf
##  [59,] -20.75        -Inf
##  [60,] -20.25        -Inf
##  [61,] -19.75        -Inf
##  [62,] -19.25        -Inf
##  [63,] -18.75        -Inf
```

```
##  [64,] -18.25        -Inf
##  [65,] -17.75        -Inf
##  [66,] -17.25        -Inf
##  [67,] -16.75        -Inf
##  [68,] -16.25        -Inf
##  [69,] -15.75        -Inf
##  [70,] -15.25        -Inf
##  [71,] -14.75        -Inf
##  [72,] -14.25        -Inf
##  [73,] -13.75        -Inf
##  [74,] -13.25        -Inf
##  [75,] -12.75        -Inf
##  [76,] -12.25        -Inf
##  [77,] -11.75        -Inf
##  [78,] -11.25        -Inf
##  [79,] -10.75        -Inf
##  [80,] -10.25        -Inf
##  [81,]  -9.75        -Inf
##  [82,]  -9.25        -Inf
##  [83,]  -8.75        -Inf
##  [84,]  -8.25        -Inf
##  [85,]  -7.75        -Inf
##  [86,]  -7.25        -Inf
##  [87,]  -6.75        -Inf
##  [88,]  -6.25        -Inf
##  [89,]  -5.75        -Inf
##  [90,]  -5.25        -Inf
##  [91,]  -4.75        -Inf
##  [92,]  -4.25        -Inf
##  [93,]  -3.75        -Inf
##  [94,]  -3.25        -Inf
##  [95,]  -2.75        -Inf
##  [96,]  -2.25        -Inf
##  [97,]  -1.75        -Inf
##  [98,]  -1.25        -Inf
##  [99,]  -0.75        -Inf
## [100,]  -0.25        -Inf
```

Now we look at where the sensitivity analysis places the extinctions.

Weak Prior 1

```
output_2 <- tess.process.output("sub3/h1/tess/tess_analysis_thinpriors_mu_lambda_weakextinction1",
numExpectedRateChanges = numExpectedRateChanges,
numExpectedMassExtinctions = numExpectedMassExtinctions)
tess.plot.singlechain.diagnostics(output_2,parameters = c("mass extinction times"),las=2)
```

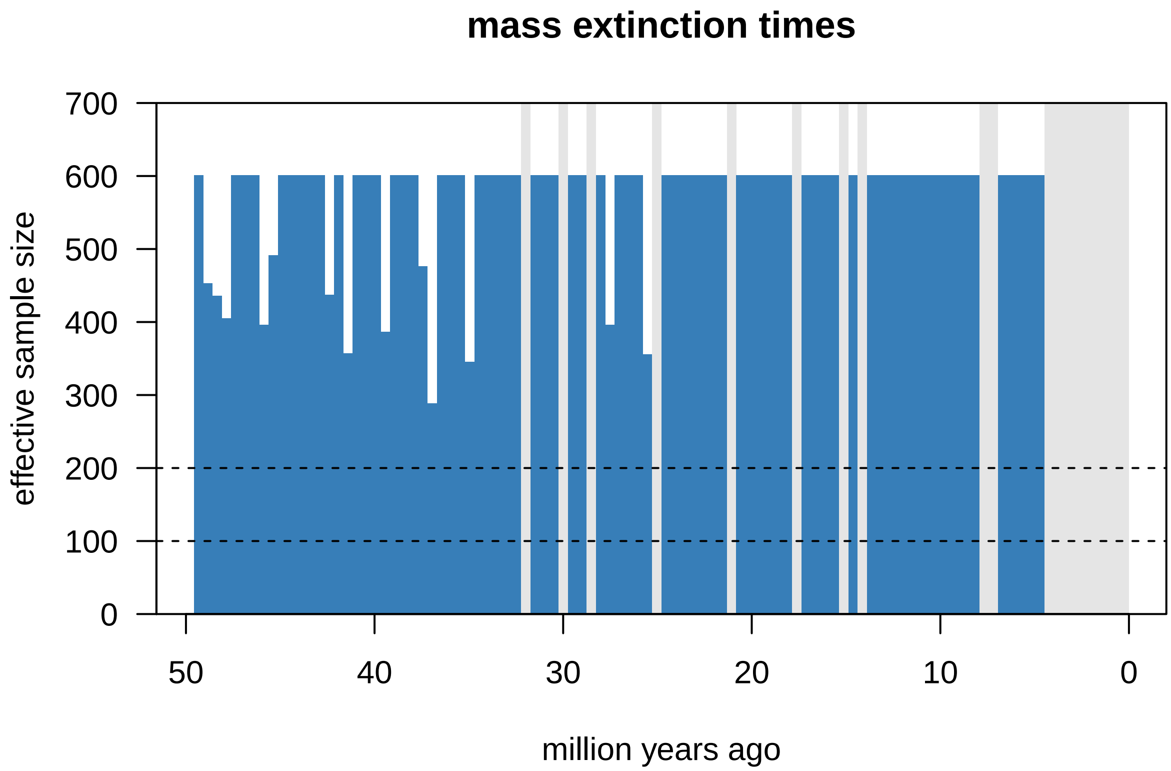
mass extinction times
effective sample size
700
600
500
400
300
200
100
0
50
40
30
20
10
0
million years ago

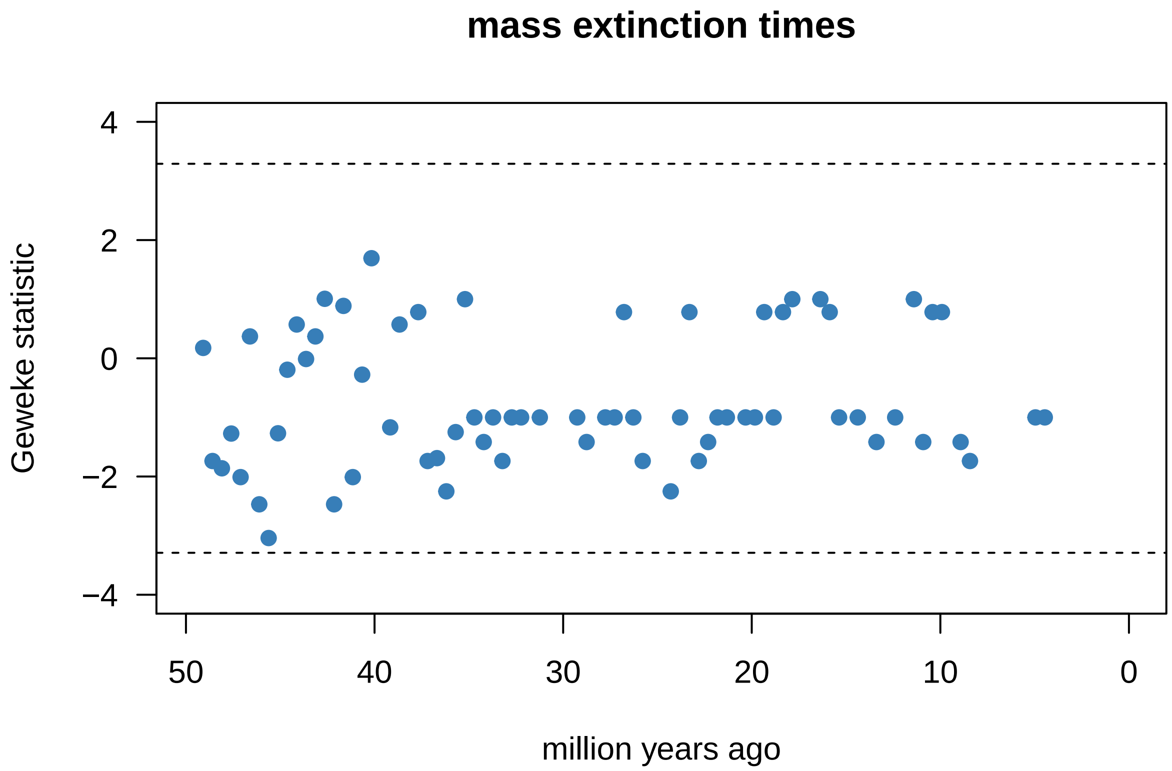


```
tess.plot.output(output_2,fig.types =  c("mass extinction times"),las=2)
```

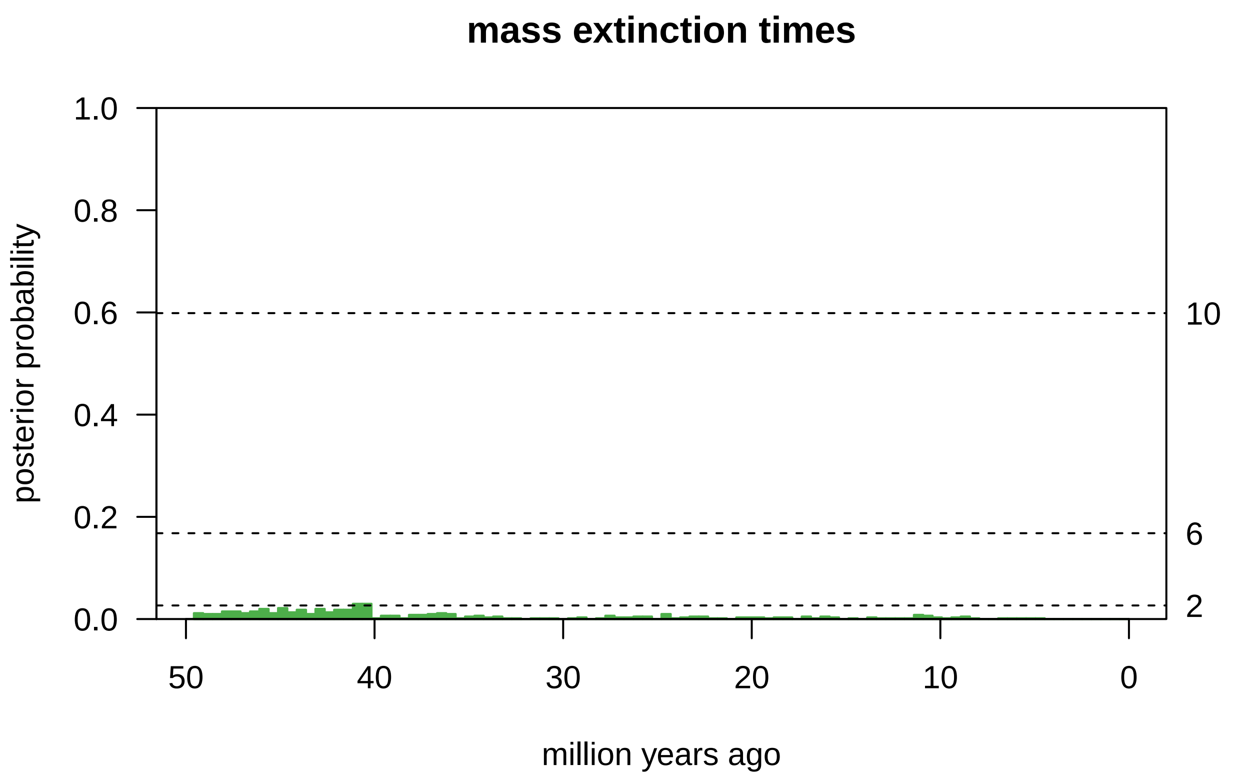


```
op2<-cbind(output_2$`mass extinction Bayes factors`,
      output_2$intervals)
op2
```

```
##               [,1]       [,2]
##  [1,]  0.318393698 49.5814642
##  [2,]  0.006728166 49.0856495
##  [3,]  0.006728166 48.5898349
##  [4,]  0.827767924 48.0940203
##  [5,]  0.827767924 47.5982056
##  [6,]  0.318393698 47.1023910
##  [7,]  0.827767924 46.6065763
##  [8,]  1.413292971 46.1107617
##  [9,]  0.318393698 45.6149471
## [10,]  1.576776858 45.1191324
## [11,]  0.588826324 44.6233178
## [12,]  1.235877510 44.1275031
## [13,]  0.006728166 43.6316885
## [14,]  1.413292971 43.1358738
## [15,]  0.588826324 42.6400592
## [16,]  1.235877510 42.1442446
## [17,]  1.235877510 41.6484299
## [18,]  2.244701182 41.1526153
## [19,]  2.244701182 40.6568006
## [20,] -3.593527272 40.1609860
## [21,] -0.810913466 39.6651714
```

```
##  [22,] -0.810913466 39.1693567
##  [23,] -3.593527272 38.6735421
##  [24,] -0.361273471 38.1777274
##  [25,] -0.361273471 37.6819128
##  [26,]  0.006728166 37.1860981
##  [27,]  0.318393698 36.6902835
##  [28,]  0.006728166 36.1944689
##  [29,] -3.593527272 35.6986542
##  [30,] -1.389624892 35.2028396
##  [31,] -0.810913466 34.7070249
##  [32,] -2.203896796 34.2112103
##  [33,] -1.389624892 33.7153956
##  [34,] -3.593527272 33.2195810
##  [35,] -3.593527272 32.7237664
##  [36,]         -Inf 32.2279517
##  [37,] -3.593527272 31.7321371
##  [38,] -3.593527272 31.2363224
##  [39,] -3.593527272 30.7405078
##  [40,]         -Inf 30.2446932
##  [41,] -3.593527272 29.7488785
##  [42,] -2.203896796 29.2530639
##  [43,]         -Inf 28.7572492
##  [44,] -3.593527272 28.2614346
##  [45,] -0.810913466 27.7656199
##  [46,] -2.203896796 27.2698053
##  [47,] -2.203896796 26.7739907
##  [48,] -1.389624892 26.2781760
##  [49,] -1.389624892 25.7823614
##  [50,]         -Inf 25.2865467
##  [51,]  0.006728166 24.7907321
##  [52,] -3.593527272 24.2949175
##  [53,] -2.203896796 23.7991028
##  [54,] -1.389624892 23.3032882
##  [55,] -1.389624892 22.8074735
##  [56,] -3.593527272 22.3116589
##  [57,] -3.593527272 21.8158442
##  [58,]         -Inf 21.3200296
##  [59,] -2.203896796 20.8242150
##  [60,] -2.203896796 20.3284003
##  [61,] -2.203896796 19.8325857
##  [62,] -3.593527272 19.3367710
##  [63,] -2.203896796 18.8409564
##  [64,] -2.203896796 18.3451418
##  [65,]         -Inf 17.8493271
##  [66,] -1.389624892 17.3535125
##  [67,] -3.593527272 16.8576978
##  [68,] -1.389624892 16.3618832
##  [69,] -2.203896796 15.8660685
##  [70,]         -Inf 15.3702539
##  [71,] -3.593527272 14.8744393
##  [72,]         -Inf 14.3786246
##  [73,] -2.203896796 13.8828100
##  [74,] -3.593527272 13.3869953
##  [75,] -3.593527272 12.8911807
```

```
##  [76,] -3.593527272 12.3953660
##  [77,] -3.593527272 11.8995514
##  [78,] -0.361273471 11.4037368
##  [79,] -0.810913466 10.9079221
##  [80,] -2.203896796 10.4121075
##  [81,] -3.593527272  9.9162928
##  [82,] -2.203896796  9.4204782
##  [83,] -1.389624892  8.9246636
##  [84,] -3.593527272  8.4288489
##  [85,]         -Inf  7.9330343
##  [86,]         -Inf  7.4372196
##  [87,] -3.593527272  6.9414050
##  [88,] -3.593527272  6.4455903
##  [89,] -3.593527272  5.9497757
##  [90,] -3.593527272  5.4539611
##  [91,] -3.593527272  4.9581464
##  [92,]         -Inf  4.4623318
##  [93,]         -Inf  3.9665171
##  [94,]         -Inf  3.4707025
##  [95,]         -Inf  2.9748879
##  [96,]         -Inf  2.4790732
##  [97,]         -Inf  1.9832586
##  [98,]         -Inf  1.4874439
##  [99,]         -Inf  0.9916293
## [100,]         -Inf  0.4958146
## [101,]  0.318393698  0.0000000
```

Weak Prior 2

```
output_3 <- tess.process.output("sub3/h1/tess/tess_analysis_thinpriors_mu_lambda_weakprior2",
numExpectedRateChanges = numExpectedRateChanges,
numExpectedMassExtinctions = numExpectedMassExtinctions)
tess.plot.singlechain.diagnostics(output_3,parameters = c("mass extinction times"),las=2)
```

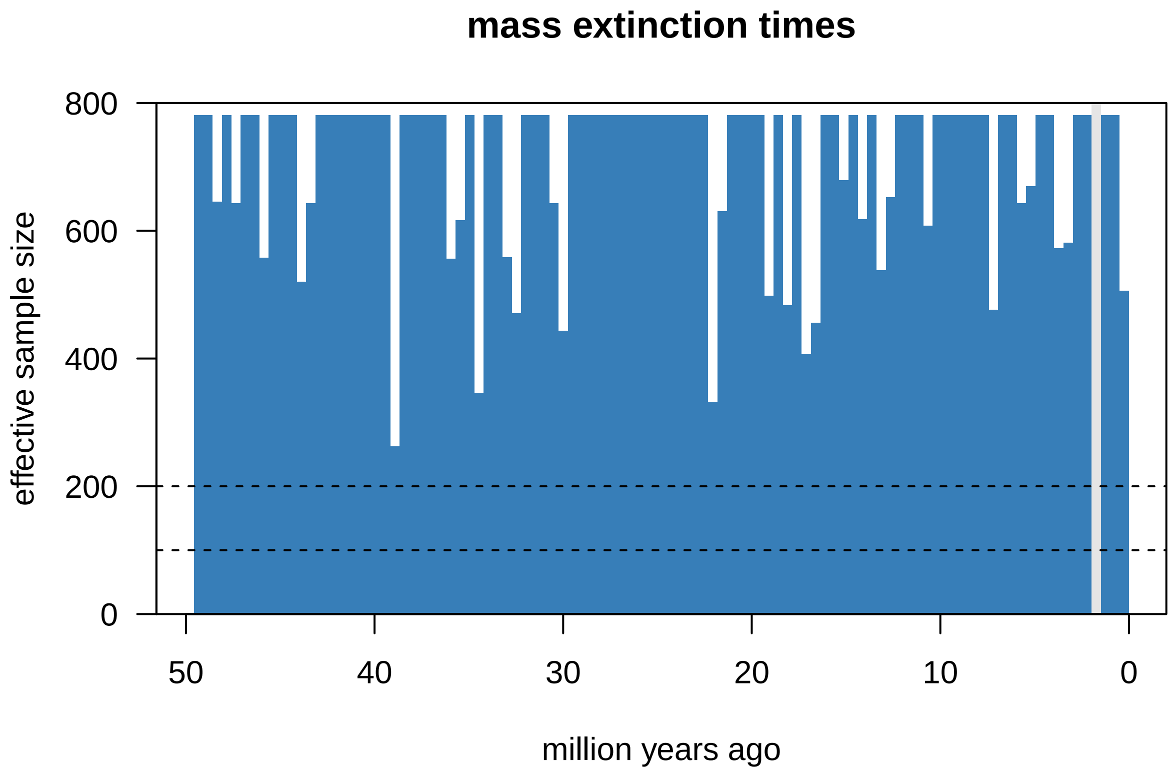
mass extinction times
effective sample size
800
600
400
200
0
50
40
30
20
10
0
million years ago

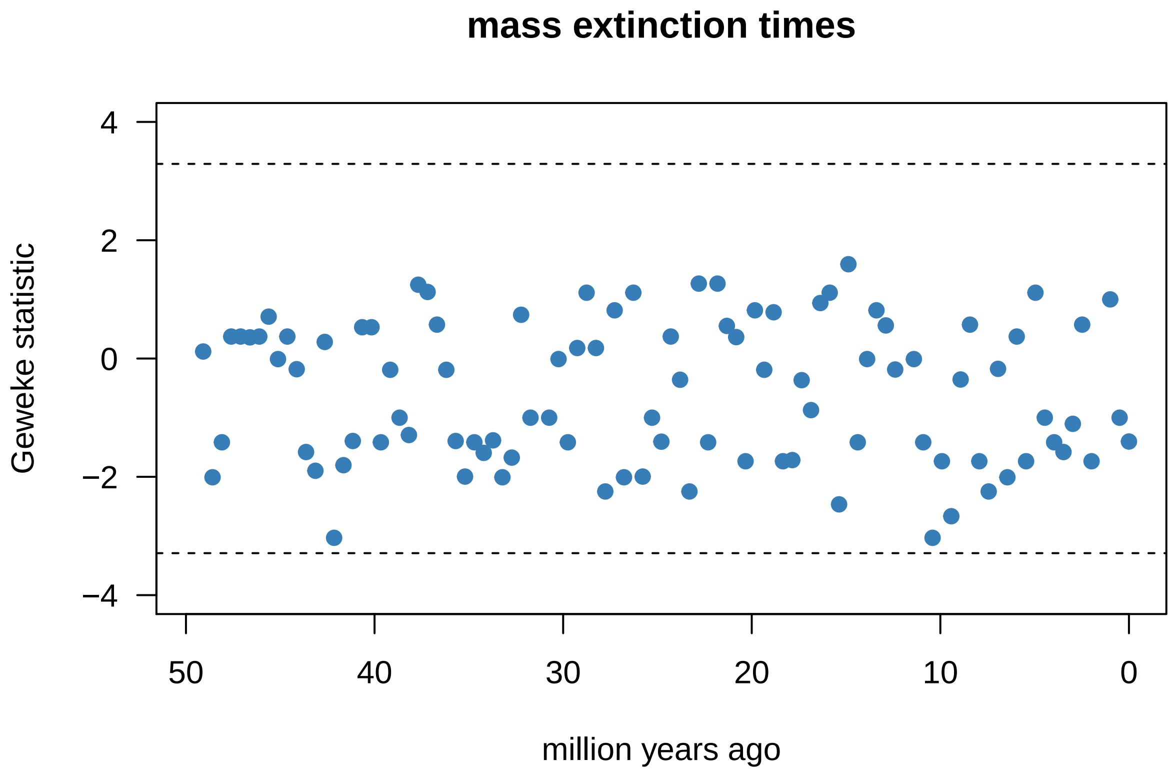


```
tess.plot.output(output_3,fig.types =  c("mass extinction times"),las=2)
```

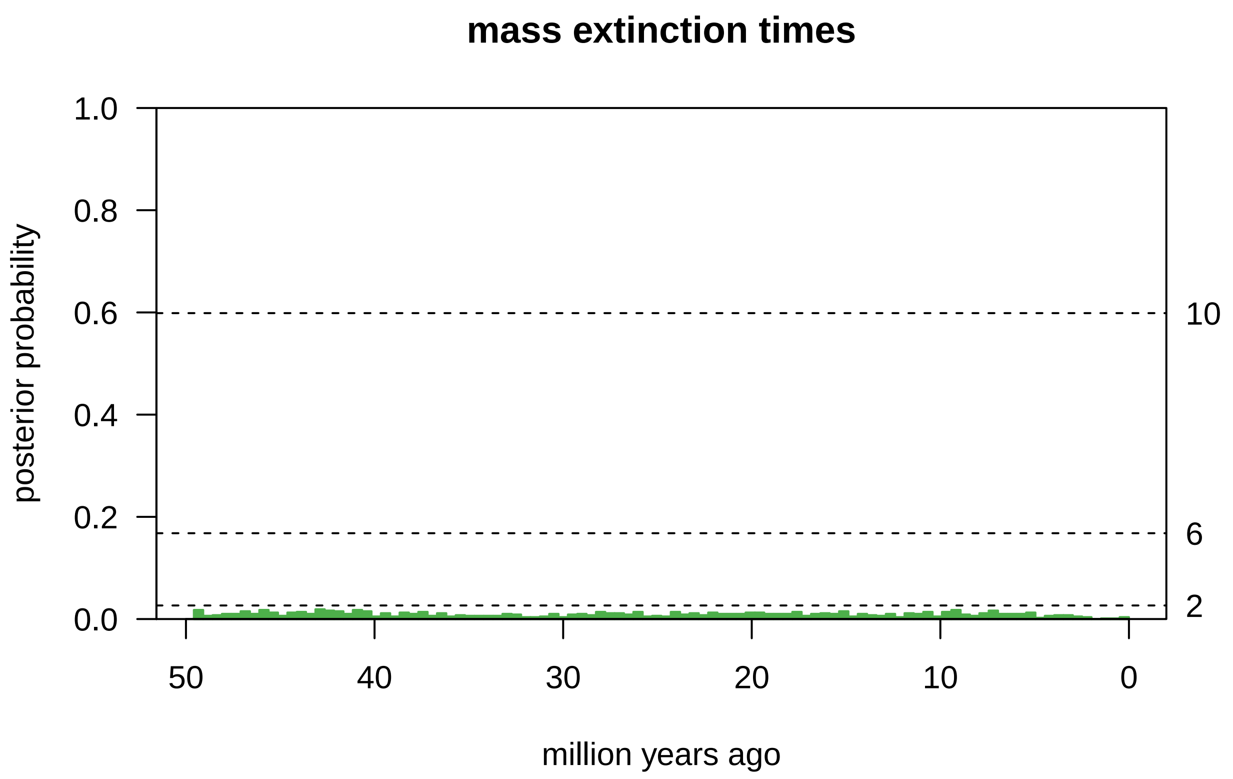


```r
op3<-cbind(output_3$`mass extinction Bayes factors`,
      output_3$intervals)
op3
```

```
##              [,1]       [,2]
##   [1,]  1.19347310 49.5814642
##   [2,] -0.88909718 49.0856495
##   [3,] -0.52187508 48.5898349
##   [4,]  0.05865702 48.0940203
##   [5,]  0.05865702 47.5982056
##   [6,]  0.87996340 47.1023910
##   [7,]  0.05865702 46.6065763
##   [8,]  1.19347310 46.1107617
##   [9,]  0.51012548 45.6149471
##  [10,] -0.88909718 45.1191324
##  [11,]  0.51012548 44.6233178
##  [12,]  0.70334155 44.1275031
##  [13,]  0.05865702 43.6316885
##  [14,]  1.33406810 43.1358738
##  [15,]  1.04265129 42.6400592
##  [16,]  0.87996340 42.1442446
##  [17,]  0.05865702 41.6484299
##  [18,]  1.19347310 41.1526153
##  [19,]  0.87996340 40.6568006
##  [20,] -1.33795994 40.1609860
##  [21,]  0.29681209 39.6651714
```

```
##  [22,] -1.33795994 39.1693567
##  [23,]  0.51012548 38.6735421
##  [24,]  0.05865702 38.1777274
##  [25,]  0.70334155 37.6819128
##  [26,] -0.88909718 37.1860981
##  [27,]  0.29681209 36.6902835
##  [28,] -1.33795994 36.1944689
##  [29,] -0.52187508 35.6986542
##  [30,] -0.88909718 35.2028396
##  [31,] -0.88909718 34.7070249
##  [32,] -0.88909718 34.2112103
##  [33,] -0.88909718 33.7153956
##  [34,]  0.05865702 33.2195810
##  [35,] -0.21099141 32.7237664
##  [36,] -1.91589643 32.2279517
##  [37,] -1.91589643 31.7321371
##  [38,] -1.33795994 31.2363224
##  [39,]  0.05865702 30.7405078
##  [40,] -1.91589643 30.2446932
##  [41,] -0.21099141 29.7488785
##  [42,]  0.05865702 29.2530639
##  [43,] -0.52187508 28.7572492
##  [44,]  0.70334155 28.2614346
##  [45,]  0.29681209 27.7656199
##  [46,]  0.29681209 27.2698053
##  [47,] -0.21099141 26.7739907
##  [48,]  0.70334155 26.2781760
##  [49,] -1.33795994 25.7823614
##  [50,] -0.88909718 25.2865467
##  [51,] -1.33795994 24.7907321
##  [52,]  0.70334155 24.2949175
##  [53,] -0.21099141 23.7991028
##  [54,]  0.29681209 23.3032882
##  [55,] -0.52187508 22.8074735
##  [56,]  0.51012548 22.3116589
##  [57,]  0.05865702 21.8158442
##  [58,]  0.05865702 21.3200296
##  [59,]  0.05865702 20.8242150
##  [60,]  0.51012548 20.3284003
##  [61,]  0.51012548 19.8325857
##  [62,]  0.05865702 19.3367710
##  [63,]  0.05865702 18.8409564
##  [64,]  0.05865702 18.3451418
##  [65,]  0.70334155 17.8493271
##  [66,] -0.88909718 17.3535125
##  [67,]  0.05865702 16.8576978
##  [68,]  0.29681209 16.3618832
##  [69,]  0.05865702 15.8660685
##  [70,]  0.87996340 15.3702539
##  [71,] -1.33795994 14.8744393
##  [72,]  0.05865702 14.3786246
##  [73,] -0.52187508 13.8828100
##  [74,] -0.88909718 13.3869953
##  [75,]  0.05865702 12.8911807
```

```
##  [76,] -1.91589643 12.3953660
##  [77,]  0.29681209 11.8995514
##  [78,]  0.05865702 11.4037368
##  [79,]  0.70334155 10.9079221
##  [80,] -1.33795994 10.4121075
##  [81,]  0.70334155  9.9162928
##  [82,]  1.19347310  9.4204782
##  [83,] -0.21099141  8.9246636
##  [84,] -0.88909718  8.4288489
##  [85,]  0.29681209  7.9330343
##  [86,]  1.04265129  7.4372196
##  [87,]  0.05865702  6.9414050
##  [88,]  0.05865702  6.4455903
##  [89,]  0.05865702  5.9497757
##  [90,]  0.51012548  5.4539611
##  [91,] -2.72939569  4.9581464
##  [92,] -0.88909718  4.4623318
##  [93,] -0.52187508  3.9665171
##  [94,] -0.52187508  3.4707025
##  [95,] -1.33795994  2.9748879
##  [96,] -1.91589643  2.4790732
##  [97,]        -Inf  1.9832586
##  [98,] -4.11825580  1.4874439
##  [99,] -4.11825580  0.9916293
## [100,] -1.91589643  0.4958146
## [101,]  1.19347310  0.0000000
```

Relaxing the magnitude of extinction decreases the magnitude of evidence for any extinction.

Now plot all tests together.

```r
par(mar=c(5.5,4.1,1,2))
layout(matrix(c(1,1,2,3,4,5), 3, 2, byrow = TRUE))


plot.new()


plot.window(xlim = c(-50,18), ylim = c(638,646))


#polygon(c(-31.5, -31.5,-40.5,-40.5), c(533,543,543,533), col = "#DDDDDD", border = "#DDDDDD")
#polygon(c(-43.5, -43.5,-45.5,-45.5), c(533,543,543,533), col = "#DDDDDD", border = "#DDDDDD")

points(AICc~year_of_ex, data=res[1:c(nrow(res)-1),], pch=19, col="black")
aicc.table3$year_of_ex<-c(10, 8, 6, 4, 2)
points(AICc~year_of_ex, data=aicc.table3, pch=19, col="gray80")
aicc.table$year_of_ex<-c(12, 14, 16, 18)
points(AICc~year_of_ex, data=aicc.table, pch=1, col="black")

axis(at = c(-50, -40, -30, -20, -10, 0), side = 1)
xlabelspositions<-seq(from=2, to=18, by=2)
text(x=xlabelspositions, y=par()$usr[3]-0.125,labels=xlabels, srt=90, adj=1, xpd=TRUE)
text(x=xlabelspositions[2], y=par()$usr[3]-0.125,labels=expression(paste(Delta," Temp. (Exp)")),
     srt=90, adj=1, xpd=TRUE)
```

```
text(x=xlabelspositions[3], y=par()$usr[3]-0.125,labels=expression(paste(Delta," Temp. (Lin)")),
     srt=90, adj=1, xpd=TRUE)

####r right here tight now
axis(at = round(c(-1, axTicks(2)),2), las = 1, side = 2)
mtext(side = 1, text = "Time",line = 3)
mtext(side = 2, text ="AICc", line = 3)
text(-47.5,542,labels="a")

abline(h=min(res$AICc), lty=2)
abline(h=min(res$AICc)+4, lty=2)


##############
# B
 #Akaike Weights
plot.new()
plot.window(xlim = c(-50,0), ylim = c(0,0.2))
points(AkaikeWeights~year_of_ex, data=res[1:c(nrow(res)-1),], pch=19, col="black",
     ylim=c(0,0.2))
axis(at = c(round(1.2 * 50), axTicks(1)), side = 1)
axis(at = round(c(-1, axTicks(2)),2), las = 1, side = 2)
mtext(side = 1, text = "Time",line = 3)
mtext(side = 2, text ="Akaike Weights", line = 3)
text(-45,0.2,labels="b")


######################
#C
plot.new()
plot.window(xlim = range((plotAt-100)/2), ylim = c(0,10))
#polygon(c(-31, -31,-36.5,-36.5), c(-1,9,9,-1), col = "#DDDDDD", border = "#DDDDDD")

points(x = c(plotAt[-1] - diff(plotAt[1:2])/2-100)/2, y = thisOutput,
       col = "black",pch=19)
#points(x = c(plotAt[-1] - diff(plotAt[1:2])/2-100)/2, y = output_2$`mass extinction Bayes factors`,
#       col = "gray80",pch=19)
#points(x = c(plotAt[-1] - diff(plotAt[1:2])/2-100)/2, y = output_3$`mass extinction Bayes factors`,
#       col = "black",pch=1)

#legend(x=c(-30,-20), y=c(7,10), col=c("black", "grey80", "black"), pch=c(19, 19, 1),
#       legend = c("95% (91-99%)", "75% (50-93%)", "25% (6-52%)"),
#       cex=1,yjust=-5,
#       x.intersp = 0.25, y.intersp = 0.2, inset = -1, box.lty = 0)

abline(h = 2 * log(output$criticalBayesFactors[1:2]), lty = 2)
axis(at = c(round(1.2 * 50), axTicks(1)), side = 1)
axis(at = round(c(-1, axTicks(2)),2), las = 1, side = 2)
mtext(side = 1, text = "Time",line = 3)
mtext(side = 2, text = "Bayes Factors", line = 3)
text(-45,10,labels="c")
text(-25, 10, "95% (91-99%) Extinction", font=1, cex=1)
```

```
#########
# D
plot.new()
plot.window(xlim = range((plotAt-100)/2), ylim = c(0,10))
#points(x = c(plotAt[-1] - diff(plotAt[1:2])/2-100)/2, y = thisOutput,
#       col = "black",pch=19)
# polygon(c(-9.5, -9.5,-8,-8), c(-1,9,9,-1), col = "#DDDDDD", border = "#DDDDDD")
# polygon(c(-31, -31,-33.5,-33.5), c(-1,9,9,-1), col = "#DDDDDD", border = "#DDDDDD")
# polygon(c(-34, -34,-34.5,-34.5), c(-1,9,9,-1), col = "#DDDDDD", border = "#DDDDDD")
# polygon(c(-35, -35,-35.5,-35.5), c(-1,9,9,-1), col = "#DDDDDD", border = "#DDDDDD")
# polygon(c(-36, -36,-36.5,-36.5), c(-1,9,9,-1), col = "#DDDDDD", border = "#DDDDDD")
# polygon(c(-37, -37,-37.5,-37.5), c(-1,9,9,-1), col = "#DDDDDD", border = "#DDDDDD")

points(x = c(plotAt[-1] - diff(plotAt[1:2])/2-100)/2, y = output_2$`mass extinction Bayes factors`,
       col = "black",pch=19)
#points(x = c(plotAt[-1] - diff(plotAt[1:2])/2-100)/2, y = output_3$`mass extinction Bayes factors`,
#       col = "black",pch=1)

#legend(x=c(-30,-20), y=c(7,10), col=c("black", "grey80", "black"), pch=c(19, 19, 1),
#       legend = c("95% (91-99%)", "75% (50-93%)", "25% (6-52%)"),
#       cex=1,yjust=-5,
#       x.intersp = 0.25, y.intersp = 0.2, inset = -1, box.lty = 0)

abline(h = 2 * log(output_2$criticalBayesFactors[1:2]), lty = 2)
axis(at = c(round(1.2 * 50), axTicks(1)), side = 1)
axis(at = round(c(-1, axTicks(2)),2), las = 1, side = 2)
mtext(side = 1, text = "Time",line = 3)
mtext(side = 2, text = "Bayes Factors", line = 3)
text(-45,10,labels="d")
text(-25, 10, "75% (50-93%) Extinction")

#########
# E
plot.new()
plot.window(xlim = range((plotAt-100)/2), ylim = c(0,10))
#points(x = c(plotAt[-1] - diff(plotAt[1:2])/2-100)/2, y = thisOutput,
#       col = "black",pch=19)
#points(x = c(plotAt[-1] - diff(plotAt[1:2])/2-100)/2, y = output_2$`mass extinction Bayes factors`,
#       col = "black",pch=19)
# polygon(c(-7.5, -7.5,-10,-10), c(-1,9,9,-1), col = "#DDDDDD", border = "#DDDDDD")
# polygon(c(-6.5, -6.5,-7,-7), c(-1,9,9,-1), col = "#DDDDDD", border = "#DDDDDD")

points(x = c(plotAt[-1] - diff(plotAt[1:2])/2-100)/2, y = output_3$`mass extinction Bayes factors`,
       col = "black",pch=19)

#legend(x=c(-30,-20), y=c(7,10), col=c("black", "grey80", "black"), pch=c(19, 19, 1),
#       legend = c("95% (91-99%)", "75% (50-93%)", "25% (6-52%)"),
#       cex=1,yjust=-5,
#       x.intersp = 0.25, y.intersp = 0.2, inset = -1, box.lty = 0)

abline(h = 2 * log(output_3$criticalBayesFactors[1:2]), lty = 2)
axis(at = c(round(1.2 * 50), axTicks(1)), side = 1)
axis(at = round(c(-1, axTicks(2)),2), las = 1, side = 2)
```

```r
mtext(side = 1, text = "Time",line = 3)
mtext(side = 2, text = "Bayes Factors", line = 3)
text(-45,10,labels="e")
text(-25, 10, "25% (6-52%) Extinction")
```

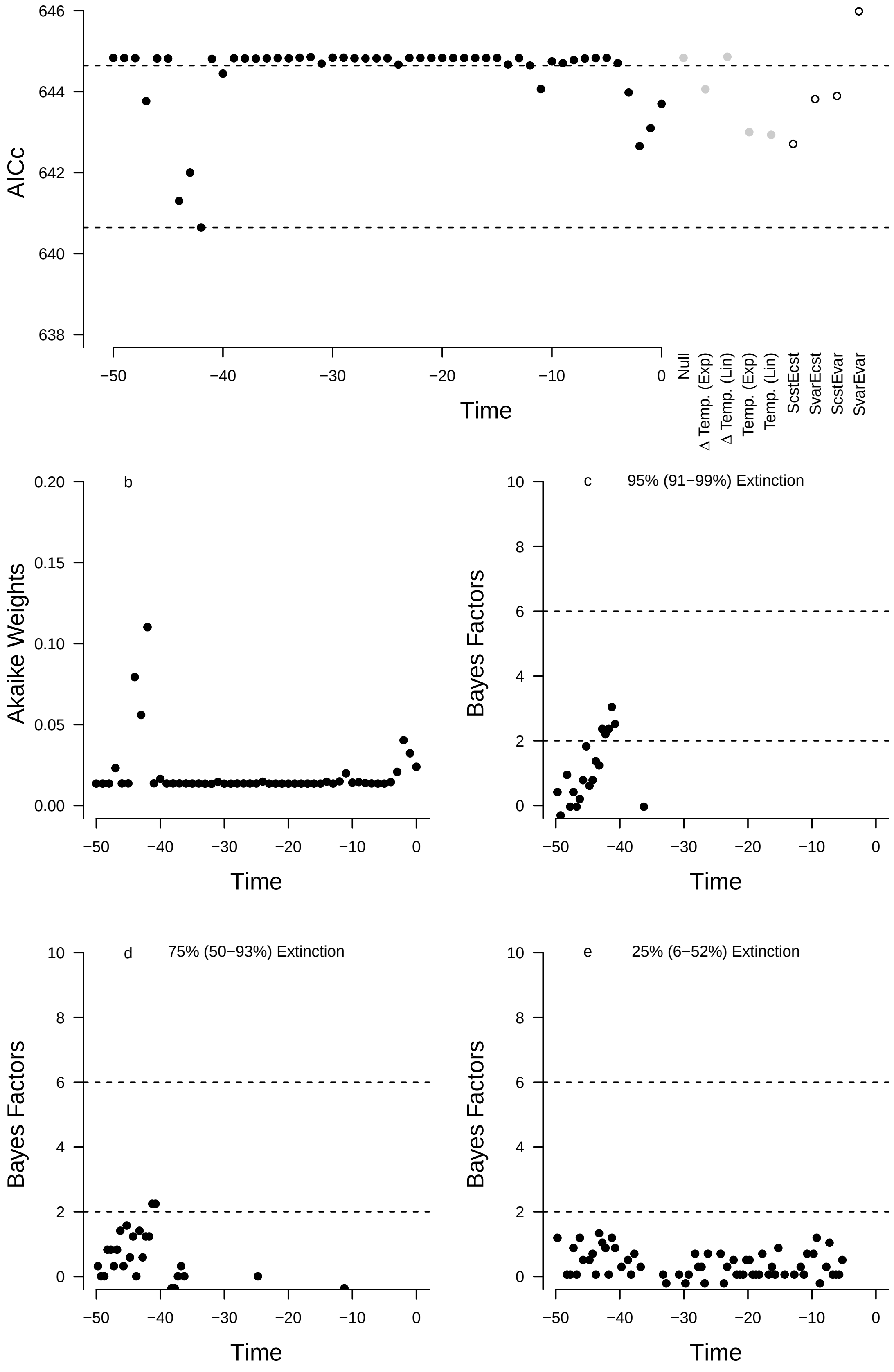

646
644
642
640
638
AICc
−50
−40
−30
−20
−10
0
Time
Null
Δ Temp. (Exp)
Δ Temp. (Lin)
Temp. (Exp)
Temp. (Lin)
ScstEcst
SvarEcst
ScstEvar
SvarEvar
b
Akaike Weights
0.20
0.15
0.10
0.05
0.00
c
95% (91−99%) Extinction
Bayes Factors
d
75% (50−93%) Extinction
e
25% (6−52%) Extinction
